# Photophysical Properties of Benzophenone-Based TADF Emitters in Relation to Their Molecular Structure

**DOI:** 10.1021/acs.jpca.1c08320

**Published:** 2022-01-21

**Authors:** Ekin Esme Bas, Pelin Ulukan, Antonio Monari, Viktorya Aviyente, Saron Catak

**Affiliations:** † Department of Chemistry, Bogazici University, Bebek, 34342 Istanbul, Turkey; ‡ Université de Lorraine and CNRS, LPCT UMR 7019, F54000 Nancy, France; § Université de Paris and CNRS, ITODYS, F75006 Paris, France

## Abstract

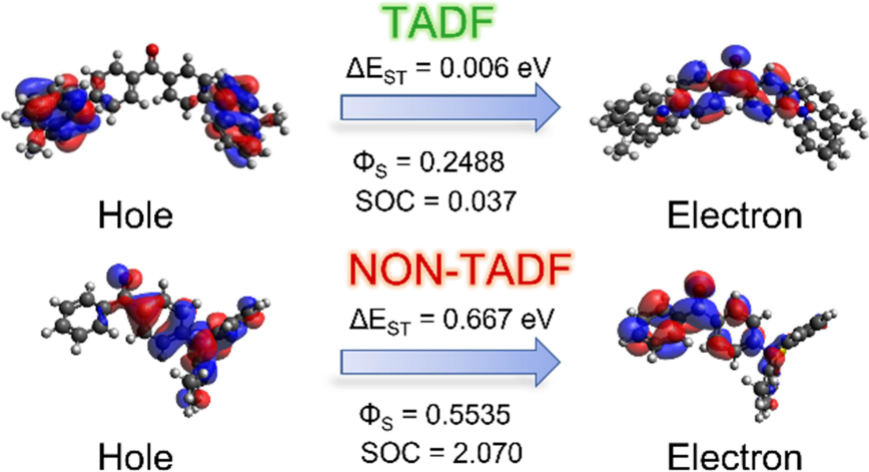

Thermally activated delayed fluorescence (TADF) materials are commonly used in various apparatus, including organic light-emitting device-based displays, as they remarkably improve the internal quantum efficiencies. Although there is a wide range of donor–acceptor-based compounds possessing TADF properties, in this computational study, we investigated TADF and some non-TADF chromophores, containing benzophenone or its structural derivatives as the acceptor core, together with various donor moieties. Following the computational modeling of the emitters, several excited state properties, such as the absorption spectra, singlet–triplet energy gaps (Δ*E*_ST_), natural transition orbitals, and the topological Φ_S_ indices, have been computed. Along with the donor–acceptor torsion angles and spin-orbit coupling values, these descriptors have been utilized to investigate potential TADF efficiency. Our study has shown that on the one hand, our photophysical/structural descriptors and computational methodologies predict the experimental results quite well, and on the other hand, our extensive benchmark can be useful to pinpoint the most promising functionals and descriptors for the study of benzophenone-based TADF emitters.

## 1 Introduction

**Figure 1:**
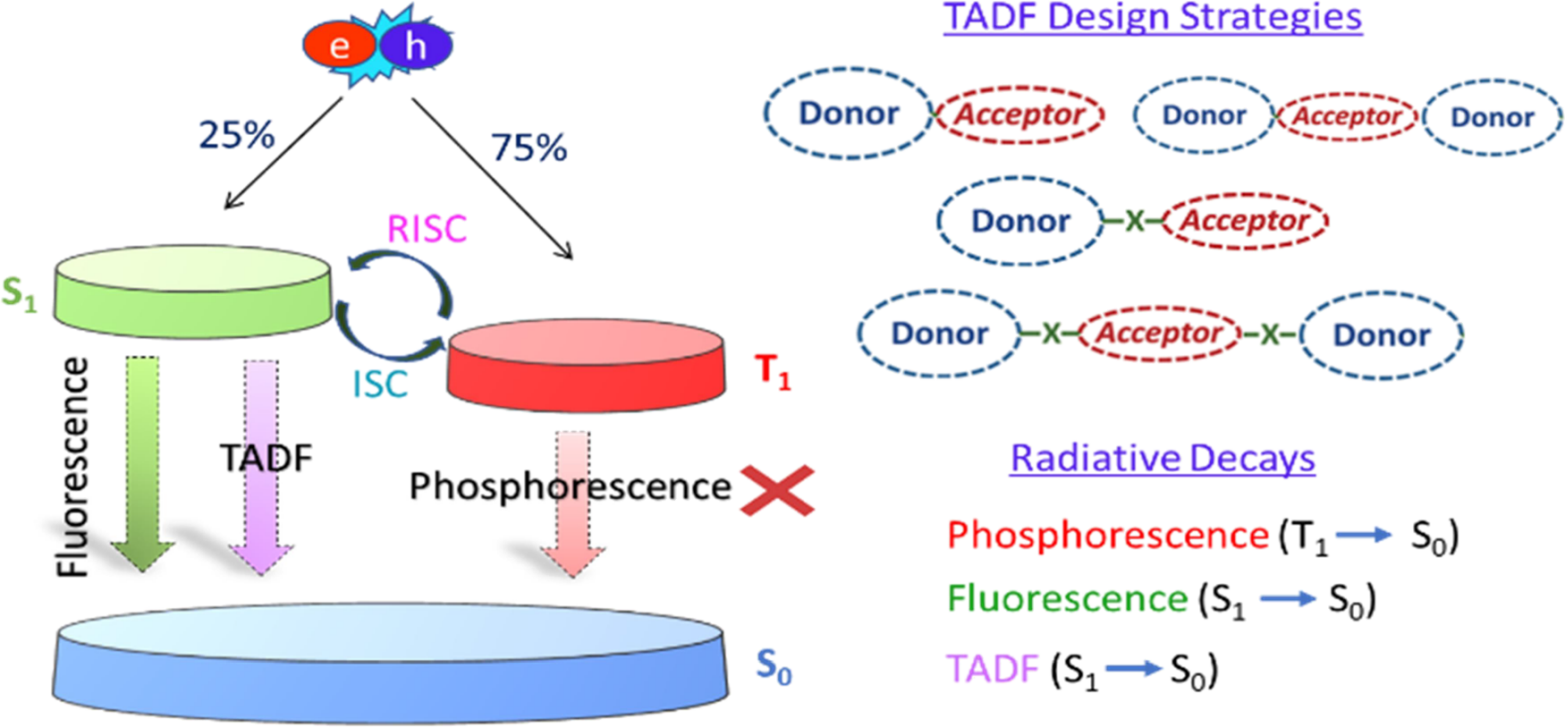
Schematic representation of ISC, RISC, TADF, and phosphorescence processes in a Jablonski diagram along with the possible design strategies for TADF emitters.

Organic light-emitting diodes (OLEDs) have attracted widespread attention since the invention of the first organic electroluminescent device in 1987.^1^ As compared to conventional LEDs and liquid crystal display systems, OLEDs do not require a backlight unit, as they are self-illuminating. Given this distinct feature, OLEDs offer several advantages, such as flexible device structures, decreased panel thickness, improved brightness, and reduced power consumption, rendering them most suitable for various devices.^2^ Nevertheless, OLEDs also display serious efficiency drawbacks, which are grounded in the fundamental spin statistics rule (Figure 1) because the population of the nonemissive triplet state comprises 75% of the generated excitons in the device, hence leading to the loss of two thirds of the applied energy.^3^ Phosphorescent organic light-emitting diodes (PhOLEDs), which contain heavy atoms, can improve the efficiencies of OLEDs with the help of enhanced intersystem crossing (ISC) and improved phosphorescence rates as a result of increased spin-orbit coupling (SOC).^4,5^ However, heavy metals instigate environmental issues while significantly increasing the cost of the device, thus limiting the commercial availability of PhOLEDs.^6^

Thermally activated delayed fluorescence (TADF) materials, first proposed by Adachi et al. in 2011, represent a most suitable alternative to overcome spin statistics burdens and achieve high internal quantum efficiencies (IQEs) in OLED displays.^7^ In TADF processes, the population of triplet excitons undergoes a slow reverse intersystem crossing (RISC), re-populating the emissive first excited singlet state (S_1_) and hence avoiding the inclusion of heavy metals. To assure efficiency, RISC or the upconversion of the triplet states [up-intersystem crossing (UISC)] require a small S_1_-T_1_ energy gap (Δ*E*_ST_). UISC can be achieved by promoting the population of intramolecular charge-transfer (ICT) states and is usually, albeit not necessarily, correlated with a small gap between the highest occupied molecular orbital and lowest unoccupied molecular orbital. To achieve efficient ICT, the molecular architecture requires the presence of a donor (D) moiety bridged to an acceptor (A) (Figure 1). The D and A groups can be separated by using bulky substituents to increase steric effects and maintain orthogonal molecular structures, hence minimizing conjugation and delocalization, or alternatively by π-bridges.^8,9^

Benzophenone is a widely used building block in the design of OLED devices, in part due to its strong and efficient electron accepting and efficient ultraviolet (UV) absorbing abilities, as well as its high ISC efficiency.^10−13^ Adachi and co-workers showed in 2014 that efficient deep blue TADF could be achieved by using benzophenone-based D-A-D frameworks.^12^ Thus far, many benzophenone-based TADF emitters possessing a small Δ*E*_ST_ have been reported to feature full-color delayed fluorescence emission, usually in a range from deep blue to green and with external quantum efficiencies up to 14.3%.^14^ Benzophenone-based luminogens can also be used to induce aggregation-induced delayed fluorescence.^15,16^

**Figure 2:**
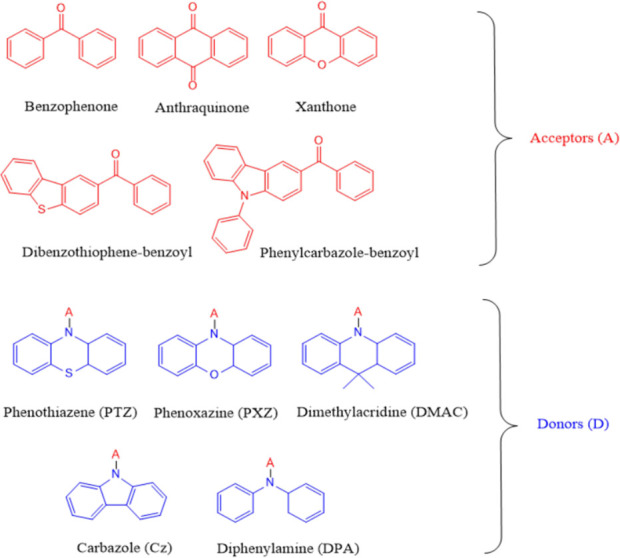
Electron-donating and -accepting moieties frequently used in the design of benzophenone-based TADF emitters.

Nonetheless, the rather floppy phenyl moieties of benzophenone itself may induce intramolecular rotations, which enhance nonradiative decay and lead to relatively low reverse ISC rates (*k*_RISC_).^17^ Hence, more compact and rigid benzophenone derivatives, such as anthraquinone,^18,19^ xanthone,^20−23^ dibenzothiophene-benzoyl^24,25^ and phenylcarbazole-benzoyl,^26^ are deemed more promising in terms of their TADF efficiencies. Figure 2 depicts the molecular structures of benzophenone derivatives commonly used in TADF materials, together with some of the most widely used D groups.

We used molecular modeling to elucidate the relationship between the molecular structure and optimal photophysical properties that are essential for effective TADF emission. Various descriptors are used to define TADF properties, which include Δ*E*_ST_, SOC, natural transition orbitals (NTOs), the topological Φ_S_ index quantifying the charge-transfer amount, and the torsion angles between D and A moieties.^27−33^ The oscillator strength (f) is also a critical parameter to attain a reasonable radiative decay rate (*k*_r_) from the S_1_ state to the ground state. The oscillator strength is usually closer to 0 for orthogonal donor–acceptor compounds, where CT is prominent.^29^ Negligible oscillator strengths represent a challenge in TADF emitters because it is essential to balance a small Δ*E*_ST_ and a sufficiently large *k*_r_ to reach high IQE in OLED devices.

In this study, a series of experimentally studied TADF emitters, employing benzophenone and its derivatives, have been investigated by quantum chemical calculations. Several non-TADF benzophenone chromophores have also been modeled for comparison. Absorption spectra of the selected compounds have been generated, including a sampling of the Franck–Condon region by Wigner distribution, to include dynamic effects. To determine the degree of charge separation in the excited state, NTOs and Φ_S_ indices have been computed, as well as the Δ*E*_ST_ values. NTO and Δ*E*_ST_ calculations have been performed on both the ground state (S_0_) and the lowest triplet state (T_1_) equilibrium geometries because geometrical reorganization can also play a role in photophysical processes. Furthermore, the SOCs between singlet and triplet excited states have been computed to better estimate RISC probability.

The mentioned descriptors have proven to be insightful in determining potential TADF efficiency, as well as shedding light on the correlation between molecular structure and TADF performance. Indeed, the investigation of the photophysical characteristics of both the excited and ground state geometries, and also the assessment of the DFT functionals for the excited state calculations of TADF emitters, may help provide a better understanding for the photophysical processes related to TADF at a molecular level.

## 2 Methodology

All ground state geometry optimizations have been performed using the Gaussian 16 program package,^34^ and a comprehensive conformational search has been carried out. UV–vis absorption spectra and Boltzmann-weighted Δ*E*_ST_ calculations (Tables S1 and S2) showed that different conformations did not exhibit significant differences in their excitation energies and on the ordering of the excited states. Hence, T_1_ geometry optimizations and related excited state calculations were carried out solely on the most stable conformer. The M06-2X functional^35^ has been used together with the 6–31 + G(d,p) basis set for S_0_ and T_1_ geometry optimizations. This choice is justified because M06-2X is well known to correctly reproduce medium-range interactions, electronic energies, and equilibrium geometries of compounds with aromatic ring systems.^35^ However, a dispersion-corrected functional, B3LYP-D3, was also tested for the geometry optimization of rather larger compounds.^36^ In order to increase the accuracy, 6–311++G(3df,3pd)^37^ and 6–311++G(2d,2p)^38^ basis sets have been used for compounds including sulfur and phosphorus, respectively. Calculations have been performed taking into account the experimentally employed solvents by the polarizable continuum model in the integral equation formalism (IEF-PCM). CYLview software package has been used for visualization purposes.^39^

Similar to ground state calculations, excited state calculations have been carried out by using Gaussian 16 software package. Tamm–Dancoff approximation (TDA) has been used, as this approach avoids unphysically stable triplet states in CT molecules while maintaining a good description of the singlet excited states, hence yielding more accurate and balanced results for Δ*E*_ST_ calculations.^40,41^ The absorption spectra as well as the energies of the lowest lying singlet and triplet excited states have been computed with different functionals (B3LYP,^42−45^ BLYP,^42,45,46^ PBE0,^43,45,47,48^ M06-2X,^42,45,49^ CAM-B3LYP,^42,45,50,51^ and LC-ωPBE^52−54^) and 6–31 + G(d,p) basis set, and the performances of these functionals have been evaluated with respect to experimental data. Absorption spectra have been modeled, including the effects of vibrational and thermal motion via Wigner distribution sampling of the equilibrium region on the potential energy surface by generating 40 conformations via the Newton-X program^55^ on the equilibrium region of the PES.

NTOs and Φ_S_ indices have been calculated for the S_1_ state by using the Gaussian 16 and Nancy_EX program packages,^56^ while hole and electron NTOs have been visualized with the Avogadro program package.^57^ Φ_S_ index can be defined as the spatial overlap between attachment and detachment densities. Values closer to 1 indicate the presence of local excitation character, whereas values approaching 0 imply that the CT character is dominant.^56^ SOC values between S_1_ and T_1_, and in some cases between S_1_ and T_2_, have been calculated using the Amsterdam Density Functional software package by utilizing a DZP basis set.^58^

## 3 Results and Discussion

**Figure 3:**
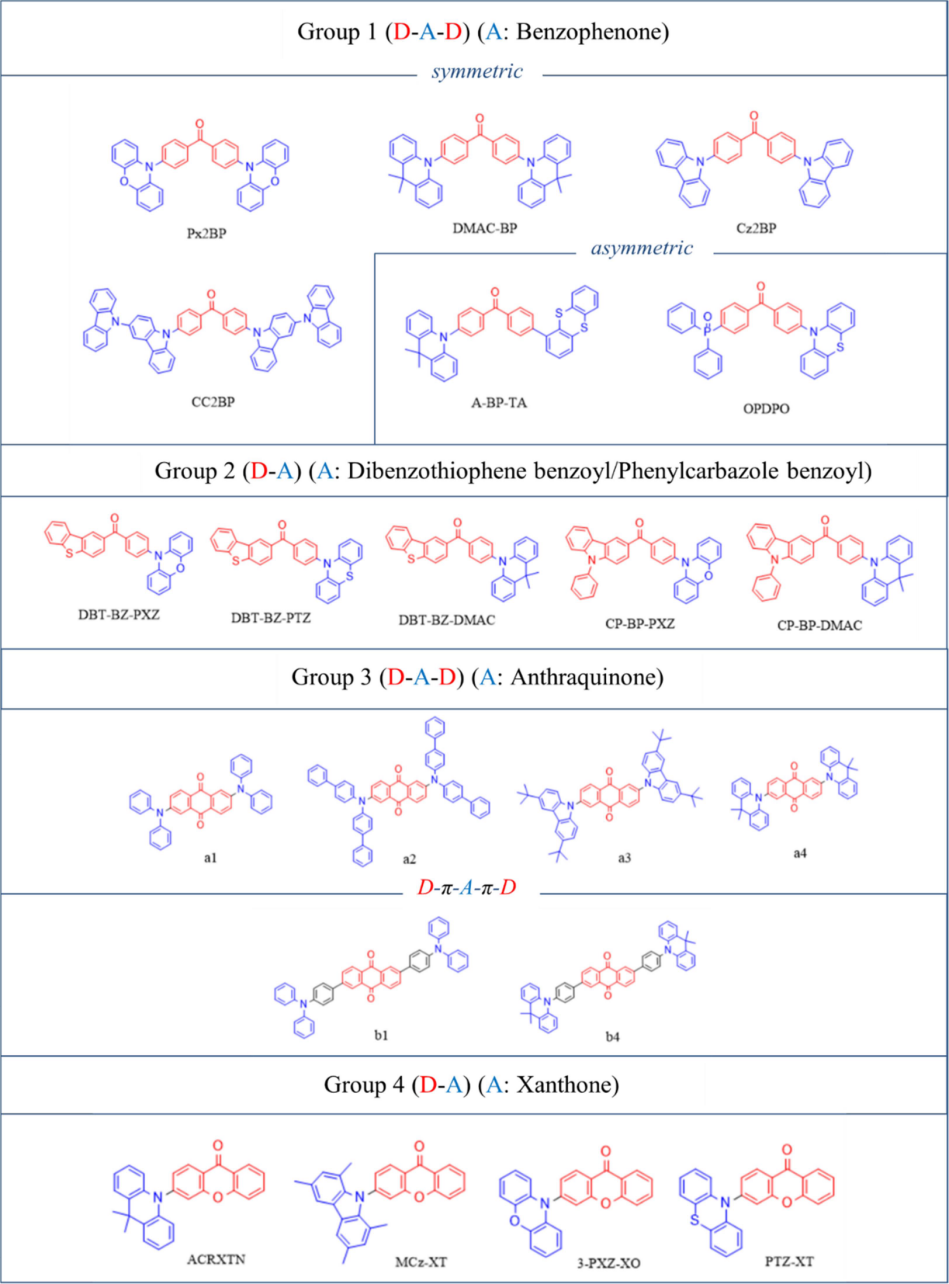
Classification of TADF emitters modeled in this study.

The benzophenone emitters investigated in this study have been grouped according to the type of A moieties used. As depicted in Figure 3, Group 1 emitters are in the form of D-A-D, and they contain benzophenone A cores (in red) bridged with various electron-donating groups. They can be further subdivided into two subgroups: symmetric and asymmetric emitters. Symmetric emitters Px2BP,^12^ DMAC-BP,^59^ Cz2BP,^12^ and CC2BP^12^ contain phenoxazine (PXZ), dimethylacridine (DMAC), and carbazole (Cz) donors (in blue), respectively. Asymmetric emitters, A-BP-TA^60^ and OPDPO,^61^ include D groups such as thianthrene, phenothiazene (PTZ), and diphenylphosphineoxide.

Group 2 emitters have fused D-A ring structures where DBT-BZ-PXZ,^25^ DBT-BZ-PTZ,^25^ and DBT-BZ-DMAC^24^ bear the A unit dibenzothiophene-benzoyl (in red), while CP-BP-PXZ^26^ and CP-BP-DMAC^26^ have the A unit of phenylcarbazole-benzoyl (in red). D groups such as PXZ, PTZ, and DMAC have been employed in these emitters as well.

Group 3 emitters have para-substituted structures in which the A unit is anthraquinone.^18^ These include D-A-D type emitters a1-a4, in which the D units are diphenylamine (DPA), bis(4-biphenyl)amine, 3,6-di-tert-butylcarbazole, and DMAC, respectively. Similarly, in the D-π-A-π-D structures, b1 and b4, the D groups are DPA and DMAC.

Group 4 emitters have D-A type of structures in which the A unit is xanthone. DMAC, PXZ, and PTZ donors have been used in ACRXTN,^23^ 3-PXZ-XO,^20^ and PTZ-XT,^62^ respectively. In MCz-XT,^21^ 1,3,6,8-tetramethylcarbazole is present as the D unit.

**Figure 4:**
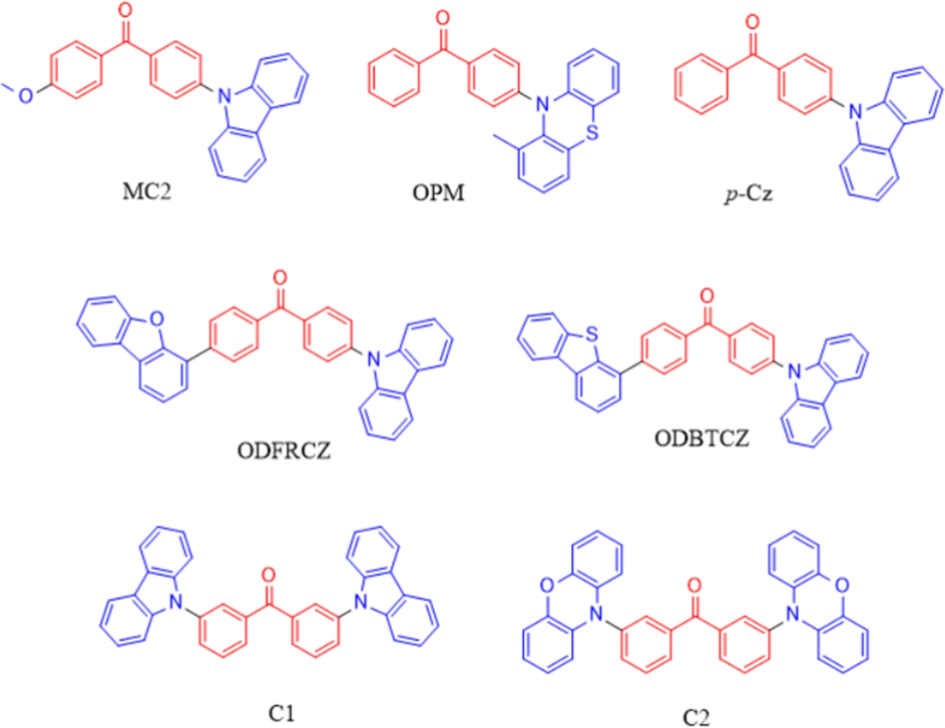
Non-TADF benzophenone emitters.

Finally, seven non-TADF compounds have been selected from literature in an attempt to elucidate the main structural differences leading to TADF emission (Figure 4). While some of these non-TADF emitters have D-A type structures, including MC2,^63^ OPM,^64^ and p-Cz,^65^ others (ODFRCZ,^66^ ODBTCZ,^67^ C1,^15^ and C2^15^) possess D-A-D type of structures.

### 3.1 Benchmark Calculations and UV–vis Absorption Spectra

M06-2X/6–31 + G(d,p) and B3LYP-D3/6–31 + G(d,p) methods have been tested for the ground state geometry optimizations of a series of compounds selected from each group. Table S1 depicts the optimized geometries at both levels of theory, and Table S2 includes the D-A torsion angles for the optimized geometries. Tables S1 and S2 suggest that there is no significant change in the geometries optimized at both levels as both functionals yielded similar structures with very close D-A torsion angles. Because the compounds investigated in this study are relatively small in which long-range interactions are not so dominant, we decided to continue with M06-2X/6–31 + G(d,p) for the geometry optimizations because the latter is computationally more affordable.

A series of widely used DFT functionals have been chosen for excited state calculations, BLYP, B3LYP, PBE0, and M06-2X. More specifically, UV–vis absorption spectra have been generated for a group of emitters, and the results have been compared with the available experimental spectra.

The conformational space has been taken into account in the modeling of four compounds (Px2BP, Cz2BP, DBT-BZ-PXZ, and DBT-BZ-DMAC) (Table S1). Their absorption spectra have been obtained as the union of the spectra of all the conformers according to their Boltzmann weights. The merged spectra have then been compared to those obtained from the most stable conformation only. Table S3 clearly demonstrates that weighted conformations exhibit similar photophysical features and spectral shapes as the most stable conformation, the maximum deviation being around 3 nm for B3LYP (DBT-BZ-PXZ), 4 nm for PBE0 (DBT-BZ-PXZ), and 2 nm for M06-2X (Cz2BP). In addition to the UV–vis absorption spectra, different conformations of Cz2BP and CC2BP have very similar Δ*E*_ST_ values, and the Boltzmann-weighted Δ*E*_ST_ values of Cz2BP and CC2BP were found to be in good agreement with the experimental findings (Table S4). Thus, henceforth, the most stable conformer will solely be considered. In Tables S5–S7, the absorption spectra for Group 1 emitters and three emitters from Group 2 have been computed by using BLYP, B3LYP, PBE0, and M06-2X. The results suggest that the spectra obtained with M06-2X exhibit hypsochromic shift as compared to the experimental spectrum.

As opposed to M06-2X, BLYP produced bathochromic shifts and lower Δ*E*_ST_ values (energies are given in Table S8; histogram charts are depicted in Figures S1 and S2) when compared with the experimental data. This behavior is most probably due to well-known unphysical stabilization of CT states by LDA functionals.^68^ In fact, hybrid B3LYP and PBE0 functionals yield the best agreement with experimental spectra and Δ*E*_ST_. In order to further confirm this finding, two long-range corrected functionals, CAM-B3LYP and LC-ωPBE, were tested for selected compounds. Table S9 depicts the Δ*E*_S1-T1_ values (eV) calculated with BLYP, B3LYP, PBE0, CAM-B3LYP, and LC-ωPBE for the S_0_ and T_1_ optimized geometries of the compounds. Similar to M06-2X, the Δ*E*_ST_ values calculated with the long-range corrected CAM-B3LYP and LC-ωPBE functionals are significantly higher as compared with experiment and the other functionals. It is also noteworthy that BLYP gave results consistent with the experiment for a few molecules (Px2BP and DMAC-BP). This is probably due to the fact that, due to the relatively small size of the compounds, the CT states are not long-range and hence can be correctly reproduced by hybrid functionals as already observed for instance in some organometallic compounds, hence outperforming medium- or long-range corrected functionals.^69^

In an attempt to take into account the role of excitation on the molecular geometry, the Δ*E*_ST_ values have been computed from the S_0_, S_1_, and T_1_ geometries with B3LYP, PBE0, and BLYP functionals for a series of compounds, and the results have been reported in Table S10. The results clearly indicate that the Δ*E*_ST_ values computed from the S_0_ and S_1_ geometries are usually very similar. This can be explained by the similar molecular structures obtained for S_0_ and S_1_ geometries (Figure S3), as TADF efficiency relies on the molecular geometry to a great extent. Also, considering the computational cost of S_1_ geometry optimizations, for the rest of the calculations, the photophysical properties have been calculated solely with the S_0_ and T_1_ geometries.

The absorption spectra of all other molecules have hence been calculated with BLYP, B3LYP, and PBE0 functionals (Figures S4–S8). From the results, it can be deduced that compounds with more rigid electron-donating groups such as PXZ, PTZ, and DMAC possess a broadened band (∼350 to 500 nm) appearing after the high intensity local excitation band (∼300 nm), which can be ascribed to the presence of CT states.^70−72^ Nevertheless, the reasonably rigid emitters of Group 4 also have blue-shifted absorption spectra, which is most likely due to the less efficient nature of the specific D-A type architecture. Indeed, the presence of a unique D unit might decrease the CT character of the electronic transitions, and coherently reduced T_1_-S_1_ upconversion efficiency has been previously reported for some D-A type emitters.^73^

Oscillator strengths calculated with B3LYP, PBE0, and BLYP functionals for the transitions from S_0_ to S_1_, and the reorganization energies between S_0_ and S_1_ geometries have also been reported in Tables S11 and S12, respectively. While the latter remain low to moderate and are between around 15 and 6 kcal/mol, no systematic behavior between the different classes of molecules can be underlined, thus hampering the use of this parameter to preview TADF efficiency. Low to moderate geometrical distortion and reorganization energies may also be another reason for the very close Δ*E*_ST_ values calculated from the S_0_ and S_1_ geometries. The oscillator strengths were calculated to be near-zero for most of the TADF emitters with very low Δ*E*_ST_ values and Φ_S_ indices, for which we assume that the CT characteristics are more pronounced. As is the case in this study, it was previously reported that the oscillator strengths computed at the TDA or full TD-DFT level might be erroneous, especially for the transitions involving CT states.^74^

### 3.2 D–A Torsion Angles

Several computational TADF descriptors were assessed in order to analyze and investigate the structural and photophysical phenomena related to TADF emissions. One of the most critical TADF descriptors is the torsion angle between the D and A units. The torsion angle must be close to 90° in an ideal TADF emitter because an effective CT configuration can be achieved by spatially separating the hole and electron densities and by breaking the conjugation pattern.^75^

**Figure 5:**
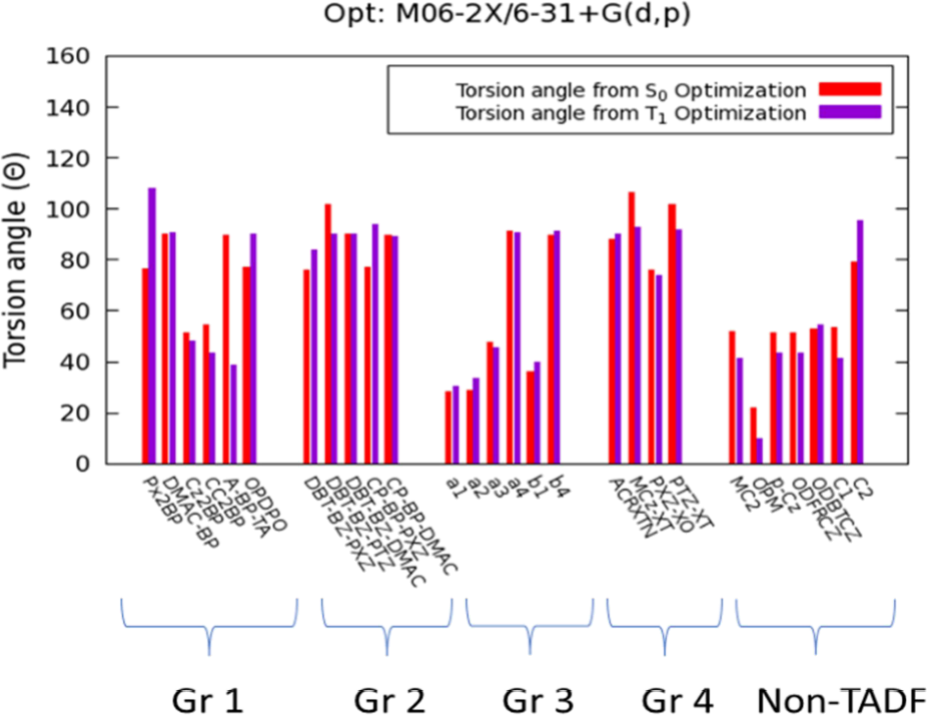
Donor–acceptor torsion angles for the S_0_ and T_1_ optimized geometries of the investigated emitters shown with stacked histograms (M06-2X/6–31 + G(d,p)).

The S_0_ and T_1_ optimized geometries of all emitters and the conformation energies (M06-2X/6–31 + G(d,p)) are given in Supporting Information (Tables S13–S17). Figure 5 demonstrates the torsion angles measured for all emitters investigated in this study.

Because symmetrical emitters have similar torsion angles for each D–A bond, only one of the torsion angles has been shown in Figure 5, while numerical values for torsion angles depicted in Figures S9–S13 are reported in Table S18. For asymmetrical emitters, the torsion angle of the electron-donating unit has been ascribed based on the NTO analysis discussed in the following sections. Similarly, if the electron–hole density is localized on a different electron-donating unit in the T_1_ equilibrium geometry, the corresponding torsion angle has been considered for the analysis of the T_1_ equilibrium geometries.

It is evident that the emitters containing rigid electron-donating moieties, including DMAC, PXZ, and PTZ, have significantly higher D–A torsion angles. While reasonably high torsion angles have been observed for Group 1, Group 2, and Group 4, Group 3 emitters bear relatively low torsion angles approximately in the range of 30–35°. This feature can be due to the presence of less rigid DPA donors whose low steric hindrance may lead to highly planar structure with extended π-conjugation patterns. The highest torsion angles belong to a4 and b4 among Group 3 emitters due to the presence of DMAC electron donors in these groups. Indeed, the additional dimethyl units in the acridine structure impose sterical constraints that restrict the free rotation of the D. Carbazole (Cz)-containing compounds also exhibit low torsion angles, around 55°, which are slightly higher than that for the DPA-bearing compounds. Interestingly, the majority of the non-TADF compounds, where the D unit is Cz, have notably lower torsion angles, along with two compounds from Group 1 emitters (Cz2BP and CC2BP). The torsion angles measured for Group 4 emitters are satisfactorily high and close to 90° regardless of the electron-donating unit. This situation can be attributed to the rigid xanthone skeleton that fixes the D–A torsion angle at a desirable position. This observation is in line with the study of Kreiza et al.^17^ Nonetheless, the obtained results suggest that such a descriptor can only provide a partial understanding of TADF efficiency because Group 3 compounds, which are known as TADF emitters, possess very low torsion angles, despite the presence of the rigid anthraquinone A. Hence, a finer analysis of the property and reorganization of the electronic density should be considered to improve TADF efficiency predictions.

While in most cases the torsion angles are similar for both T_1_ and S_0_ equilibrium geometries, there is a sharp decrease in the torsion angle for T_1_ equilibrium geometry in A-BP-TA. Indeed, the hole density in the triplet state is localized on the less rigid electron D thianthrene, causing a rather important planarization of the global structure. This will be better understood in the following section.

### 3.3 NTOs and Φ_S_ Indices

NTOs and Φ_S_ indices have been calculated for all emitters in an attempt to determine the electron–hole density reorganization. Accordingly, electron and hole NTOs describing electronic density reorganization in the S_1_ state are shown in Tables S19–S28. Only the NTOs generated from B3LYP/6–31 + G(d,p) densities are given because no remarkable difference has been observed with BLYP and PBE0. Φ_S_ values describing the spatial overlap between attachment and detachment densities^76^ are also reported in Tables S29–S33.

In general, the overlap between electron and hole densities has been observed to be high for compounds bearing low D-A torsion angles. This is expected because low torsion angles cause high degrees of π-delocalization and a corresponding planarization of the molecular core. As a result, compounds bearing Cz and DPA donors, including Cz2BP, CC2BP, a1, a2, a3, b1, and most of the non-TADF compounds, show highly overlapping hole and electron NTOs. Interestingly, in non-TADF emitters ODBTCZ and ODFRCZ, the highly flexible dibenzothiophene and dibenzofuran units did not show electron-donating ability at S_0_ or T_1_ equilibrium geometries. Obviously, because high Φ_S_ indices are indicative of spatially overlapping densities, the corresponding excited states have a more prominent local excitation character. On the other hand, emitters with more rigid D units (DMAC, PXZ, and PTZ) usually have low amounts of overlap between their NTOs, and consequently small Φ_S_ values, even in the presence of small torsion angles. Hence, even if more computationally expensive, the explicit analysis of the excited state density is much more informative in predicting TADF potential.

**Figure 6:**
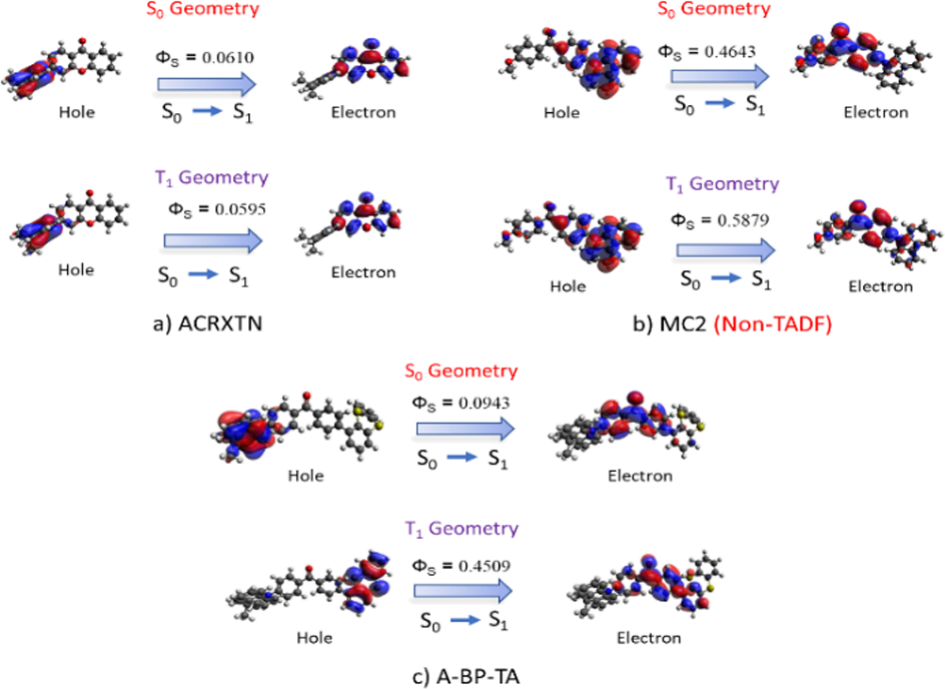
Hole–Electron levels and Φ_S_ indices calculated with B3LYP/6–31 + G(d,p) for the molecules (a) ACRXTN, (b) MC2, and (c) A-BP-TA.

It is also noteworthy that the structural reorganization may have an undeniable impact on the amount of CT from the accessible excited states, as in the case of excited state twisting.^77^ Indeed, some compounds, particularly those containing butterfly-shaped PXZ, PTZ, or thianthrene units, underwent drastic changes; more specifically, T_1_ equilibrium geometries exhibited planarization in the butterfly-shaped moieties (Table 1). This, in turn, is related to a change in the shape of the NTOs obtained from the T_1_ equilibrium geometries. The change in the NTO distribution is indicative of the fact that geometric relaxation leads, for some compounds, such as Px2BP, A-BP-TA, and b1, to the population of a different diabatic state. The most obvious change in the NTO localization pattern is seen in A-BP-TA (Figure 6), where at T_1_ equilibrium geometry, the hole NTO is localized mostly on the now planar thianthrene unit instead of the rigid DMAC unit. This effect also produces an increase in the Φ_S_ indices at the T_1_ equilibrium geometry for Px2BP and its meta-substituted analogue C2 (Tables S19 and S28). The electron NTO localizes only on one of the PXZ units, which becomes planar at T_1_ equilibrium geometry; this feature is also indicative of a consistent pattern, which is observed in two other similar cases. In the π-bridged anthraquinone-based b1 and b4, similar changes have been observed in the transition from S_0_ to T_1_ equilibrium geometry (Table S24). These consist of slight rotations in the bridging phenyl links, which cause disruptions in orthogonality and give rise to an increased electron delocalization. For a better visualization, the hole and electron NTOs and Φ_S_ indices, calculated at T_1_ and S_0_ equilibrium geometries for two TADF and one non-TADF compounds, are shown in Figure 6.

**Table 1:**
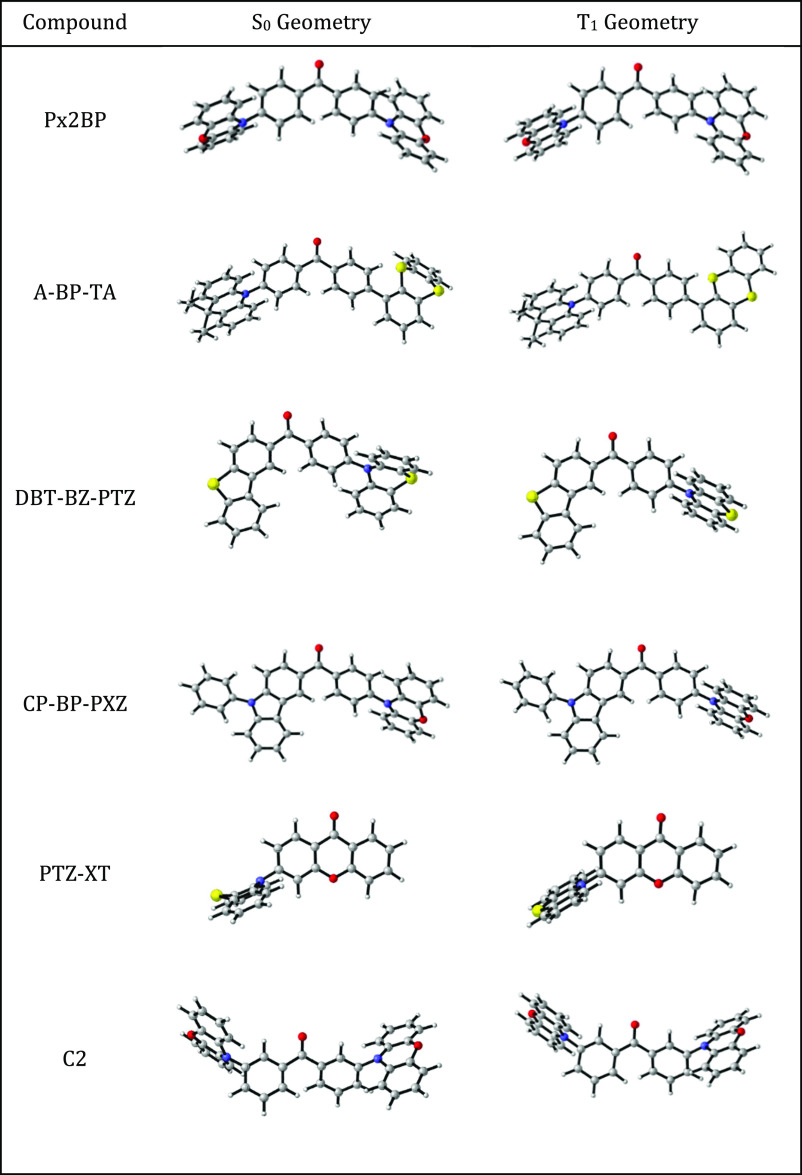
S_0_ and T_1_ Geometries of Selected Emitters with Butterfly-Shaped Electron Donors

**Figure 7:**
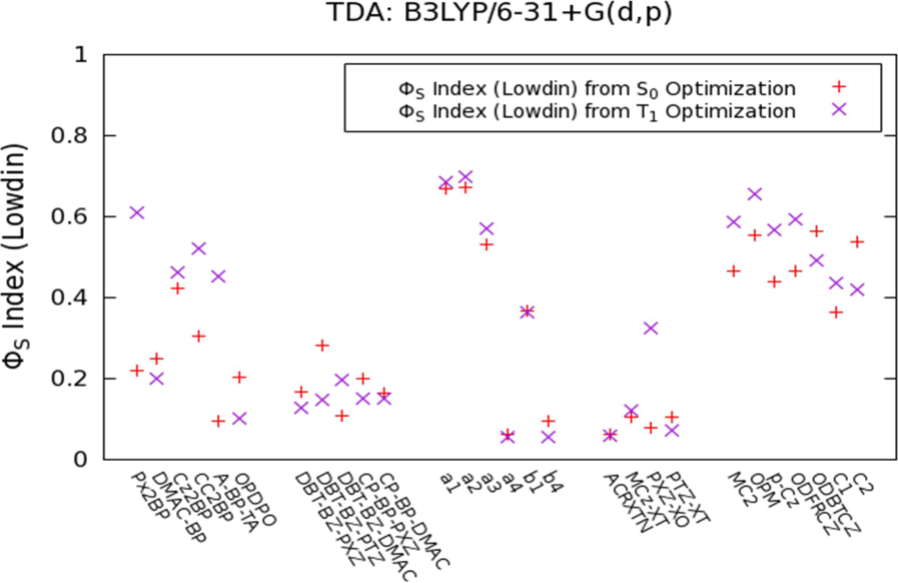
Φ_S_ indices for the TADF emitters calculated from S_0_ and T_1_ geometries with B3LYP/6–31 + G(d,p).

As expected, non-TADF compounds exhibit higher Φ_S_, which are due to the presence of weaker electron donors like Cz, dibenzofuran, and dibenzothiophene. However, despite the fact that the only noticeable structural difference is related to the position of the PXZ substituents, the meta-substituted non-TADF C2 also exhibits higher Φ_S_ indices compared to its para-substituted analogue, Px2BP, in line with its classification as a non-TADF compound. Φ_S_ indices calculated with B3LYP for all compounds are depicted in Figure 7, and the Φ_S_ indices calculated with PBE0 and BLYP are given in Figures S14–S15. TADF behavior is clearly inferred from these values, hence making this descriptor quite promising for the discrimination and prediction of optical properties with TADF potential.

### 3.4 Singlet–Triplet Energy Gaps (Δ*E*_ST_)

The energy difference between the first singlet and triplet excited states is an important descriptor for predicting the possibility of TADF emission because the upconversion of T_1_ to S_1_ can be achieved only if the energy gap is low enough to be overcome by thermal energy. According to previous studies, Δ*E*_ST_ values above 0.3 eV usually decrease the likelihood of the RISC process, whereas the upconversion of the triplet becomes more likely if the Δ*E*_ST_ value is below 0.1 eV.^5^

**Figure 8:**
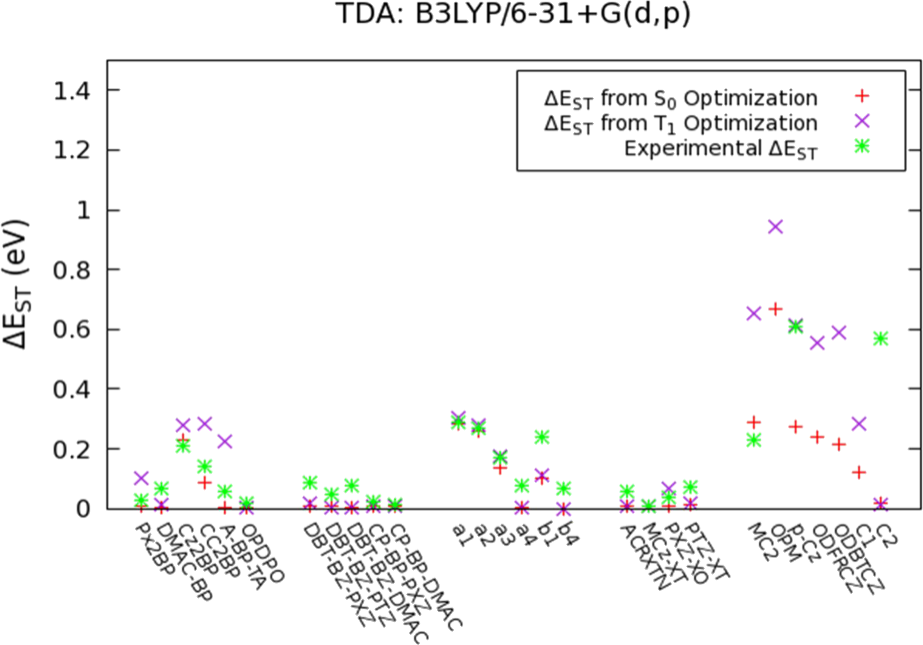
Δ*E*_ST_ values for TADF emitters calculated from S_0_ and T_1_ geometries with B3LYP/6–31 + G(d,p). (Experimental Δ*E*_ST_ values also included).

Even though the RISC process usually takes place from the T_1_ state, upper lying triplet excited states may also influence the efficiency of RISC if they are in close proximity to T_1_. Spin-vibronic coupling may cause state mixing between triplet energy levels, giving rise to strongly coupled S_1_ and T_1_ states. Therefore, the strong impact of internal conversion on ISC and RISC processes cannot be underestimated.^78^ Thus, the energy differences between S_1_-T_2_ (Δ*E*_S1-T2_) states have also been reported along with the Δ*E*_S1-T1_ values when the T_2_ state lies below S_1_ for any given compound. The calculated values together with the experimental findings are reported in Tables S34–S38 and in Figure 8.

Figure 8 depicts the Δ*E*_S1-T1_ values calculated with B3LYP for almost all groups except for Group 3 because Δ*E*_S1-T2_ values calculated for them are in a better agreement with the experimental findings. It is evident that the lowest Δ*E*_ST_ values have been obtained from the BLYP calculations, which is consistent with the lowest Φ_S_ indices computed with this functional. On the other hand, Δ*E*_ST_ values calculated with B3LYP and PBE0 are closer to the experimental ones. Overall, the calculated Δ*E*_ST_ values are usually consistent with the experimental observation, especially for the S_0_ geometries. The Δ*E*_ST_ values calculated with PBE0 and BLYP are also given in Figures S16 and S17.

It is noteworthy that Δ*E*_ST_ values are closely correlated with torsion angles and Φ_S_ indices because the compounds possessing low torsion angles and high Φ_S_ indices, such as Cz2BP, CC2BP, most of the Group 3 compounds, and non-TADF compounds, also have relatively higher Δ*E*_S1-T2_ and in some cases even high Δ*E*_S1-T1_ values. Freely rotating donors DPA and sterically less hindered donors Cz lead to higher Δ*E*_ST_ values because of enhanced geometrical relaxation. Despite having DPA donors, b1 has been observed to possess relatively low Δ*E*_ST_ values, which may have been acquired through the phenyl bridges that lead to a higher spatial separation of electron and hole densities. Also, as expected, emitters with DMAC, PXZ, or PTZ donors have satisfying Δ*E*_ST_ values usually at both S_0_ and T_1_ equilibrium geometries due to the induced orthogonality. There is a sharp increase in the Δ*E*_ST_ values obtained from the T_1_ equilibrium geometry of A-BP-TA, where the electron-donating group is the less rigid thianthrene moiety. This is also consistent with the NTO calculations previously mentioned, pointing out the close relationship between Δ*E*_ST_ and electron–hole separation. This result for A-BP-TA also further proves the reliability of NTO calculations and Φ_S_ index in elucidating the excited state properties by relating them to the molecular structure.

Almost all of the non-TADF compounds have high Δ*E*_ST_ values due to higher amounts of density overlap introduced by freely rotating D–A moieties, which planarize to enhance conjugation. However, C2 is an exception as it exhibits notably lower Δ*E*_ST_ values in spite of its high Φ_S_ index and overlapping NTOs. The calculated Δ*E*_ST_ value is also lower as compared with its experimental Δ*E*_ST_ value, clearly identifying this compound as an outlier. Still, out of 28 benzophenone derivatives investigated in this study, C2 is the only emitter in which an inverse relationship between Δ*E*_ST_ values and Φ_S_ indices was observed, and this can also be resulting from the insufficiency of our methodology in reproducing excited state energies for this compound.

### 3.5 Spin-Orbit Couplings

**Figure 9:**
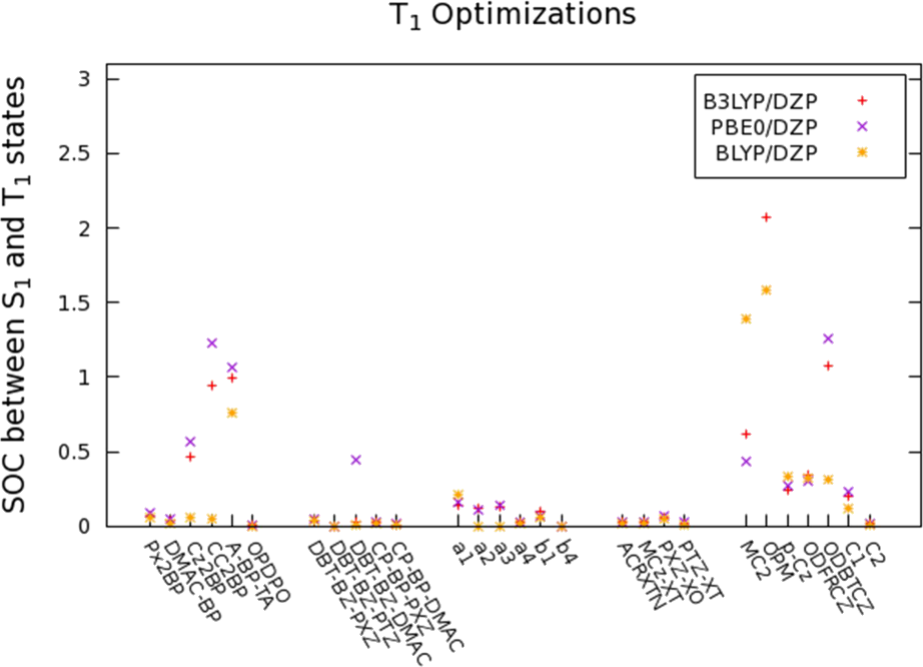
SOC values for TADF emitters calculated with B3LYP, BLYP, and PBE0 using the T_1_ geometries.

The last descriptor analyzed in this study is the SOC. The mixing of the wavefunctions of singlet and triplet energy levels are indeed strongly dictated by SOC.^79^ Therefore, by performing SOC calculations, crucial information regarding the feasibility of ISC or RISC processes is obtained. Indeed, SOC matrix elements significantly different from zero are necessary to attain RISC and, hence, TADF emission. However, SOC usually increases with atomic number (Z); thus small organic chromophores are usually less prone to ISC and RISC as compared to heavy-metal containing complexes.^80^ Additionally, the coupling of CT states produce negligible SOC values as a consequence of El-Sayed’s rule, which highlights the need for local triplet excited states for an effective RISC process.^79^ Efficient TADF emitters usually have lower SOC values due to their orthogonal D–A type molecular backbones, where CT character is more pronounced; this unfavorable factor is, however, compensated by small singlet–triplet energy gaps, which are inversely proportional to the (R)ISC probability. Hence, SOC between the S_1_ and T_1_ states of TADF emitters could also indicate the presence of CT states. Because we are dealing with RISC, calculations were solely performed on equilibrium T_1_ geometries. SOC between S_1_ and T_2_ states have also been computed from the T_1_ geometries if the T_2_ lies below the S_1_ state, in a similar approach adopted for Δ*E*_ST_ calculations. The calculated SOC values have been reported in Tables S39–S43. For a better comprehension, the SOC values calculated with B3LYP, BLYP, and PBE0 functionals are shown in Figure 9.

The results indicate that the compounds possessing lower Δ*E*_ST_ values and Φ_S_ indices, such as most of Group 1 emitters (Px2BP, DMAC-BP, and OPDPO), Group 2 emitters, a4 and b4, and Group 4 emitters, exhibit extremely low SOC values as a result of the higher CT character of their S_1_ and T_1_ geometries.

Higher SOC values have been obtained for compounds with less steric hindrance, including Cz2BP, CC2BP, most of Group 3 emitters, and all non-TADF emitters, because local excitations become more favorable in these emitters. A-BP-TA also exhibits relatively high SOC values because its hole density becomes localized on the flexible thianthrene unit in its T_1_ geometry, reducing the CT character in a similar manner observed in its Δ*E*_ST_ values and Φ_S_ index. Additionally, higher SOC constants obtained for S_1_-T_2_ interactions may indicate that the triplet upconversion might be taking place from the T_2_ rather than the T_1_ state.

Table 2 summarizes the D–A torsion angle, Φ_S_ index, Δ*E*_ST_, and SOC results of the TADF and non-TADF emitters investigated, showing a consistent behavior for all descriptors studied, pointing toward the successful accuracy of NTOs and Φ_S_ in inferring the TADF behavior. More importantly, in most cases, the TADF capability may be correctly predicted by calculating the excited state indicators at the Franck–Condon geometry, hence avoiding rather expensive excited state optimizations.

**Table 2:** D–A Torsion Angles (°), Φ_S_ Indices (Lowdin Charge Population), Δ*E*_ST_ Values (eV), and SOC Constants for the TADF and Non-TADF Emitters Calculated from the T_1_ Geometries (TDA: B3LYP/6–31 + G(d,p))

group	compound	torsion angle	Φ_S_ index	Δ*E*_ST_	SOC
group 1	Px2BP	107.90	0.6097	0.104	0.073
	DMAC-BP	90.91	0.1991	0.013	0.037
	Cz2BP	48.19	0.4603	0.278	0.465
	CC2BP	43.33	0.5206	0.284	0.942
	A-BP-TA	38.90	0.4509	0.225	0.999
	OPDPO	90.06	0.1009	0.004	0.004
group 2	DBT-BZ-PXZ	83.84	0.1279	0.018	0.042
	DBT-BZ-PTZ	90.21	0.1480	0.005	0.002
	DBT-BZ-DMAC	90.11	0.1967	0.004	0.026
	CP-BP-PXZ	93.80	0.1494	0.011	0.026
	CP-BP-DMAC	88.90	0.1514	0.008	0.009
group 3	a1	30.54	0.6839	0.481	0.144
	a2	33.32	0.6992	0.428	0.120
	a3	45.42	0.5689	0.272	0.134
	a4	90.67	0.0546	0.007	0.025
	b1	39.68	0.3627	0.346	0.097
	b4	91.00	0.0544	0.001	0.000
group 4	ACRXTN	90.21	0.0595	0.008	0.025
	MCz-XT	92.89	0.1199	0.011	0.026
	3-PXZ-XO	74.04	0.3243	0.066	0.060
	PTZ-XT	91.49	0.0721	0.018	0.022
non-TADF	MC2	41.45	0.5879	0.651	0.617
	OPM	21.76	0.6566	0.944	2.070
	*p*-Cz	43.66	0.5675	0.613	0.246
	ODFRCZ	43.69	0.5936	0.553	0.345
	ODBTCZ	52.73	0.4911	0.591	1.078
	C1	41.19	0.4369	0.283	0.202
	C2	95.14	0.4208	0.016	0.015

## 4 Conclusions

In this study, the photophysical and structural properties of 21 benzophenone-based TADF emitters and 7 benzophenone-based non-TADF emitters have been investigated by using computational descriptors with the aim to elucidate the factors causing RISC. The main descriptors have been identified as the torsion angle between the D and A, NTOs, Φ_S_ indices, Δ*E*_ST_ values, and SOC constants.

It was observed that the orthogonality of the molecular backbone is an important factor for achieving a successful RISC process. The compounds adopting a rigid molecular structure where free rotations are more restricted tend to have lower Δ*E*_ST_ values and Φ_S_ indices due to increased ICT through well separated electron and hole densities. Conversely, the emitters possessing less rigid and more freely rotating D-A arrangements were observed to have higher Δ*E*_ST_ values and Φ_S_ indices due to increased local excitation character induced by high amounts of overlap between the hole and electron and enhanced π-delocalization. Although molecular rigidity relies on both the molecular structures of the D and A, it was shown that the role of D units is more dominant, as freely rotating electron donors such as Cz, DPA, or thianthrene disrupt the orthogonality of the compounds, which leads to, in most cases, lower torsion angles (∼30 to 55°). The more rigid electron donors, such as DMAC, PXZ, or PTZ, enhance TADF efficiency through increased orthogonality and electron/hole separation. However, the A rigidity must also be taken into account. The compounds presenting more rigid and planar benzophenone derivatives, such as xanthone, as the A moiety, exhibit satisfying TADF characteristics, regardless of the electron donors. On the contrary, the emitters bearing the benzophenone A, in which the loose phenyl rings have more free rotation, exhibit less satisfying TADF properties. The presence of a π-bridge may also favorably affect the TADF properties owing to the increased spatial separation of D and A units as observed in compounds b1 and b4. Moreover, SOC values were calculated to be lower for the TADF compounds when the presence of highly twisted D-A frameworks induces lower Δ*E*_ST_ values due to enhanced electron/hole separation.

Excited state calculations were carried out using both S_0_ and T_1_ geometries because excited state processes, such as RISC, should take place from the T_1_ equilibrium geometry. In some cases, a significant geometrical relaxation was observed, in particular for the butterfly-shaped PXZ, PTZ, or thianthrene units, which become planar in T_1_ geometry. In these cases, different indicators were obtained for calculations performed at S_0_ or T_1_ geometries. However, in general, the outcome of the photophysical properties can be correctly inferred by solely considering the Franck–Condon geometry. The benchmark studies revealed that B3LYP and PBE0 can reproduce the excited state energies and UV–vis absorption spectra correctly for almost all emitters, while BLYP yielded satisfying results for several compounds only.

In summary, this study gives a comprehensive outlook on the relationship between molecular structure and photophysical features of a series of benzophenone-based TADF and non-TADF compounds, employing computational tools and several descriptors. It can be concluded that these descriptors, especially the topological Φ_S_ index, can be safely used to investigate TADF properties of different classes of compounds, as they predict the experimental findings quite accurately.

## Supporting Information Available

3D representations of the optimized geometries at M06-2X/6–31 + G(d,p) and B3LYP-D3/6–31 + G(d,p) levels, investigation of the conformational effects, benchmark calculations, UV–vis absorption spectra, investigated donor–acceptor torsion angles, hole and electron NTOs, Φ_S_ indices, low lying singlet–triplet energy gaps, spin-orbit coupling values, and S_0_, T_1_, and S_1_ cartesian coordinates of the most stable conformations optimized at the M06-2X/6–31 + G(d,p) level of theory (PDF)
