# Supplementary material for: Photophysical Properties of Benzophenone-Based TADF Emitters in Relation to Their Molecular Structure
Source: J Phys Chem A. 2022 Jan 21;126(4):473–84. doi: 10.1021/acs.jpca.1c08320 (PMC8895462; doi:10.1021/acs.jpca.1c08320)
Supplement: Supplementary file 1 — jp1c08320_si_001.pdf [file jp1c08320_si_001.pdf]

Supporting Information

Photophysical Properties of Benzophenone Based TADF Emitters in Relation to Their Molecular Structure

Ekin Esme Bas,[a] Pelin Ulukan,[a] Antonio Monari,[b,c] Viktorya Aviyente*,[a] and Saron Catak *,[a]

[a]Department of Chemistry, Bogazici University, Bebek, 34342 Istanbul, Turkey

[b]Université de Lorraine and CNRS, LPCT UMR 7019, F54000 Nancy, France

[c]Université de Paris and CNRS, ITODYS, F75006 Paris, France

Table of Contents

Table S1. Ground state geometry optimizations of different TADF and non-TADF compounds calculated with M06-2X/6-31+G(d,p) and B3LYP-D3/6-31+G(d,p). S4

Table S2. Torsion angles for the ground state geometry optimizations of different TADF and non-TADF compounds calculated with M06-2X/6-31+G(d,p) and B3LYP-D3/6-31+G(d,p). S5

Table S3. UV-Vis absorption spectra for the most stable conformation and weighted conformations of the emitters Px2BP, Cz2BP, DBT-BZ-PXZ and DBT-BZ-DMAC calculated at different levels of theory S6

Table S4. ΔE_{ST} values (eV) and relative Gibbs free energies (ΔG_{rel}, in kcal/mol) for all ground state conformations of Cz2BP and CC2BP, together with their Boltzmann weighted and experimental ΔE_{ST} values (eV) S7

Table S5. Absorption spectra for Px2BP, DMAC-BP and Cz2BP calculated at different levels of theory and experimental λ_{max} values in nm. S8

Table S6. Absorption spectra for CC2BP, A-BP-TA and OPDPO calculated at different levels of theory and experimental λ_{max} values in nm S9

Table S7. Absorption spectra for DBT-BZ-PXZ, DBT-BZ-PTZ and DBT-BZ-DMAC calculated at different levels of theory and experimental λ_{max} values in nm S10

Table S8. ΔE_{S1-T1} values (eV) calculated at different level of theories (BLYP, B3LYP, PBE0, M06-2X) for the S_0 geometries of Group 1 and Group 2 emitters together with the experimental values. S11

Table S9. ΔE_{S1-T1} values (eV) calculated at different level of theories (BLYP, B3LYP, PBE0, CAM-B3LYP, LC-ωPBE) for the S_0 and T_1 geometries of selected emitters chosen from each group together with the experimental values. S13

Table S10. ΔE_{S1-T1} values (eV) calculated at different level of theories (BLYP, B3LYP, PBE0) for the S_0, S_1 and T_1 geometries of selected emitters chosen from each group together with the experimental values. S14

Table S11. Oscillator strengths ($S_0 \rightarrow S_1$) for the S_0 and T_1 geometries of the investigated TADF and non-TADF calculated with B3LYP, PBE0 and BLYP functionals. (Basis set: 6-31+G(d,p)) S20

Table S12. Reorganization energies (kcal/mol) between the S_1 and S_0 geometries of selected compounds. S21

Table S13. 3D representations of the most stable ground state (S_0) and T_1 structures of Group 1 emitters along with the relative Gibbs free energies (kcal/mol) for the conformations, optimized at M06-2X/6-31+G(d,p) level of theory. S22

Table S14. 3D representations of the most stable ground state (S_0) and T_1 structures of Group 2 emitters along with the relative Gibbs free energies (kcal/mol) for the conformations, optimized at M06-2X/6-31+G(d,p) level of theory. S23

Table S15. 3D representations of the most stable ground state (S_0) and T_1 structures of Group 3 emitters along with the relative Gibbs free energies (kcal/mol) for the conformations, optimized at M06-2X/6-31+G(d,p) level of theory S24

Table S16. 3D representations of the most stable ground state (S_0) and T_1 structures of Group 4 emitters along with the relative Gibbs free energies (kcal/mol) for the conformations, optimized at M06-2X/6-31+G(d,p) level of theory S25

Table S17. 3D representations of the most stable ground state (S_0) and T_1 structures of non-TADF emitters along with the relative Gibbs free energies (kcal/mol) for the conformations, optimized at M06-2X/6-31+G(d,p) level of theory. S26

Table S18. Measured torsion angles of the S_0 and T_1 optimized geometries of the investigated compounds.....S30

Table S19. Hole and electron NTOs for Px2BP, DMAC-BP and Cz2BP (TDA: B3LYP/6-31+G(d,p))....S31

Table S20. Hole and electron NTOs for CC2BP, A-BP-TA and OPDPO (TDA: B3LYP/6-31+G(d,p)) S32

Table S21. Hole and electron NTOs for DBT-BZ-PXZ, DBT-BZ-PTZ and DBT-BZ-DMAC (TDA: B3LYP/6-31+G(d,p))....S33

Table S22. Hole and electron NTOs for CP-BP-PXZ and CP-BP-DMAC (TDA: B3LYP/6-31+G(d,p))....S34

Table S23. Hole and electron NTOs for a1, a2 and a3 (TDA: B3LYP/6-31+G(d,p)) S35

Table S24. Hole and electron NTOs for a4, b1 and b4 (TDA: B3LYP/6-31+G(d,p))....S36

Table S25. Hole and electron NTOs for ACRXTN, MCz-XT and 3-PXZ-XO (TDA: B3LYP/6-31+G(d,p)) ...S37

Table S26. Hole and electron NTOs for PTZ-XT, MC2 and OPM (TDA: B3LYP/6-31+G(d,p))....S38

Table S27. Hole and electron NTOs for *p*-Cz, ODFRCZ and ODBTCZ (TDA: B3LYP/6-31+G(d,p))....S39

Table S28. Hole and electron NTOs for C1 and C2 (TDA: B3LYP/6-31+G(d,p)) S40

Table S29. Φ_s indices for Group 1 emitters in Lowdin (L) and Mulliken (M) charge distributions for the excitations from S_0 to S_1 calculated with different functionals. S41

Table S30. Φ_s indices for Group 2 emitters in Lowdin (L) and Mulliken (M) charge distributions for the excitations from S_0 to S_1 calculated with different functionals. S42

Table S31. Φ_s indices for Group 3 emitters in Lowdin (L) and Mulliken (M) charge distributions for the excitations from S_0 to S_1 calculated with different functionals. S43

Table S32. Φ_s indices for Group 4 emitters in Lowdin (L) and Mulliken (M) charge distributions for the excitations from S_0 to S_1 calculated with different functionals. S44

Table S33. Φ_s indices for non-TADF emitters in Lowdin (L) and Mulliken (M) charge distributions for the excitations from S_0 to S_1 calculated with different functionals. S45

Table S34. Low lying singlet-triplet energy gaps (ΔE_{S1-T1}, ΔE_{S1-T2}) in eV for Group 1 emitters together with the experimental values S47

Table S35. Low lying singlet-triplet energy gaps (ΔE_{S1-T1}) in eV for Group 2 emitters together with the experimental values S48

Table S36. Low lying singlet-triplet energy gaps (ΔE_{S1-T1}, ΔE_{S1-T2}) in eV for Group 3 emitters together with the experimental values S49

Table S37. Low lying singlet-triplet energy gaps (ΔE_{S1-T1}) in eV for Group 4 emitters together with the experimental values S50

Table S38. Low lying singlet-triplet energy gaps (ΔE_{S1-T1}, ΔE_{S1-T2}) in eV for non-TADF emitters together with the experimental values S51

Table S39. Spin-orbit coupling (SOC) values between different energy states for the T_1 geometries of Group 1 emitters S53

Table S40. Spin-orbit coupling (SOC) values between S_1 and T_1 for the T_1 geometries of Group 2 emitters S54

Table S41. Spin-orbit coupling (SOC) values between different energy states for the T_1 geometries of Group 3 emitters S55

Table S42. Spin-orbit coupling (SOC) values between S_1 and T_1 for the T_1 geometries of Group 4 emitters S56

Table S43. Spin-orbit coupling (SOC) values between different energy states for the T_1 geometries of non-TADF emitters S57

List of Figures

Figure S1. Histogram chart including ΔE_{S1-T1} values calculated at different level of theories for the S_0 geometries of Group 1 emitters together with the experimental values (The basis set is 6-31+G(d,p)). S12

Figure S2. Histogram chart including ΔE_{S1-T1} values calculated at different level of theories for the S_0 geometries of three molecules from Group 2 emitters together with the experimental values (The basis set is 6-31+G(d,p) S12

Figure S3. The 3D representations for the S_0 and S_1 optimized geometries of the compounds given in Table S10. .... S14

Figure S4. Absorption spectra of Group 1 emitters calculated at different levels of theory........ S15

Figure S5. Absorption spectra of Group 2 emitters calculated at different levels of theory........ S16

Figure S6. Absorption spectra of Group 3 emitters calculated at different levels of theory........ S17

Figure S7. Absorption spectra of Group 4 emitters calculated at different levels of theory........ S18

Figure S8. Absorption spectra of non-TADF emitters calculated at different levels of theory. S19

Figure S9. Group 1 emitters together with their analyzed torsion angles (For A-BP-TA, θ_1 has been taken for the S_0 optimized geometry, θ_2 has been taken for the T_1 optimized geometry). S27

Figure S10. Group 2 emitters with together their analyzed torsion angles. S27

Figure S11. Group 3 emitters together with their analyzed torsion angles (For b1, θ_1 has been taken for the S_0 optimized geometry, θ_2 has been taken for the T_1 optimized geometry). S28

Figure S12. Group 4 emitters together with their analyzed torsion angles. S28

Figure S13. Non-TADF emitters together with their analyzed torsion angles S29

Figure S14. Φ_s indices for the TADF emitters calculated from S_0 and T_1 geometries with PBE0/6-31+G(d,p). S46

Figure S15. Φ_s indices for the TADF emitters calculated from S_0 and T_1 geometries with BLYP/6-31+G(d,p)S46

Figure S16. ΔE_{ST} values for TADF emitters calculated from S_0 and T_1 geometries with PBE0/6-31+G(d,p). (Experimental ΔE_{ST} values also included) S52

Figure S17. ΔE_{ST} values for TADF emitters calculated from S_0 and T_1 geometries with BLYP/6-31+G(d,p). (Experimental ΔE_{ST} values also included) S52

Cartesian Coordinates

S_0, T_1 and S_1 cartesian coordinates of the most stable conformations optimized at M06-2X/6-31+G(d,p) level of theory....................S58

Ground State Geometry Optimization with Different Methods

Table S1. Ground state geometry optimizations of different TADF and non-TADF compounds calculated with M06-2X/6-31+G(d,p) and B3LYP-D3/6-31+G(d,p).

	**Computational Method**	
**Compound**	**M06-2X/6-31+G(d,p)**	**B3LYP-D3/6-31+G(d,p)**
**CC2BP (Group 1)**	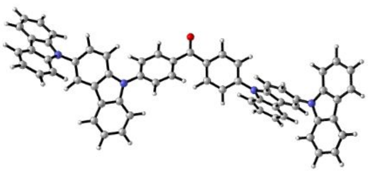	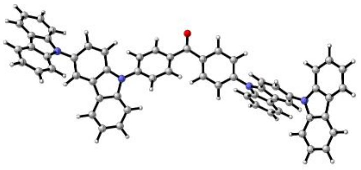
**CP-BP-DMAC (Group 2)**	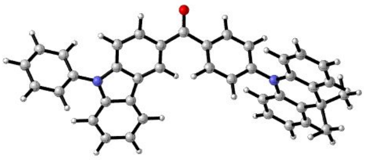	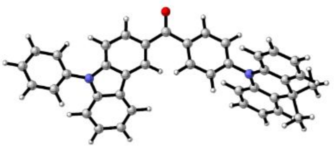
**b1 (Group 3)**	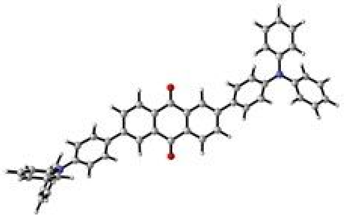	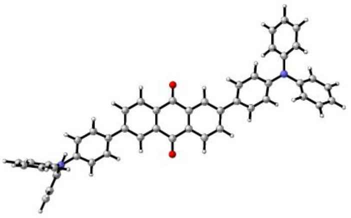
**ACRXTN (Group 4)**	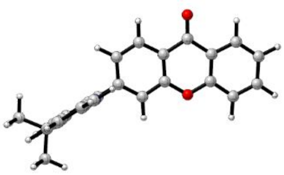	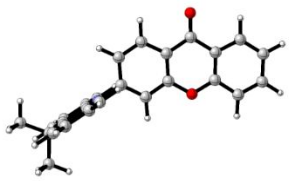
**C1 (Non-TADF)**	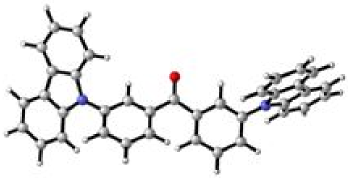	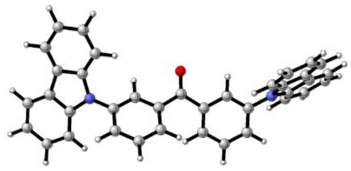

Table S2. Torsion angles for the ground state geometry optimizations of different TADF and non-TADF compounds calculated with M06-2X/6-31+G(d,p) and B3LYP-D3/6-31+G(d,p).

	Computational Method	
**Compound**	**M06-2X/6-31+G(d,p)**	**B3LYP-D3/6-31+G(d,p)**
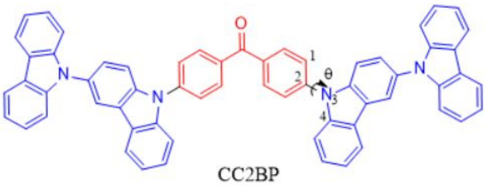 CC2BP	θ = 54.35	θ = 54.32
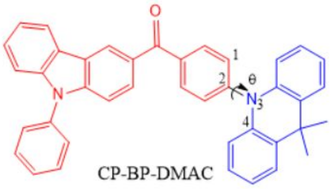 CP-BP-DMAC	θ = 89.82	θ = 89.60
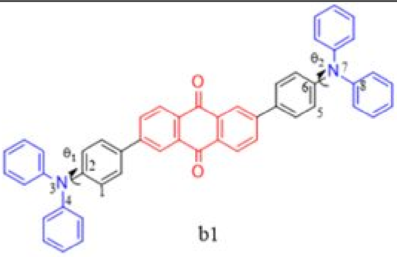 b1	θ = 36.16	θ = 36.27
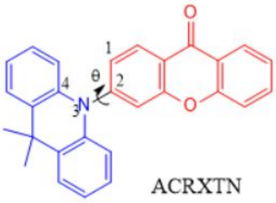 ACRXTN	θ_1 = 87.89	θ_1 = 88.93
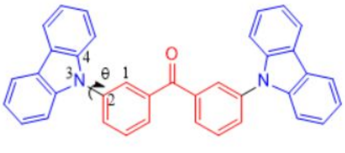 C1	θ = 53.63	θ = 55.13

Investigation of the Conformational Effects

Table S3. UV-Vis absorption spectra for the most stable conformation and weighted conformations of the emitters Px2BP, Cz2BP, DBT-BZ-PXZ and DBT-BZ-DMAC calculated at different levels of theory.

Compound	The Most Stable Conformation	Weighted Conformations
Px2BP in toluene	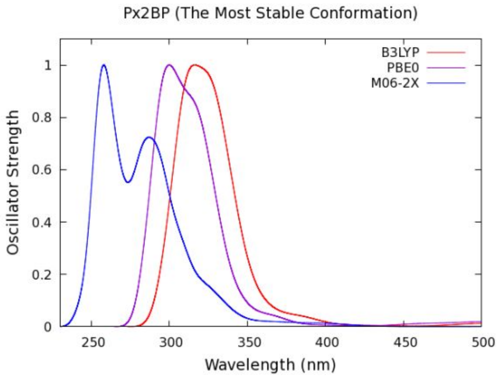	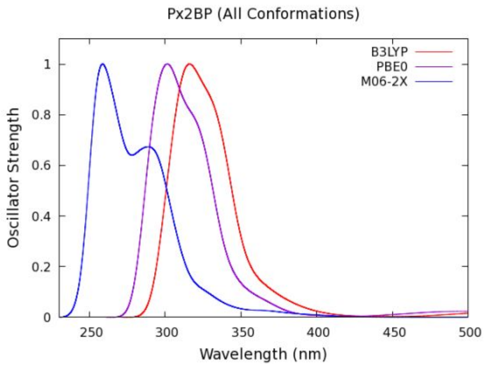
Cz2BP in toluene	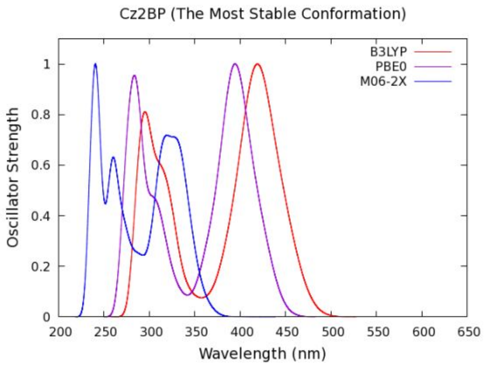	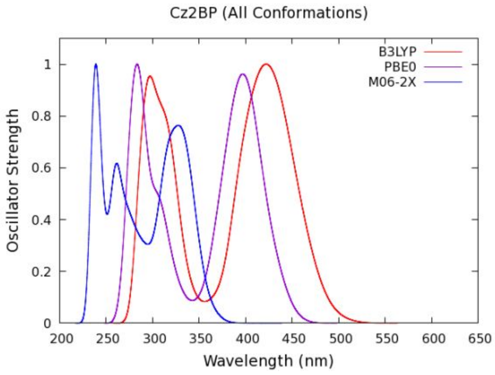
DBT-BZ-PXZ in THF	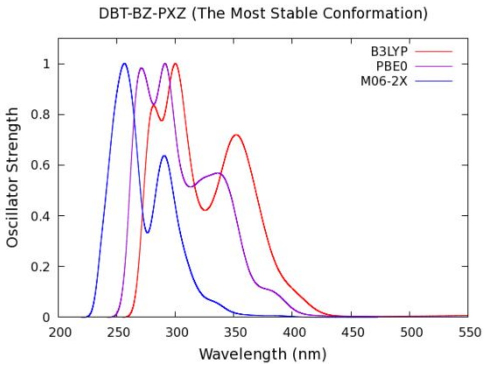	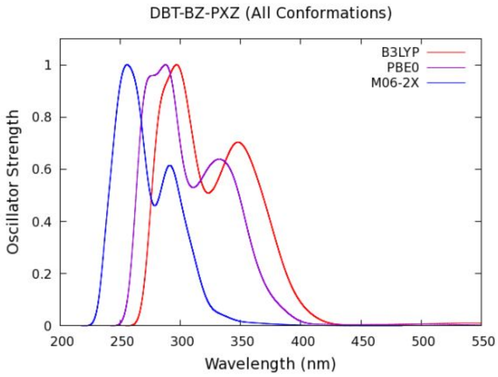
DBT-BZ-DMAC in THF	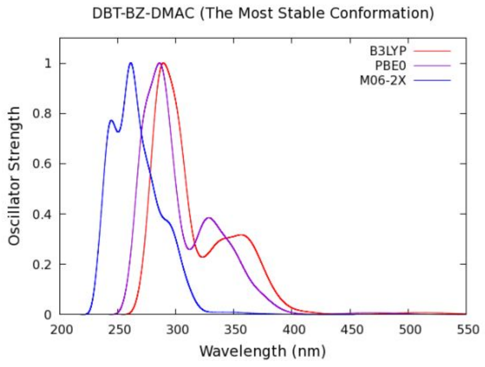	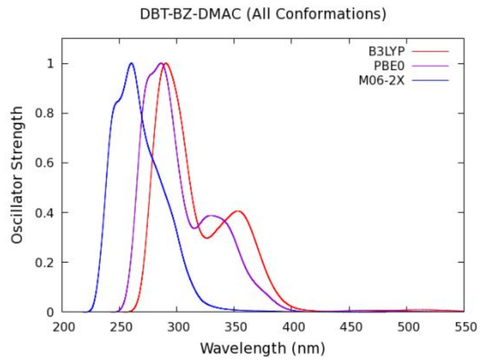

Table S4. ΔE_{ST} values (eV) and relative Gibbs free energies (ΔG_{rel}, in kcal/mol) for all ground state conformations of Cz2BP and CC2BP, together with their Boltzmann weighted and experimental ΔE_{ST} values (eV).

Compound	Conformation	ΔG_{rel}	ΔE_{ST}	Boltzmann Weighted ΔE_{ST}	Exp. ΔE_{ST}
Cz2BP	1	0.00	0.23	0.21	0.21
	2	0.12	0.21		
	3	0.13	0.21		
	4	0.28	0.18		
	5	0.30	0.18		
	6	0.51	0.23		
CC2BP	1	0.00	0.08	0.08	0.14
	2	0.10	0.07		
	3	0.17	0.07		
	4	0.31	0.09		
	5	0.40	0.08		
	6	0.56	0.08		

Benchmark Calculations

Table S5. Absorption spectra for Px2BP, DMAC-BP and Cz2BP calculated at different levels of theory and experimental λ_{max} values in nm.

Compound	Absorption Spectra	Exp. λ_{max}
Px2BP in toluene	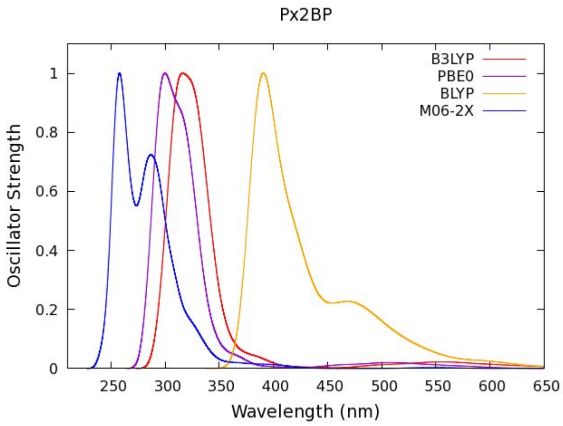	234, 413
DMAC-BP in DCM	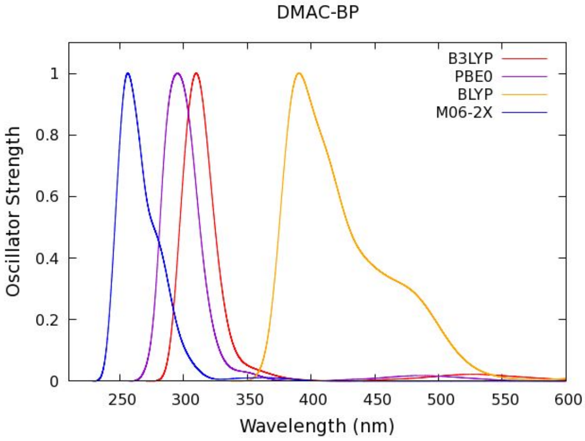	370
Cz2BP in toluene	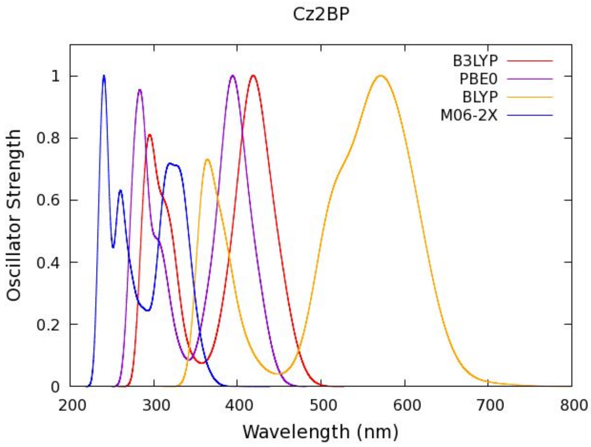	340, 353

Table S6. Absorption spectra for CC2BP, A-BP-TA and OPDPO calculated at different levels of theory and experimental λ_{max} values in nm.

Compound	Absorption Spectra	Exp. λ_{max}
CC2BP in toluene	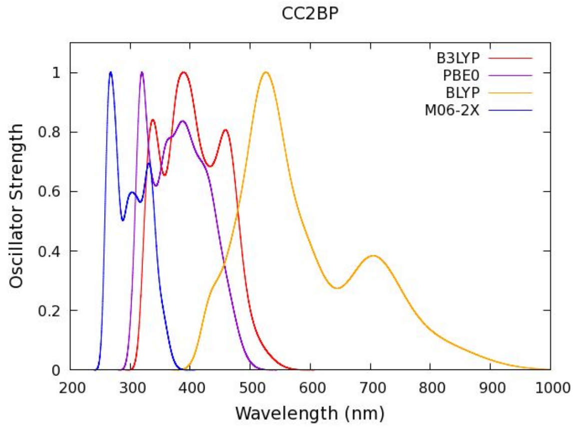	345
A-BP-TA in THF	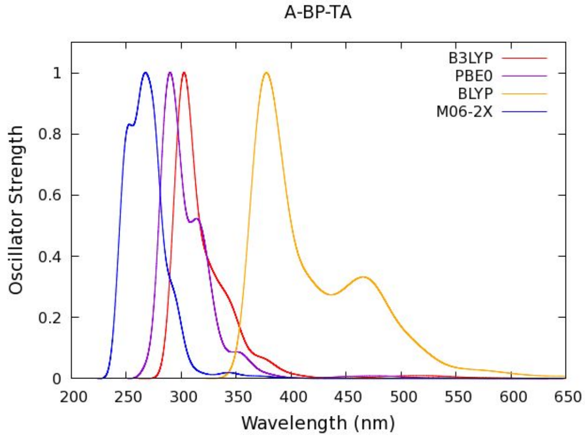	265, 285
OPDPO in THF	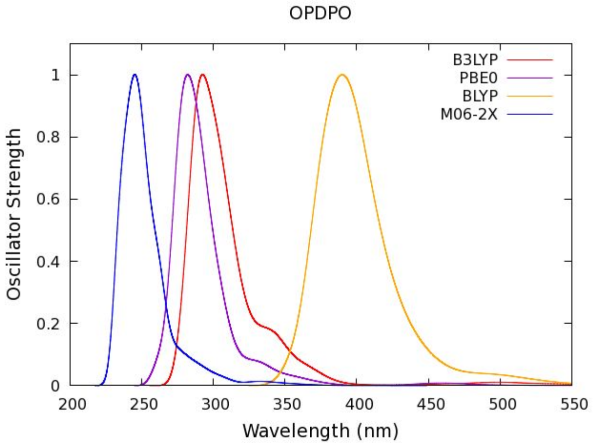	348

Table S7. Absorption spectra for DBT-BZ-PXZ, DBT-BZ-PTZ and DBT-BZ-DMAC calculated at different levels of theory and experimental λ_{max} values in nm.

Compound	Method	ΔE_{S1-T1}	Exp. ΔE_{ST}

Compound	Absorption Spectra	Exp. λ_{max}
DBT-BZ-PXZ in THF	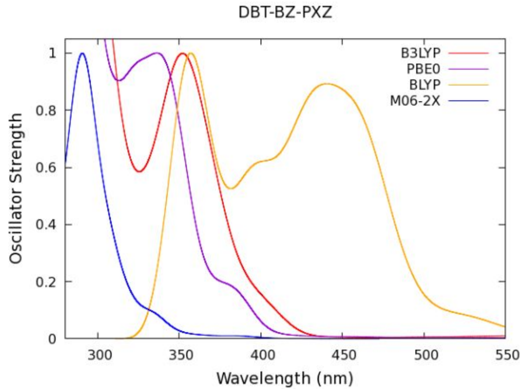	394, 312
DBT-BZ-PTZ in THF	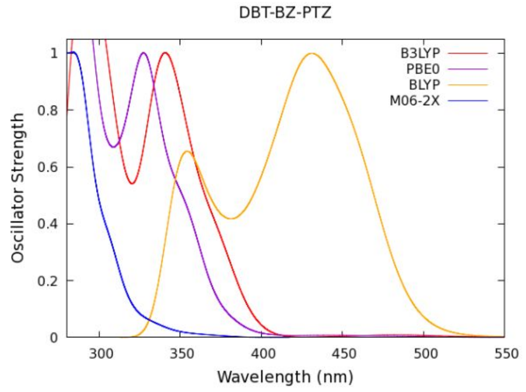	330
DBT-BZ-DMAC in THF	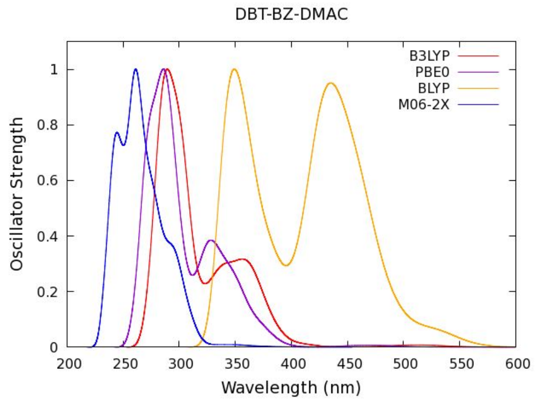	300

Table S8. $\Delta E_{S1\text{-}T1}$ values (eV) calculated at different level of theories (BLYP, B3LYP, PBE0, M06-2X) for the S_0 geometries of Group 1 and Group 2 emitters together with the experimental values.

Px2BP	BLYP	0.004	0.03
	B3LYP	0.007	
	PBE0	0.010	
	M06-2X	0.162	
DMAC-BP	BLYP	0.004	0.07
	B3LYP	0.006	
	PBE0	0.009	
	M06-2X	0.178	
Cz2BP	BLYP	0.099	0.21
	B3LYP	0.232	
	PBE0	0.321	
	M06-2X	0.496	
CC2BP	BLYP	0.017	0.14
	B3LYP	0.085	
	PBE0	0.165	
	M06-2X	0.484	
A-BP-TA	BLYP	0.004	0.06
	B3LYP	0.006	
	PBE0	0.008	
	M06-2X	0.215	
OPDPO	BLYP	0.003	0.02
	B3LYP	0.006	
	PBE0	0.010	
	M06-2X	0.361	
DBT-BZ-PXZ	BLYP	0.004	0.09
	B3LYP	0.008	
	PBE0	0.011	
	M06-2X	0.241	
DBT-BZ-PTZ	BLYP	0.004	0.05
	B3LYP	0.009	
	PBE0	0.015	
	M06-2X	0.402	
DBT-BZ-DMAC	BLYP	0.004	0.08
	B3LYP	0.006	
	PBE0	0.009	
	M06-2X	0.235	

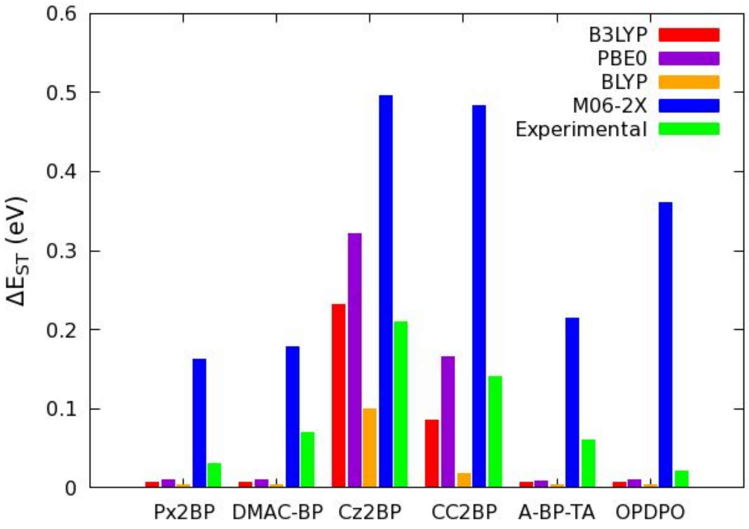

Figure S1. Histogram chart including $\Delta E_{S1\text{-}T1}$ values calculated at different level of theories for the S_0 geometries of Group 1 emitters together with the experimental values (The basis set is 6-31+G(d,p)).

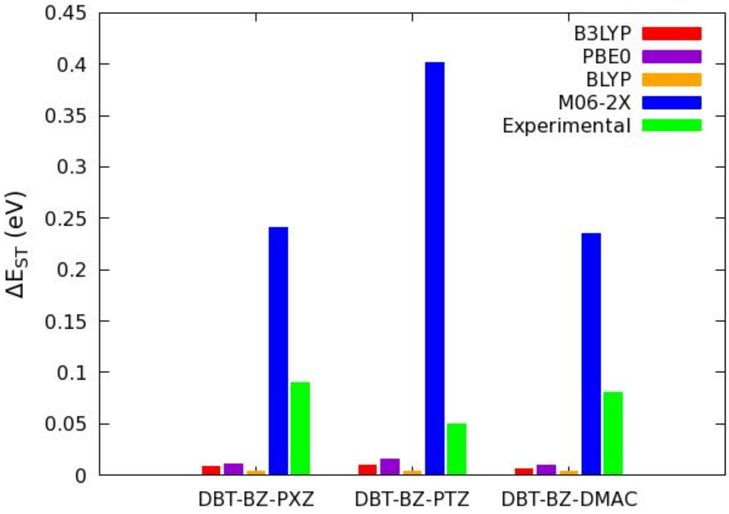

Figure S2. Histogram chart including $\Delta E_{S1\text{-}T1}$ values calculated at different level of theories for the S_0 geometries of three molecules from Group 2 emitters together with the experimental values (The basis set is 6-31+G(d,p).

Table S9. $\Delta E_{S1\text{-}T1}$ values (eV) calculated at different level of theories (BLYP, B3LYP, PBE0, CAM-B3LYP, LC-ωPBE) for the S_0 and T_1 geometries of selected emitters chosen from each group together with the experimental values.

Compound	Method	ΔE_{S1-T1} (S_0 Geometry)	ΔE_{S1-T1} (T_1 Geometry)	Exp. ΔE_{ST}
CC2BP	BLYP	0.017	0.015	0.14
	B3LYP	0.085	0.284	
	PBE0	0.165	0.406	
	CAM-B3LYP	0.660	0.889	
	LC-ωPBE	0.692	1.079	
CP-BP-DMAC	BLYP	0.004	0.006	0.016
	B3LYP	0.007	0.008	
	PBE0	0.010	0.011	
	CAM-B3LYP	0.456	0.121	
	LC-ωPBE	0.740	0.646	
b1	BLYP	0.085	0.088	0.24
	B3LYP	0.104	0.110	
	PBE0	0.124	0.097	
	CAM-B3LYP	0.506	0.641	
	LC-ωPBE	0.595	0.989	
ACRXTN	BLYP	0.006	0.006	0.06
	B3LYP	0.010	0.008	
	PBE0	0.013	0.011	
	CAM-B3LYP	0.083	0.017	
	LC-ωPBE	0.647	0.606	
C1	BLYP	0.060	0.101	-
	B3LYP	0.123	0.283	
	PBE0	0.161	0.343	
	CAM-B3LYP	0.562	0.651	
	LC-ωPBE	0.650	0.780	

Compound	Method	ΔE_{S1-T1} (S_0 Geometry)	ΔE_{S1-T1} (S_1 Geometry)	ΔE_{S1-T1} (T_1 Geometry)	Exp. ΔE_{ST}
Px2BP (Group 1)	BLYP	0.004	0.005	0.165	0.03
	B3LYP	0.007	0.007	0.104	
	PBE0	0.010	0.010	0.106	
DBT-BZ-PXZ (Group 2)	BLYP	0.004	0.009	0.026	0.09
	B3LYP	0.008	0.012	0.018	
	PBE0	0.011	0.014	0.020	
a1 (Group 3)	BLYP	0.254	0.005	0.309	0.29
	B3LYP	0.284	0.007	0.302	
	PBE0	0.317	0.010	0.324	
MCz-XT (Group 4)	BLYP	0.006	0.006	0.009	0.011
	B3LYP	0.009	0.009	0.011	
	PBE0	0.012	0.011	0.014	
p-Cz (Non-TADF)	BLYP	0.164	0.188	0.443	0.61
	B3LYP	0.275	0.325	0.613	
	PBE0	0.358	0.442	0.697	

Table S10. ΔE_{S1-T1} values (eV) calculated at different level of theories (BLYP, B3LYP, PBE0) for the S_0, S_1 and T_1 geometries of selected emitters chosen from each group together with the experimental values.

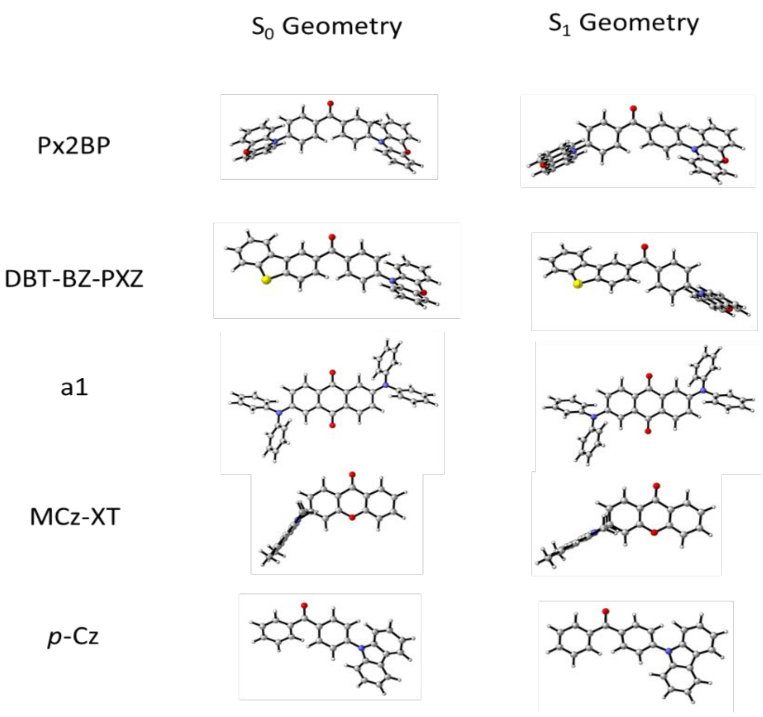

Figure S3. The 3D representations for the S_0 and S_1 optimized geometries of the compounds given in Table S10.

UV-Vis Absorption Spectra

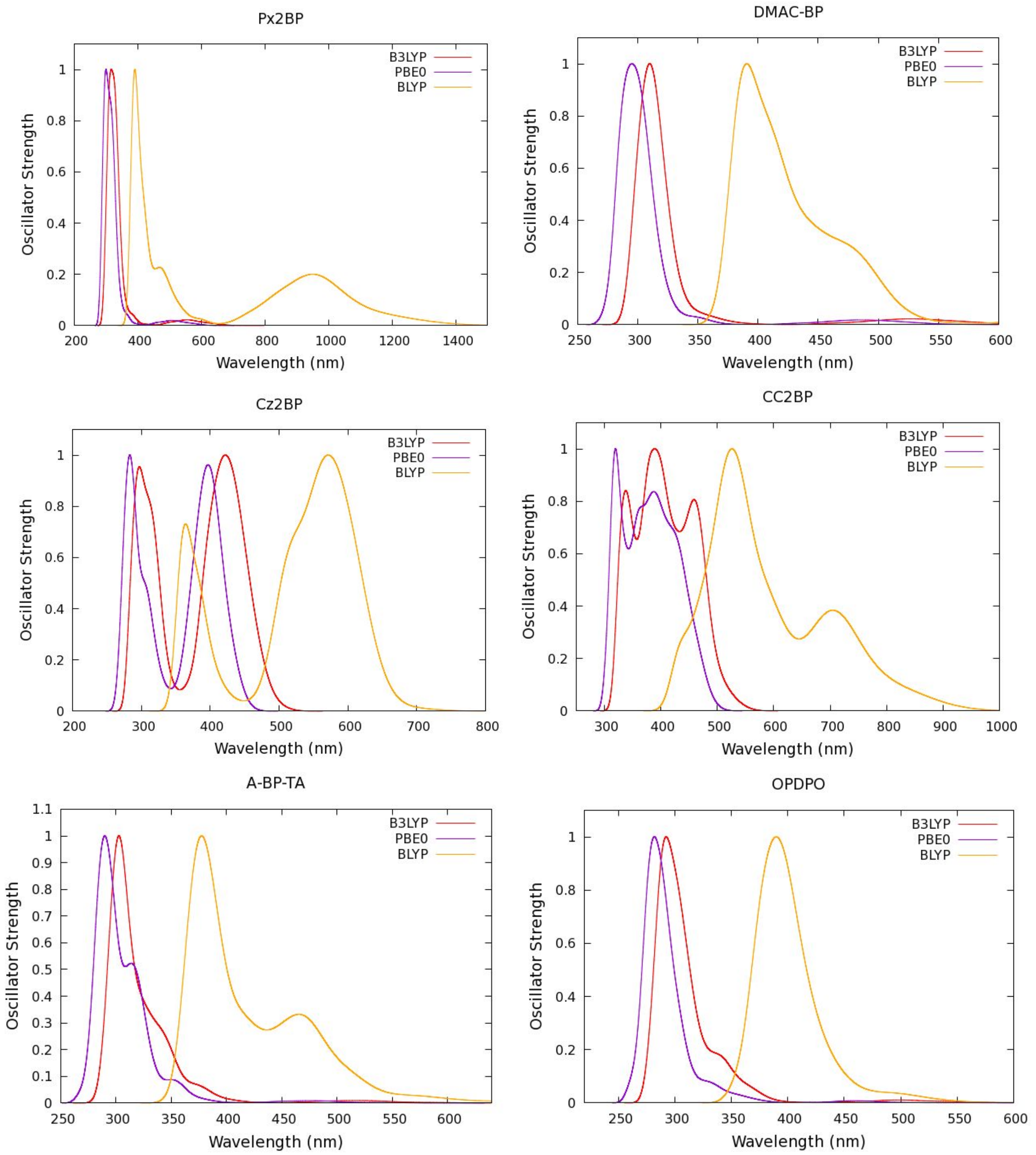

Figure S4. Absorption spectra of Group 1 emitters calculated at different levels of theory. Full-width half maximum values (nm) are given as follows, Px2BP (51 for B3LYP, 61 for PBE0, 46 for BLYP), DMAC-BP (39 for B3LYP, 50 for PBE0, 61 for BLYP), Cz2BP (165 for B3LYP, 140 for PBE0, 109 for BLYP), CC2BP (70 for B3LYP, 31 for PBE0, 120 for BLYP), A-BP-TA (35 for B3LYP, 62 for PBE0, 48 for BLYP), OPDPO (44 for B3LYP, 48 for PBE0, 61 for BLYP)

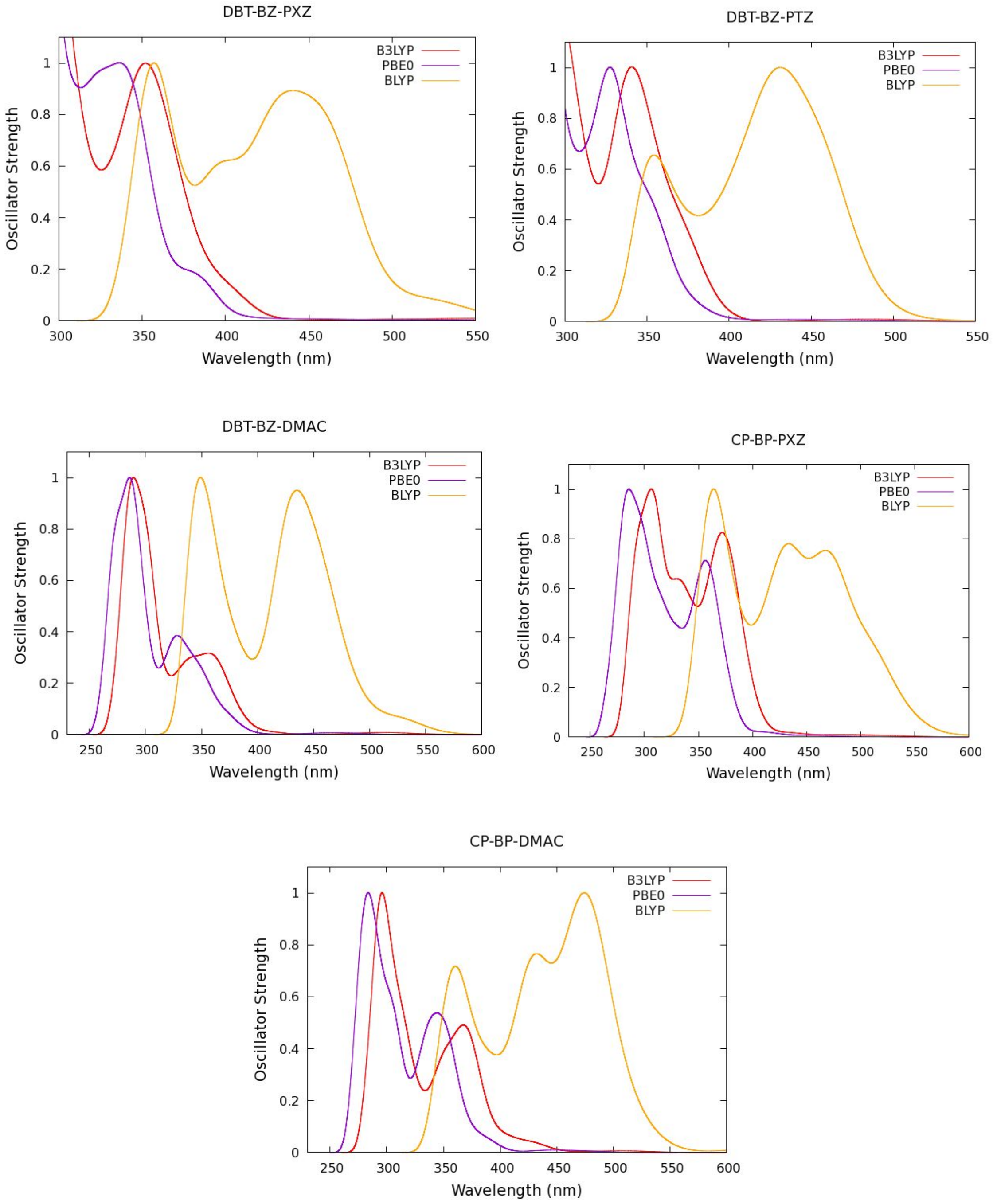

Figure S5. Absorption spectra of Group 2 emitters calculated at different levels of theory. Full-width half maximum values (nm) are given as follows, DBT-BZ-PXZ (39 for B3LYP, 45 for PBE0, 38 for BLYP), DBT-BZ-PTZ (36 for B3LYP, 33 for PBE0, 77 for BLYP), DBT-BZ-DMAC (45 for B3LYP, 46 for PBE0, 35 for BLYP), CP-BP-PXZ (47 for B3LYP, 63 for PBE0, 42 for BLYP), CP-BP-DMAC (34 for B3LYP, 41 for PBE0, 105 for BLYP)

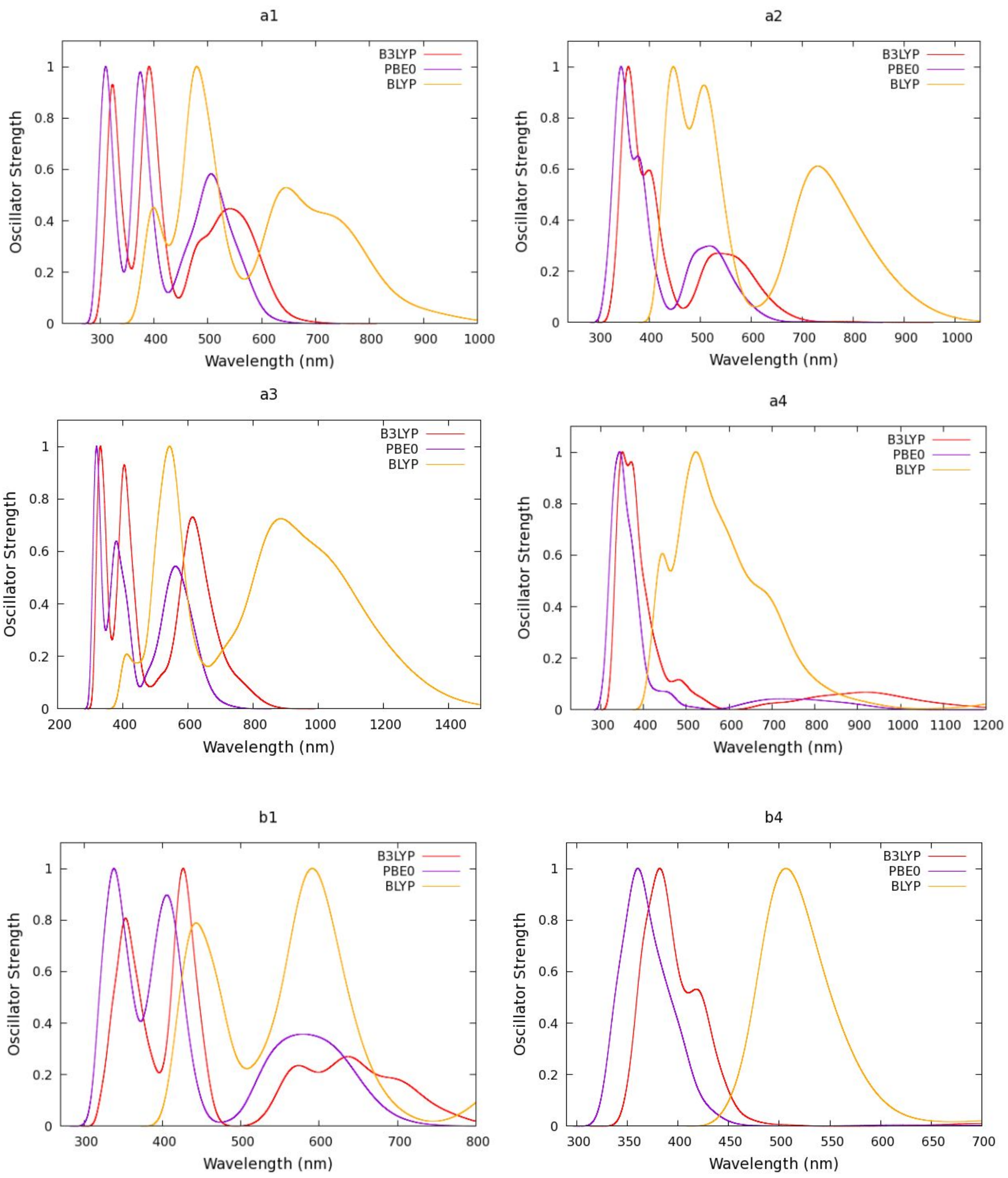

Figure S6. Absorption spectra of Group 3 emitters calculated at different level of theories. Full-width half maximum values (nm) are given as follows, a1 (29 for B3LYP, 43 for PBE0, 71 for BLYP), a2 (79 for B3LYP, 83 for PBE0, 114 for BLYP), a3 (40 for B3LYP, 37 for PBE0, 120 for BLYP), a4 (103 for B3LYP, 87 for PBE0, 192 for BLYP), b1 (37 for B3LYP, 51 for PBE0, 102 for BLYP), b4 (59 for B3LYP, 71 for PBE0, 83 for BLYP)

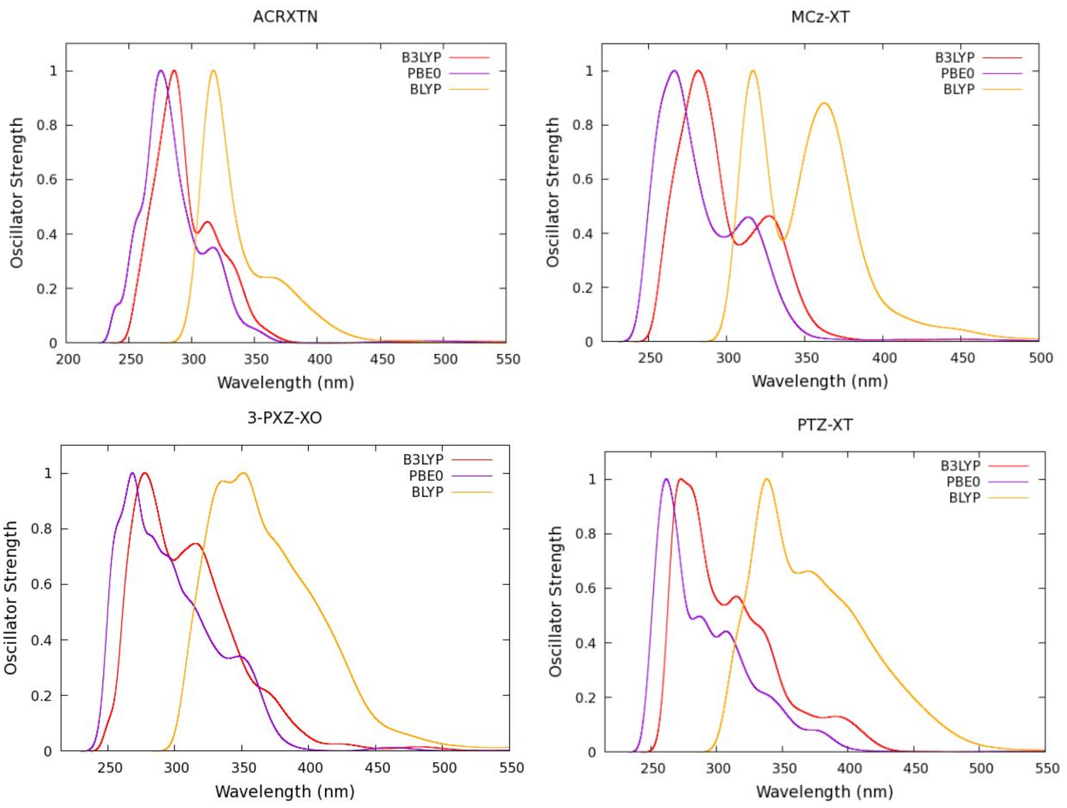

Figure S7. Absorption spectra of Group 4 emitters calculated at different level of theories. Full-width half maximum values (nm) are given as follows, ACRXTN (36 for B3LYP, 34 for PBE0, 29 for BLYP), MCz-XT (30 for B3LYP, 32 for PBE0, 21 for BLYP), 3-PXZ-XO (77 for B3LYP, 74 for PBE0, 101 for BLYP), PTZ-XT (70 for B3LYP, 36 for PBE0, 106 for BLYP)

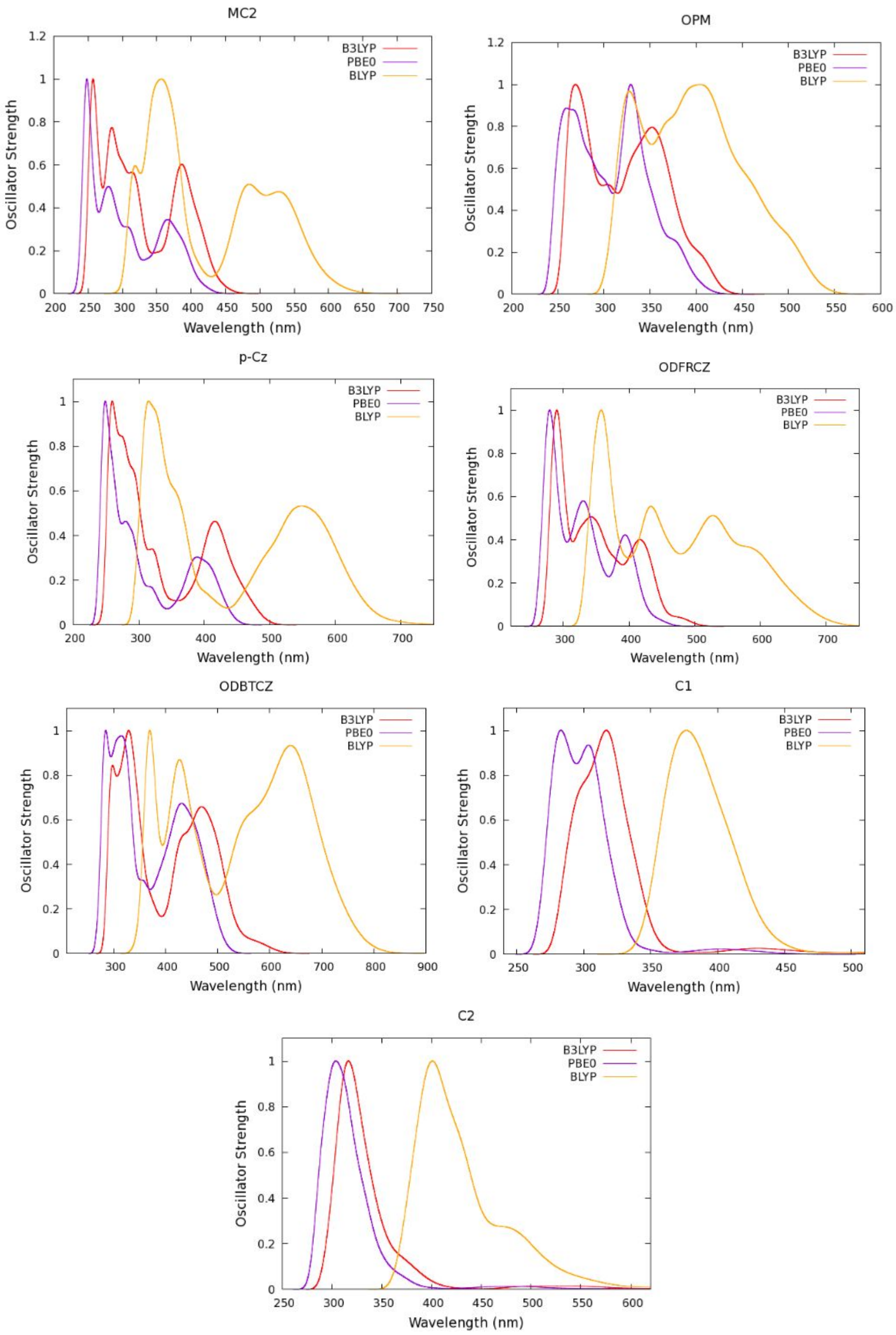

Figure S8. Absorption spectra of non-TADF emitters calculated at different level of theories. Full-width half maximum values (nm) are given as follows, MC2 (9 for B3LYP, 21 for PBE0, 80 for BLYP), OPM (49 for B3LYP, 39 for PBE0, 160 for BLYP), *p*-Cz (56 for B3LYP, 31 for PBE0, 70 for BLYP), ODFRCZ (33 for B3LYP, 47 for PBE0, 44 for BLYP), ODBTCZ (71 for B3LYP, 85 for PBE0, 32 for BLYP), C1 (52 for B3LYP, 51 for PBE0, 59 for BLYP), C2 (42 for B3LYP, 55 for PBE0, 70 for BLYP).

Table S11. Oscillator strengths ($S_0 \rightarrow S_1$) for the S_0 and T_1 geometries of the investigated TADF and non-TADF calculated with B3LYP, PBE0 and BLYP functionals. (Basis set: 6-31+G(d,p))

		S_0 Geometry			T_1 Geometry		
**Group**	**Compound**	**B3LYP**	**PBE0**	**BLYP**	**B3LYP**	**PBE0**	**BLYP**
Group 1	Px2BP	0.0001	0.0001	0.0001	0.0833	0.0836	0.0074
	DMAC-BP	0.0000	0.0000	0.0000	0.0055	0.0056	0.0063
	Cz2BP	0.4329	0.4810	0.3080	0.6715	0.7240	0.5327
	CC2BP	0.2302	0.3003	0.0921	0.3716	0.4880	0.0608
	A-BP-TA	0.0006	0.0006	0.0006	0.0766	0.0854	0.0005
	OPDPO	0.0000	0.0001	0.0000	0.0001	0.0001	0.0001
Group 2	DBT-BZ-PXZ	0.0004	0.0005	0.0004	0.0119	0.0121	0.0143
	DBT-BZ-PTZ	0.0001	0.0001	0.0001	0.0000	0.0000	0.0000
	DBT-BZ-DMAC	0.0002	0.0002	0.0003	0.0002	0.0002	0.0002
	CP-BP-PXZ	0.0003	0.0004	0.0003	0.0042	0.0044	0.0044
	CP-BP-DMAC	0.0002	0.0002	0.0002	0.0001	0.0001	0.0002
Group 3	a1	0.0000	0.0000	0.0000	0.0000	0.0000	0.0000
	a2	0.0000	0.0000	0.0000	0.0000	0.0000	0.0000
	a3	0.0000	0.0000	0.2612	0.0000	0.0000	0.3484
	a4	0.0000	0.0000	0.0000	0.0000	0.0000	0.0000
	b1	0.2969	0.2488	0.3055	0.3514	0.4725	0.2488
	b4	0.0000	0.0000	0.0004	0.0012	0.0012	0.0028
Group 4	ACRXTN	0.0001	0.0001	0.0001	0.0001	0.0000	0.0001
	MCz-XT	0.0001	0.0001	0.0001	0.0038	0.0040	0.0035
	PXZ-XO	0.0001	0.0001	0.0000	0.0554	0.0567	0.0609
	PTZ-XT	0.0000	0.0000	0.0000	0.0004	0.0004	0.0004
Non-TADF	MC2	0.2864	0.3172	0.1991	0.7082	0.7434	0.5808
	OPM	0.4243	0.2618	0.1734	0.7383	0.4178	0.3451
	p-Cz	0.2581	0.2864	0.1810	0.6160	0.6530	0.4823
	ODFRCZ	0.2926	0.3295	0.1879	0.7291	0.7715	0.5642
	ODBTCZ	0.2895	0.3270	0.1904	0.3106	0.4829	0.1163
	C1	0.0042	0.0041	0.0048	0.0142	0.0167	0.0021
	C2	0.0010	0.0008	0.0013	0.0005	0.0005	0.0007

Table S12. Reorganization energies (kcal/mol) between the S_1 and S_0 geometries of selected compounds.

Group	Compound	Reorganization Energy
Group 1	Px2BP	12.63
	Cz2BP	6.30
	A-BP-TA	12.95
	OPDPO	15.46
Group 2	DBT-BZ-PXZ	10.84
	DBT-BZ-PTZ	14.93
	DBT-BZ-DMAC	10.42
	CP-BP-PXZ	13.52
	CP-BP-DMAC	12.94
Group 3	a1	7.77
	a4	8.45
Group 4	ACRXTN	6.21
	MCz-XT	6.99
	PXZ-XO	8.66
	PTZ-XT	12.27
Non-TADF	MC2	12.81
	OPM	17.82
	p-Cz	6.39
	ODFRCZ	7.54
	ODBTCZ	6.71
	C1	6.13

3D Representations of the Optimized Ground State Geometries

Table S13. 3D representations of the most stable ground state (S_0) and T_1 structures of Group 1 emitters along with the relative Gibbs free energies (kcal/mol) for the conformations, optimized at M06-2X/6-31+G(d,p) level of theory.

Emitter	Conf.	Rel. Gibbs Free Energy	Optimized Geometry at S_0 Level	Optimized Geometry at T_1 Level
Px2BP in toluene	1<br>2<br>3<br>4<br>5<br>6	0.00<br>0.00<br>0.24<br>0.25<br>0.51<br>0.51	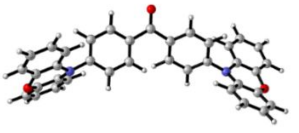	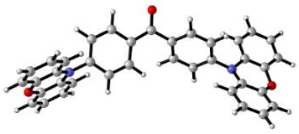
DMAC-BP in DCM	1<br>2	0.00<br>0.24	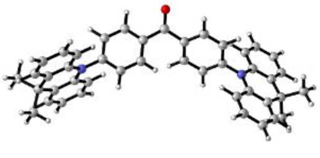	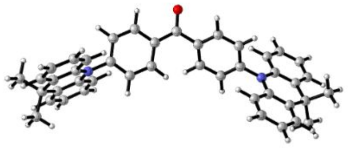
Cz2BP in toluene	1<br>2<br>3<br>4<br>5<br>6	0.00<br>0.01<br>0.12<br>0.13<br>0.28<br>0.30	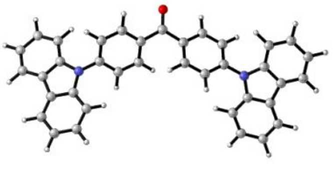	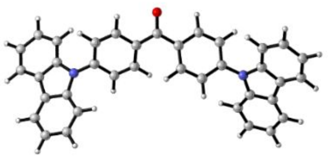
CC2BP in toluene	1<br>2<br>3<br>4<br>5<br>6	0.00<br>0.10<br>0.17<br>0.31<br>0.40<br>0.56	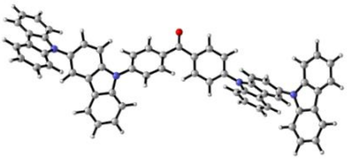	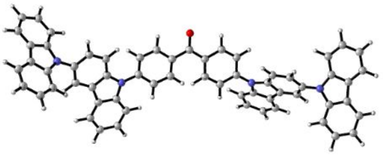
A-BP-TA in THF	1<br>2<br>3<br>4<br>5<br>6<br>7<br>8	0.00<br>0.47<br>0.47<br>0.58<br>0.62<br>0.72<br>0.79<br>0.80	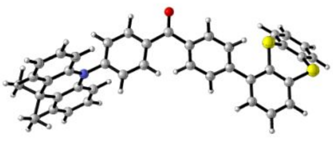	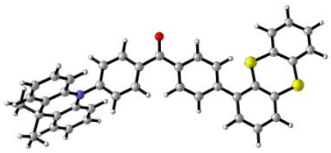
OPDPO in THF	1<br>2<br>3<br>4<br>5<br>6<br>7<br>8	0.00<br>0.14<br>0.26<br>0.47<br>0.53<br>0.92<br>0.95<br>2.84	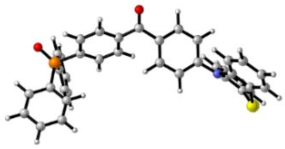	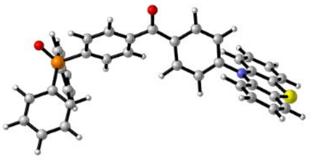

Table S14. 3D representations of the most stable ground state (S_0) and T_1 structures of Group 2 emitters along with the relative Gibbs free energies (kcal/mol) for the conformations, optimized at M06-2X/6-31+G(d,p) level of theory.

Emitter	Conf.	Rel. Gibbs Free Energy	Optimized Geometry at S_0 Level	Optimized Geometry at T_1 Level
DBT-BZ-PXZ in THF	1<br>2<br>3<br>4	0.00<br>0.07<br>0.23<br>0.37	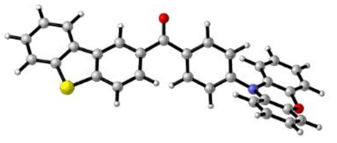	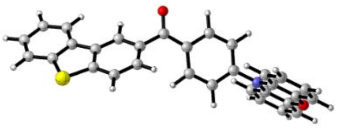
DBT-BZ-PTZ in THF	1<br>2<br>3<br>4	0.00<br>0.15<br>0.25<br>0.57	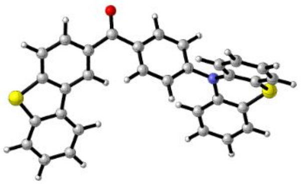	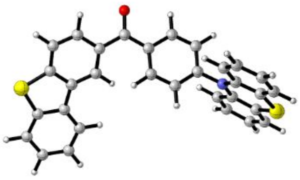
DBT-BZ-DMAC in THF	1<br>2	0.00<br>0.17	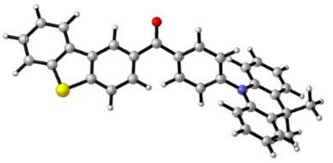	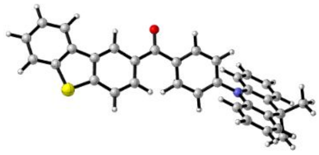
CP-BP-PXZ in THF	1<br>2<br>3<br>4<br>5<br>6<br>7<br>8	0.00<br>0.10<br>0.10<br>0.15<br>0.18<br>0.23<br>0.35<br>0.44	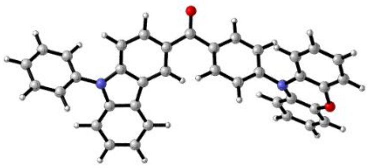	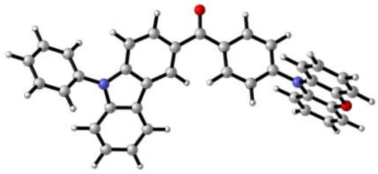
CP-BP-DMAC in THF	1<br>2<br>3<br>4	0.00<br>0.05<br>0.42<br>0.44	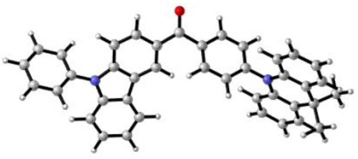	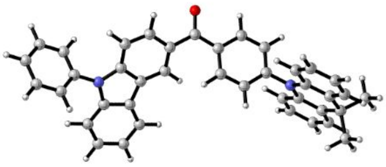

Table S15. 3D representations of the most stable ground state (S_0) and T_1 structures of Group 3 emitters along with the relative Gibbs free energies (kcal/mol) for the conformations, optimized at M06-2X/6-31+G(d,p) level of theory.

Emitter	Conf.	Rel. Gibbs Free Energy	Optimized Geometry at S_0 Level	Optimized Geometry at T_1 Level

a1 in toluene	1	0.00	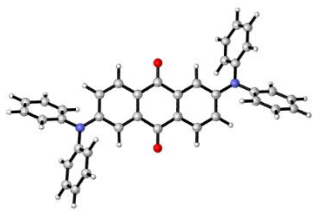	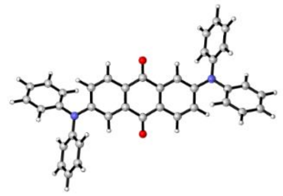
a2 in toluene	1	0.00	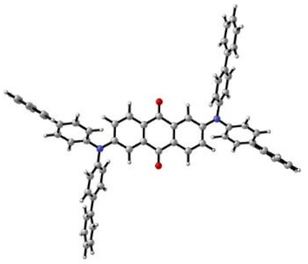	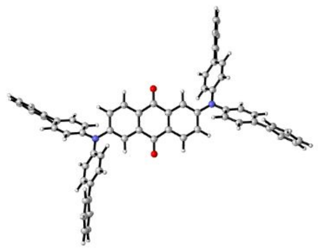
a3 in toluen	1<br>2<br>3	0.00<br>0.04<br>0.05	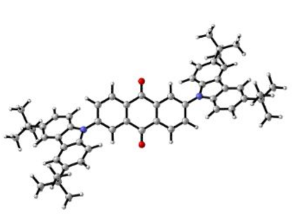	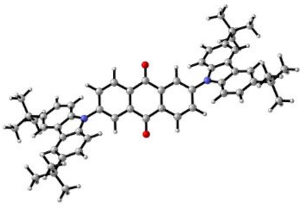
a4 in toluene	1	0.00	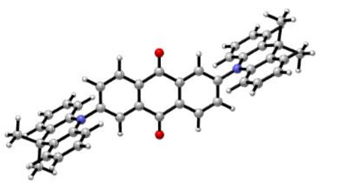	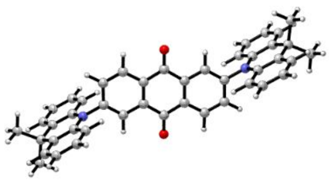
b1 in toluene	1<br>2<br>3<br>4<br>5<br>6	0.00<br>0.02<br>0.37<br>0.47<br>0.69<br>0.87	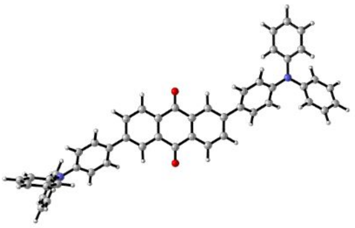	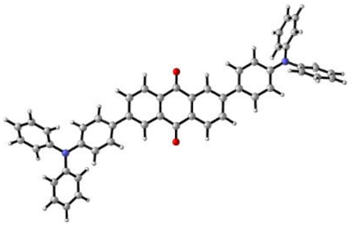
b4 in toluene	1<br>2<br>3	0.00<br>0.45<br>0.49	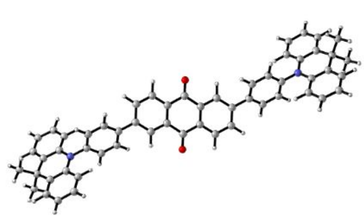	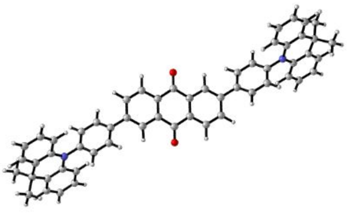

Table S16. 3D representations of the most stable ground state (S_0) and T_1 structures of Group 4 emitters along with the relative Gibbs free energies (kcal/mol) for the conformations, optimized at M06-2X/6-31+G(d,p) level of theory.

Emitter	Conf.	Rel. Gibbs Free Energy	Optimized Geometry at S_0 Level	Optimized Geometry at T_1 Level
ACRXTN in DCM	1	0.00	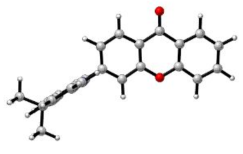	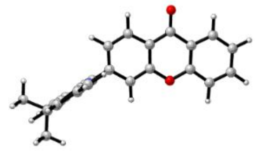
MCz-XT in toluene	1<br>2	0.00<br>0.63	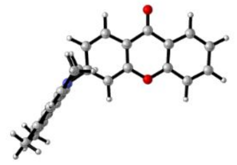	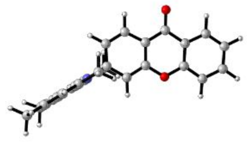
3-PXZ-XO in toluene	1<br>2	0.00<br>0.10	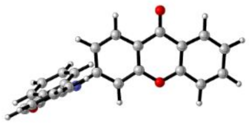	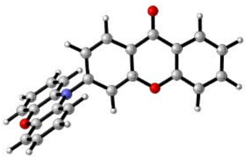
PTZ-XT in THF	1<br>2<br>3<br>4	0.00<br>0.00<br>1.67<br>1.71	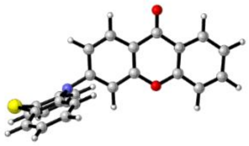	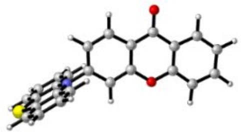

Table S17. 3D representations of the most stable ground state (S_0) and T_1 structures of non-TADF emitters along with the relative Gibbs free energies (kcal/mol) for the conformations, optimized at M06-2X/6-31+G(d,p) level of theory.

Emitter	Conf.	Rel. Gibbs Free Energy	Optimized Geometry at S_0 Level	Optimized Geometry at T_1 Level
MC2 in toluene	1<br>2<br>3<br>4	0.00<br>0.03<br>0.03<br>0.16	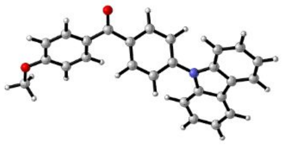	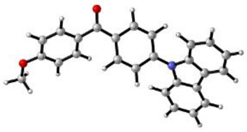
OPM in toluene	1<br>2<br>3<br>4	0.00<br>0.03<br>0.13<br>0.27	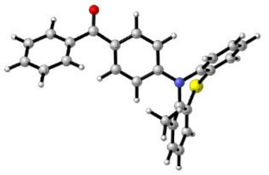	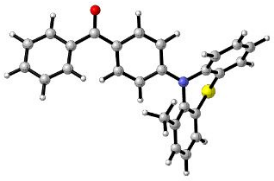
p-Cz in THF	1<br>2<br>3<br>4	0.00<br>0.17<br>0.00<br>0.17	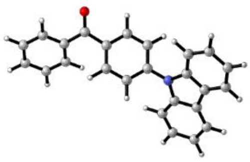	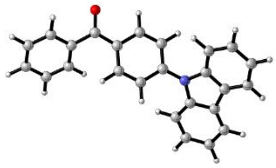
ODFRCZ in DCM	1<br>2<br>3<br>4	0.00<br>0.30<br>0.37<br>0.42	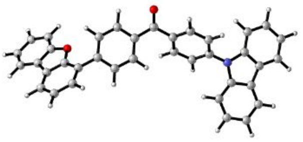	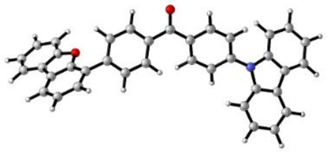
ODBTCZ in DCM	1<br>2<br>3<br>4	0.00<br>0.04<br>0.27<br>0.32	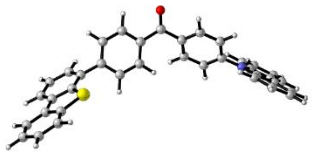	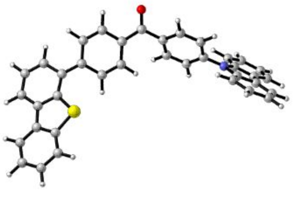
C1 in THF	1<br>2<br>3	0.00<br>0.03<br>0.50	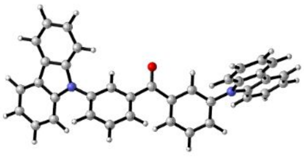	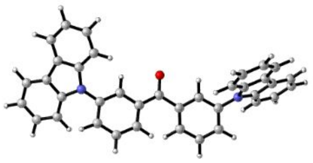
C2 in THF	1<br>2<br>3	0.00<br>0.21<br>0.58	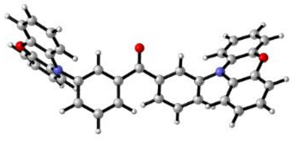	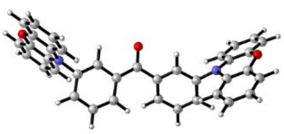

Investigated Donor-Acceptor Torsion Angles

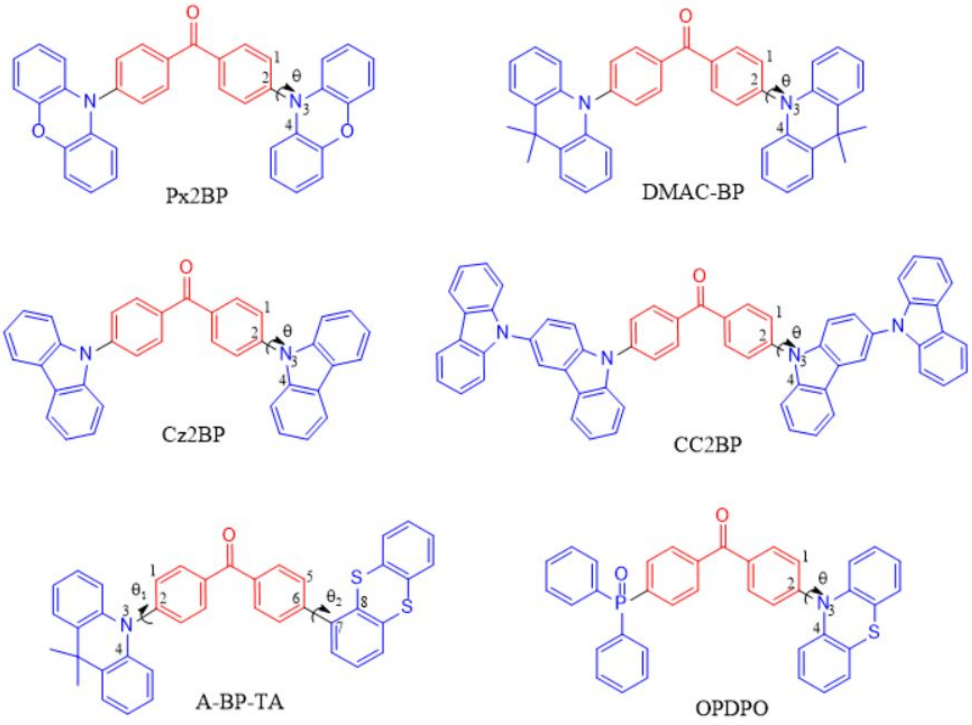

Figure S9. Group 1 emitters together with their analyzed torsion angles (For A-BP-TA, θ_1 has been taken for the S_0 optimized geometry, θ_2 has been taken for the T_1 optimized geometry).

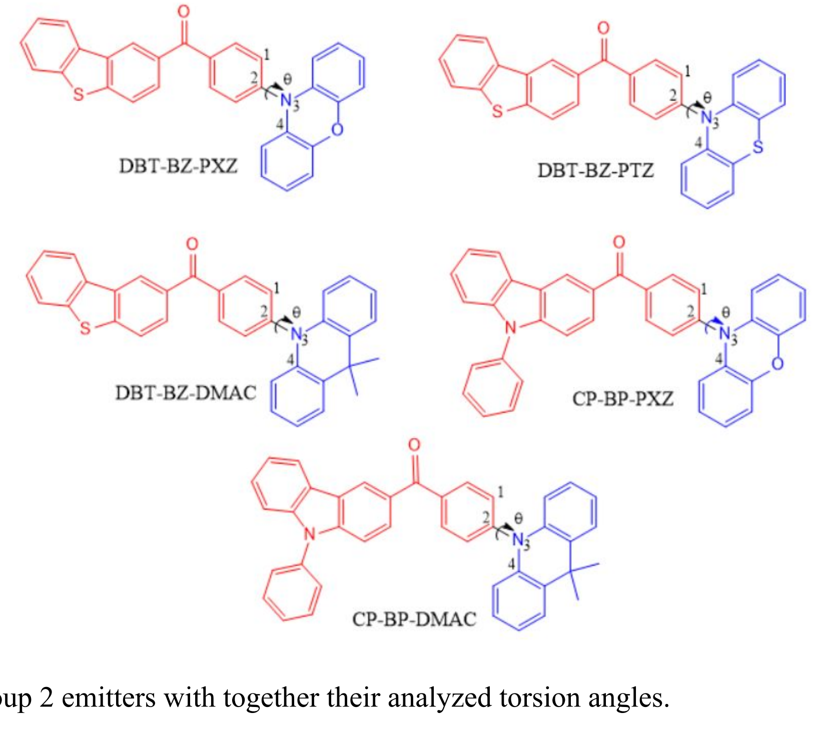

Figure S10. Group 2 emitters with together their analyzed torsion angles.

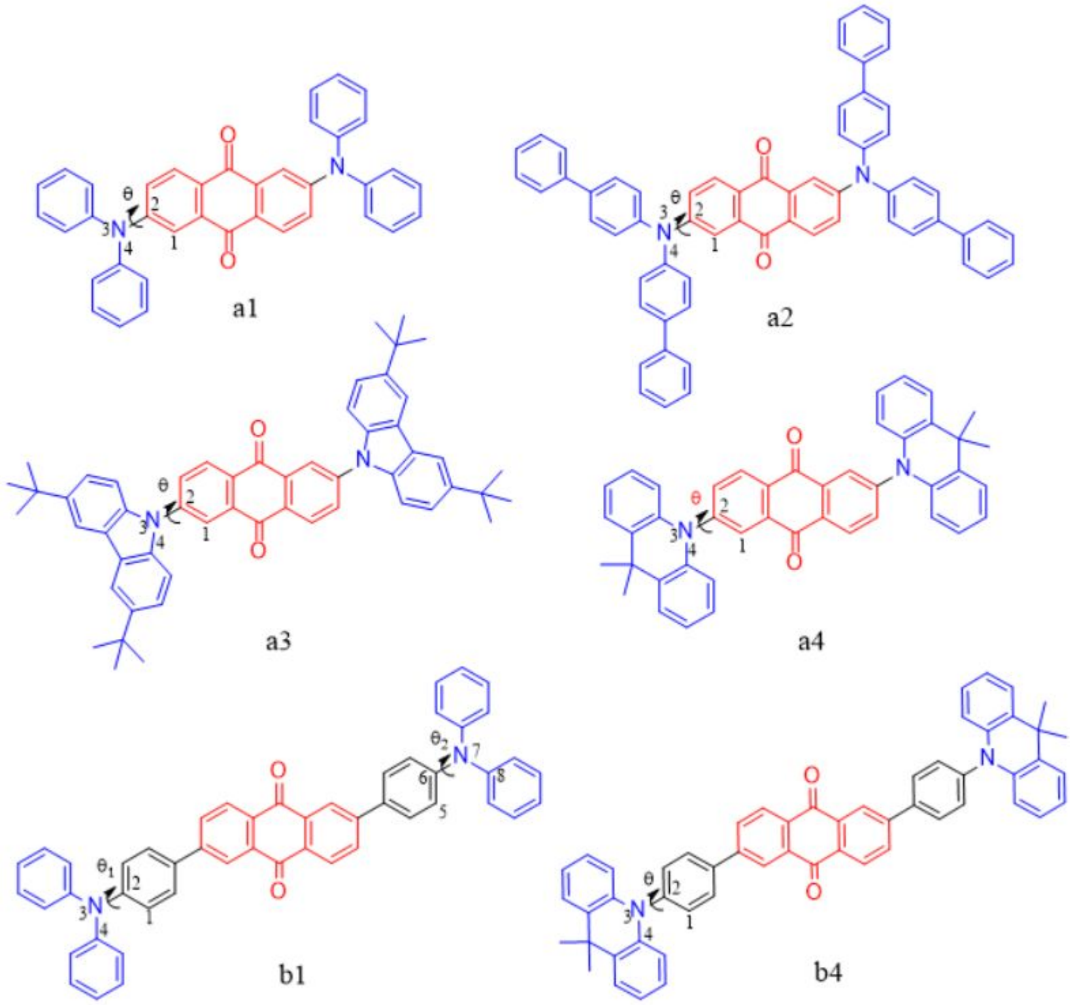

Figure S11. Group 3 emitters together with their analyzed torsion angles (For b1, θ_1 has been taken for the S_0 optimized geometry, θ_2 has been taken for the T_1 optimized geometry).

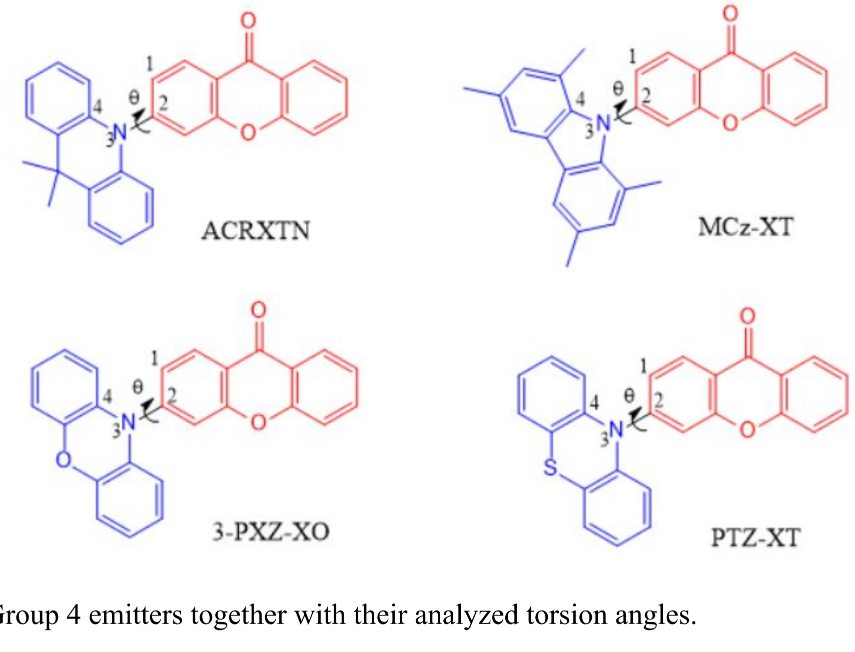

Figure S12. Group 4 emitters together with their analyzed torsion angles.

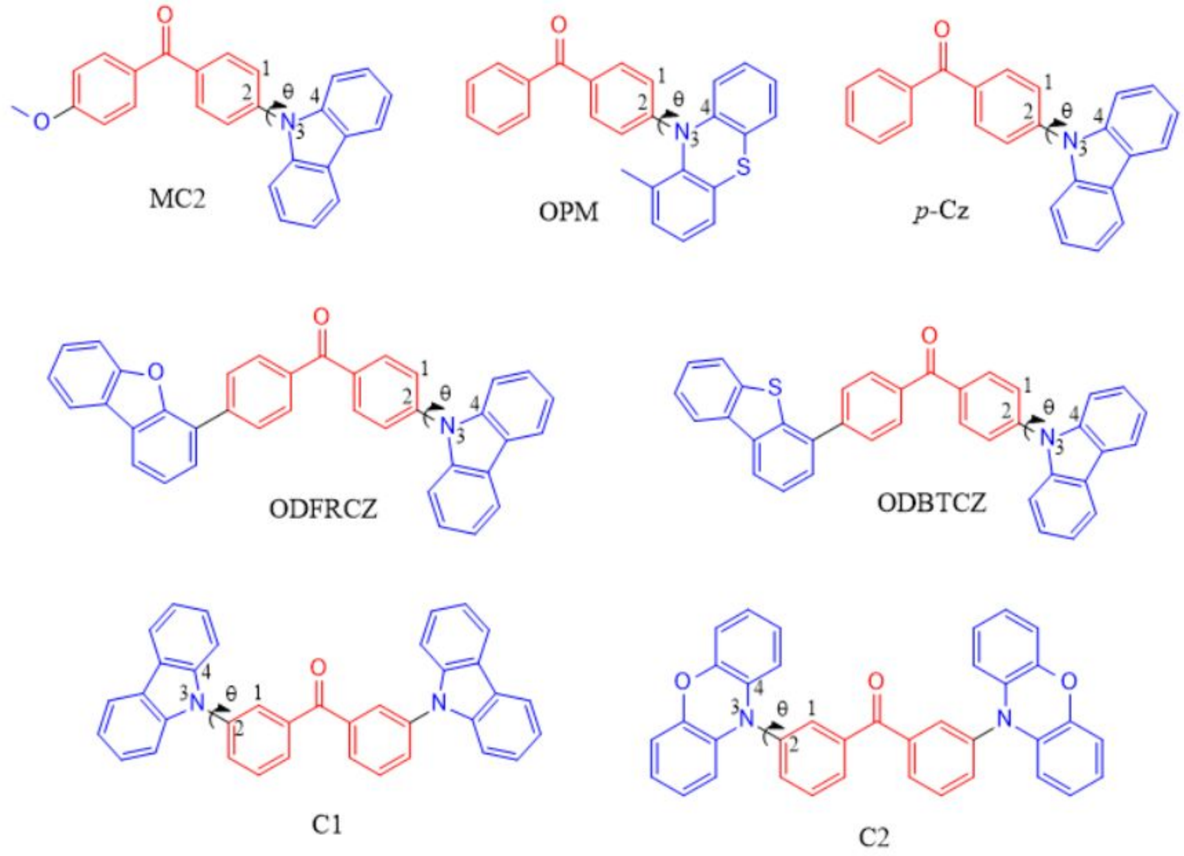

Figure S13. Non-TADF emitters together with their analyzed torsion angles.

Table S18. Measured torsion angles of the S_0 and T_1 optimized geometries of the investigated compounds.

Group	Compound	Torsion Angle (θ) from the S_0 Optimized Geometry	Torsion Angle (θ) from the T_1 Optimized Geometry
Group 1	Px2BP	76.41	107.90
	DMAC-BP	90.29	90.91
	Cz2BP	51.43	48.19
	CC2BP	54.35	43.33
	A-BP-TA	89.41	38.90
	OPDPO	76.93	90.06
Group 2	DBT-BZ-PXZ	76.10	83.84
	DBT-BZ-PTZ	101.79	90.21
	DBT-BZ-DMAC	90.14	90.11
	CP-BP-PXZ	77.26	93.80
	CP-BP-DMAC	89.82	88.90
Group 3	a1	28.01	30.54
	a2	28.81	33.32
	a3	47.84	45.42
	a4	90.95	90.67
	b1	36.16	39.68
	b4	89.40	91.00
Group 4	ACRXTN	87.79	90.21
	MCz-XT	106.62	92.89
	3-PXZ-XO	75.81	74.04
	PTZ-XT	101.64	91.49
Non-TADF	MC2	52.06	41.45
	OPM	21.76	10.05
	p-Cz	51.59	43.66
	ODFRCZ	51.45	43.69
	ODBTCZ	52.73	54.20
	C1	53.63	41.19
	C2	79.38	95.14

Occupied and Virtual NTOs

Table S19. Hole and electron NTOs for Px2BP, DMAC-BP and Cz2BP (TDA: B3LYP/6-31+G(d,p)).

Compound	Hole	Electron
Px2BP	**S_0 Optimization**	
	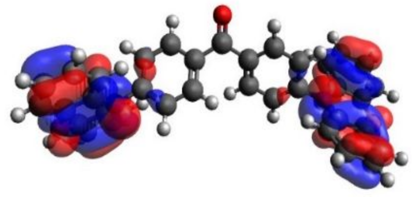	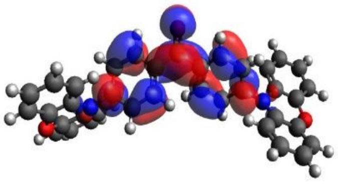
	**T_1 Optimization**	
	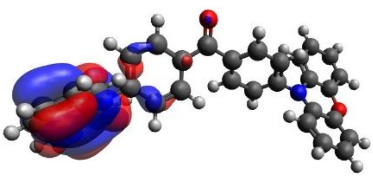	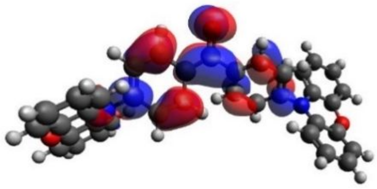
DMAC-BP	**S_0 Optimization**	
	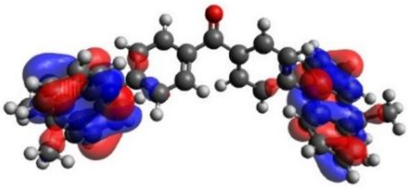	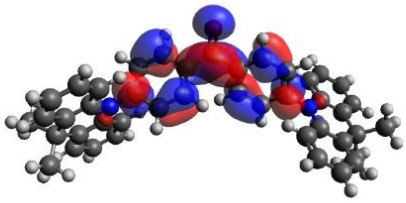
	**T_1 Optimization**	
	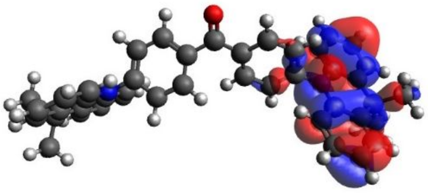	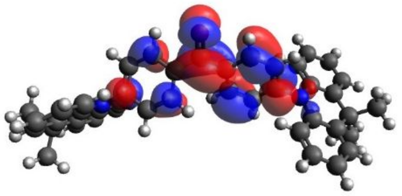
Cz2BP	**S_0 Optimization**	
	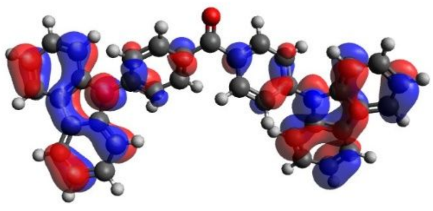	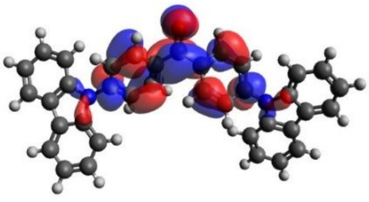
	**T_1 Optimization**	
	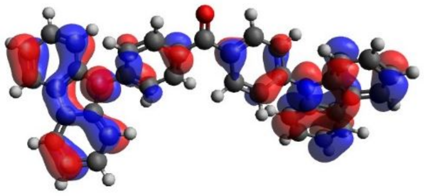	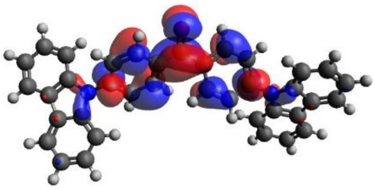

Table S20. Hole and electron NTOs for CC2BP, A-BP-TA and OPDPO (TDA: B3LYP/6-31+G(d,p)).

Compound	Hole	Electron
CC2BP	**S_0 Optimization**	
	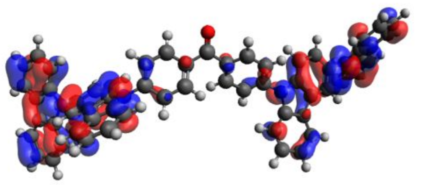	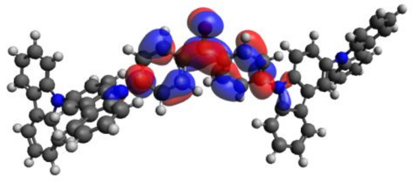
	**T_1 Optimization**	
	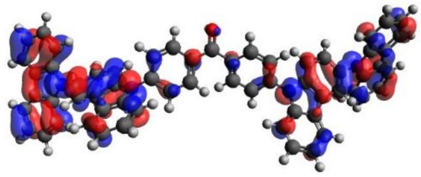	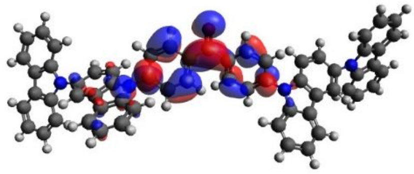
A-BP-TA	**S_0 Optimization**	
	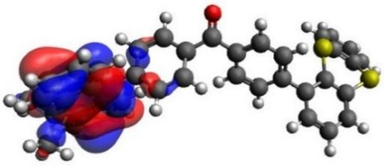	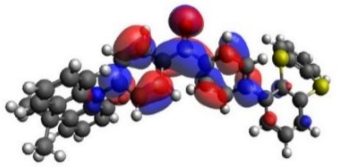
	**T_1 Optimization**	
	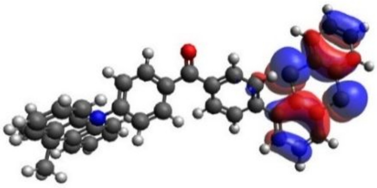	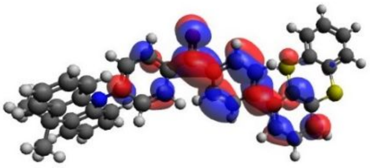
OPDPO	**S_0 Optimization**	
	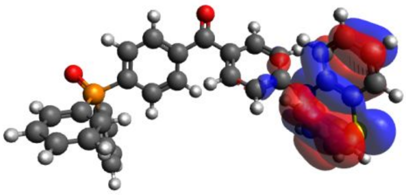	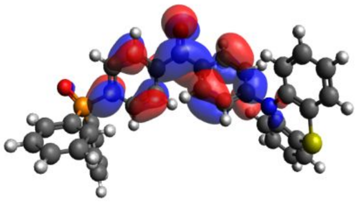
	**T_1 Optimization**	
	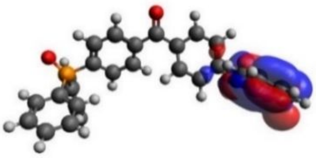	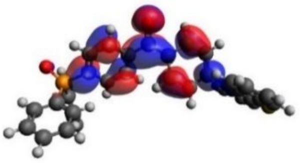

Table S21. Hole and electron NTOs for DBT-BZ-PXZ, DBT-BZ-PTZ and DBT-BZ-DMAC (TDA: B3LYP/6-31+G(d,p)).

**Molecule**	**Hole**	**Electron**
DBT-BZ-PXZ	**S_0 Optimization**	
	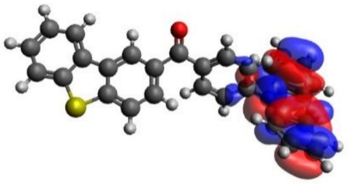	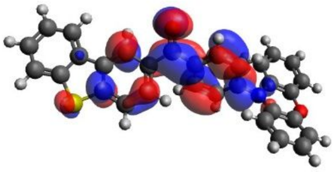
	**T_1 Optimization**	
	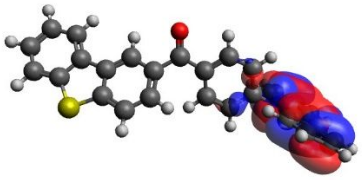	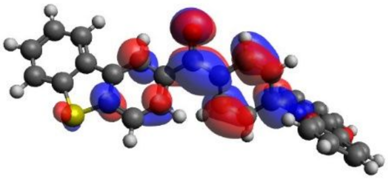
DBT-BZ-PTZ	**S_0 Optimization**	
	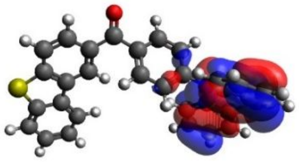	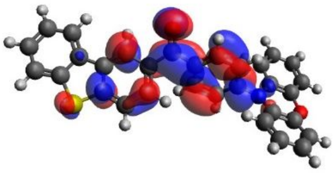
	**T_1 Optimization**	
	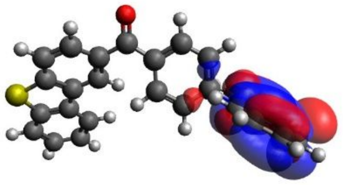	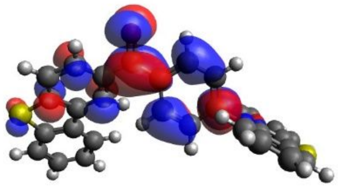
DBT-BZ-DMAC	**S_0 Optimization**	
	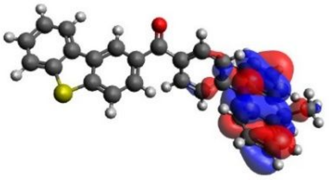	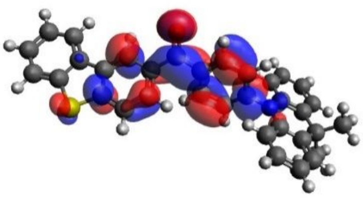
	**T_1 Optimization**	
	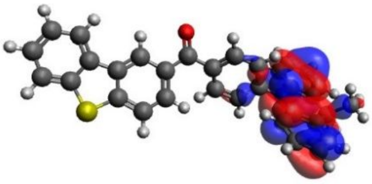	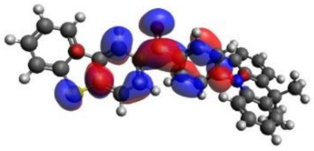

Table S22. Hole and electron NTOs for CP-BP-PXZ and CP-BP-DMAC. (TDA: B3LYP/6-31+G(d,p)).

Molecule	Hole	Electron
CP-BP-PXZ	**S_0 Optimization**	
	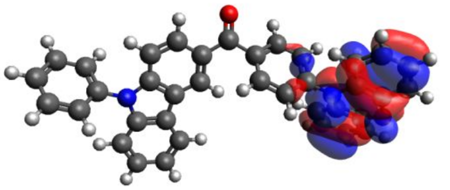	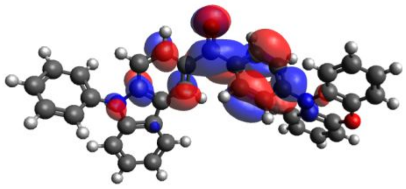
	**T_1 Optimization**	
	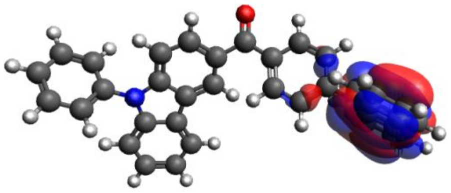	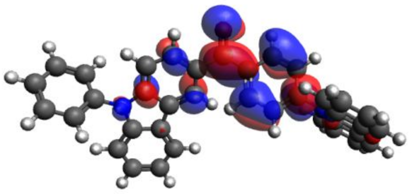
CP-BP-DMAC	**S_0 Optimization**	
	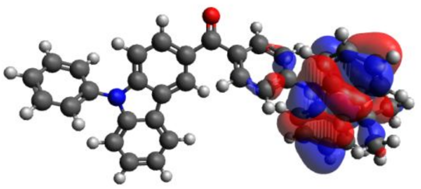	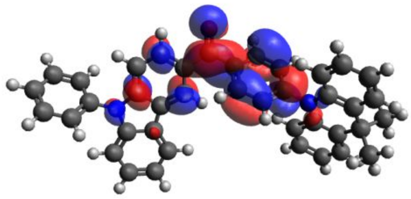
	**T_1 Optimization**	
	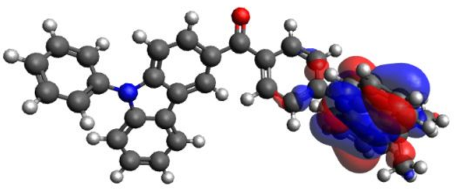	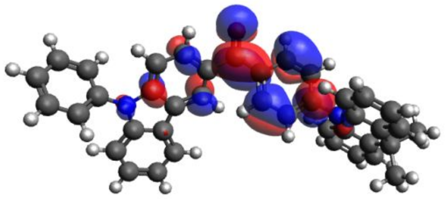

Table S23. Hole and electron NTOs for a1, a2 and a3. (TDA: B3LYP/6-31+G(d,p)).

Molecule	Hole	Electron
a1	S_0 Optimization	
	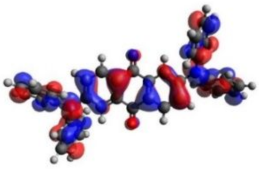	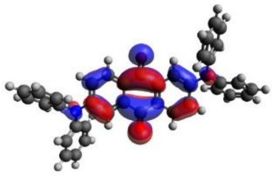
	T_1 Optimization	
	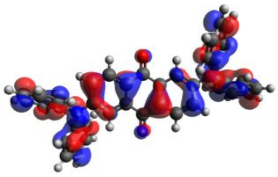	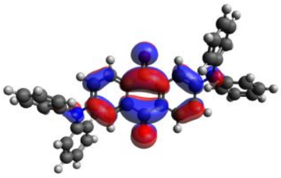
a2	S_0 Optimization	
	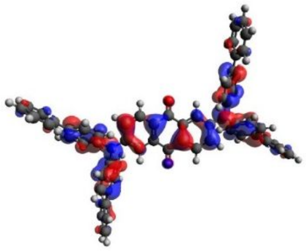	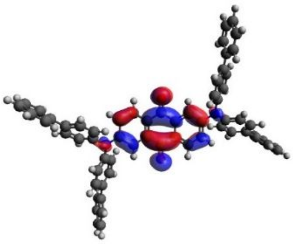
	T_1 Optimization	
	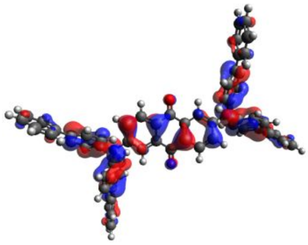	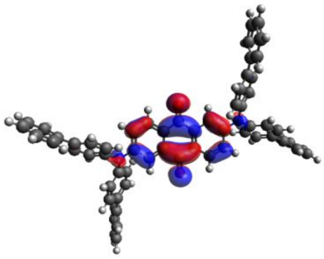
a3	S_0 Optimization	
	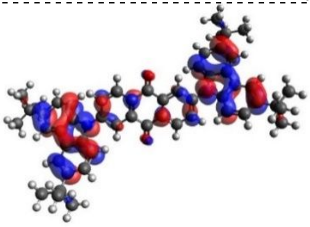	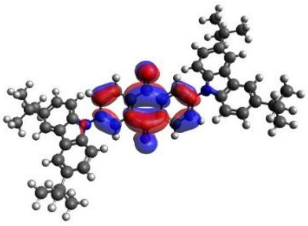
	T_1 Optimization	
	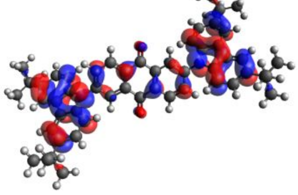	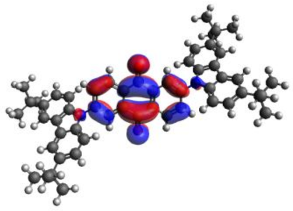

Table S24. Hole and electron NTOs for a4, b1 and b4. (TDA: B3LYP/6-31+G(d,p)).

Molecule	Hole	Electron
a4	**S_0 Optimization**	
	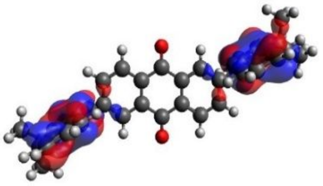	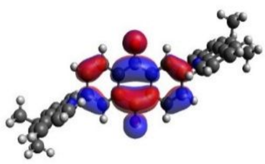
	**T_1 Optimization**	
	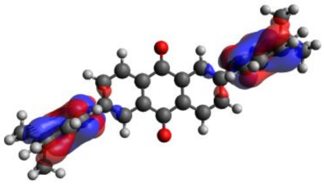	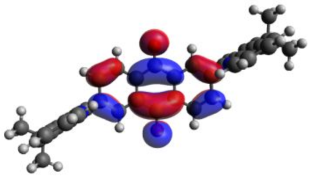
b1	**S_0 Optimization**	
	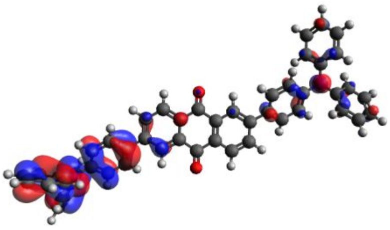	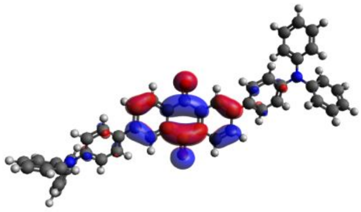
	**T_1 Optimization**	
	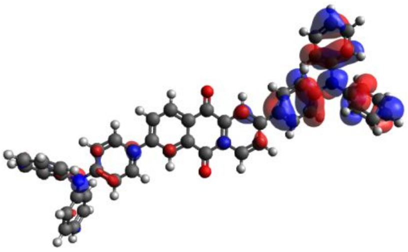	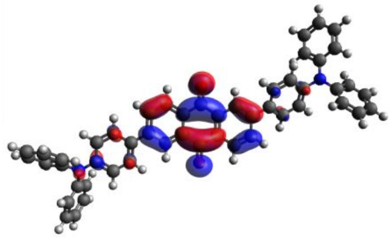
b4	**S_0 Optimization**	
	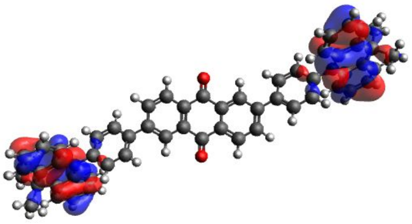	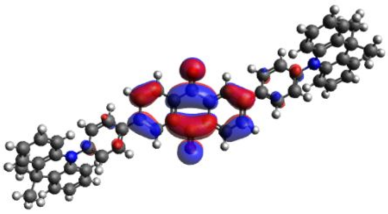
	**T_1 Optimization**	
	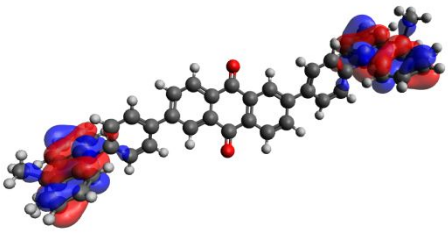	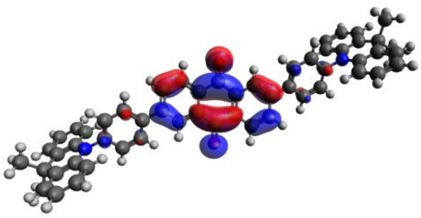

Table S25. Hole and electron NTOs for ACRXTN, MCz-XT and 3-PXZ-XO (TDA: B3LYP/6-31+G(d,p)).

Molecule	Hole	Electron
ACRXTN	**S_0 Optimization**	
	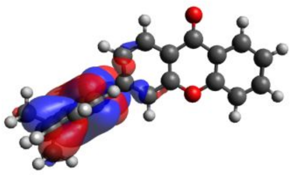	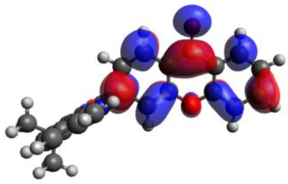
	**T_1 Optimization**	
	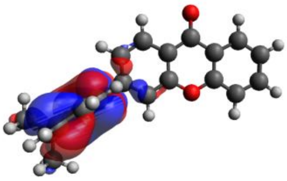	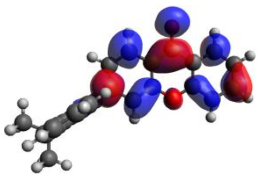
MCz-XT	**S_0 Optimization**	
	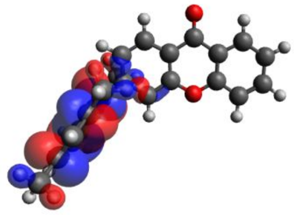	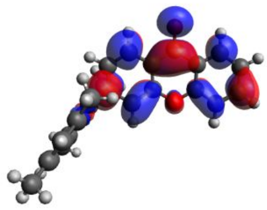
	**T_1 Optimization**	
	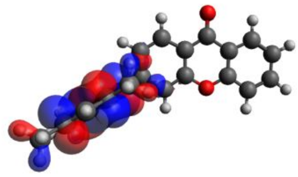	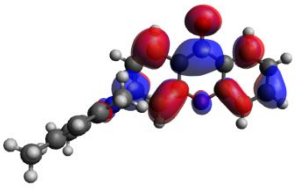
3-PXZ-XO	**S_0 Optimization**	
	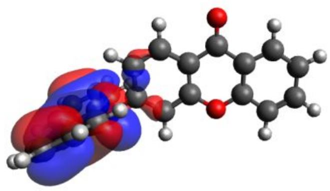	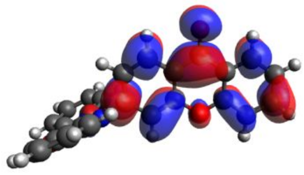
	**T_1 Optimization**	
	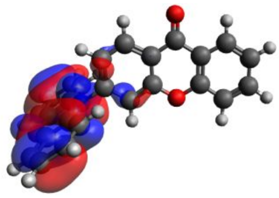	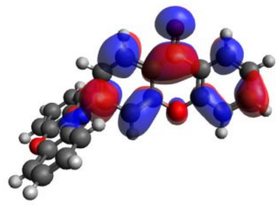

Table S26. Hole and electron NTOs for PTZ-XT, MC2 and OPM (TDA: B3LYP/6-31+G(d,p)).

Molecule	Hole	Electron
PTZ-XT	**S_0 Optimization**	
	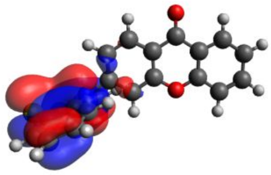	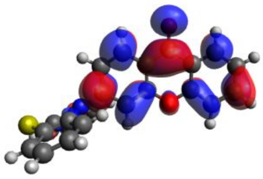
	**T_1 Optimization**	
	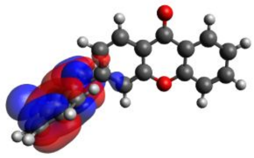	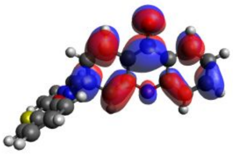
MC2	**S_0 Optimization**	
	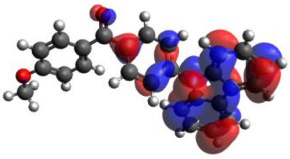	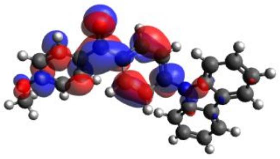
	**T_1 Optimization**	
	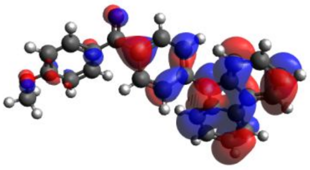	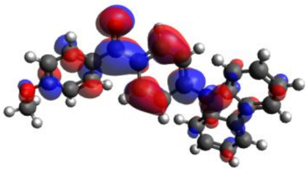
OPM	**S_0 Optimization**	
	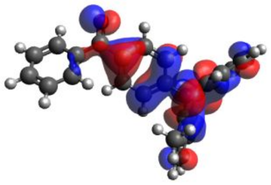	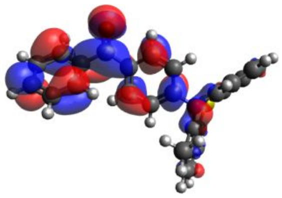
	**T_1 Optimization**	
	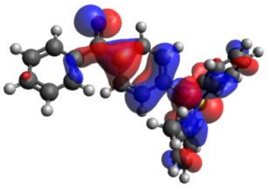	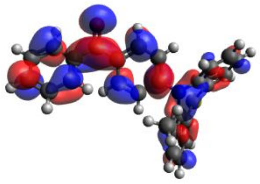

Table S27. Hole and electron NTOs for *p*-Cz, ODFRCZ and ODBTCZ (TDA: B3LYP/6-31+G(d,p)).

Molecule	Hole	Electron
p-Cz	**S_0 Optimization**	
	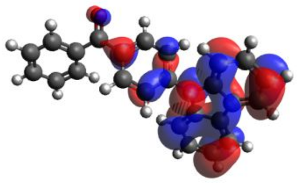	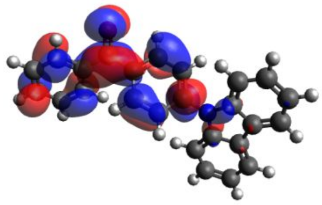
	**T_1 Optimization**	
	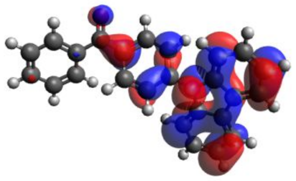	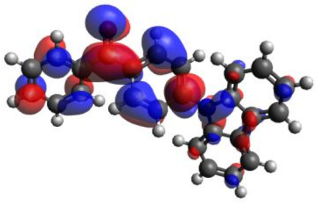
ODFRCZ	**S_0 Optimization**	
	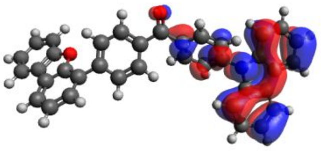	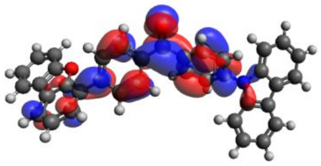
	**T_1 Optimization**	
	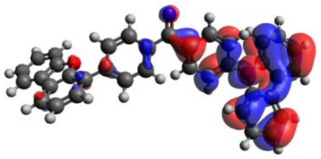	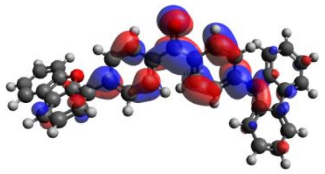
ODBTCZ	**S_0 Optimization**	
	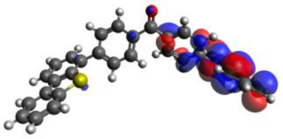	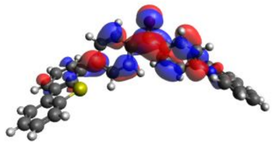
	**T_1 Optimization**	
	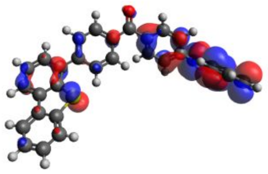	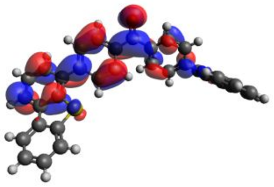

Table S28. Hole and electron NTOs for C1 and C2 (TDA: B3LYP/6-31+G(d,p)).

Molecule	Hole	Electron
C1	**S_0 Optimization**	
	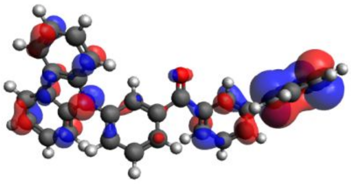	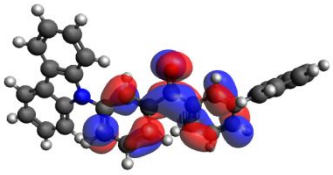
	**T_1 Optimization**	
	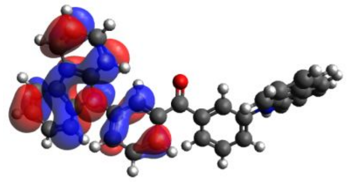	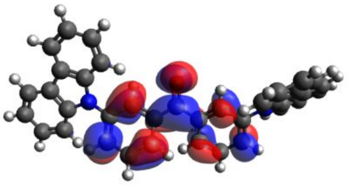
C2	**S_0 Optimization**	
	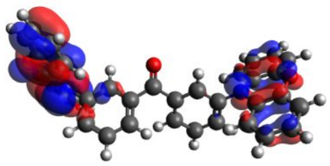	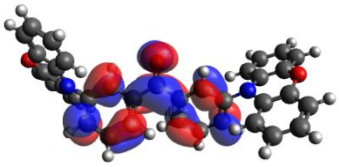
	**T_1 Optimization**	
	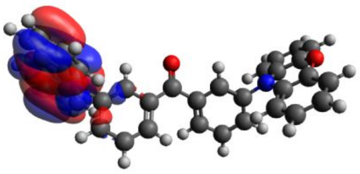	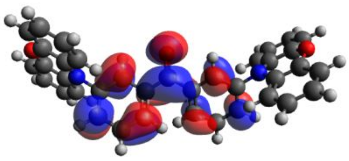

Φ_s Indices

Table S29. Φ_s indices for Group 1 emitters in Lowdin (L) and Mulliken (M) charge distributions for the excitations from S_0 to S_1 calculated with different functionals.

Compound	Transition	Method	S_0 Geometry		T_1 Geometry	
			L	M	L	M
Px2BP	$S_0 \rightarrow S_1$	BLYP	0.2055	0.3682	0.3708	0.4718
		B3LYP	0.2191	0.3793	0.6097	0.7479
		PBE0	0.2594	0.4034	0.5827	0.7093
DMAC-BP	$S_0 \rightarrow S_1$	BLYP	0.2250	0.3392	0.1939	0.2765
		B3LYP	0.2488	0.3519	0.1991	0.2920
		PBE0	0.2515	0.3240	0.2169	0.2911
Cz2BP	$S_0 \rightarrow S_1$	BLYP	0.3584	0.4066	0.4085	0.4703
		B3LYP	0.4209	0.4763	0.4603	0.5073
		PBE0	0.4596	0.5099	0.4904	0.5269
CC2BP	$S_0 \rightarrow S_1$	BLYP	0.1807	0.2195	0.2586	0.2972
		B3LYP	0.3059	0.3566	0.5206	0.6021
		PBE0	0.3703	0.4126	0.5681	0.6363
A-BP-TA	$S_0 \rightarrow S_1$	BLYP	0.0642	0.1109	0.1226	0.1942
		B3LYP	0.0943	0.1396	0.4509	0.5170
		PBE0	0.1208	0.1489	0.4743	0.5342
OPDPO	$S_0 \rightarrow S_1$	BLYP	0.2336	0.2336	0.1213	0.1477
		B3LYP	0.2019	0.2609	0.1009	0.1265
		PBE0	0.2183	0.2777	0.1255	0.1427

Table S30. Φ_s indices for Group 2 emitters in Lowdin (L) and Mulliken (M) charge distributions for the excitations from S_0 to S_1 calculated with different functionals.

Compound	Transition	Method	S_0 Geometry		T_1 Geometry	
			L	M	L	M
DBT-BZ-PXZ	$S_0 \rightarrow S_1$	BLYP	0.1763	0.2764	0.1633	0.2490
		B3LYP	0.1667	0.2660	0.1279	0.2146
		PBE0	0.1774	0.2641	0.1318	0.2060
DBT-BZ-PTZ	$S_0 \rightarrow S_1$	BLYP	0.1991	0.3187	0.1372	0.1827
		B3LYP	0.2825	0.4175	0.1480	0.2004
		PBE0	0.3762	0.4940	0.1531	0.1988
DBT-BZ-DMAC	$S_0 \rightarrow S_1$	BLYP	0.1057	0.1781	0.1359	0.1978
		B3LYP	0.1080	0.1814	0.1967	0.2656
		PBE0	0.1274	0.1767	0.1998	0.2473
CP-BP-PXZ	$S_0 \rightarrow S_1$	BLYP	0.1743	0.2630	0.1425	0.2189
		B3LYP	0.1994	0.2907	0.1494	0.2357
		PBE0	0.2185	0.2949	0.1695	0.2403
CP-BP-DMAC	$S_0 \rightarrow S_1$	BLYP	0.1162	0.1958	0.1163	0.1753
		B3LYP	0.1618	0.2473	0.1514	0.2138
		PBE0	0.1634	0.2198	0.1639	0.2136

Table S31. Φ_s indices for Group 3 emitters in Lowdin (L) and Mulliken (M) charge distributions for the excitations from S_0 to S_1 calculated with different functionals.

Compound	Transition	Method	S_0 Geometry		T_1 Geometry	
			L	M	L	M
a1	$S_0 \rightarrow S_1$	BLYP	0.6604	0.7240	0.6843	0.7351
		B3LYP	0.6685	0.7327	0.6839	0.7329
		PBE0	0.6625	0.7143	0.6833	0.7260
a2	$S_0 \rightarrow S_1$	BLYP	0.6932	0.7263	0.6253	0.7404
		B3LYP	0.6704	0.7094	0.6992	0.7284
		PBE0	0.6780	0.7128	0.7124	0.7476
a3	$S_0 \rightarrow S_1$	BLYP	0.4419	0.4952	0.4780	0.5468
		B3LYP	0.5299	0.5520	0.5689	0.5896
		PBE0	0.5827	0.5973	0.6219	0.6335
a4	$S_0 \rightarrow S_1$	BLYP	0.0444	0.0475	0.0497	0.0552
		B3LYP	0.0607	0.0634	0.0546	0.0591
		PBE0	0.0611	0.0601	0.0597	0.0614
b1	$S_0 \rightarrow S_1$	BLYP	0.4099	0.4517	0.3369	0.3588
		B3LYP	0.3682	0.3996	0.3627	0.3784
		PBE0	0.4419	0.4656	0.5492	0.5688
b4	$S_0 \rightarrow S_1$	BLYP	0.0262	0.0549	0.0408	0.0730
		B3LYP	0.0929	0.1262	0.0544	0.0862
		PBE0	0.1075	0.1335	0.0817	0.1082

Table S32. Φ_s indices for Group 4 emitters in Lowdin (L) and Mulliken (M) charge distributions for the excitations from S_0 to S_1 calculated with different functionals.

Compound	Transition	Method	S_0 Geometry		T_1 Geometry	
			L	M	L	M
ACRXTN	$S_0 \rightarrow S_1$	BLYP	0.0460	0.0622	0.0516	0.0628
		B3LYP	0.0610	0.0820	0.0595	0.0711
		PBE0	0.0803	0.1056	0.0665	0.0767
MCz-XT	$S_0 \rightarrow S_1$	BLYP	0.0682	0.1062	0.1272	0.1371
		B3LYP	0.1038	0.1531	0.1199	0.1294
		PBE0	0.1117	0.1402	0.1244	0.1323
3-PXZ-XO	$S_0 \rightarrow S_1$	BLYP	0.0507	0.0885	0.3427	0.4065
		B3LYP	0.0767	0.1311	0.3243	0.3972
		PBE0	0.0918	0.1535	0.2937	0.3537
PTZ-XT	$S_0 \rightarrow S_1$	BLYP	0.0776	0.1619	0.0706	0.0757
		B3LYP	0.1028	0.1755	0.0721	0.0760
		PBE0	0.1178	0.1796	0.0810	0.0841

Table S33. Φ_s indices for non-TADF emitters in Lowdin (L) and Mulliken (M) charge distributions for the excitations from S_0 to S_1 calculated with different functionals.

Compound	Transition	Method	S_0 Geometry		T_1 Geometry	
			L	M	L	M
MC2	$S_0 \rightarrow S_1$	BLYP	0.4323	0.4719	0.5595	0.5967
		B3LYP	0.4643	0.5079	0.5879	0.6259
		PBE0	0.4913	0.5304	0.6026	0.6384
OPM	$S_0 \rightarrow S_1$	BLYP	0.4026	0.4926	0.4907	0.5580
		B3LYP	0.5535	0.6354	0.6566	0.7241
		PBE0	0.5611	0.6271	0.6043	0.6658
p-Cz	$S_0 \rightarrow S_1$	BLYP	0.3970	0.4344	0.5410	0.5907
		B3LYP	0.4389	0.4847	0.5675	0.6088
		PBE0	0.4885	0.5292	0.5923	0.6335
ODFRCZ	$S_0 \rightarrow S_1$	BLYP	0.3811	0.4128	0.5612	0.6112
		B3LYP	0.4664	0.5039	0.5936	0.6435
		PBE0	0.4965	0.5273	0.6101	0.6540
ODBTCZ	$S_0 \rightarrow S_1$	BLYP	0.4692	0.4929	0.4296	0.4722
		B3LYP	0.5648	0.5846	0.4911	0.5257
		PBE0	0.5989	0.6107	0.5548	0.5862
C1	$S_0 \rightarrow S_1$	BLYP	0.3273	0.3846	0.2953	0.3449
		B3LYP	0.3624	0.4358	0.4369	0.5282
		PBE0	0.4214	0.4902	0.4764	0.5549
C2	$S_0 \rightarrow S_1$	BLYP	0.5548	0.6764	0.3857	0.4607
		B3LYP	0.5380	0.6559	0.4208	0.5038
		PBE0	0.4828	0.5937	0.4114	0.4866

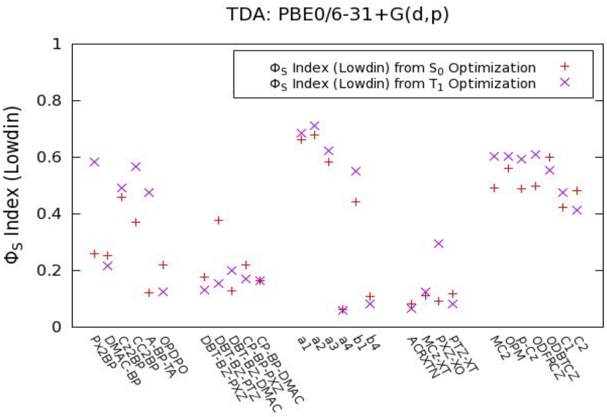

Figure S14. Φ_s indices for the TADF emitters calculated from S_0 and T_1 geometries with PBE0/6-31+G(d,p).

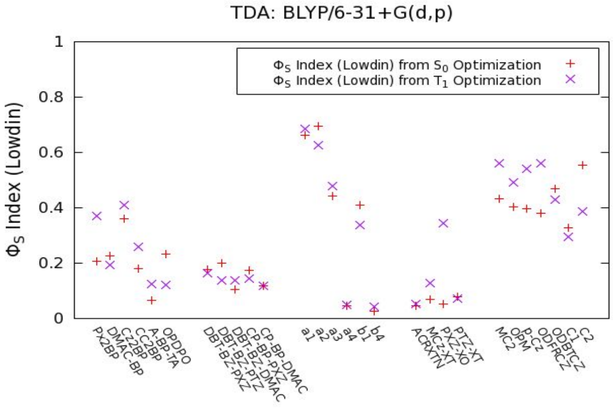

Figure S15. Φ_s indices for the TADF emitters calculated from S_0 and T_1 geometries with BLYP/6-31+G(d,p).

Low Lying Singlet-Triplet Energy Gaps

Table S34. Low lying singlet-triplet energy gaps (ΔE_{S1-T1}, ΔE_{S1-T2}) in eV for Group 1 emitters together with the experimental values.

Compound	Method	S_0 Geometry		T_1 Geometry		Exp. ΔE_{ST}
		ΔE_{S1-T1}	ΔE_{S1-T2}	ΔE_{S1-T1}	ΔE_{S1-T2}	
Px2BP	BLYP	0.004	0.002	0.165	-	0.03
	B3LYP	0.007	0.005	0.104	-	
	PBE0	0.010	0.007	0.106	-	
DMAC-BP	BLYP	0.004	0.001	0.014	-	0.07
	B3LYP	0.006	0.003	0.013	-	
	PBE0	0.009	0.006	0.017	-	
Cz2BP	BLYP	0.099	0.090	0.149	0.138	0.21
	B3LYP	0.232	0.165	0.278	0.224	
	PBE0	0.321	0.216	0.366	0.271	
CC2BP	BLYP	0.017	0.013	0.015	-	0.14
	B3LYP	0.085	0.069	0.284	0.013	
	PBE0	0.165	0.114	0.406	0.025	
A-BP-TA	BLYP	0.004	-	0.066	0.002	0.06
	B3LYP	0.006	-	0.225	-	
	PBE0	0.008	-	0.275	-	
OPDPO	BLYP	0.003	-	0.003	-	0.02
	B3LYP	0.006	-	0.004	-	
	PBE0	0.010	-	0.005	-	

Table S35. Low lying singlet-triplet energy gaps (ΔE_{S1-T1}) in eV for Group 2 emitters together with the experimental values.

Compound	Method	S_0 Geometry	T_1 Geometry	Exp. ΔE_{ST}
		ΔE_{S1-T1}	ΔE_{S1-T1}	
DBT-BZ-PXZ	BLYP	0.004	0.026	0.09
	B3LYP	0.008	0.018	
	PBE0	0.011	0.020	
DBT-BZ-PTZ	BLYP	0.004	0.004	0.05
	B3LYP	0.009	0.005	
	PBE0	0.015	0.007	
DBT-BZ-DMAC	BLYP	0.004	0.002	0.08
	B3LYP	0.006	0.004	
	PBE0	0.009	0.009	
CP-BP-PXZ	BLYP	0.005	0.011	0.024
	B3LYP	0.009	0.011	
	PBE0	0.013	0.013	
CP-BP-DMAC	BLYP	0.004	0.006	0.016
	B3LYP	0.007	0.008	
	PBE0	0.010	0.011	

Table S36. Low lying singlet-triplet energy gaps (ΔE_{S1-T1}, ΔE_{S1-T2}) in eV for Group 3 emitters together with the experimental values.

Compound	Method	S_0 Geometry		T_1 Geometry		Exp. ΔE_{ST}
		ΔE_{S1-T1}	ΔE_{S1-T2}	ΔE_{S1-T1}	ΔE_{S1-T2}	
a1	BLYP	0.375	0.254	0.440	0.309	0.29
	B3LYP	0.443	0.284	0.481	0.302	
	PBE0	0.483	0.317	0.513	0.324	
a2	BLYP	0.338	0.238	0.376	0.277	0.27
	B3LYP	0.398	0.258	0.428	0.280	
	PBE0	0.435	0.286	0.455	0.295	
a3	BLYP	0.182	0.138	0.239	0.183	0.17
	B3LYP	0.211	0.138	0.272	0.177	
	PBE0	0.234	0.151	0.291	0.184	
a4	BLYP	0.005	0.004	0.005	0.004	0.08
	B3LYP	0.008	0.004	0.007	0.004	
	PBE0	0.010	0.005	0.008	0.004	
b1	BLYP	0.107	0.085	0.127	0.088	0.24
	B3LYP	0.150	0.104	0.346	0.110	
	PBE0	0.179	0.124	0.400	0.097	
b4	BLYP	0.001	0.001	0.002	0.002	0.07
	B3LYP	0.001	0.001	0.001	0.001	
	PBE0	0.001	0.001	0.001	0.001	

Table S37. Low lying singlet-triplet energy gaps (ΔE_{S1-T1}) in eV for Group 4 emitters together with the experimental values.

Compound	Method	S_0 Geometry	T_1 Geometry	Exp. ΔE_{ST}
		ΔE_{S1-T1}	ΔE_{S1-T1}	
ACRXTN	BLYP	0.006	0.006	0.06
	B3LYP	0.010	0.008	
	PBE0	0.013	0.011	
MCz-XT	BLYP	0.006	0.009	0.011
	B3LYP	0.009	0.011	
	PBE0	0.012	0.014	
3-PXZ-XO	BLYP	0.006	0.089	0.036
	B3LYP	0.011	0.066	
	PBE0	0.015	0.070	
PTZ-XT	BLYP	0.006	0.012	0.071
	B3LYP	0.013	0.018	
	PBE0	0.020	0.022	

Table S38. Low lying singlet-triplet energy gaps ($\Delta E_{S1\text{-}T1}$, $\Delta E_{S1\text{-}T2}$) in eV for non-TADF emitters together with the experimental values.

**Compound**	**Method**	**S_0 Geometry**		**T_1 Geometry**		**Exp. ΔE_{ST}**
		**$\Delta E_{S1\text{-}T1}$**	**$\Delta E_{S1\text{-}T2}$**	**$\Delta E_{S1\text{-}T1}$**	**$\Delta E_{S1\text{-}T2}$**	
MC2	BLYP	0.152	-	0.445	0.118	0.23
	B3LYP	0.289	0.037	0.651	0.152	
	PBE0	0.392	0.170	0.743	0.241	
OPM	BLYP	0.338	-	0.595	0.220	-
	B3LYP	0.667	0.307	0.944	0.350	
	PBE0	0.755	0.436	1.019	0.422	
p-Cz	BLYP	0.164	-	0.443	-	0.61
	B3LYP	0.275	-	0.613	0.011	
	PBE0	0.358	0.053	0.697	0.127	
ODFRCZ	BLYP	0.131	-	0.372	-	-
	B3LYP	0.238	0.038	0.553	0.024	
	PBE0	0.322	0.187	0.637	0.124	
ODBTCZ	BLYP	0.124	-	0.059	-	-
	B3LYP	0.218	0.048	0.591	-	
	PBE0	0.292	0.179	0.765	-	
C1	BLYP	0.060	0.057	0.101	-	-
	B3LYP	0.123	0.118	0.283	-	
	PBE0	0.161	0.156	0.343	-	
C2	BLYP	0.012	-	0.011	-	0.57
	B3LYP	0.019	-	0.016	-	
	PBE0	0.026	0.004	0.019	-	

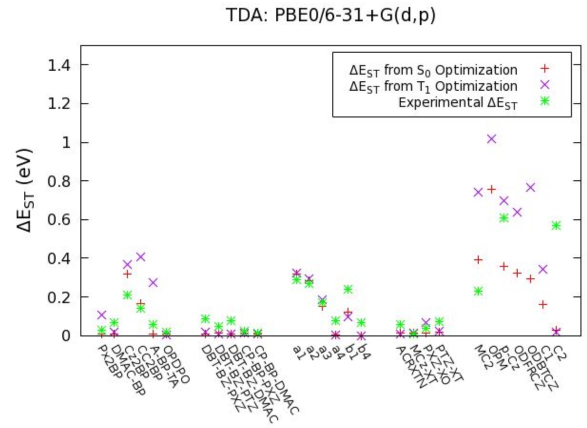

Figure S16. ΔE_{ST} values for TADF emitters calculated from S_0 and T_1 geometries with PBE0/6-31+G(d,p). (Experimental ΔE_{ST} values also included)

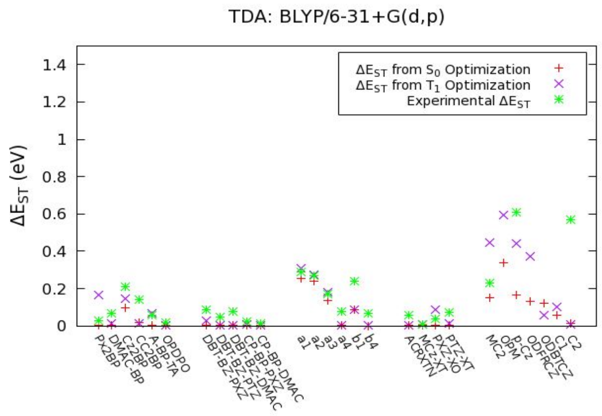

Figure S17. ΔE_{ST} values for TADF emitters calculated from S_0 and T_1 geometries with BLYP/6-31+G(d,p). (Experimental ΔE_{ST} values also included)

Spin-Orbit Coupling Values

Table S39. Spin-orbit coupling (SOC) values between different energy states for the T_1 geometries of Group 1 emitters.

**Compound**	**Method**	**SOC**	
		**S_1-T_1**	**S_1-T_2**
Px2BP	BLYP	0.060	-
	B3LYP	0.073	-
	PBE0	0.086	-
DMAC-BP	BLYP	0.020	-
	B3LYP	0.037	-
	PBE0	0.045	-
Cz2BP	BLYP	0.057	0.158
	B3LYP	0.465	0.105
	PBE0	0.565	0.117
CC2BP	BLYP	0.048	-
	B3LYP	0.942	0.868
	PBE0	1.231	2.142
A-BP-TA	BLYP	0.764	-
	B3LYP	0.999	-
	PBE0	1.068	0.291
OPDPO	BLYP	0.003	-
	B3LYP	0.004	-
	PBE0	0.004	-

Table S40. Spin-orbit coupling (SOC) values between S_1 and T_1 for the T_1 geometries of Group 2 emitters.

**Compound**	**Method**	**SOC**
		**S_1-T_1**
DBT-BZ-PXZ	BLYP	0.040
	B3LYP	0.042
	PBE0	0.046
DBT-BZ-PTZ	BLYP	0.002
	B3LYP	0.002
	PBE0	0.002
DBT-BZ-DMAC	BLYP	0.005
	B3LYP	0.026
	PBE0	0.446
CP-BP-PXZ	BLYP	0.022
	B3LYP	0.026
	PBE0	0.028
CP-BP-DMAC	BLYP	0.004
	B3LYP	0.009
	PBE0	0.013

Table S41. Spin-orbit coupling (SOC) values between different energy states for the T_1 geometries of Group 3 emitters.

**Compound**	**Method**	**SOC**	
		**S_1-T_1**	**S_1-T_2**
a1	BLYP	0.216	0.000
	B3LYP	0.144	0.000
	PBE0	0.163	0.000
a2	BLYP	0.000	0.117
	B3LYP	0.120	0.000
	PBE0	0.109	0.000
a3	BLYP	0.000	0.079
	B3LYP	0.134	0.000
	PBE0	0.138	0.000
a4	BLYP	0.016	0.000
	B3LYP	0.025	0.000
	PBE0	0.031	0.000
b1	BLYP	0.057	0.023
	B3LYP	0.097	0.059
	PBE0	0.072	0.006
b4	BLYP	0.000	0.004
	B3LYP	0.000	0.004
	PBE0	0.000	0.005

Table S42. Spin-orbit coupling (SOC) values between S_1 and T_1 for the T_1 geometries of Group 4 emitters.

**Compound**	**Method**	**SOC**
		**S_1-T_1**
ACRXTN	BLYP	0.016
	B3LYP	0.025
	PBE0	0.029
MCz-XT	BLYP	0.018
	B3LYP	0.026
	PBE0	0.029
3-PXZ-XO	BLYP	0.047
	B3LYP	0.060
	PBE0	0.065
PTZ-XT	BLYP	0.005
	B3LYP	0.022
	PBE0	0.033

Table S43. Spin-orbit coupling (SOC) values between different energy states for the T_1 geometries of non-TADF emitters.

**Compound**	**Method**	**SOC**	
		**S_1-T_1**	**S_1-T_2**
MC2	BLYP	1.390	2.878
	B3LYP	0.617	4.203
	PBE0	0.435	4.494
OPM	BLYP	1.581	4.635
	B3LYP	2.070	8.383
	PBE0	8.381	7.830
p-Cz	BLYP	0.335	-
	B3LYP	0.246	5.306
	PBE0	0.274	5.500
ODFRCZ	BLYP	0.321	-
	B3LYP	0.345	3.449
	PBE0	0.299	3.825
ODBTCZ	BLYP	0.314	-
	B3LYP	1.078	-
	PBE0	1.262	-
C1	BLYP	0.121	-
	B3LYP	0.202	-
	PBE0	0.231	-
C2	BLYP	0.011	-
	B3LYP	0.015	-
	PBE0	0.016	-

S_0 and T_1 cartesian coordinates of the most stable conformations optimized at M06-2X/6-31+G(d,p) level of theory

Px2BP-S_0 Geometry (Solvent: Toluene)

H	-0.59472200	1.10720700	0.14573100
C	-1.44306100	0.61872900	-0.32346600
C	-3.64281900	-0.59585800	-1.54508700
C	-1.29756300	-0.00870600	-1.56412400
C	-2.68862600	0.64760800	0.30237500
C	-3.78064200	0.03514500	-0.30518500
C	-2.40674600	-0.60615200	-2.17693100
H	-2.82566800	1.14295300	1.25812800
H	-2.28136900	-1.07290100	-3.14857300
H	-4.50954400	-1.06478400	-2.00237100
C	0.00007700	-0.00224000	-2.31428400
O	0.00012400	-0.00324000	-3.53316900
C	1.29766100	0.00545200	-1.56404200
C	3.78067300	-0.03632800	-0.30489300
C	2.40689300	0.60184900	-2.17779000
C	1.44308200	-0.61989900	-0.32232900
C	2.68862000	-0.64776000	0.30362400
C	3.64292500	0.59260600	-1.54585800
H	2.28156000	1.06697300	-3.15021700
H	0.59472900	-1.10760300	0.14764500
H	2.82560500	-1.14151500	1.26020700
H	4.50967700	1.06077400	-2.00386800
N	5.05243700	-0.06709700	0.34835500
C	6.02449700	-0.97644400	-0.11856500
C	8.03039700	-2.76883700	-0.92791700
C	5.75441000	-1.96078100	-1.07025000
C	7.31741300	-0.90767400	0.42140600
C	8.31199200	-1.78239300	0.01995700
C	6.75205000	-2.85456100	-1.46656900
H	4.76008200	-2.03544600	-1.49639100
H	9.29517300	-1.68389600	0.46799100
H	6.51669300	-3.61614700	-2.20235200
H	8.80758900	-3.45982600	-1.23522100
C	5.51011200	1.10249500	0.98904000
C	6.51594900	3.35321500	2.33659000
C	6.81676700	1.11630500	1.50008800
C	4.71647200	2.23656600	1.16699100
C	5.21682500	3.35212100	1.84262100
C	7.32025900	2.22551600	2.15700300
H	3.70162700	2.24452600	0.78493800
H	4.57893300	4.21943900	1.97571600
H	8.33793100	2.18392100	2.53077500
H	6.90814200	4.21836900	2.85966000
O	7.62475900	0.00370000	1.40870200
N	-5.05246400	0.06713100	0.34786800
C	-6.02391200	0.97672200	-0.11982700
C	-8.02855700	2.76978800	-0.93081200
C	-5.75311100	1.96008600	-1.07231900
C	-7.31694000	0.90920000	0.42004600
C	-8.31088600	1.78428300	0.01782300
C	-6.75012000	2.85421100	-1.46945000
H	-4.75871600	2.03370600	-1.49848500
H	-9.29418200	1.68678400	0.46582200
H	-6.51420700	3.61501500	-2.20586200
H	-8.80526500	3.46105200	-1.23871900
C	-5.51078900	-1.10144400	0.98988200
C	-6.51776800	-3.35007600	2.34008200
C	-6.81752300	-1.11406300	1.50076600
C	-4.71765100	-2.23562300	1.16938600
C	-5.21857200	-3.35014200	1.84630000
C	-7.32156000	-2.22224900	2.15899600

H	-3.70273700	-2.24446100	0.78753100
H	-4.58105600	-4.21755700	1.98056200
H	-8.33926500	-2.17974800	2.53257800
H	-6.91040900	-4.21441700	2.86415800
O	-7.62510600	-0.00129200	1.40789100

Px2BP-T_1 Geometry (Solvent: Toluene)

H	0.62351000	-0.44345300	0.27766100
C	1.46961300	-0.09557400	-0.30427100
C	3.70578000	0.64189700	-1.83205000
C	1.27129300	0.39137300	-1.63610300
C	2.72808600	-0.18694500	0.25053500
C	3.85512900	0.19525700	-0.50401500
C	2.44859100	0.72959700	-2.38291800
H	2.86051900	-0.57653800	1.25704500
H	2.31543500	1.06942600	-3.40370800
H	4.58486600	0.92307200	-2.40759000
C	-0.00004400	0.48304000	-2.30990500
O	-0.06289200	0.67145600	-3.55771700
C	-1.28674200	0.33778000	-1.56227000
C	-3.79699500	0.11208700	-0.31894200
C	-2.37210500	-0.25836900	-2.22877600
C	-1.50020600	0.84727400	-0.27227600
C	-2.74452700	0.73582900	0.34540900
C	-3.61058300	-0.38233900	-1.61272000
H	-2.21815300	-0.61571500	-3.24181000
H	-0.69785400	1.36762000	0.24185200
H	-2.91485300	1.14098400	1.33871900
H	-4.44436800	-0.85607800	-2.12493900
N	-5.07036600	-0.00304800	0.32717600
C	-6.09875800	0.88977100	-0.02374500
C	-8.21941000	2.63715600	-0.62031500
C	-5.90912300	1.96773300	-0.89060900
C	-7.37121300	0.70612000	0.54001500
C	-8.42098400	1.55728100	0.24328000
C	-6.96311900	2.83743400	-1.18046300
H	-4.93219800	2.12796500	-1.33309400
H	-9.38400200	1.36698200	0.70564200
H	-6.78887300	3.67134700	-1.85231700
H	-9.04017600	3.30922200	-0.84585700
C	-5.44051900	-1.23601500	0.89195300
C	-6.27457600	-3.64187300	2.08681500
C	-6.72882600	-1.36920200	1.43433300
C	-4.57827000	-2.33217200	0.96192900
C	-4.99319500	-3.52380800	1.56152300
C	-7.14765100	-2.55317400	2.01542600
H	-3.57839200	-2.24695800	0.55122700
H	-4.30234800	-4.35915200	1.60911700
H	-8.15461300	-2.60081300	2.41686800
H	-6.60064800	-4.56661600	2.55024300
O	-7.60308900	-0.30305700	1.45159200
N	5.16617200	0.10325500	0.07610600
C	6.04337100	-0.88864200	-0.32850300
C	7.88511400	-2.85355800	-1.09117000
C	5.68376800	-1.85852800	-1.28750500
C	7.33272400	-0.94729400	0.24475700
C	8.25213100	-1.92153200	-0.13562000
C	6.59838500	-2.82223300	-1.66001700
H	4.68827000	-1.83123800	-1.71390100
H	9.22973000	-1.92527700	0.33284000
H	6.31595800	-3.56501400	-2.39727700
H	8.59295800	-3.61672000	-1.39469500
C	5.56639300	1.01010300	1.04268200
C	6.45606000	2.79548000	3.00583500

C	6.85612600	0.89678900	1.60651400
C	4.72932300	2.06092000	1.47212900
C	5.17416900	2.93499500	2.44276800
C	7.29992800	1.78168700	2.58599000
H	3.74864100	2.16537600	1.02410200
H	4.52705200	3.74141800	2.76821000
H	8.29843800	1.65423300	2.98827100
H	6.79147300	3.49093400	3.76708000
O	7.71774900	-0.06796200	1.20176400

Px2BP-S_1 Geometry (Solvent: Toluene)

H	-0.70212000	-1.42801000	-0.54322000
C	-1.50172500	-1.00919800	0.06044900
C	-3.58964300	-0.01400200	1.61523800
C	-1.29343000	-0.78268600	1.42633200
C	-2.73648200	-0.73685300	-0.52753300
C	-3.77547800	-0.23037800	0.24745600
C	-2.36179700	-0.30174400	2.19823000
H	-2.90827700	-0.92244100	-1.58359000
H	-2.20765400	-0.16567300	3.26392900
H	-4.41445000	0.37175100	2.20886800
C	-0.00945400	-1.12455300	2.13067300
O	-0.09950400	-1.64589500	3.26772800
C	1.25977100	-0.84678500	1.51102600
C	3.83831400	-0.32987300	0.47435400
C	2.44186200	-1.35401700	2.14857800
C	1.44704500	-0.02752100	0.35255900
C	2.69827200	0.22880600	-0.15720600
C	3.69525500	-1.11554500	1.64583700
H	2.31284800	-1.94533600	3.04869800
H	0.59165900	0.44012000	-0.12330500
H	2.82296600	0.86595400	-1.02836000
H	4.57893800	-1.51950900	2.13292600
N	5.13780900	-0.06976400	-0.05320200
C	5.85557800	1.03539400	0.36859500
C	7.36757100	3.24468000	1.16987000
C	5.33910700	1.93085500	1.32638900
C	7.13987300	1.27689600	-0.17060100
C	7.89332200	2.37544500	0.22635700
C	6.09215400	3.01987500	1.71740500
H	4.35574100	1.74263600	1.73887900
H	8.87328200	2.52254700	-0.21278400
H	5.69288100	3.70617000	2.45484000
H	7.94808700	4.10386600	1.48617800
C	5.69422900	-0.92064900	-0.99167600
C	6.88475000	-2.59023900	-2.89031800
C	6.98115000	-0.64222300	-1.50557400
C	5.00928800	-2.06293000	-1.45179900
C	5.60390700	-2.88308500	-2.38977300
C	7.57495900	-1.47127700	-2.45022600
H	4.02398400	-2.27328000	-1.05496100
H	5.07519600	-3.76126500	-2.74122900
H	8.56340500	-1.22002100	-2.81717000
H	7.34017400	-3.24289900	-3.62642300
O	7.68010700	0.44409600	-1.09407700
N	-5.03862100	0.05374400	-0.36606000
C	-6.09867000	-0.85600000	-0.19811500
C	-8.28040200	-2.61345000	0.03873800
C	-5.94994300	-2.09245100	0.43302400
C	-7.36135400	-0.51859000	-0.71016300
C	-8.44102900	-1.37539100	-0.58863800
C	-7.03437700	-2.96592800	0.54386400
H	-4.98133500	-2.37315700	0.83145400
H	-9.39481600	-1.06284100	-1.00086100
H	-6.89184500	-3.92387300	1.03285600
H	-9.12460200	-3.28865500	0.12585700

C	-5.36516900	1.38826200	-0.66478000
C	-6.11417100	4.01352800	-1.33970800
C	-6.64578100	1.67180600	-1.16572400
C	-4.46709200	2.44612100	-0.51028400
C	-4.83981800	3.74830800	-0.85233100
C	-7.02286600	2.96288200	-1.49098900
H	-3.47239100	2.24610300	-0.12776000
H	-4.12168100	4.55232600	-0.72947800
H	-8.02583000	3.12513200	-1.87194000
H	-6.40749400	5.02380100	-1.60334600
O	-7.55402000	0.66101700	-1.39895300

DMAC-BP-S_0 Geometry (Solvent: DCM)

H	2.24990800	-1.23741800	3.57061900
C	2.38447400	-0.74418300	2.61343400
C	2.69696900	0.56367200	0.16672900
C	1.29510700	-0.09077200	2.02504100
C	3.61732900	-0.76341200	1.97225400
C	3.77053300	-0.10698800	0.74986800
C	1.45631300	0.56451400	0.79895200
H	4.46784600	-1.27892500	2.40795700
H	0.62376400	1.09455800	0.34709700
H	2.84193600	1.07845900	-0.77811900
C	-0.00026700	-0.06140900	2.77597700
O	-0.00093800	-0.09003000	3.99658100
C	-1.29475200	0.00218100	2.02575100
C	-3.76931200	0.07308300	0.75088100
C	-1.45412100	-0.59669500	0.77098700
C	-2.38531000	0.62701100	2.64239300
C	-3.61774700	0.67374600	2.00181100
C	-2.69444000	-0.56887800	0.13858600
H	-0.62046500	-1.10467400	0.29637800
H	-2.25215800	1.07586800	3.62139400
H	-4.46937500	1.16745800	2.46004500
H	-2.83832000	-1.04069900	-0.82858600
N	5.03961000	-0.12066400	0.09215600
C	5.95965400	0.90034300	0.36792800
C	7.76743800	2.96888600	0.95555800
C	5.62557500	1.91244400	1.28718500
C	7.21477100	0.92209700	-0.26643300
C	8.09152500	1.96556100	0.04796600
C	6.52076500	2.93389700	1.57648600
H	4.66013600	1.89871700	1.77884800
H	9.06435900	1.99421100	-0.43471800
H	6.23731000	3.70128300	2.28998600
H	8.47589500	3.76110500	1.17247500
C	5.32526900	-1.14893900	-0.81629900
C	5.84847800	-3.23228200	-2.62717200
C	6.56164000	-1.18843800	-1.48585600
C	4.36797500	-2.15142000	-1.06098900
C	4.62856700	-3.18035900	-1.95643000
C	6.79052100	-2.23864000	-2.38083100
H	3.41479000	-2.12507100	-0.54641200
H	3.87242700	-3.94059200	-2.12568400
H	7.74145000	-2.28135200	-2.90436200
H	6.06492300	-4.03048500	-3.32901300
C	7.65223200	-0.14005700	-1.27326300
C	8.92545800	-0.83782900	-0.74728100
H	9.73318700	-0.11469100	-0.60284400
H	9.27560500	-1.59327300	-1.45645100
H	8.72535900	-1.32836400	0.20986000
C	7.96546300	0.54284100	-2.62232900
H	8.75810700	1.28740100	-2.50636400
H	7.07515600	1.04385100	-3.01366000
H	8.30196500	-0.19057300	-3.36049100
N	-5.03809800	0.11372400	0.09371400

C	-5.96076300	-0.91501300	0.32911300
C	-7.77294200	-3.00172200	0.83271000
C	-7.21628500	-0.90792900	-0.30490000
C	-5.62851000	-1.96471200	1.20592900
C	-6.52583500	-2.99513600	1.45365900
C	-8.09521500	-1.96142500	-0.03287100
H	-4.66261900	-1.97357800	1.69679900
H	-6.24367100	-3.79182500	2.13480800
H	-9.06811500	-1.96854200	-0.51616300
H	-8.48302800	-3.80065200	1.01726700
C	-5.32466000	1.18254300	-0.76626900
C	-5.84809700	3.34531900	-2.48141700
C	-4.36791900	2.19570300	-0.96461000
C	-6.56107500	1.25214700	-1.43314700
C	-6.78987900	2.34124000	-2.28039500
C	-4.62858300	3.26389400	-1.81281200
H	-3.41497700	2.14658100	-0.45122700
H	-7.74038800	2.40670700	-2.80235900
H	-3.87280000	4.03143600	-1.94709900
H	-6.06440100	4.17407600	-3.14694800
C	-7.65062300	0.19401000	-1.26961100
C	-7.95393000	-0.43369000	-2.64745800
H	-7.06000400	-0.91650000	-3.05320200
H	-8.28714000	0.32893400	-3.35707900
H	-8.74553800	-1.18403800	-2.56685500
C	-8.92856100	0.86704900	-0.72336000
H	-9.27616800	1.65045400	-1.40276400
H	-8.73544300	1.31801900	0.25444600
H	-9.73595400	0.13745000	-0.61412100

DMAC-BP-T_1 Geometry (Solvent: DCM)

H	2.20786000	0.12612300	3.64274500
C	2.37183300	0.24825900	2.57710500
C	2.77824900	0.62447000	-0.14946700
C	1.28609400	0.65553400	1.77805400
C	3.62355100	0.00483300	2.02425400
C	3.82948000	0.18709000	0.65598100
C	1.52286500	0.85614600	0.40466800
H	4.45243000	-0.32557800	2.64490400
H	0.72816100	1.23308200	-0.23143900
H	2.95696900	0.79340000	-1.20810600
C	-0.01146400	0.94178000	2.44634500
O	0.01536900	1.36229800	3.64941500
C	-1.27176400	0.73383600	1.77048200
C	-3.82909700	0.33390900	0.63640400
C	-1.44924100	-0.02524000	0.57356900
C	-2.45698200	1.24566500	2.38590600
C	-3.70640000	1.05935100	1.82869600
C	-2.69947500	-0.21966200	0.01744000
H	-0.59826100	-0.50268500	0.10108800
H	-2.34951300	1.79873500	3.31203100
H	-4.59456600	1.46864300	2.30367200
H	-2.81690300	-0.81296800	-0.88565900
N	5.11868200	-0.06013000	0.08267000
C	6.05432900	0.97981000	0.02849100
C	7.89221200	3.10684100	-0.05211000
C	5.71638800	2.24895000	0.53702400
C	7.33019200	0.77315600	-0.52870800
C	8.22093100	1.85146700	-0.55351800
C	6.62529200	3.29768600	0.49635300
H	4.73499900	2.40930600	0.96709700
H	9.20893200	1.70365200	-0.98070200
H	6.33710200	4.26473200	0.89683200
H	8.61179500	3.91777200	-0.08943300
C	5.41239100	-1.33671100	-0.40979800
C	5.94340100	-3.92086900	-1.38498100

C	6.66662600	-1.61746400	-0.98352900
C	4.44240000	-2.35492500	-0.33041800
C	4.70657500	-3.62993000	-0.81242600
C	6.89862600	-2.91208000	-1.45968400
H	3.47760000	-2.14089900	0.11368600
H	3.93984500	-4.39488600	-0.73688000
H	7.86311400	-3.14012800	-1.90495500
H	6.16254600	-4.91282400	-1.76552400
C	7.77368400	-0.57156800	-1.10196200
C	9.01689100	-1.06427800	-0.32965100
H	9.83636700	-0.34443800	-0.41146200
H	9.36969400	-2.01947500	-0.72917500
H	8.77989100	-1.19920400	0.72980000
C	8.13930400	-0.38940800	-2.59114000
H	8.94528700	0.34086400	-2.70725200
H	7.27149000	-0.03916200	-3.15783700
H	8.47843600	-1.33360300	-3.02692100
N	-5.13558300	0.12558100	0.05468400
C	-5.90564100	-0.93803400	0.50328100
C	-7.40734600	-3.08240300	1.45094500
C	-7.19308200	-1.16980000	-0.03840100
C	-5.37963100	-1.78890800	1.50724900
C	-6.12842400	-2.84854200	1.97320300
C	-7.91929100	-2.25086400	0.46037500
H	-4.38934000	-1.60141500	1.90168500
H	-5.72246200	-3.49678800	2.74133400
H	-8.90975200	-2.45365400	0.06714000
H	-8.00115800	-3.91411100	1.81439100
C	-5.57526000	0.99427900	-0.93358800
C	-6.39490800	2.76362000	-2.92023500
C	-4.73342000	2.06399400	-1.32777500
C	-6.84530500	0.81529100	-1.53318900
C	-7.22353600	1.71798800	-2.52677700
C	-5.14425600	2.93646100	-2.31286700
H	-3.77108700	2.18968600	-0.84856100
H	-8.18968000	1.60701900	-3.00742900
H	-4.49738000	3.75354200	-2.61131000
H	-6.72312300	3.44651300	-3.69634200
C	-7.78704100	-0.29967000	-1.12696100
C	-8.08611700	-1.18143600	-2.36495100
H	-7.16780200	-1.63444100	-2.74744600
H	-8.53746800	-0.58126500	-3.15814900
H	-8.78723200	-1.97729500	-2.10378800
C	-9.10577400	0.32530600	-0.60689100
H	-9.56821900	0.93782800	-1.38428800
H	-8.91690000	0.95290500	0.26787600
H	-9.81347000	-0.45910500	-0.32922200

Cz2BP-S_0 Geometry (Solvent: Toluene)

H	-0.61977500	0.14075200	-1.08635800
C	-1.45502600	0.61055300	-0.57636600
C	-3.62852600	1.83638900	0.67579500
C	-1.29445900	1.85934000	0.03415200
C	-2.69704500	-0.01548900	-0.58052200
C	-3.78275900	0.59039500	0.05727200
C	-2.39532000	2.47231500	0.64390500
H	-2.83692600	-0.96408200	-1.08896500
H	-2.26327900	3.44548400	1.10569300
H	-4.47406400	2.28917900	1.18382900
C	-0.00000200	2.60913100	-0.00004800
O	-0.00000200	3.82959600	-0.00001500
C	1.29445800	1.85934200	-0.03422300
C	3.78275800	0.59039700	-0.05729600
C	1.45501200	0.61055300	0.57629300
C	2.39533100	2.47232000	-0.64395000

C	3.62853800	1.83639400	-0.67581800
C	2.69703100	-0.01548900	0.58047300
H	0.61974900	0.14075000	1.08626500
H	2.26329900	3.44549100	-1.10573700
H	4.47408700	2.28918600	-1.18383200
H	2.83690200	-0.96408400	1.08891500
N	-5.03897700	-0.05414700	0.07398400
C	-5.27527100	-1.36709500	0.48172100
C	-6.25965000	-3.85067200	1.21808900
C	-4.37994600	-2.31249000	0.98870400
C	-6.65487000	-1.64337000	0.35976400
C	-7.14586100	-2.89877200	0.72942300
C	-4.89271900	-3.55339400	1.34964600
H	-3.32514200	-2.08865700	1.10607000
H	-8.20478200	-3.12257400	0.64001400
H	-4.21942600	-4.30707000	1.74553100
H	-6.62418300	-4.83037700	1.50828000
C	-6.25277400	0.51719400	-0.30953700
C	-8.86107300	1.16216700	-0.98605900
C	-7.27984200	-0.43839200	-0.14820000
C	-6.50862500	1.78977600	-0.82691700
C	-7.82422300	2.09499200	-1.15707000
C	-8.59471500	-0.10708800	-0.48792700
H	-5.71377500	2.51277400	-0.97530600
H	-8.05176600	3.07690900	-1.55965800
H	-9.39292400	-0.83368800	-0.36815600
H	-9.87623100	1.43615600	-1.25313600
N	5.03897700	-0.05414400	-0.07398400
C	6.25276500	0.51719700	0.30956400
C	8.86104800	1.16216900	0.98614600
C	7.27983800	-0.43838700	0.14824500
C	6.50860300	1.78977700	0.82695600
C	7.82419400	2.09499300	1.15713800
C	8.59470200	-0.10708300	0.48800200
H	5.71374800	2.51277300	0.97533100
H	8.05172700	3.07690800	1.55973600
H	9.39291500	-0.83368200	0.36824500
H	9.87620000	1.43615800	1.25324600
C	5.27528100	-1.36708800	-0.48172500
C	6.25967800	-3.85066100	-1.21808400
C	4.37996800	-2.31248300	-0.98873200
C	6.65487700	-1.64336300	-0.35973900
C	7.14587700	-2.89876300	-0.72939400
C	4.89274900	-3.55338400	-1.34967000
H	3.32516500	-2.08865000	-1.10612000
H	8.20479600	-3.12256500	-0.63996300
H	4.21946600	-4.30705800	-1.74557300
H	6.62421800	-4.83036400	-1.50827300

Cz2BP-T_1 Geometry (Solvent: Toluene)

H	-0.67668900	-0.23354000	-0.80457200
C	-1.49401200	0.34369900	-0.38918300
C	-3.68090400	1.86645000	0.46554800
C	-1.27724100	1.68399100	0.03865200
C	-2.75241000	-0.22240100	-0.37192500
C	-3.86308700	0.52559300	0.06713300
C	-2.42404100	2.42831000	0.44055900
H	-2.90534100	-1.22886000	-0.75060700
H	-2.26842900	3.45607100	0.74770300
H	-4.53210200	2.43553100	0.82792300
C	0.00000000	2.39733400	-0.00000700
O	0.00000000	3.66647100	0.00000000
C	1.27724100	1.68399200	-0.03866400
C	3.86308600	0.52559400	-0.06713700
C	1.49401000	0.34369800	0.38917000
C	2.42404200	2.42831100	-0.44056500

C	3.68090600	1.86645200	-0.46555000
C	2.75240800	-0.22240200	0.37191600
H	0.67668600	-0.23354100	0.80455500
H	2.26843200	3.45607300	-0.74770700
H	4.53210500	2.43553300	-0.82792000
H	2.90533800	-1.22886100	0.75059600
N	-5.14198000	-0.05612300	0.09115300
C	-5.45911000	-1.30198600	0.63065500
C	-6.57971900	-3.63725200	1.59566200
C	-4.63083000	-2.20360100	1.30474300
C	-6.84304600	-1.53839500	0.46624800
C	-7.40489500	-2.71574700	0.94958900
C	-5.21339600	-3.37674500	1.77502400
H	-3.58030600	-1.99261000	1.46945900
H	-8.46667200	-2.91054200	0.83442100
H	-4.59689400	-4.09893800	2.29975400
H	-7.00184300	-4.56088400	1.97706100
C	-6.30759200	0.51602400	-0.41938900
C	-8.83435800	1.18849600	-1.31494600
C	-7.38836300	-0.36927500	-0.20555500
C	-6.46506600	1.72121400	-1.10927700
C	-7.74701300	2.04425200	-1.54398100
C	-8.66054400	-0.02795000	-0.65396200
H	-5.62289500	2.37298900	-1.31216800
H	-7.90336600	2.97502800	-2.07887600
H	-9.50046100	-0.69846900	-0.50032300
H	-9.82004700	1.47107900	-1.66896600
N	5.14198000	-0.05612200	-0.09115400
C	6.30759000	0.51602400	0.41939400
C	8.83435300	1.18849500	1.31496100
C	7.38836200	-0.36927400	0.20556200
C	6.46506200	1.72121300	1.10928400
C	7.74700700	2.04425100	1.54399400
C	8.66054100	-0.02795000	0.65397400
H	5.62289000	2.37298800	1.31217300
H	7.90335800	2.97502600	2.07889000
H	9.50045900	-0.69846900	0.50033800
H	9.82004100	1.47107800	1.66898600
C	5.45911200	-1.30198500	-0.63065600
C	6.57972500	-3.63724900	-1.59566200
C	4.63083400	-2.20359800	-1.30474900
C	6.84304800	-1.53839300	-0.46624400
C	7.40489800	-2.71574400	-0.94958600
C	5.21340300	-3.37674100	-1.77502900
H	3.58031100	-1.99260700	-1.46946800
H	8.46667500	-2.91053900	-0.83441400
H	4.59690200	-4.09893400	-2.29976200
H	7.00185100	-4.56088000	-1.97706200

Cz2BP-S_1 Geometry (Solvent: Toluene)

H	-0.67025600	-0.06722400	-0.96213200
C	-1.49787700	0.45876700	-0.49858500
C	-3.67335300	1.84773300	0.57217200
C	-1.30807800	1.74982000	0.03122800
C	-2.75342400	-0.12949000	-0.47602800
C	-3.84674900	0.55713500	0.06555100
C	-2.42041900	2.44433900	0.54679200
H	-2.90260700	-1.11618700	-0.90404000
H	-2.28720200	3.43908900	0.95876300
H	-4.52171400	2.37153000	1.00197400
C	0.00002400	2.40356400	0.00041100
O	0.00003200	3.69953500	0.00043300
C	1.30811100	1.74980500	-0.03061900
C	3.84673800	0.55704200	-0.06542900
C	1.49797200	0.45874900	0.49916600
C	2.42037100	2.44428600	-0.54640400

C	3.67327600	1.84763000	-0.57205100
C	2.75350000	-0.12954000	0.47638200
H	0.67042600	-0.06721100	0.96288300
H	2.28710300	3.43903900	-0.95835300
H	4.52156900	2.37140000	-1.00202000
H	2.90274200	-1.11622500	0.90440300
N	-5.12160500	-0.05296300	0.08886800
C	-5.41773300	-1.29664300	0.64319400
C	-6.50620700	-3.64988400	1.61912800
C	-4.57565400	-2.19056600	1.30986400
C	-6.79612800	-1.55767900	0.47706200
C	-7.33951600	-2.74790700	0.96878700
C	-5.14022200	-3.36690800	1.78973400
H	-3.52263600	-1.97426000	1.45568300
H	-8.39762200	-2.96035400	0.84730100
H	-4.50972300	-4.08022400	2.31106600
H	-6.91195900	-4.57881700	2.00569100
C	-6.29417900	0.49149400	-0.43096300
C	-8.84184300	1.11143200	-1.32448600
C	-7.35761500	-0.41213100	-0.21071500
C	-6.48320000	1.69794800	-1.11039500
C	-7.76945000	1.99180300	-1.54847500
C	-8.64169600	-0.09313500	-0.66128100
H	-5.65855900	2.37774500	-1.29688100
H	-7.94611000	2.92245100	-2.07836200
H	-9.46773600	-0.77910400	-0.49809200
H	-9.83266400	1.37485100	-1.67927500
N	5.12159400	-0.05304800	-0.08890300
C	6.29427100	0.49150500	0.43059800
C	8.84211700	1.11162400	1.32347400
C	7.35767100	-0.41214400	0.21027300
C	6.48342300	1.69807700	1.10978500
C	7.76976200	1.99202200	1.54754000
C	8.64184300	-0.09305800	0.66051600
H	5.65881400	2.37789600	1.29632900
H	7.94652500	2.92276300	2.07722900
H	9.46785500	-0.77904500	0.49726800
H	9.83301100	1.37511500	1.67800700
C	5.41762200	-1.29681200	-0.64309500
C	6.50591300	-3.65021500	-1.61884400
C	4.57541300	-2.19085500	-1.30944200
C	6.79605200	-1.55781100	-0.47719700
C	7.33934800	-2.74811900	-0.96883000
C	5.13989100	-3.36727800	-1.78922200
H	3.52236500	-1.97458200	-1.45508500
H	8.39748000	-2.96053800	-0.84752400
H	4.50929000	-4.08068900	-2.31030100
H	6.91159300	-4.57921300	-2.00532800

CC2BP-S_0 Geometry (Solvent: Toluene)

H	0.71688400	-2.60164900	0.20674800
C	1.52915900	-2.31363800	-0.45337500
C	3.63103500	-1.61041000	-2.15195300
C	1.26087600	-1.91155900	-1.76614400
C	2.83893400	-2.36199200	0.01276600
C	3.88987900	-2.00533200	-0.83531300
C	2.32188400	-1.57007400	-2.61235400
H	3.05297300	-2.66336000	1.03326000
H	2.10526900	-1.28676000	-3.63730300
H	4.45888200	-1.35879300	-2.80746500
C	-0.12511700	-1.90688400	-2.33265600
O	-0.30280000	-2.13731200	-3.51725700
C	-1.29144500	-1.61833500	-1.44034500
C	-3.55148400	-1.04519700	0.09480500
C	-2.53044000	-2.17914100	-1.77065800
C	-1.19670400	-0.75871000	-0.34046600

C	-2.32519300	-0.45712400	0.41454000
C	-3.65207700	-1.91469600	-0.99694400
H	-2.59552300	-2.83686700	-2.63141700
H	-0.24934700	-0.28940100	-0.09357000
H	-2.26691400	0.24353500	1.24126500
H	-4.60516100	-2.38220200	-1.22307600
N	5.22164100	-2.04914700	-0.36178600
C	5.83489400	-3.14515000	0.24521100
C	7.48901100	-5.01344000	1.44823300
C	5.33055500	-4.42867000	0.47073800
C	7.15649500	-2.79486900	0.59713400
C	7.98740600	-3.74001800	1.20627200
C	6.17544600	-5.35031200	1.07776300
H	4.32306000	-4.70212600	0.17605900
H	9.00620200	-3.48212800	1.47987000
H	5.81089200	-6.35528400	1.26503700
H	8.11890400	-5.75957100	1.92067800
C	6.13266000	-0.99746400	-0.40301100
C	8.25685000	0.76255700	-0.20377600
C	7.34611500	-1.41905000	0.18536100
C	5.96244100	0.30545600	-0.87886000
C	7.03536100	1.17875200	-0.76386300
C	8.42191100	-0.53369100	0.27114500
H	5.02219700	0.63660800	-1.30620900
H	6.94025900	2.20598400	-1.10232100
H	9.37275700	-0.83722900	0.69994900
N	-4.69450800	-0.75689000	0.87425000
C	-5.92475000	-0.32518000	0.38387200
C	-8.50210700	0.56996600	-0.07222100
C	-6.81006900	-0.13770400	1.46872800
C	-6.30832500	-0.04618400	-0.93085700
C	-7.60262700	0.40911800	-1.14160500
C	-8.11505700	0.29964300	1.23533300
H	-5.62014000	-0.15806000	-1.76143300
H	-7.93662400	0.65495300	-2.14498000
H	-8.82295100	0.43062000	2.04889200
C	-4.77854100	-0.85029600	2.26407700
C	-5.46110600	-0.91085300	4.94935800
C	-3.81652500	-1.27638600	3.18377600
C	-6.07713200	-0.47541800	2.67160600
C	-6.41681800	-0.50438000	4.02738700
C	-4.17786200	-1.29706800	4.52577900
H	-2.82723100	-1.58824100	2.86703600
H	-7.41287900	-0.21743400	4.35105400
H	-3.45010900	-1.62271200	5.26220100
H	-5.70617800	-0.93815900	6.00570400
N	9.32945300	1.68900500	-0.11105000
C	9.93680000	2.33645500	-1.18035300
C	11.45812300	3.83176100	-2.94651100
C	10.95951700	3.17984400	-0.68930200
C	9.67003000	2.22037200	-2.54728300
C	10.44318500	2.98009700	-3.41736100
C	11.72294300	3.93337500	-1.58629400
H	8.89210000	1.55876700	-2.91441800
H	10.25936200	2.91106500	-4.48496900
H	12.51230200	4.58544700	-1.22376300
H	12.04096600	4.41073700	-3.65517100
C	9.94541700	2.10102900	1.06515200
C	11.45382600	3.23137500	3.09467500
C	9.65927600	1.73848900	2.38424900
C	10.96601300	3.02765600	0.75170300
C	11.72516500	3.59322400	1.78092300
C	10.42795900	2.31566300	3.38819400
H	8.86470800	1.03642400	2.61522000
H	12.51271900	4.30631200	1.55510500
H	10.22886200	2.05329500	4.42255900
H	12.03487700	3.66023900	3.90436200

N	-9.82052300	1.02691600	-0.33790700
C	-10.75640300	0.37828400	-1.13437300
C	-12.93659300	-0.52476700	-2.58324100
C	-11.94769100	1.13875900	-1.16046300
C	-10.64194700	-0.83680800	-1.81525400
C	-11.74657700	-1.27231500	-2.53773700
C	-13.04441100	0.67782900	-1.89539000
H	-9.72698900	-1.41913000	-1.77669100
H	-11.68792700	-2.21255400	-3.07706400
H	-13.96651100	1.25104100	-1.92479800
H	-13.77835400	-0.89521100	-3.15887700
C	-10.38971500	2.19599100	0.15335000
C	-11.93692400	4.38634100	0.84475800
C	-9.82304600	3.17462900	0.97440500
C	-11.71279700	2.30493200	-0.33281600
C	-12.48916300	3.41274200	0.02147300
C	-10.61591100	4.26545800	1.31109500
H	-8.80189800	3.08733000	1.33118600
H	-13.50718100	3.51031900	-0.34443900
H	-10.20316100	5.04212400	1.94733500
H	-12.52680000	5.25127700	1.12946800

CC2BP-T_1 Geometry (Solvent: Toluene)

H	0.64471400	-1.71440900	-0.86254000
C	1.48884000	-1.13137600	-1.21061900
C	3.68411900	0.37831100	-2.17444500
C	1.25569300	0.10935600	-1.92985000
C	2.74984700	-1.57969100	-0.97569700
C	3.89561800	-0.82630800	-1.42259200
C	2.42614300	0.81150200	-2.44448400
H	2.90879300	-2.48311200	-0.39525900
H	2.25166700	1.68721800	-3.05727500
H	4.54471900	0.89688000	-2.58650300
C	-0.03893300	0.64447600	-2.24185000
O	-0.14271200	1.56924900	-3.09264500
C	-1.28449900	0.10995900	-1.61242200
C	-3.71083200	-0.74778400	-0.50234600
C	-2.44526200	0.04406800	-2.39466600
C	-1.36655000	-0.23887600	-0.25787900
C	-2.57207800	-0.65454300	0.29940100
C	-3.64539400	-0.40066700	-1.85430000
H	-2.38695600	0.34168000	-3.43663200
H	-0.49374200	-0.14118400	0.38142400
H	-2.64288300	-0.88861800	1.35747000
H	-4.53534600	-0.48448800	-2.47108000
N	5.17436200	-1.26532200	-1.13268300
C	5.63207700	-2.59306900	-1.21965200
C	7.00019900	-4.99081600	-1.28912100
C	4.96229200	-3.71840600	-1.69882400
C	6.98565900	-2.64056800	-0.82913700
C	7.67503400	-3.84814900	-0.85920100
C	5.66632800	-4.92079400	-1.71136800
H	3.94604900	-3.66394200	-2.07147600
H	8.71947200	-3.89828100	-0.56779700
H	5.17229000	-5.81682700	-2.07151300
H	7.52013600	-5.94226200	-1.31827600
C	6.23950700	-0.46529200	-0.69934000
C	8.58568700	0.65375600	0.23638200
C	7.37575700	-1.27699900	-0.49810400
C	6.24642400	0.89676300	-0.39272100
C	7.43532000	1.44471700	0.07197500
C	8.56037900	-0.71696600	-0.04037800
H	5.35394800	1.50616100	-0.47535400
H	7.47863000	2.49451400	0.34301800
H	9.45403900	-1.31694100	0.10155000
N	-4.93924600	-1.18137200	0.05962000

C	-6.13694500	-0.47820600	0.02772400
C	-8.70488500	0.52198800	0.25406800
C	-7.13192200	-1.23126000	0.69258800
C	-6.40815600	0.78305700	-0.51110800
C	-7.69949900	1.27428000	-0.38118600
C	-8.43153200	-0.73024200	0.79221500
H	-5.63590100	1.36877000	-0.99802100
H	-7.95017300	2.25736500	-0.76798300
H	-9.22197600	-1.29586300	1.27755100
C	-5.14636900	-2.37877400	0.73757600
C	-6.06053400	-4.60564100	2.10985400
C	-4.24550900	-3.41672700	0.99229900
C	-6.49655000	-2.45035300	1.14789100
C	-6.95257400	-3.57549900	1.84192500
C	-4.72299700	-4.52369300	1.68329800
H	-3.21481000	-3.36284400	0.65815700
H	-7.98808600	-3.64222900	2.16251900
H	-4.04616000	-5.34532000	1.89566600
H	-6.39669000	-5.48524600	2.64854200
N	9.77458300	1.24746500	0.71163300
C	10.39846200	2.37087000	0.16834100
C	12.01739300	4.49946000	-0.55144900
C	11.56577600	2.65087900	0.91210700
C	10.03698300	3.13088400	-0.94672800
C	10.86100900	4.19691300	-1.28985800
C	12.37796800	3.72781400	0.54645500
H	9.15269400	2.89772300	-1.53022300
H	10.60420800	4.80582900	-2.15078800
H	13.27827900	3.95328300	1.11028500
H	12.63573600	5.34013300	-0.84771700
C	10.52786200	0.80295200	1.79859900
C	12.31719300	0.35441300	3.86500800
C	10.27607000	-0.25462200	2.67618100
C	11.64819200	1.64808600	1.95605100
C	12.54973700	1.41563300	2.99843000
C	11.18758300	-0.46538800	3.70477600
H	9.39850400	-0.88352200	2.57086300
H	13.41477200	2.05882300	3.13033900
H	11.01718700	-1.27999500	4.40148800
H	13.00792300	0.15980600	4.67857900
N	-10.01646200	1.06079700	0.35252000
C	-10.83401500	1.40148500	-0.71774500
C	-12.81140300	2.20106200	-2.48429800
C	-12.05956800	1.90327500	-0.22261200
C	-10.58570500	1.28296600	-2.08805800
C	-11.58971300	1.69073400	-2.95828600
C	-13.05326300	2.30655800	-1.12011000
H	-9.64609800	0.88421900	-2.45660000
H	-11.42583000	1.61100900	-4.02842100
H	-13.99987600	2.69415400	-0.75465100
H	-13.57177800	2.51106100	-3.19349800
C	-10.69559700	1.33337400	1.53364300
C	-12.42541600	2.05402900	3.57342600
C	-10.26636500	1.17343800	2.85416000
C	-11.97136500	1.85794200	1.22292700
C	-12.84043700	2.21811300	2.25758400
C	-11.14910400	1.53963000	3.86330100
H	-9.27991300	0.78208400	3.08086600
H	-13.82356400	2.62226300	2.03414100
H	-10.84341400	1.42714600	4.89884400
H	-13.08860600	2.32781300	4.38732000

A-BP-TA-S_0 Geometry (Solvent: THF)

H	2.09922900	-1.17642800	-3.18748500
C	2.39359500	-1.00728100	-2.15676400
C	3.12077300	-0.61701100	0.50979300

C	1.45879500	-1.22561600	-1.13794600
C	3.67776500	-0.57527400	-1.84698800
C	4.03847600	-0.38236200	-0.51253400
C	1.82816600	-1.03051900	0.19768200
H	4.40956900	-0.38763600	-2.62689600
H	1.11778400	-1.22215800	0.99571400
H	3.42719800	-0.47404000	1.54153300
C	0.10280300	-1.73494100	-1.52162800
O	-0.02506200	-2.44033600	-2.51047200
C	-1.08921900	-1.37793900	-0.69194300
C	-3.41301800	-0.74559500	0.74859000
C	-1.17862200	-0.16842300	0.00517700
C	-2.17068200	-2.26640400	-0.66984500
C	-3.31567100	-1.95859000	0.05260700
C	-2.33462900	0.14517500	0.71452400
H	-0.35969000	0.54351600	-0.02518800
H	-2.09603500	-3.20133500	-1.21587300
H	-4.13654600	-2.66947600	0.09150500
H	-2.40839000	1.09493000	1.23587500
C	-4.62305200	-0.41452700	1.54773600
C	-6.84323000	0.20146700	3.15416800
C	-5.91744100	-0.41357900	0.99223000
C	-4.46915100	-0.11270500	2.90570500
C	-5.56618400	0.19226600	3.70563600
C	-7.02066600	-0.11542200	1.80610000
H	-3.47137700	-0.13120700	3.33387800
H	-5.42717900	0.42436800	4.75609600
H	-7.70799200	0.44876400	3.76150100
S	-8.67214300	-0.18118500	1.17339600
S	-6.16088700	-0.77619200	-0.72749500
C	-7.36749500	0.43881400	-1.16664000
C	-9.32106300	2.25970000	-1.97806100
C	-7.23335100	1.11322700	-2.38309800
C	-8.47208200	0.69394200	-0.34695400
C	-9.43837300	1.62030800	-0.74619800
C	-8.21892200	2.00683200	-2.79569000
H	-6.36200800	0.92666100	-3.00315900
H	-10.28015500	1.82856700	-0.09310800
H	-8.11775600	2.51474000	-3.74901600
H	-10.08318700	2.96552000	-2.29122600
N	5.36048900	0.05656500	-0.19137400
C	6.35726500	-0.90006900	0.04536500
C	8.31703900	-2.85739800	0.51366300
C	6.04603500	-2.26979000	-0.04332000
C	7.66672400	-0.50277700	0.37044800
C	8.61858000	-1.50196400	0.59808200
C	7.01619300	-3.23572400	0.18867100
H	5.03900000	-2.57954600	-0.29618500
H	9.63381300	-1.20904500	0.85065800
H	6.74865700	-4.28504000	0.11333100
H	9.08428600	-3.60175800	0.69756200
C	5.62210700	1.43140500	-0.11921700
C	6.09390200	4.19790600	0.01135600
C	6.91004900	1.89855900	0.19899000
C	4.58797100	2.35352400	-0.36703400
C	4.82328900	3.72051900	-0.30226800
C	7.11151600	3.28144100	0.25602400
H	3.59455800	1.99736300	-0.61262200
H	4.00754200	4.40919900	-0.49885700
H	8.10162900	3.65567100	0.50067900
H	6.29136600	5.26311500	0.06515000
C	8.08382500	0.96258100	0.48145000
C	9.20868100	1.24438900	-0.53832200
H	10.07319300	0.60116900	-0.35130400
H	9.54405000	2.28318900	-0.47023800
H	8.85573900	1.06116900	-1.55743100
C	8.60959500	1.23039200	1.90842300

H	9.46559400	0.58816500	2.13438800
H	7.82682900	1.03565800	2.64743400
H	8.93406600	2.26946800	2.01445100

A-BP-TA-T_1 Geometry (Solvent: THF)

H	2.00693600	-2.69205400	-1.75813800
C	2.36239700	-1.96635800	-1.03343300
C	3.25976700	-0.14411300	0.87330500
C	1.47145400	-1.49460800	-0.05956300
C	3.67783600	-1.51602800	-1.07232000
C	4.12580800	-0.60288200	-0.11741200
C	1.93888800	-0.58560900	0.89927600
H	4.36803900	-1.86814100	-1.83386300
H	1.27440100	-0.24078600	1.68583700
H	3.63053700	0.55022600	1.62199800
C	0.08036900	-2.05924900	-0.03172200
O	-0.05704800	-3.27937900	-0.28374700
C	-1.04988800	-1.21003000	0.27060500
C	-3.40737100	0.35175800	0.77704700
C	-1.01403300	0.21638100	0.27883300
C	-2.31196100	-1.82041900	0.52788500
C	-3.43928100	-1.07620200	0.77580100
C	-2.14108700	0.96146800	0.53047200
H	-0.09194700	0.73499300	0.03667600
H	-2.35626900	-2.90413500	0.55978100
H	-4.35728900	-1.59214100	1.04325800
H	-2.07618700	2.04514500	0.48282800
C	-4.56586800	1.15536100	1.08924800
C	-6.85195100	2.62803200	1.98795200
C	-5.90782400	0.81237900	0.65058000
C	-4.44560500	2.33409000	1.84557400
C	-5.55308800	3.04888800	2.28509800
C	-7.03119600	1.52203400	1.15805300
H	-3.45563500	2.64684500	2.16063300
H	-5.40951500	3.92649500	2.90655300
H	-7.71379200	3.15646600	2.38080000
S	-8.69126900	1.12635700	0.87048100
S	-5.96875100	-0.28174900	-0.67023500
C	-7.61940000	-0.59085800	-1.04744600
C	-10.19843900	-1.36495300	-1.80534200
C	-7.80894400	-1.50321800	-2.10932300
C	-8.74033800	-0.05756400	-0.38311100
C	-10.03218700	-0.46682700	-0.77134200
C	-9.07932800	-1.88280400	-2.48446600
H	-6.93868200	-1.90830900	-2.61680200
H	-10.89400000	-0.05864700	-0.25232900
H	-9.21285800	-2.58573500	-3.29881300
H	-11.19830500	-1.66779700	-2.09576100
N	5.48147900	-0.14362000	-0.14820700
C	6.45732100	-0.84912300	0.56720500
C	8.37394600	-2.30202500	2.02240700
C	6.09468000	-1.99817800	1.29554500
C	7.79786500	-0.42188100	0.56604300
C	8.72671400	-1.16636300	1.30048600
C	7.04301100	-2.71441800	2.01353500
H	5.06355600	-2.33068900	1.29689700
H	9.76469200	-0.84547300	1.30818200
H	6.73465200	-3.59683100	2.56556800
H	9.12453900	-2.85246000	2.57953000
C	5.79716000	0.99916800	-0.89283600
C	6.37375900	3.29915900	-2.40337600
C	7.11732100	1.48379700	-0.94038900
C	4.78415400	1.67215600	-1.60251100
C	5.07106900	2.80782600	-2.34803800
C	7.37068600	2.63027000	-1.70038500
H	3.76705600	1.30031500	-1.56867800

H	4.27000900	3.30575700	-2.88556900
H	8.38597900	3.01451600	-1.74358400
H	6.61140500	4.18544000	-2.98195700
C	8.27160700	0.81308700	-0.19800100
C	9.35103400	0.39167200	-1.21864000
H	10.20216400	-0.07509300	-0.71469000
H	9.72449800	1.25887500	-1.77068000
H	8.93963300	-0.32404500	-1.93644200
C	8.88095700	1.82053700	0.80120700
H	9.72334600	1.37442000	1.33764700
H	8.13127900	2.13305400	1.53412600
H	9.24803000	2.71031300	0.28150700

A-BP-TA-S_1 Geometry (Solvent: THF)

H	2.26134400	0.83832600	-3.37956200
C	2.50375100	0.50543900	-2.37570700
C	3.07482200	-0.40688200	0.21744100
C	1.42886000	0.03373100	-1.54712600
C	3.79888400	0.55290200	-1.92753300
C	4.10157200	0.10795000	-0.61542400
C	1.77938900	-0.43920000	-0.24124000
H	4.59842400	0.92668500	-2.56185200
H	1.01793000	-0.87060400	0.39992900
H	3.32512600	-0.78421700	1.20505400
C	0.10734200	-0.00758000	-2.10606800
O	-0.11362700	0.19789400	-3.32931900
C	-1.07971600	-0.31030000	-1.23683500
C	-3.42076800	-0.83427200	0.25761200
C	-1.25044800	0.23151800	0.04379500
C	-2.10914000	-1.10151500	-1.76478500
C	-3.25526100	-1.37102700	-1.02634600
C	-2.40319200	-0.02802800	0.78077000
H	-0.48897300	0.88630100	0.45733000
H	-1.99231500	-1.50567500	-2.76541900
H	-4.02488100	-2.01652600	-1.44265600
H	-2.52797900	0.41808600	1.76384200
C	-4.62931900	-1.13146100	1.07051600
C	-6.84855900	-1.75336300	2.68528200
C	-5.93745200	-0.91171800	0.59390900
C	-4.46514100	-1.66671900	2.35429700
C	-5.55869300	-1.97414300	3.15775300
C	-7.03739600	-1.24008400	1.40099200
H	-3.45712400	-1.85115200	2.71378800
H	-5.40768300	-2.38607100	4.14999400
H	-7.71272500	-1.97940200	3.30167200
S	-8.69675200	-1.06704000	0.80851900
S	-6.20165200	-0.20874300	-1.01440600
C	-7.52290100	0.91841900	-0.68632600
C	-9.65202200	2.68907900	-0.32846800
C	-7.48546300	2.18679000	-1.27210700
C	-8.62065800	0.54691800	0.09737200
C	-9.67565100	1.44185100	0.29115900
C	-8.55700000	3.06158100	-1.10910300
H	-6.61984400	2.47675000	-1.85962700
H	-10.51229700	1.15242400	0.91947800
H	-8.52888700	4.03832900	-1.58056200
H	-10.48192500	3.37388700	-0.18886700
N	5.44400800	0.14690800	-0.14235900
C	6.25862400	-0.96531400	-0.32626100
C	7.82349400	-3.23139500	-0.72806300
C	5.71965700	-2.09627200	-0.98549900
C	7.59669100	-0.96607500	0.13527000
C	8.35184000	-2.11588500	-0.08105100
C	6.50138100	-3.21778000	-1.18184400
H	4.69511000	-2.07128600	-1.33230900

H	9.38096600	-2.14974300	0.26092600
H	6.08457700	-4.08114000	-1.68751300
H	8.44367600	-4.10829900	-0.87821400
C	5.90497100	1.29622700	0.49138200
C	6.75319300	3.63280700	1.74277900
C	7.23297700	1.37264400	0.97454000
C	5.01604300	2.38741200	0.64179900
C	5.44290100	3.54428300	1.26382500
C	7.62549900	2.55617600	1.59476500
H	4.00489900	2.30396100	0.26590700
H	4.75884900	4.37746100	1.37660600
H	8.63730000	2.64809900	1.97548900
H	7.09615700	4.53804600	2.23174500
C	8.21545000	0.22513400	0.84104300
C	9.44119000	0.70744700	0.02808100
H	10.17635000	-0.09517800	-0.06431600
H	9.92267100	1.55016800	0.52945700
H	9.13949800	1.02243600	-0.97425300
C	8.66910100	-0.21170200	2.25520800
H	9.39893400	-1.02181100	2.18722900
H	7.81581200	-0.55812300	2.84417600
H	9.13934100	0.62374200	2.77895000

OPDPO-S_0 Geometry (Solvent: THF)

H	2.24983900	-3.17102800	-2.53066800
C	2.44812600	-2.23607600	-2.01665900
C	2.92521900	0.20319600	-0.74427500
C	1.39561300	-1.33727000	-1.80798000
C	3.72701900	-1.92871900	-1.56867500
C	3.96953900	-0.70717500	-0.92793500
C	1.63794600	-0.11605200	-1.17125600
H	4.54667800	-2.62348600	-1.72671100
H	0.83358800	0.59976400	-1.03360600
H	3.11144700	1.16584100	-0.27615500
C	0.04528900	-1.68966200	-2.35554400
C	-1.18919800	-1.19062300	-1.67086100
C	-3.56128600	-0.36242800	-0.46800100
C	-2.33325300	-0.97281500	-2.44973000
C	-1.24428200	-1.00155400	-0.28714900
C	-2.43484700	-0.59654100	0.31473200
C	-3.51254700	-0.54803000	-1.85350000
H	-2.28112300	-1.13525300	-3.52137200
H	-0.36984300	-1.19087300	0.32749900
H	-2.49982600	-0.45803700	1.38898500
H	-4.40117000	-0.36016500	-2.44976000
O	-0.04599300	-2.37853900	-3.35843200
N	-4.76711400	0.07331400	0.17536700
C	-5.79302900	-0.88771000	0.36067500
C	-7.85918300	-2.76425400	0.76811500
C	-5.72036100	-2.16955100	-0.19997200
C	-6.91014500	-0.57282800	1.15337400
C	-7.94232500	-1.49149600	1.32948200
C	-6.73838200	-3.09817800	0.01409600
H	-4.86280700	-2.45528700	-0.79648900
H	-8.80057500	-1.20783000	1.93133700
H	-6.64839900	-4.08639800	-0.42497200
H	-8.65720200	-3.48172100	0.92475900
C	-5.08289900	1.45305900	0.09519700
C	-5.74496800	4.19275400	-0.01956700
C	-6.13552300	1.97565600	0.86615700
C	-4.35536700	2.33510800	-0.71452600
C	-4.67807700	3.69101800	-0.75874700
C	-6.47794800	3.32357400	0.78623100
H	-3.52482200	1.97069600	-1.30650600
H	-4.08920700	4.35083100	-1.38757700

H	-7.30987000	3.69012800	1.38028000
H	-6.00445400	5.24488300	-0.06246500
S	-6.95171200	0.94819300	2.04215900
P	5.66480500	-0.38142000	-0.36578400
C	5.81669500	1.42007700	-0.30266100
C	6.04068000	4.20117900	-0.35818700
C	5.59133300	2.16366300	0.86010100
C	6.16276600	2.07315400	-1.49250000
C	6.27323100	3.46102200	-1.51884200
C	5.70175900	3.55383800	0.82927200
H	5.33612600	1.66298600	1.79004200
H	6.35212100	1.49100500	-2.39017500
H	6.54404200	3.96443300	-2.44126600
H	5.52930500	4.12856600	1.73344500
H	6.12936800	5.28278000	-0.37889400
C	5.74271400	-0.96604300	1.34387100
C	6.01326800	-1.83708300	3.98090400
C	4.62101300	-1.03417400	2.17740400
C	6.99884000	-1.34412400	1.83216500
C	7.13228100	-1.77823600	3.14937500
C	4.75904900	-1.46679900	3.49542900
H	3.63975800	-0.75634800	1.80197000
H	7.86272500	-1.30565400	1.17476400
H	8.10654400	-2.07383400	3.52490900
H	3.88748500	-1.52056800	4.13968000
H	6.11751500	-2.17763600	5.00623800
O	6.68291000	-1.02271700	-1.24566000

OPDPO-T_1 Geometry (Solvent: THF)

H	2.26209200	-1.94597400	-3.48817900
C	2.46425100	-1.27399100	-2.66090200
C	2.96093300	0.51167900	-0.58444900
C	1.37628400	-0.56754100	-2.09732600
C	3.75373900	-1.12236200	-2.17700600
C	4.02178000	-0.23436900	-1.12131600
C	1.66715600	0.34874100	-1.06094400
H	4.57108200	-1.68537900	-2.62063200
H	0.88171300	0.97986800	-0.65874100
H	3.15018600	1.24155800	0.19908400
C	0.04412500	-0.75720300	-2.69762800
C	-1.18142900	-0.50720500	-1.94964900
C	-3.66261600	-0.14251600	-0.66062800
C	-2.39881500	-0.42441800	-2.68310400
C	-1.27867900	-0.43986900	-0.53218500
C	-2.49654700	-0.25749100	0.10187000
C	-3.61641500	-0.23541400	-2.05441600
H	-2.34682800	-0.51069200	-3.76241100
H	-0.39618100	-0.57909500	0.08140300
H	-2.55595400	-0.22344500	1.18660400
H	-4.53518700	-0.15950300	-2.63053800
O	-0.03148000	-1.16429300	-3.90355000
N	-4.93596600	0.04257900	0.00361500
C	-5.63786200	-1.09536100	0.37637300
C	-7.02100200	-3.44339700	1.11516000
C	-5.10098500	-2.37441400	0.09304300
C	-6.88828200	-1.03303300	1.03992900
C	-7.56659200	-2.21103700	1.40232400
C	-5.78025100	-3.51704400	0.45574900
H	-4.14855500	-2.44891900	-0.41468700
H	-8.52331800	-2.13218500	1.90901400
H	-5.34611900	-4.48362500	0.22588000
H	-7.54771500	-4.34821300	1.39595300
C	-5.36863900	1.34387900	0.21643100
C	-6.20157600	4.01162600	0.61592800
C	-6.59553500	1.64129300	0.85977500
C	-4.56865000	2.42746400	-0.21916300

C	-4.97973100	3.72768800	-0.02145500
C	-6.99899600	2.97536000	1.05118100
H	-3.62720200	2.22544100	-0.71258000
H	-4.34791100	4.53864000	-0.36607700
H	-7.94576400	3.17329600	1.54435200
H	-6.51720300	5.03786500	0.76473000
S	-7.67142800	0.43975900	1.45317300
P	5.70365300	-0.13141000	-0.50507000
C	5.85721000	1.50011200	0.27315400
C	6.07557800	4.07447700	1.33482100
C	5.56564700	1.73013400	1.62153900
C	6.26697400	2.56382700	-0.53944500
C	6.37535000	3.84762600	-0.00942200
C	5.67240200	3.01684200	2.14947700
H	5.26025500	0.90712100	2.26243600
H	6.50678000	2.37809700	-1.58276000
H	6.69596400	4.66893000	-0.64236400
H	5.44675900	3.19114300	3.19673700
H	6.16165700	5.07458400	1.74809600
C	5.83690800	-1.32067200	0.85860100
C	6.16721500	-3.10950200	2.97813500
C	4.71900400	-1.84391300	1.51528900
C	7.12196700	-1.70409400	1.26216900
C	7.28604400	-2.59549600	2.31967000
C	4.88581400	-2.73557800	2.57546500
H	3.71937100	-1.56176700	1.19572700
H	7.98796100	-1.30790900	0.73888100
H	8.28352600	-2.89243400	2.62776100
H	4.01554400	-3.14206400	3.08085600
H	6.29534300	-3.80611800	3.80080200
O	6.74208500	-0.36086300	-1.55415600

OPDPO-S_1 Geometry (Solvent: THF)

H	-2.19391000	-1.83562100	3.50939000
C	-2.41772500	-1.15504700	2.69416000
C	-2.97631100	0.65756100	0.65470000
C	-1.38533500	-0.34361100	2.19642500
C	-3.69856400	-1.08900200	2.16140200
C	-3.98800500	-0.18458100	1.12933400
C	-1.68978400	0.57508900	1.18134100
H	-4.48632400	-1.72891300	2.54994900
H	-0.92405200	1.25619600	0.82311300
H	-3.19255800	1.39159300	-0.11710300
C	-0.03904300	-0.42669000	2.85221100
C	1.16395100	-0.29030600	2.07759300
C	3.62968400	-0.08372600	0.70546600
C	2.41092800	-0.18398900	2.78053000
C	1.23000400	-0.33663900	0.64823400
C	2.42656800	-0.23519600	-0.02359400
C	3.60916400	-0.07328500	2.11921600
H	2.38224700	-0.18113800	3.86467500
H	0.32661700	-0.49305600	0.06865700
H	2.46000200	-0.28559600	-1.10845700
H	4.54288800	0.02507600	2.66694300
O	-0.01585600	-0.62522000	4.09747000
N	4.87199000	0.04134500	0.00837400
C	5.56320400	-1.12511500	-0.30321300
C	6.90476000	-3.53063700	-0.90154200
C	5.01918600	-2.37421500	0.07951800
C	6.80071300	-1.11903400	-0.99429200
C	7.45718500	-2.32445700	-1.28560100
C	5.67915100	-3.54878500	-0.21513000
H	4.07641700	-2.39736300	0.60896500
H	8.40388200	-2.29314500	-1.81577500
H	5.24168900	-4.49251900	0.08969100
H	7.41881400	-4.45710500	-1.13046100

C	5.30949500	1.32303800	-0.31039300
C	6.13153300	3.95017400	-0.91653300
C	6.52379500	1.56789400	-0.99918300
C	4.51910800	2.43569900	0.06370500
C	4.92521900	3.71945000	-0.23455700
C	6.92048600	2.88121900	-1.29395900
H	3.58918400	2.26675600	0.58951100
H	4.30226700	4.55470900	0.06410800
H	7.85515400	3.04264800	-1.82182500
H	6.44609100	4.96143300	-1.14773300
S	7.58860200	0.31889000	-1.52169900
P	-5.66258300	-0.17995500	0.45842700
C	-5.86470900	1.42439900	-0.35915100
C	-6.16753700	3.96465900	-1.47823200
C	-5.58888700	1.63059900	-1.71450300
C	-6.30074600	2.49413300	0.43229600
C	-6.45064700	3.76109800	-0.12654200
C	-5.73863600	2.90082900	-2.27113900
H	-5.26196000	0.80321800	-2.33839300
H	-6.52685500	2.32666800	1.48173300
H	-6.79056700	4.58729900	0.48961700
H	-5.52575600	3.05700400	-3.32383300
H	-6.28632200	4.95172300	-1.91402600
C	-5.67819200	-1.41745300	-0.86446200
C	-5.83421700	-3.29483000	-2.92454300
C	-4.52223900	-1.79951900	-1.55361900
C	-6.91112800	-1.98633300	-1.20392400
C	-6.98789100	-2.92279800	-2.23286400
C	-4.60263600	-2.73511800	-2.58442100
H	-3.55960900	-1.37337100	-1.28297300
H	-7.80227200	-1.69926300	-0.65281100
H	-7.94466400	-3.36488100	-2.49173800
H	-3.70389100	-3.03147600	-3.11576300
H	-5.89423300	-4.02627100	-3.72440500
O	-6.71245900	-0.43600400	1.48786800

DBT-BZ-PXZ-S_0 Geometry (Solvent: THF)

H	3.92316900	-1.67065400	1.10561900
C	3.82402400	-0.68012500	0.67148500
C	3.50681600	1.90651300	-0.40749100
C	2.57455800	-0.06132800	0.67381500
C	4.92151500	-0.02587600	0.11133900
C	4.74991100	1.26840900	-0.42400800
C	2.42309100	1.23143900	0.13451600
H	1.45516300	1.72101500	0.16443500
H	3.39027800	2.90806700	-0.80739700
C	1.44010200	-0.78644100	1.32092700
O	1.64951100	-1.59241500	2.21511700
C	0.03392000	-0.52971900	0.86814500
C	-2.61766500	-0.18740000	0.09309500
C	-0.27493800	-0.24480800	-0.46513900
C	-0.99524200	-0.65403600	1.81044300
C	-2.31763900	-0.47192000	1.42831700
C	-1.60389500	-0.08210600	-0.85438600
H	0.51445300	-0.17177800	-1.20666400
H	-0.74319700	-0.89049900	2.83914000
H	-3.12464300	-0.55186700	2.15116400
H	-1.86321100	0.12542900	-1.88750900
N	-3.97778700	-0.00781200	-0.31454000
C	-4.62155300	1.20466500	0.00446100
C	-6.00338700	3.59122200	0.54867800
C	-3.95260800	2.30621500	0.54115800
C	-5.99511900	1.32278600	-0.25729100
C	-6.68291400	2.49222300	0.01721900
C	-4.64050600	3.49327000	0.80516100

H	-2.88962500	2.23977000	0.74491300
H	-7.74500300	2.52973300	-0.20167100
H	-4.09750700	4.33815300	1.21528000
H	-6.54092800	4.51013100	0.75503900
C	-4.78622800	-1.15104000	-0.47878900
C	-6.48762100	-3.35240600	-0.87712200
C	-6.15530800	-0.97280600	-0.72902500
C	-4.28490300	-2.45313200	-0.43363500
C	-5.13170400	-3.54544600	-0.63798400
C	-6.99996700	-2.05317600	-0.91697400
H	-3.22884300	-2.61430600	-0.24752700
H	-4.71761000	-4.54761300	-0.60599200
H	-8.05100800	-1.85986300	-1.10490200
H	-7.14793800	-4.19818400	-1.03397500
O	-6.69470500	0.29107300	-0.84721300
S	6.22882700	1.92400200	-1.06780900
C	7.11264600	0.47822800	-0.63438300
C	8.21239300	-1.93221200	0.17777500
C	8.47318100	0.25141100	-0.85564200
C	6.29679200	-0.48488600	-0.01236300
C	6.86098400	-1.70080000	0.39482200
C	9.01309600	-0.96089200	-0.44415000
H	9.09187200	1.00241500	-1.33590800
H	6.24544100	-2.45456100	0.87676200
H	10.06791400	-1.15719700	-0.60644600
H	8.65505900	-2.87174500	0.49127700

DBT-BZ-PXZ-T_1 Geometry (Solvent: THF)

H	3.86943700	-0.91241600	1.69796700
C	3.79007100	-0.18472500	0.89543300
C	3.55495800	1.75590300	-1.12762800
C	2.56133900	0.45005800	0.68967400
C	4.89098600	0.11523100	0.08840800
C	4.76319300	1.08349700	-0.92555800
C	2.47167000	1.43344700	-0.32230700
H	1.54229600	1.97896000	-0.45358400
H	3.46729600	2.52587900	-1.88788500
C	1.43739300	0.12744900	1.61820300
O	1.72673500	-0.14703300	2.82644400
C	0.07232200	0.09988200	1.15829400
C	-2.65361300	-0.04185300	0.42038000
C	-0.33434900	0.10527900	-0.21388700
C	-0.96815700	-0.02793400	2.13541100
C	-2.29850900	-0.08744400	1.77803400
C	-1.66532100	0.03958600	-0.57470400
H	0.41115800	0.12325600	-1.00099700
H	-0.67990800	-0.07084100	3.17953900
H	-3.07508400	-0.17052300	2.53467600
H	-1.95377100	0.03448100	-1.62279900
N	-4.04343100	-0.10709200	0.04344600
C	-4.81743000	1.03771200	0.04392500
C	-6.46573500	3.29822500	0.02074300
C	-4.28904400	2.29751100	0.39385600
C	-6.17969400	0.94850700	-0.31883300
C	-7.00281800	2.07266700	-0.33024200
C	-5.10813800	3.40714000	0.38034400
H	-3.24344300	2.37032000	0.66794600
H	-8.04204400	1.95542000	-0.61533600
H	-4.69880600	4.37390400	0.65002200
H	-7.09596500	4.18030500	0.01617200
C	-4.61042800	-1.31598000	-0.31249700
C	-5.84685100	-3.71431300	-1.05163500
C	-5.97478700	-1.35610800	-0.67671200
C	-3.87001800	-2.51625100	-0.31981700
C	-4.48658600	-3.69436600	-0.68611200
C	-6.59246100	-2.54913900	-1.04600300

H	-2.82540100	-2.49079200	-0.03375800
H	-3.91463200	-4.61507100	-0.69014700
H	-7.64178300	-2.53118200	-1.31727100
H	-6.31606700	-4.64904800	-1.33701400
O	-6.73350300	-0.23474300	-0.67424200
S	6.24715400	1.32080200	-1.81877400
C	7.07276000	0.12398500	-0.84826400
C	8.08194000	-1.81208700	0.86046200
C	8.40747400	-0.26599400	-0.98417400
C	6.23581200	-0.44257900	0.13243200
C	6.75580000	-1.42049600	0.99034200
C	8.90324400	-1.23721300	-0.12234700
H	9.04153700	0.17950600	-1.74411400
H	6.12318700	-1.86708000	1.75188200
H	9.93773900	-1.55293400	-0.21192600
H	8.48782300	-2.56870200	1.52394900

DBT-BZ-PXZ-S_1 Geometry (Solvent: THF)

H	3.81593700	-0.97807000	1.65790100
C	3.75257600	-0.18831800	0.91454300
C	3.56131600	1.91206000	-0.94933400
C	2.55447100	0.51249700	0.78058400
C	4.84992600	0.12617400	0.10763000
C	4.74252200	1.17710100	-0.82333500
C	2.47924800	1.57003200	-0.14908000
H	1.56331300	2.14841200	-0.22306400
H	3.49204900	2.73856300	-1.64941600
C	1.42308900	0.16680400	1.70861100
O	1.71931200	-0.10309800	2.90238600
C	0.07011400	0.13727500	1.22913900
C	-2.64276500	-0.02399000	0.43909300
C	-0.30979700	0.17657200	-0.15213100
C	-0.98590000	-0.03046100	2.18904400
C	-2.30275600	-0.10230700	1.81474300
C	-1.62523300	0.09984400	-0.54303100
H	0.45284100	0.23227800	-0.92175000
H	-0.71171900	-0.09283200	3.23673900
H	-3.09040100	-0.21798100	2.55462400
H	-1.89471800	0.11661700	-1.59533800
N	-4.00474400	-0.11052100	0.03987600
C	-4.79681600	1.02571600	-0.00066000
C	-6.46052200	3.26749600	-0.10035700
C	-4.28919300	2.29356100	0.34440000
C	-6.14908000	0.91363900	-0.39411300
C	-6.97845600	2.02799700	-0.44435300
C	-5.11713600	3.39785700	0.29233200
H	-3.25238200	2.37707400	0.64585100
H	-8.00947900	1.89843800	-0.75236900
H	-4.72439800	4.37211900	0.55835000
H	-7.10063200	4.14161700	-0.13616000
C	-4.55512200	-1.33370700	-0.30783200
C	-5.73738900	-3.76013500	-1.02752000
C	-5.91076100	-1.39651700	-0.70120900
C	-3.79653600	-2.52046000	-0.28065700
C	-4.38760300	-3.71668900	-0.63757000
C	-6.50082200	-2.60291400	-1.05949700
H	-2.75810600	-2.47103400	0.02274300
H	-3.80219800	-4.62825800	-0.61546800
H	-7.54400300	-2.60727100	-1.35343600
H	-6.18980100	-4.70528300	-1.30533200
O	-6.68263600	-0.28331300	-0.73914000
S	6.21692300	1.42290600	-1.72864700
C	7.00923600	0.11818200	-0.87630500
C	7.96782600	-1.98799900	0.65039000
C	8.32238700	-0.31547100	-1.07508800
C	6.16920100	-0.48926000	0.07641200

C	6.66323300	-1.55289000	0.84286000
C	8.79258300	-1.37208700	-0.30428000
H	8.95935700	0.16186500	-1.81288800
H	6.02804700	-2.03132700	1.58259100
H	9.81000900	-1.72314200	-0.44353000
H	8.35426800	-2.81095800	1.24263500

DBT-BZ-PTZ-S_0 Geometry (Solvent: THF)

H	2.31241300	-0.00251800	-0.68025700
C	3.12073000	-0.69518500	-0.46436700
C	5.23203500	-2.49798400	0.05082200
C	2.94277200	-2.06566300	-0.64472100
C	4.35841600	-0.20969200	-0.03188100
C	5.40112700	-1.12002200	0.23088400
C	4.00568200	-2.95763100	-0.39692300
H	3.84287400	-4.01792000	-0.55924300
H	6.04356000	-3.18865400	0.25425200
C	1.66366200	-2.62940500	-1.16968300
C	0.37168800	-1.91414600	-0.90910400
C	-2.10064500	-0.70749600	-0.46407900
C	-0.63894500	-2.00592300	-1.87060900
C	0.13274100	-1.22436000	0.28624400
C	-1.10453200	-0.63013000	0.51159700
C	-1.86974900	-1.39317400	-1.65492900
H	-0.44863200	-2.55655400	-2.78611800
H	0.90184600	-1.17071300	1.05038000
H	-1.30540600	-0.10474800	1.44110600
H	-2.65965400	-1.44238500	-2.39747700
O	1.66211700	-3.66929900	-1.81107900
N	-3.38010300	-0.09065800	-0.26188400
C	-3.46369000	1.31270000	-0.44109000
C	-3.68604000	4.10198000	-0.80382100
C	-2.32510900	2.10578800	-0.63606500
C	-4.72025600	1.94251800	-0.45697400
C	-4.82505400	3.32336700	-0.60770100
C	-2.44041900	3.48227700	-0.82747300
H	-1.34240300	1.65091800	-0.65263500
H	-5.81061900	3.77930300	-0.59513300
H	-1.54083900	4.06779400	-0.98732900
H	-3.77598100	5.17401800	-0.94082900
C	-4.30338000	-0.76412800	0.57688500
C	-6.18143500	-2.07242000	2.22588000
C	-5.63411500	-0.31810500	0.65277500
C	-3.94124600	-1.89317400	1.32323300
C	-4.87608300	-2.54421600	2.12741100
C	-6.55202000	-0.94883400	1.48948200
H	-2.93073300	-2.27918800	1.27060100
H	-4.56804700	-3.42288300	2.68468800
H	-7.56697500	-0.56569300	1.53887900
H	-6.90598400	-2.57100100	2.86035700
S	-6.18778900	0.96832100	-0.41644900
S	6.85355900	-0.32955100	0.77452600
C	6.09821500	1.24265300	0.65489900
C	4.61624800	3.56538700	0.35688500
C	6.70019900	2.46759300	0.95326700
C	4.76500700	1.16568200	0.21078600
C	4.02347500	2.34527700	0.06283600
C	5.94737800	3.62511200	0.80005700
H	7.72890100	2.51322200	1.29538000
H	2.99392700	2.30478900	-0.28061700
H	6.39612300	4.58693700	1.02588600
H	4.04714900	4.48206200	0.24353900

DBT-BZ-PTZ-T_1 Geometry (Solvent: THF)

H	2.49787000	0.32244300	-0.69014700
C	3.23987400	-0.45770900	-0.54594200
C	5.22090800	-2.44058400	-0.27448900
C	2.95131000	-1.77536400	-0.91479300
C	4.49655300	-0.12337600	-0.02873900
C	5.47810000	-1.12095900	0.11196000
C	3.97145600	-2.74804700	-0.78834400
H	3.74902400	-3.75953900	-1.11199900
H	5.98387400	-3.20679200	-0.17683300
C	1.65854200	-2.19828900	-1.52694400
C	0.40668300	-1.60761900	-1.12397500
C	-2.14271700	-0.58910700	-0.43638200
C	-0.76140400	-1.90986100	-1.89665200
C	0.21019400	-0.80057200	0.04092500
C	-1.03400400	-0.30206500	0.37456500
C	-2.00303800	-1.40548200	-1.56760800
H	-0.64268200	-2.54818800	-2.76478600
H	1.04052300	-0.59980700	0.70873300
H	-1.16502200	0.29999600	1.27037100
H	-2.87454600	-1.63329300	-2.17699900
O	1.69344600	-3.11242500	-2.41305500
N	-3.44001200	-0.05038500	-0.09038300
C	-3.79042100	1.18148600	-0.62531300
C	-4.44409300	3.68843400	-1.74783500
C	-2.88786700	1.84915400	-1.48834100
C	-5.02753100	1.81176200	-0.34355600
C	-5.34051100	3.06088300	-0.91010800
C	-3.21174300	3.07216200	-2.03368300
H	-1.93756900	1.38497400	-1.71674700
H	-6.29672100	3.51876100	-0.67678400
H	-2.50262400	3.56051600	-2.69270600
H	-4.69008800	4.65048700	-2.18251000
C	-4.24682500	-0.79885400	0.75521100
C	-5.83760000	-2.36689100	2.48224800
C	-5.52709200	-0.36003000	1.17468700
C	-3.78755400	-2.05181000	1.22890700
C	-4.56789200	-2.81314200	2.07124100
C	-6.30912200	-1.15142200	2.03560200
H	-2.81309900	-2.40627400	0.91982400
H	-4.19188000	-3.76905000	2.41841700
H	-7.28612600	-0.78893600	2.33974700
H	-6.44405700	-2.97327700	3.14519600
S	-6.22442400	1.13408200	0.68793500
S	6.97413500	-0.50200800	0.77208700
C	6.31450700	1.11348800	0.86632800
C	4.97620200	3.54208300	0.88253800
C	6.98119100	2.24844400	1.33500100
C	4.98498400	1.17793500	0.40572300
C	4.31827500	2.41022700	0.41912000
C	6.30154200	3.46073800	1.33851500
H	8.00592300	2.18410800	1.68686700
H	3.29271100	2.47762300	0.06732900
H	6.80338300	4.35343600	1.69780400
H	4.46281700	4.49802900	0.89279700

DBT-BZ-PTZ-S_1 Geometry (Solvent: THF)

H	2.53004000	0.36744300	-0.81955300
C	3.24155800	-0.42941300	-0.62031000
C	5.11675800	-2.48349800	-0.17243700
C	2.92695600	-1.74339300	-0.96286500
C	4.48306600	-0.12752100	-0.04735300
C	5.40880300	-1.16181200	0.17951900
C	3.88566200	-2.75600000	-0.74866500
H	3.63765800	-3.76856500	-1.05050400
H	5.83857800	-3.27626300	-0.00331700
C	1.64109200	-2.12048100	-1.64299200

C	0.39495600	-1.55463200	-1.21038700
C	-2.14884700	-0.56386300	-0.45781900
C	-0.77959400	-1.81495700	-1.99697700
C	0.21019500	-0.80788200	-0.00105800
C	-1.02246200	-0.32315100	0.36820000
C	-2.01275700	-1.32950000	-1.64155000
H	-0.66433100	-2.40769100	-2.89810500
H	1.04961300	-0.64275700	0.66609400
H	-1.14617300	0.23000000	1.29523700
H	-2.88806400	-1.52292700	-2.25646200
O	1.71304100	-2.94915400	-2.59006700
N	-3.42596100	-0.04265900	-0.08604700
C	-3.78119200	1.21236300	-0.57129200
C	-4.42885900	3.75547900	-1.59898000
C	-2.87557700	1.91091800	-1.40463800
C	-5.02053100	1.82887100	-0.26707500
C	-5.32978400	3.09493900	-0.78657300
C	-3.19631700	3.15524400	-1.90530500
H	-1.92516900	1.45422700	-1.64530500
H	-6.28602400	3.54549700	-0.53988000
H	-2.48547700	3.66928200	-2.54200900
H	-4.67639400	4.73326700	-1.99608200
C	-4.23220800	-0.82121100	0.73894600
C	-5.80350000	-2.45470900	2.41360600
C	-5.51308300	-0.40045500	1.17615700
C	-3.76376500	-2.08655500	1.16560500
C	-4.53613100	-2.88293100	1.98501200
C	-6.28397100	-1.22410700	2.01021000
H	-2.78889100	-2.42249400	0.83893000
H	-4.15483200	-3.84826200	2.29756200
H	-7.26164900	-0.87945000	2.33192700
H	-6.40636500	-3.08454500	3.05775800
S	-6.22046100	1.10785300	0.73695800
S	6.89240000	-0.58568800	0.90121800
C	6.30197400	1.05922500	0.90766000
C	5.07384700	3.54152800	0.77514500
C	6.99296200	2.17952000	1.37645700
C	5.00350700	1.16536500	0.37297100
C	4.39180000	2.42446400	0.31128000
C	6.36824100	3.41881300	1.30500200
H	7.99353000	2.08341000	1.78566600
H	3.39061800	2.52393700	-0.09813500
H	6.88926200	4.30082800	1.66328100
H	4.60364300	4.51830000	0.72799800

DBT-BZ-DMAC-S_0 Geometry (Solvent: THF)

H	4.42329200	-1.70464800	1.08478600
C	4.28626600	-0.70270900	0.68850600
C	3.87470200	1.91179000	-0.28696300
C	3.03074300	-0.10351200	0.77962400
C	5.34147300	-0.01472200	0.08850300
C	5.12309900	1.29316500	-0.39406500
C	2.83218900	1.20322600	0.29175200
H	1.86056500	1.67629300	0.39022600
H	3.72229400	2.92351200	-0.64727300
C	1.94163300	-0.86536100	1.46223400
C	0.51167500	-0.61967800	1.08583400
C	-2.17994800	-0.29438600	0.44818100
C	0.13494400	-0.29966900	-0.22336800
C	-0.46920800	-0.78752700	2.07020200
C	-1.81201100	-0.61306100	1.75631800
C	-1.21166800	-0.14660400	-0.54335600
H	0.88744400	-0.19240100	-0.99843200
H	-0.16549700	-1.05073600	3.07829200
H	-2.58357200	-0.72497900	2.51213200

H	-1.52125400	0.08745100	-1.55736100
O	2.20891500	-1.69090400	2.32255300
N	-3.56106300	-0.12204400	0.12177700
C	-4.30630500	-1.23361400	-0.29508700
C	-5.75499400	-3.49289600	-1.12506300
C	-3.68852300	-2.49501200	-0.38540100
C	-5.66706800	-1.09929100	-0.62450200
C	-6.35967200	-2.24340600	-1.03394900
C	-4.40638000	-3.61050800	-0.79588900
H	-2.64056900	-2.60256500	-0.13205500
H	-7.41130800	-2.15376900	-1.29142300
H	-3.90479000	-4.57126800	-0.85626100
H	-6.32724200	-4.35627500	-1.44705600
C	-4.13184300	1.15320200	0.22993300
C	-5.22657900	3.73047600	0.46078100
C	-5.48699300	1.35937100	-0.08552900
C	-3.34351500	2.23783400	0.65816800
C	-3.88644100	3.51084400	0.77157600
C	-6.00069700	2.65424500	0.03909800
H	-2.29973100	2.08259300	0.90397100
H	-3.25562200	4.32901200	1.10479900
H	-7.04566200	2.82748600	-0.20223000
H	-5.66318500	4.71986600	0.54462200
S	6.55438800	1.99033700	-1.09929600
C	7.47922100	0.54404200	-0.76547800
C	8.65387400	-1.87553800	-0.09900200
C	8.82695000	0.34364800	-1.07331500
C	6.71294300	-0.44987900	-0.12905400
C	7.31481800	-1.67026100	0.20397500
C	9.40486700	-0.87356300	-0.73421400
H	9.40670700	1.11840800	-1.56432200
H	6.73776600	-2.44799400	0.69528900
H	10.45059900	-1.04991500	-0.96414200
H	9.12541700	-2.81849200	0.15680600
C	-6.40843000	0.23444000	-0.55346900
C	-6.95613000	0.58160600	-1.95487800
H	-7.63597300	-0.19785100	-2.31048900
H	-6.13649000	0.67917700	-2.67282000
H	-7.51041100	1.52420600	-1.93292800
C	-7.58641000	0.10402000	0.43640800
H	-7.21977500	-0.14248900	1.43718100
H	-8.27664100	-0.68223500	0.11786000
H	-8.14993800	1.03950300	0.49563800

DBT-BZ-DMAC-T_1 Geometry (Solvent: THF)

H	4.52264400	-1.69371000	1.13382800
C	4.38582600	-0.76537000	0.59356800
C	3.86327200	1.70286300	-0.74678900
C	3.00866700	-0.27142300	0.42372700
C	5.42995500	-0.06867300	0.05372600
C	5.19609900	1.15814900	-0.64043500
C	2.81650000	1.01069400	-0.22430900
H	1.82190100	1.43720300	-0.26979300
H	3.70961400	2.66563200	-1.22348900
C	1.94976800	-1.04984400	0.99555500
C	0.51213100	-0.75401200	0.73594700
C	-2.22764100	-0.33732500	0.32292700
C	0.02990800	-0.37577300	-0.52568600
C	-0.40580700	-0.94746300	1.77892000
C	-1.76476200	-0.73193700	1.57848900
C	-1.33134200	-0.16742900	-0.73090900
H	0.71547100	-0.27288100	-1.36190700
H	-0.03576200	-1.26898300	2.74717300
H	-2.47728500	-0.86848100	2.38703900
H	-1.70985300	0.11548400	-1.70896700
O	2.24895700	-1.99821000	1.78283800

N	-3.62605000	-0.11874800	0.11216000
C	-4.42776100	-1.18701800	-0.31041700
C	-5.98894800	-3.36493600	-1.15888500
C	-3.85105300	-2.45527100	-0.51306300
C	-5.80496800	-1.00605400	-0.53444300
C	-6.55352400	-2.10978100	-0.95611500
C	-4.62402300	-3.52980600	-0.93225000
H	-2.79115100	-2.59798300	-0.33959300
H	-7.61808600	-1.98277500	-1.13203800
H	-4.15252600	-4.49641100	-1.07997600
H	-6.60403800	-4.19618900	-1.48655100
C	-4.15614900	1.15930800	0.32838200
C	-5.16600500	3.74402300	0.77767200
C	-5.52507600	1.41297100	0.12480200
C	-3.31180500	2.20246000	0.75429800
C	-3.81267900	3.47844500	0.97592100
C	-5.99560900	2.70953900	0.35694900
H	-2.25761300	2.00927100	0.91324800
H	-3.13858000	4.26323000	1.30493000
H	-7.05069000	2.91798500	0.20299700
H	-5.57013700	4.73630600	0.94722400
S	6.61240300	1.88130300	-1.23419500
C	7.61928000	0.59487900	-0.60035800
C	8.90396400	-1.57184200	0.52099900
C	9.00390400	0.51203800	-0.71974100
C	6.86165300	-0.38381000	0.06742900
C	7.51691100	-1.47677600	0.63188300
C	9.64073700	-0.58811100	-0.14805300
H	9.56840500	1.27905600	-1.23931400
H	6.95322800	-2.24511000	1.15192600
H	10.71884000	-0.67973100	-0.22421700
H	9.41822700	-2.42055200	0.95914500
C	-6.50623800	0.33622700	-0.33487300
C	-7.14676700	0.77439200	-1.66987000
H	-7.86944300	0.03049800	-2.01720500
H	-6.37966900	0.89687200	-2.44019000
H	-7.67521800	1.72501200	-1.55411700
C	-7.61158600	0.17373000	0.73113900
H	-7.17861800	-0.13673300	1.68658100
H	-8.34238400	-0.57910600	0.42207800
H	-8.14602000	1.11577700	0.88365300

DBT-BZ-DMAC-S_1 Geometry (Solvent: THF)

H	4.29137500	-0.99419600	1.64783900
C	4.21736200	-0.20110700	0.90906200
C	4.00011300	1.90734800	-0.94289500
C	3.01855200	0.50258100	0.79761100
C	5.30189900	0.11424500	0.08526200
C	5.18139400	1.16898700	-0.83976000
C	2.93085600	1.56466000	-0.12591900
H	2.01543100	2.14582100	-0.18197200
H	3.92110400	2.73687200	-1.63837000
C	1.90085800	0.15426700	1.74123800
C	0.54104100	0.13297500	1.28313200
C	-2.18667300	-0.00775600	0.53463200
C	0.13848300	0.18368800	-0.09120400
C	-0.50135300	-0.03582800	2.25771400
C	-1.82433700	-0.09688000	1.90357200
C	-1.18345300	0.11651900	-0.46151800
H	0.88847500	0.24017800	-0.87324500
H	-0.21149900	-0.10771500	3.30071200
H	-2.60131400	-0.21221700	2.65480100
H	-1.46964200	0.14162600	-1.50928600
O	2.21537300	-0.12424000	2.92878300
N	-3.55700000	-0.07702700	0.15558800
C	-4.10514200	-1.31100600	-0.17882400

C	-5.12480200	-3.81461200	-0.84116600
C	-3.27629000	-2.45711600	-0.11913200
C	-5.46114300	-1.41834300	-0.56980600
C	-5.93913000	-2.68498700	-0.89570300
C	-3.78827800	-3.69673700	-0.44909500
H	-2.24422200	-2.35110600	0.18777500
H	-6.97399800	-2.80157300	-1.19993800
H	-3.15001000	-4.57147100	-0.40161400
H	-5.53418800	-4.78429400	-1.10247900
C	-4.31264900	1.09085200	0.13128100
C	-5.75778600	3.46917000	0.11480800
C	-5.67801600	1.06152000	-0.23977500
C	-3.68517600	2.30868300	0.48663100
C	-4.40779300	3.48568600	0.47710600
C	-6.37198500	2.26891800	-0.23764700
H	-2.63917700	2.30543400	0.76338400
H	-3.92323600	4.41582700	0.75085800
H	-7.41992300	2.28158500	-0.51860400
H	-6.33234000	4.38891300	0.10491600
S	6.64083400	1.41464200	-1.76918300
C	7.44437100	0.10498400	-0.93511200
C	8.42363300	-2.00900500	0.56751000
C	8.75297600	-0.33093200	-1.15758500
C	6.61906500	-0.50405500	0.02941700
C	7.12344700	-1.57167300	0.78345600
C	9.23362000	-1.39142900	-0.39863700
H	9.37849100	0.14771600	-1.90428500
H	6.49970300	-2.05147000	1.53198500
H	10.24776300	-1.74423600	-0.55624500
H	8.81806900	-2.83502800	1.15013400
C	-6.38647200	-0.21872900	-0.63847700
C	-6.90596700	-0.07032800	-2.08918600
H	-7.44283400	-0.97144000	-2.39449800
H	-6.07597700	0.09400700	-2.78138700
H	-7.59439200	0.77475600	-2.16177600
C	-7.58002500	-0.45160300	0.31914500
H	-7.23225800	-0.56166500	1.34965700
H	-8.12475100	-1.35506200	0.03537300
H	-8.27517300	0.38982200	0.27298800

CP-BP-PXZ-S_0 Geometry (Solvent: THF)

H	0.96549700	0.43647200	-0.60476900
C	1.83361600	-0.21338000	-0.54827600
C	4.11536500	-1.90939800	-0.44076400
C	1.72173700	-1.58120100	-0.80997200
C	3.08521600	0.31661000	-0.24126500
C	4.21626200	-0.53394900	-0.19076600
C	2.86574700	-2.40972300	-0.75978100
H	2.74232600	-3.46641500	-0.97291400
H	4.98275400	-2.55836600	-0.38656600
C	0.43201000	-2.20317000	-1.21152100
O	0.40871700	-3.23182500	-1.87418300
C	-0.86513800	-1.56680000	-0.80240600
C	-3.34194600	-0.51873200	-0.07975800
C	-1.95154000	-1.65937900	-1.68163100
C	-1.03238500	-0.95913500	0.44530500
C	-2.27493100	-0.44217500	0.81014400
C	-3.18444300	-1.12629600	-1.32849200
H	-1.81415900	-2.14921600	-2.64028800
H	-0.20262500	-0.90768800	1.14327100
H	-2.42596600	0.02072800	1.78002000
H	-4.03111500	-1.17824400	-2.00724400
N	-4.60972500	0.02689800	0.29955300
C	-5.62585700	-0.85704100	0.71563300
C	-7.72007400	-2.50962600	1.60050600

C	-5.41224800	-2.21971400	0.93083800
C	-6.90765700	-0.33809800	0.95366200
C	-7.94540700	-1.14834800	1.38071700
C	-6.45352800	-3.03772100	1.37665200
H	-4.42801600	-2.64108400	0.75873500
H	-8.91780900	-0.69675900	1.54776700
H	-6.26146700	-4.09214500	1.54446200
H	-8.53082400	-3.14237100	1.94444700
C	-5.00699400	1.25923700	-0.25664400
C	-5.89792600	3.72655600	-1.26719400
C	-6.30496300	1.72396700	0.00521000
C	-4.16250800	2.05590300	-1.03206800
C	-4.60583900	3.28462700	-1.52804300
C	-6.75266800	2.93351100	-0.49736200
H	-3.15313200	1.71967700	-1.24170100
H	-3.92898600	3.88934000	-2.12226800
H	-7.76640500	3.24452200	-0.26716300
H	-6.24601100	4.67829200	-1.65320900
O	-7.16038500	1.01119600	0.81897800
C	4.94022200	1.54170100	0.31922700
C	3.69545600	4.00673400	0.50866500
C	5.72334900	2.65414100	0.63696000
C	3.55217600	1.64948500	0.08342200
C	2.92642900	2.89631400	0.18288800
C	5.07900300	3.88255300	0.72870000
H	6.79183700	2.56521400	0.80243600
H	1.85947000	2.99381300	0.00525100
H	5.65988400	4.76580900	0.97442300
H	3.22737200	4.98194500	0.59092800
N	5.32789300	0.21271700	0.15001700
C	6.65150500	-0.28670800	0.29393900
C	7.32625500	-0.11641400	1.50390400
C	7.26467600	-0.94074600	-0.77578100
C	8.62668900	-0.59811800	1.63806300
H	6.82813200	0.38409100	2.32879900
C	8.55846600	-1.43607900	-0.62660000
H	6.72977300	-1.05060200	-1.71440500
C	9.24248500	-1.26191400	0.57667700
H	9.15389000	-0.46399800	2.57695500
H	9.03535800	-1.94801100	-1.45599500
H	10.25272800	-1.64210500	0.68695100

CP-BP-PXZ-T_1 Geometry (Solvent: THF)

H	-1.08561000	0.82502700	0.88948700
C	-1.89741500	0.10604700	0.82234300
C	-4.05508000	-1.73156700	0.71977700
C	-1.70811900	-1.21386400	1.24087500
C	-3.14761500	0.51158700	0.34621200
C	-4.21801700	-0.40682100	0.29992500
C	-2.80591000	-2.10617000	1.19177800
H	-2.64653900	-3.11989100	1.54518900
H	-4.87374400	-2.44282400	0.67981600
C	-0.42604000	-1.71365800	1.82688700
O	-0.49692300	-2.54768200	2.78478800
C	0.84689100	-1.27375200	1.32056600
C	3.43175500	-0.54193600	0.43054900
C	2.02776000	-1.62110600	2.05858000
C	1.04663200	-0.57434100	0.08597000
C	2.30642400	-0.21688900	-0.34819600
C	3.28642500	-1.25642700	1.63190100
H	1.90335200	-2.17919200	2.97967200
H	0.19859300	-0.34710600	-0.55051300
H	2.43817300	0.30299900	-1.29388000
H	4.16799700	-1.51522400	2.21368900
N	4.74515600	-0.15173100	-0.01666500
C	5.44868100	-0.95444800	-0.89397000

C	6.94710100	-2.51178500	-2.67017200
C	4.92617500	-2.17315800	-1.37416300
C	6.73155500	-0.54769900	-1.32345100
C	7.47933200	-1.32117400	-2.20868800
C	5.67102800	-2.93524200	-2.24971100
H	3.94454900	-2.48959900	-1.04223800
H	8.45891700	-0.96838900	-2.51022900
H	5.26695500	-3.87180900	-2.61645800
H	7.51926900	-3.12293400	-3.35891700
C	5.30771200	1.02886500	0.42970000
C	6.52793000	3.39832000	1.27996800
C	6.59553200	1.39348600	-0.02171100
C	4.63323600	1.88636900	1.32326600
C	5.24149800	3.05262400	1.73806000
C	7.20563700	2.57270700	0.40086400
H	3.64403200	1.61008600	1.66819600
H	4.72061100	3.70951500	2.42516200
H	8.19512900	2.81072500	0.02768300
H	6.99205400	4.31853300	1.61630600
O	7.28229500	0.60940300	-0.88605100
C	-5.01326000	1.54939900	-0.50632900
C	-3.88501400	4.05707000	-0.86288300
C	-5.81895700	2.57540100	-1.00922200
C	-3.65775400	1.76854100	-0.16623600
C	-3.09374100	3.03459800	-0.35154200
C	-5.23444000	3.82540200	-1.18265100
H	-6.86298300	2.40675600	-1.25155100
H	-2.05389400	3.21536700	-0.09447800
H	-5.83720100	4.63994700	-1.57206900
H	-3.46198700	5.04497200	-1.01283500
N	-5.34309700	0.22826400	-0.22176300
C	-6.61852100	-0.36431300	-0.40322700
C	-7.22954100	-0.32980100	-1.65886800
C	-7.25854200	-0.98023800	0.67496100
C	-8.48741400	-0.90410400	-1.83008600
H	-6.71289300	0.13795100	-2.49153100
C	-8.50736100	-1.56963600	0.49046600
H	-6.77800300	-0.98680300	1.64863200
C	-9.12661400	-1.52904800	-0.75907700
H	-8.96189500	-0.87372300	-2.80566400
H	-9.00141700	-2.05064900	1.32850200
H	-10.10255600	-1.98265500	-0.89764800

CP-BP-PXZ-S_1 Geometry (Solvent: THF)

H	-1.08711300	0.87538000	0.98612100
C	-1.89228500	0.15044800	0.90310300
C	-4.01617000	-1.72385900	0.73874100
C	-1.69860700	-1.16632100	1.32020100
C	-3.13752200	0.53923100	0.40105200
C	-4.18955500	-0.39896300	0.32327200
C	-2.77384700	-2.07940000	1.24301800
H	-2.60554500	-3.09197100	1.59605200
H	-4.82167100	-2.44781100	0.67235400
C	-0.41386600	-1.64074800	1.93863600
O	-0.49868000	-2.40577700	2.93503800
C	0.85199700	-1.22757400	1.39890300
C	3.42478700	-0.53361300	0.44170600
C	2.04251300	-1.53800400	2.14232500
C	1.03034300	-0.59129100	0.12710900
C	2.27543100	-0.25171900	-0.34494100
C	3.29084900	-1.19664900	1.69047500
H	1.92636600	-2.04854500	3.09246400
H	0.16965700	-0.39899400	-0.50493000
H	2.39317300	0.21808100	-1.31741600
H	4.17982700	-1.42422400	2.27290100
N	4.71243100	-0.15782700	-0.02740200

C	5.42055600	-0.99098300	-0.87889400
C	6.90905800	-2.61398900	-2.59711900
C	4.89742200	-2.22681800	-1.30704900
C	6.69932400	-0.59509100	-1.33078600
C	7.44154600	-1.40144900	-2.18562600
C	5.63898100	-3.02444900	-2.15667200
H	3.91805500	-2.52908700	-0.95723200
H	8.41906300	-1.06132800	-2.50729500
H	5.23485800	-3.97501000	-2.48432300
H	7.48152600	-3.24907900	-3.26352700
C	5.27498700	1.04702800	0.36372600
C	6.47774100	3.45510300	1.11114900
C	6.55939200	1.39822600	-0.10809500
C	4.59805200	1.93368800	1.22396200
C	5.19831200	3.12303200	1.58886500
C	7.15976200	2.59567600	0.26303500
H	3.61304700	1.66264200	1.58343300
H	4.67552000	3.80365400	2.25043300
H	8.14614300	2.82671600	-0.12217300
H	6.93881700	4.39103000	1.40547000
O	7.24814900	0.58163200	-0.94212700
C	-4.99825000	1.54698200	-0.49323800
C	-3.90810200	4.07597700	-0.80996300
C	-5.81002600	2.55975700	-1.01267900
C	-3.65657200	1.78928300	-0.11812700
C	-3.11104200	3.06628800	-0.28310600
C	-5.24439800	3.82089600	-1.16555500
H	-6.84402200	2.37250000	-1.28271300
H	-2.08164600	3.26479500	0.00123200
H	-5.85174600	4.62614000	-1.56689800
H	-3.50007300	5.07230600	-0.94461300
N	-5.31107000	0.21828700	-0.22267200
C	-6.56936300	-0.39777100	-0.44606700
C	-7.13759400	-0.37417200	-1.72169800
C	-7.23383900	-1.02420900	0.61069400
C	-8.37859000	-0.97072200	-1.93480500
H	-6.60151100	0.10312800	-2.53647200
C	-8.46526500	-1.63556100	0.38466700
H	-6.78619900	-1.02195100	1.59997300
C	-9.04238600	-1.60621200	-0.88516400
H	-8.82042200	-0.94904200	-2.92580300
H	-8.97889600	-2.12492500	1.20588300
H	-10.00510500	-2.07675000	-1.05601800

CP-BP-DMAC-S_0 Geometry (Solvent: THF)

H	1.36710300	0.32342400	-0.76686000
C	2.25136500	-0.29420900	-0.64078500
C	4.57643400	-1.90792900	-0.34991700
C	2.19340100	-1.67572500	-0.84036200
C	3.47118800	0.29025400	-0.30569100
C	4.62402600	-0.51956600	-0.16389900
C	3.35839100	-2.46323100	-0.69898100
H	3.27701500	-3.53233700	-0.86532700
H	5.45969400	-2.52500700	-0.22532700
C	0.94295200	-2.35667200	-1.27055400
C	-0.39008300	-1.74655000	-0.94738300
C	-2.93062200	-0.75222100	-0.38082200
C	-1.43153900	-1.91099600	-1.86767200
C	-0.63304700	-1.09158500	0.26506100
C	-1.90554700	-0.60316500	0.55155000
C	-2.69645700	-1.40386300	-1.59234500
H	-1.23620400	-2.43518200	-2.79766600
H	0.16246100	-0.98278600	0.99569100
H	-2.11325700	-0.10606200	1.49427200
H	-3.51012900	-1.51049000	-2.30350200

O	0.98305400	-3.41242300	-1.88849000
N	-4.23307500	-0.23788300	-0.09140600
C	-5.16186300	-1.06224200	0.55815900
C	-6.98520300	-2.75624500	1.86129400
C	-4.80244800	-2.37682900	0.91023100
C	-6.45060300	-0.58763400	0.86146000
C	-7.33431300	-1.45598400	1.51067500
C	-5.70523000	-3.21247900	1.55415600
H	-3.81083400	-2.74614600	0.67738700
H	-8.33281800	-1.10191200	1.75114800
H	-5.40153200	-4.22180300	1.81370600
H	-7.70003400	-3.39874300	2.36409100
C	-4.54524600	1.07535700	-0.46672600
C	-5.11916300	3.71500000	-1.23973100
C	-5.81554100	1.61488500	-0.19430700
C	-3.58017600	1.86269100	-1.12257200
C	-3.86579800	3.16697000	-1.50386700
C	-6.06889800	2.93166000	-0.59181200
H	-2.60128900	1.44979300	-1.33551000
H	-3.10295400	3.75121900	-2.00897500
H	-7.04570900	3.36061700	-0.38679300
H	-5.35528900	4.73255700	-1.53197500
C	5.26095400	1.60046900	0.27487500
C	3.93390600	4.02976800	0.28812600
C	5.99429000	2.75191800	0.57174700
C	3.88235700	1.65148400	-0.02655000
C	3.21493800	2.88044300	-0.01627600
C	5.30909900	3.96155700	0.57464900
H	7.05638300	2.70601800	0.78799700
H	2.15475700	2.93408500	-0.24552100
H	5.85091800	4.87403300	0.80217400
H	3.43304400	4.99198800	0.30169600
C	-6.91542600	0.82547700	0.51350200
C	-8.14806400	0.73688600	-0.41230000
H	-8.96156000	0.19198600	0.07496600
H	-7.89251600	0.21870100	-1.34121300
H	-8.51823400	1.73485400	-0.66368900
C	-7.30641400	1.56129500	1.81355400
H	-6.44595700	1.63497200	2.48509900
H	-8.10730900	1.02947200	2.33511900
H	-7.66266000	2.57228800	1.59604500
N	5.69608300	0.27831300	0.18806900
C	7.02581500	-0.17130600	0.41530900
C	7.63851400	0.07775500	1.64444200
C	7.70665800	-0.85666000	-0.59212600
C	8.94451400	-0.35682500	1.86076600
H	7.08856400	0.60155900	2.42053300
C	9.00571800	-1.30432900	-0.36046200
H	7.21928900	-1.02803600	-1.54727100
C	9.62770000	-1.05176700	0.86236900
H	9.42336700	-0.16160200	2.81471600
H	9.53498400	-1.84076300	-1.14121000
H	10.64198100	-1.39546200	1.03678100

CP-BP-DMAC-T_1 Geometry (Solvent: THF)

H	1.54186000	0.78964800	-1.00392400
C	2.35210700	0.07595100	-0.88224100
C	4.50524400	-1.75309200	-0.63425600
C	2.17219000	-1.26333200	-1.23840700
C	3.59082800	0.50576900	-0.39732300
C	4.65901600	-0.40871700	-0.27828800
C	3.26778700	-2.15153900	-1.11765400
H	3.11627200	-3.18234100	-1.42163200
H	5.32165000	-2.46145600	-0.53736300
C	0.90312600	-1.79187800	-1.82704700
C	-0.37995700	-1.34059300	-1.35835400

C	-2.98522100	-0.60814100	-0.52767800
C	-1.54769800	-1.73197000	-2.09473800
C	-0.60376100	-0.59021000	-0.15858200
C	-1.87368400	-0.23267600	0.24648000
C	-2.81631300	-1.36855400	-1.69600900
H	-1.40529300	-2.32452700	-2.99134000
H	0.23348700	-0.32254900	0.47659500
H	-2.02325100	0.32440700	1.16805300
H	-3.68792900	-1.66413600	-2.27530400
O	0.99418300	-2.66322200	-2.75002300
N	-4.31334800	-0.23118100	-0.10247100
C	-5.01980500	-1.08801600	0.72932100
C	-6.38343500	-2.85273200	2.39569600
C	-4.41072700	-2.30209300	1.13398800
C	-6.32485400	-0.75396800	1.16514700
C	-6.97985500	-1.66032600	1.99853500
C	-5.09105700	-3.17202700	1.95920100
H	-3.41186000	-2.53679700	0.78922100
H	-7.98152800	-1.43452600	2.34883800
H	-4.62160600	-4.09973300	2.26603500
H	-6.92342800	-3.53367500	3.04471500
C	-4.84058100	0.97594000	-0.53890100
C	-5.83771200	3.41061700	-1.44904100
C	-6.13848300	1.37932300	-0.14286000
C	-4.05619000	1.79531700	-1.38810800
C	-4.55517200	3.00014300	-1.83558100
C	-6.60734200	2.60459900	-0.61649900
H	-3.06747700	1.46498400	-1.67976300
H	-3.95188000	3.62378900	-2.48542400
H	-7.59853300	2.94205800	-0.33209000
H	-6.23574300	4.35726000	-1.79828300
C	5.43544200	1.58616900	0.44782100
C	4.30099700	4.10830100	0.64834100
C	6.22922300	2.63666900	0.91806900
C	4.08909000	1.78727900	0.06318900
C	3.52188500	3.06092100	0.16974200
C	5.64179600	3.89361800	1.01324400
H	7.26671500	2.48094300	1.19461000
H	2.48901100	3.22803700	-0.12226700
H	6.23538900	4.72701200	1.37593100
H	3.87522900	5.10238300	0.73715500
C	-7.01461600	0.53176600	0.75686300
C	-8.31736300	0.17984200	-0.00392200
H	-8.98000500	-0.41266700	0.63089000
H	-8.09343400	-0.39303300	-0.90751900
H	-8.84775700	1.09095500	-0.28973100
C	-7.36065900	1.34298100	2.03008300
H	-6.45293100	1.60116700	2.58167700
H	-8.01494400	0.76297000	2.68485700
H	-7.88207900	2.26508500	1.76330900
N	5.77146200	0.25262500	0.23842000
C	7.03831900	-0.33131000	0.49261900
C	7.60945700	-0.22697500	1.76309600
C	7.71015300	-1.00819100	-0.52813500
C	8.85953900	-0.79220100	2.00639200
H	7.06832700	0.28785300	2.55127000
C	8.95079100	-1.58785900	-0.27100100
H	7.26082500	-1.06888500	-1.51472300
C	9.53046700	-1.47738600	0.99314400
H	9.30295100	-0.70738400	2.99330200
H	9.46959800	-2.11615600	-1.06442800
H	10.50026400	-1.92353600	1.18780900

CP-BP-DMAC-S_1 Geometry (Solvent: THF)

H	1.54226000	0.85819700	-1.07344700

C	2.34689300	0.13800200	-0.95214200
C	4.46936400	-1.72640900	-0.68582200
C	2.16449900	-1.19086000	-1.33432600
C	3.58037100	0.54392600	-0.43469800
C	4.63174000	-0.38947200	-0.30602600
C	3.23902400	-2.09902700	-1.20683100
H	3.07981500	-3.12174700	-1.53386100
H	5.27396300	-2.44667700	-0.58015800
C	0.89394700	-1.68706700	-1.96457000
C	-0.38315700	-1.26940900	-1.45803400
C	-2.98077800	-0.58528700	-0.55663700
C	-1.55879700	-1.61341600	-2.21038000
C	-0.58972900	-0.59967200	-0.20801800
C	-1.84651700	-0.26616200	0.23697600
C	-2.81800300	-1.27773500	-1.78556900
H	-1.42183800	-2.14727900	-3.14493400
H	0.25837800	-0.37637700	0.43099600
H	-1.98520300	0.22696300	1.19512000
H	-3.69544600	-1.53347500	-2.37387000
O	1.00205700	-2.47697100	-2.93980300
N	-4.28353100	-0.23504400	-0.10484200
C	-4.96726700	-1.10828200	0.73543900
C	-6.26572600	-2.90699200	2.41604000
C	-4.33557400	-2.31877400	1.10856700
C	-6.26501800	-0.79467800	1.20461700
C	-6.88632900	-1.71577400	2.04433800
C	-4.98521600	-3.20728600	1.94311400
H	-3.34492800	-2.53825000	0.73292100
H	-7.88224800	-1.50660500	2.42074900
H	-4.49771000	-4.13319200	2.22581200
H	-6.78239500	-3.59935400	3.07169500
C	-4.84243600	0.97076700	-0.51743100
C	-5.89147900	3.39482600	-1.39451900
C	-6.13968800	1.34906200	-0.09744800
C	-4.08333700	1.81028400	-1.36705300
C	-4.60937300	3.01216100	-1.79902600
C	-6.63539100	2.56739200	-0.55551500
H	-3.09228000	1.50014500	-1.67085400
H	-4.02427700	3.65174200	-2.44979900
H	-7.62758000	2.88640300	-0.25352500
H	-6.31232400	4.33633800	-1.73019200
C	5.41941300	1.58135000	0.47044800
C	4.31933500	4.11645900	0.68786200
C	6.21816700	2.61064000	0.97747600
C	4.08613500	1.80981000	0.05856000
C	3.53549500	3.09006800	0.17351200
C	5.64771600	3.87462400	1.08045400
H	7.24612000	2.43350000	1.27585200
H	2.51246200	3.27809400	-0.13942300
H	6.24500700	4.69251900	1.47117200
H	3.90707400	5.11551700	0.78377300
C	-6.98404000	0.48361500	0.81811600
C	-8.29701600	0.11505800	0.08565000
H	-8.93694900	-0.48986600	0.73209500
H	-8.08427900	-0.45149800	-0.82470100
H	-8.84762900	1.01871000	-0.18564000
C	-7.31099300	1.28391200	2.10199600
H	-6.39384100	1.55399800	2.63201200
H	-7.93945200	0.69136500	2.77085500
H	-7.85264900	2.19922600	1.85247000
N	5.74009300	0.24591600	0.24620200
C	6.99165000	-0.36223300	0.52243600
C	7.52678200	-0.29509400	1.81077300
C	7.68267200	-1.02457200	-0.49471400
C	8.76133500	-0.88392000	2.07620200
H	6.97036200	0.20982300	2.59470800
C	8.90726300	-1.62798600	-0.21605600

H	7.26092900	-1.05591700	-1.49480000
C	9.45146400	-1.55507800	1.06644300
H	9.17743100	-0.82834700	3.07696800
H	9.44142400	-2.14535700	-1.00645000
H	10.40900500	-2.01954700	1.27812400

a1-S_0 Geometry (Solvent: Toluene)

H	-2.81501900	1.24883300	1.66773600
C	-1.86949000	0.82296700	1.98770400
C	0.57822000	-0.25179200	2.77289800
C	-0.95245500	0.41946400	1.01220300
C	-1.57508300	0.70054300	3.33496300
C	-0.34256800	0.15683000	3.75105500
C	0.27858400	-0.11691500	1.42364300
H	-2.29233300	1.03065000	4.07855900
H	1.53396600	-0.68253500	3.05149800
C	-1.29602300	0.56817700	-0.42074200
O	-2.36653600	1.03424300	-0.77942000
C	1.29602300	-0.56817700	0.42074200
O	2.36653600	-1.03424300	0.77942000
C	0.95245500	-0.41946400	-1.01220300
C	0.34256800	-0.15683000	-3.75105500
C	-0.27858400	0.11691500	-1.42364300
C	1.86949000	-0.82296700	-1.98770400
C	1.57508300	-0.70054300	-3.33496300
C	-0.57822000	0.25179200	-2.77289800
H	2.81501900	-1.24883300	-1.66773600
H	2.29233300	-1.03065000	-4.07855900
H	-1.53396600	0.68253500	-3.05149800
N	0.04150100	-0.03063200	-5.10959000
C	1.07468200	0.13143800	-6.07494800
C	3.08035300	0.46301600	-7.99558700
C	2.09991900	1.05958300	-5.86624000
C	1.05250800	-0.62459100	-7.25064400
C	2.04891300	-0.45147600	-8.20782400
C	3.10205500	1.21436300	-6.82027600
H	2.10572700	1.65385400	-4.95723100
H	0.25013900	-1.33895900	-7.40890100
H	2.02278200	-1.04187500	-9.11824700
H	3.89469800	1.93588400	-6.64914100
H	3.85873300	0.59165100	-8.74042300
C	-1.30696000	-0.06749800	-5.56556900
C	-3.94148800	-0.15064500	-6.49845100
C	-1.75091600	0.87436500	-6.49818700
C	-2.18326800	-1.05531800	-5.10557800
C	-3.49698000	-1.08722200	-5.56508300
C	-3.06162200	0.82530600	-6.96566600
H	-1.06374000	1.63609800	-6.85415700
H	-1.83147400	-1.79097700	-4.38838100
H	-4.17136000	-1.85499400	-5.19938600
H	-3.39779300	1.56068500	-7.68977100
H	-4.96439900	-0.18186600	-6.85875100
N	-0.04150100	0.03063200	5.10959000
C	1.30696000	0.06749800	5.56556900
C	3.94148800	0.15064500	6.49845100
C	2.18326800	1.05531800	5.10557800
C	1.75091600	-0.87436500	6.49818700
C	3.06162200	-0.82530600	6.96566600
C	3.49698000	1.08722200	5.56508300
H	1.83147400	1.79097700	4.38838100
H	1.06374000	-1.63609800	6.85415700
H	3.39779300	-1.56068500	7.68977100
H	4.17136000	1.85499400	5.19938600
H	4.96439900	0.18186600	6.85875100
C	-1.07468200	-0.13143800	6.07494800
C	-3.08035300	-0.46301600	7.99558700

C	-2.09991900	-1.05958300	5.86624000
C	-1.05250800	0.62459100	7.25064400
C	-2.04891300	0.45147600	8.20782400
C	-3.10205500	-1.21436300	6.82027600
H	-2.10572700	-1.65385400	4.95723100
H	-0.25013900	1.33895900	7.40890100
H	-2.02278200	1.04187500	9.11824700
H	-3.89469800	-1.93588400	6.64914100
H	-3.85873300	-0.59165100	8.74042300

a1-T$_1$ Geometry (Solvent: Toluene)

H	-2.72152800	1.43296400	1.64680400
C	-1.80919600	0.94832700	1.97817400
C	0.59458800	-0.26616600	2.75406900
C	-0.91196200	0.48190600	0.98025800
C	-1.52408700	0.81569900	3.30884600
C	-0.29817800	0.19485500	3.71715700
C	0.30817700	-0.13347100	1.38970200
H	-2.20069700	1.20379500	4.06273000
H	1.52275500	-0.75349700	3.03469400
C	-1.26895800	0.65151600	-0.42036600
O	-2.33838300	1.20209000	-0.77156500
C	1.26895800	-0.65151600	0.42036600
O	2.33838300	-1.20209000	0.77156500
C	0.91196200	-0.48190600	-0.98025800
C	0.29817800	-0.19485500	-3.71715700
C	-0.30817700	0.13347100	-1.38970200
C	1.80919600	-0.94832700	-1.97817400
C	1.52408700	-0.81569900	-3.30884600
C	-0.59458800	0.26616600	-2.75406900
H	2.72152800	-1.43296400	-1.64680400
H	2.20069700	-1.20379500	-4.06273000
H	-1.52275500	0.75349700	-3.03469400
N	0.01041300	-0.08201000	-5.07967900
C	1.05186500	0.13239600	-6.01637600
C	3.10110400	0.55540000	-7.86444200
C	2.10095500	1.00877000	-5.71048000
C	1.02700900	-0.52689200	-7.25260600
C	2.04874100	-0.31039800	-8.16960600
C	3.12217300	1.21104000	-6.63346900
H	2.10123300	1.53127000	-4.75898400
H	0.21266300	-1.20779100	-7.47945800
H	2.02856300	-0.82832200	-9.12293500
H	3.93039000	1.89381700	-6.39269200
H	3.89750900	0.71902700	-8.58272000
C	-1.33107100	-0.12206300	-5.54105700
C	-3.96312700	-0.21405100	-6.46341100
C	-1.76014600	0.77975900	-6.52228100
C	-2.22076500	-1.07233100	-5.02401400
C	-3.53150900	-1.11097800	-5.48486100
C	-3.07262700	0.72773100	-6.97973400
H	-1.06689800	1.51891800	-6.91103000
H	-1.87733800	-1.77251900	-4.26930100
H	-4.21630100	-1.84971300	-5.08182400
H	-3.40295700	1.43459900	-7.73382600
H	-4.98727800	-0.24804800	-6.81962300
N	-0.01041300	0.08201000	5.07967900
C	1.33107100	0.12206300	5.54105700
C	3.96312700	0.21405100	6.46341100
C	2.22076500	1.07233100	5.02401400
C	1.76014600	-0.77975900	6.52228100
C	3.07262700	-0.72773100	6.97973400
C	3.53150900	1.11097800	5.48486100
H	1.87733800	1.77251900	4.26930100
H	1.06689800	-1.51891800	6.91103000
H	3.40295700	-1.43459900	7.73382600

H	4.21630100	1.84971300	5.08182400
H	4.98727800	0.24804800	6.81962300
C	-1.05186500	-0.13239600	6.01637600
C	-3.10110400	-0.55540000	7.86444200
C	-2.10095500	-1.00877000	5.71048000
C	-1.02700900	0.52689200	7.25260600
C	-2.04874100	0.31039800	8.16960600
C	-3.12217300	-1.21104000	6.63346900
H	-2.10123300	-1.53127000	4.75898400
H	-0.21266300	1.20779100	7.47945800
H	-2.02856300	0.82832200	9.12293500
H	-3.93039000	-1.89381700	6.39269200
H	-3.89750900	-0.71902700	8.58272000

a1-S_1 Geometry (Solvent: Toluene)

H	-2.57497900	1.66536900	1.64836400
C	-1.70677400	1.10509700	1.97941100
C	0.57903800	-0.31552700	2.76129300
C	-0.85719200	0.56416600	0.99096600
C	-1.42644300	0.95329400	3.31617300
C	-0.26450100	0.22948400	3.71669300
C	0.30063000	-0.15957800	1.39549500
H	-2.05992400	1.40332100	4.07380700
H	1.46343200	-0.87648500	3.04659300
C	-1.20542300	0.76693400	-0.42045400
O	-2.21626300	1.41101400	-0.76369800
C	1.20542300	-0.76693400	0.42045400
O	2.21626300	-1.41101400	0.76369800
C	0.85719200	-0.56416600	-0.99096600
C	0.26450100	-0.22948400	-3.71669300
C	-0.30063000	0.15957800	-1.39549500
C	1.70677400	-1.10509700	-1.97941100
C	1.42644300	-0.95329400	-3.31617300
C	-0.57903800	0.31552700	-2.76129300
H	2.57497900	-1.66536900	-1.64836400
H	2.05992400	-1.40332100	-4.07380700
H	-1.46343200	0.87648500	-3.04659300
N	-0.01672300	-0.10587800	-5.09096600
C	1.03352400	0.15202300	-5.99541800
C	3.13548100	0.64293900	-7.77410100
C	2.10586200	0.97189600	-5.61080900
C	1.01930900	-0.41942900	-7.27840300
C	2.06410900	-0.16878300	-8.15763400
C	3.15006000	1.20775200	-6.49929200
H	2.10336600	1.42902100	-4.62660600
H	0.19576100	-1.06451600	-7.56728100
H	2.04990100	-0.62057800	-9.14418200
H	3.97259100	1.84684500	-6.19527100
H	3.95044300	0.83223000	-8.46462500
C	-1.35243900	-0.16876900	-5.55122200
C	-3.99290200	-0.30015000	-6.45679200
C	-1.79647700	0.70669300	-6.55195900
C	-2.23779600	-1.10841200	-5.00309400
C	-3.54954900	-1.16740200	-5.45605100
C	-3.11057000	0.63363100	-7.00100200
H	-1.11352700	1.44496300	-6.95978600
H	-1.88831200	-1.78481000	-4.22983100
H	-4.22765200	-1.89895400	-5.02915300
H	-3.44970400	1.32081200	-7.76931700
H	-5.01882400	-0.34942500	-6.80590000
N	0.01672300	0.10587800	5.09096600
C	1.35243900	0.16876900	5.55122200
C	3.99290200	0.30015000	6.45679200
C	2.23779600	1.10841200	5.00309400
C	1.79647700	-0.70669300	6.55195900
C	3.11057000	-0.63363100	7.00100200

C	3.54954900	1.16740200	5.45605100
H	1.88831200	1.78481000	4.22983100
H	1.11352700	-1.44496300	6.95978600
H	3.44970400	-1.32081200	7.76931700
H	4.22765200	1.89895400	5.02915300
H	5.01882400	0.34942500	6.80590000
C	-1.03352400	-0.15202300	5.99541800
C	-3.13548100	-0.64293900	7.77410100
C	-2.10586200	-0.97189600	5.61080900
C	-1.01930900	0.41942900	7.27840300
C	-2.06410900	0.16878300	8.15763400
C	-3.15006000	-1.20775200	6.49929200
H	-2.10336600	-1.42902100	4.62660600
H	-0.19576100	1.06451600	7.56728100
H	-2.04990100	0.62057800	9.14418200
H	-3.97259100	-1.84684500	6.19527100
H	-3.95044300	-0.83223000	8.46462500

a2-S_0 Geometry (Solvent: Toluene)

H	1.65521900	-2.65263600	1.57642000
C	1.97926600	-1.74662900	1.07463100
C	2.77306800	0.60149700	-0.19945000
C	1.00859300	-0.88885000	0.54790100
C	3.32606900	-1.44207000	0.97477400
C	3.74547000	-0.26037100	0.33096900
C	1.42330700	0.29256000	-0.08789300
H	4.06680500	-2.11148800	1.39884000
H	3.05676400	1.51613600	-0.70929100
C	-0.42450600	-1.24324300	0.67315200
O	-0.78520500	-2.27057400	1.22608900
C	0.42452500	1.24318300	-0.67345500
O	0.78522600	2.27049900	-1.22642000
C	-1.00857400	0.88879900	-0.54818700
C	-3.74545600	0.26034900	-0.33119800
C	-1.42328900	-0.29260700	0.08761400
C	-1.97924900	1.74658500	-1.07490300
C	-3.32605300	1.44204100	-0.97501900
C	-2.77305100	-0.60153100	0.19919700
H	-1.65520400	2.65258600	-1.57670100
H	-4.06678800	2.11146500	-1.39907500
H	-3.05674100	-1.51616700	0.70904400
N	-5.10587700	-0.04672500	-0.22532300
C	-6.07979600	0.98676200	-0.15300600
C	-8.03068100	3.01405900	0.00702200
C	-5.89015900	2.08512000	0.69128900
C	-7.25115900	0.90139800	-0.91095900
C	-8.21412500	1.90038500	-0.82318200
C	-6.85049400	3.08783800	0.75905700
H	-4.98377200	2.15435300	1.28546300
H	-7.40838900	0.04193700	-1.55578300
H	-9.12965500	1.80642000	-1.39989000
H	-6.67578500	3.94656100	1.40070600
C	-5.54570100	-1.39791500	-0.16445000
C	-6.44944200	-4.06457800	-0.05771100
C	-6.51231100	-1.77923900	0.77033700
C	-5.03653100	-2.35253200	-1.05006400
C	-5.47790200	-3.66900100	-0.98686400
C	-6.96051000	-3.09448700	0.81421900
H	-6.91928700	-1.03781000	1.45131800
H	-4.28575100	-2.06181100	-1.77882200
H	-5.05145400	-4.40375900	-1.66344400
H	-7.72905500	-3.36905300	1.53084700
N	5.10589100	0.04672400	0.22511800
C	5.54570500	1.39792300	0.16442900
C	6.44943200	4.06461000	0.05804800

C	5.03641700	2.35245500	1.05006900
C	6.51242300	1.77934900	-0.77020600
C	6.96061400	3.09460500	-0.81391000
C	5.47778200	3.66893500	0.98704400
H	4.28555200	2.06166300	1.77871100
H	6.91948900	1.03799000	-1.45120900
H	7.72924300	3.36924400	-1.53042000
H	5.05124100	4.40362300	1.66364000
C	6.07981500	-0.98675500	0.15278900
C	8.03068800	-3.01406500	-0.00726100
C	5.89019400	-2.08508000	-0.69155400
C	7.25115600	-0.90143200	0.91078000
C	8.21411600	-1.90042400	0.82299200
C	6.85052200	-3.08780400	-0.75933200
H	4.98382400	-2.15428200	-1.28575600
H	7.40837600	-0.04200000	1.55564600
H	9.12963000	-1.80649000	1.39973100
H	6.67582300	-3.94650000	-1.40101900
C	6.92386400	5.46972200	-0.00038200
C	7.82309000	8.13051100	-0.11343100
C	7.18136700	6.08684300	-1.23181100
C	7.12391800	6.20848300	1.17343800
C	7.56875000	7.52720500	1.11781700
C	7.62782600	7.40491500	-1.28819100
H	7.00701200	5.53696600	-2.15235800
H	6.95103200	5.73835500	2.13748900
H	7.72480600	8.08133600	2.03823700
H	7.81402600	7.86870700	-2.25192100
H	8.16998900	9.15799700	-0.15711500
C	-9.05722600	4.08292100	0.08937900
C	-11.00074100	6.10778200	0.24426500
C	-9.36144000	4.69128000	1.31453300
C	-9.74335300	4.50618400	-1.05684000
C	-10.70698800	5.50895200	-0.98037000
C	-10.32376500	5.69523500	1.39152400
H	-8.85629500	4.35893300	2.21710200
H	-9.50291400	4.06221500	-2.01881000
H	-11.22254700	5.82897500	-1.88055300
H	-10.55126300	6.14951100	2.35090700
H	-11.75105900	6.88970200	0.30409800
C	-6.92388200	-5.46968000	0.00091200
C	-7.82313000	-8.13044400	0.11433100
C	-7.18122600	-6.08668300	1.23243400
C	-7.12410300	-6.20854800	-1.17281100
C	-7.56894700	-7.52725800	-1.11700700
C	-7.62769600	-7.40474300	1.28899800
H	-7.00673800	-5.53672100	2.15290500
H	-6.95133800	-5.73851200	-2.13693000
H	-7.72513600	-8.08147300	-2.03735400
H	-7.81377200	-7.86844300	2.25279600
H	-8.17003800	-9.15792200	0.15815800
C	9.05722700	-4.08293200	-0.08963100
C	11.00073000	-6.10780300	-0.24454000
C	9.36146400	-4.69125100	-1.31479800
C	9.74332500	-4.50623800	1.05659000
C	10.70695400	-5.50901100	0.98010900
C	10.32378300	-5.69521200	-1.39180000
H	8.85634300	-4.35887100	-2.21736700
H	9.50286600	-4.06230000	2.01856900
H	11.22249000	-5.82906700	1.88029200
H	10.55129900	-6.14945800	-2.35119300
H	11.75104300	-6.88972600	-0.30438200

a2-T_1 Geometry (Solvent: Toluene)

H	-2.49994400	1.76809000	1.66957300

C	-1.63081000	1.20482600	1.99263300
C	0.66006200	-0.22192100	2.74454600
C	-0.82208200	0.61080000	0.98877700
C	-1.31481900	1.09237300	3.31940900
C	-0.14726400	0.36433000	3.71206200
C	0.34144000	-0.11163000	1.38380200
H	-1.92256400	1.57386400	4.07842500
H	1.54355600	-0.79027800	3.01818200
C	-1.20946900	0.76242400	-0.40660100
O	-2.22851300	1.40734800	-0.74668600
C	1.20946900	-0.76242400	0.40660100
O	2.22851300	-1.40734800	0.74668600
C	0.82208200	-0.61080000	-0.98877700
C	0.14726400	-0.36433000	-3.71206200
C	-0.34144000	0.11163000	-1.38380200
C	1.63081000	-1.20482600	-1.99263300
C	1.31481900	-1.09237300	-3.31940900
C	-0.66006200	0.22192100	-2.74454600
H	2.49994400	-1.76809000	-1.66957300
H	1.92256400	-1.57386400	-4.07842500
H	-1.54355600	0.79027800	-3.01818200
N	-0.17447800	-0.27024200	-5.07369800
C	0.85325200	-0.15298600	-6.03705800
C	2.90698000	0.07233600	-7.94947800
C	1.98043800	0.63680500	-5.77350100
C	0.75353500	-0.82524500	-7.26320400
C	1.76796200	-0.70827700	-8.20185600
C	2.99149000	0.73979900	-6.71932500
H	2.05502300	1.16596000	-4.82870200
H	-0.11913500	-1.43650500	-7.47047800
H	1.67004700	-1.22182100	-9.15348000
H	3.86771400	1.33948600	-6.49263800
C	-1.52541600	-0.24498000	-5.49344200
C	-4.20776700	-0.21315100	-6.34520100
C	-1.93085500	0.62147100	-6.51691400
C	-2.46616700	-1.09479100	-4.89586800
C	-3.78680700	-1.07238700	-5.31893300
C	-3.25466400	0.63081500	-6.93354300
H	-1.20587500	1.28388000	-6.97908900
H	-2.15531300	-1.76437500	-4.10052700
H	-4.50814200	-1.72201500	-4.83289100
H	-3.55127100	1.29670700	-7.73823000
N	0.17447800	0.27024200	5.07369800
C	1.52541600	0.24498000	5.49344200
C	4.20776700	0.21315100	6.34520100
C	2.46616700	1.09479100	4.89586800
C	1.93085500	-0.62147100	6.51691400
C	3.25466400	-0.63081500	6.93354300
C	3.78680700	1.07238700	5.31893300
H	2.15531300	1.76437500	4.10052700
H	1.20587500	-1.28388000	6.97908900
H	3.55127100	-1.29670700	7.73823000
H	4.50814200	1.72201500	4.83289100
C	-0.85325200	0.15298600	6.03705800
C	-2.90698000	-0.07233600	7.94947800
C	-1.98043800	-0.63680500	5.77350100
C	-0.75353500	0.82524500	7.26320400
C	-1.76796200	0.70827700	8.20185600
C	-2.99149000	-0.73979900	6.71932500
H	-2.05502300	-1.16596000	4.82870200
H	0.11913500	1.43650500	7.47047800
H	-1.67004700	1.22182100	9.15348000
H	-3.86771400	-1.33948600	6.49263800
C	5.61975600	0.19860100	6.79604700
C	8.29502700	0.17223000	7.65335200
C	6.24684100	-1.00497300	7.14653600
C	6.35550900	1.38856600	6.88151700

C	7.68132800	1.37562700	7.30686600
C	7.57296500	-1.01802700	7.57090400
H	5.70013400	-1.93953700	7.05923900
H	5.87825500	2.33298100	6.63586100
H	8.23328300	2.30785500	7.37499600
H	8.04573300	-1.96068000	7.82817100
H	9.32873600	0.16187100	7.98379900
C	3.98968200	0.18739700	-8.95570000
C	6.04460300	0.40262600	-10.85940800
C	4.65756200	1.40380600	-9.15314700
C	4.36831200	-0.91924800	-9.72813900
C	5.38714000	-0.81276000	-10.67132500
C	5.67575700	1.51053600	-10.09701300
H	4.36066500	2.27804100	-8.58080800
H	3.87911900	-1.87624100	-9.57030200
H	5.67383300	-1.68287500	-11.25355200
H	6.17625500	2.46271300	-10.24237400
H	6.83878500	0.48566700	-11.59442200
C	-5.61975600	-0.19860100	-6.79604700
C	-8.29502700	-0.17223000	-7.65335200
C	-6.24684100	1.00497300	-7.14653600
C	-6.35550900	-1.38856600	-6.88151700
C	-7.68132800	-1.37562700	-7.30686600
C	-7.57296500	1.01802700	-7.57090400
H	-5.70013400	1.93953700	-7.05923900
H	-5.87825500	-2.33298100	-6.63586100
H	-8.23328300	-2.30785500	-7.37499600
H	-8.04573300	1.96068000	-7.82817100
H	-9.32873600	-0.16187100	-7.98379900
C	-3.98968200	-0.18739700	8.95570000
C	-6.04460300	-0.40262600	10.85940800
C	-4.65756200	-1.40380600	9.15314700
C	-4.36831200	0.91924800	9.72813900
C	-5.38714000	0.81276000	10.67132500
C	-5.67575700	-1.51053600	10.09701300
H	-4.36066500	-2.27804100	8.58080800
H	-3.87911900	1.87624100	9.57030200
H	-5.67383300	1.68287500	11.25355200
H	-6.17625500	-2.46271300	10.24237400
H	-6.83878500	-0.48566700	11.59442200

a3-S_0 Geometry (Solvent: Toluene)

H	1.48268400	2.18302200	-2.30111200
C	1.86668200	1.48214800	-1.56736200
C	2.80807500	-0.34923100	0.32054400
C	0.95913000	0.75295800	-0.79515300
C	3.23173300	1.29167400	-1.41109800
C	3.70905200	0.37797900	-0.45936800
C	1.43848800	-0.17043000	0.14565700
H	3.93893700	1.83038100	-2.03330500
H	3.15992200	-1.04220100	1.07801100
C	-0.49989300	0.97398500	-0.98802500
C	0.49984400	-0.97404900	0.98846300
O	-0.91867200	1.78403600	-1.79651800
O	0.91863300	-1.78405500	1.79699700
C	-0.95918000	-0.75300900	0.79560500
C	-3.70909600	-0.37800600	0.45983500
C	-1.43853700	0.17036800	-0.14521600
C	-1.86673300	-1.48219100	1.56782100
C	-3.23178000	-1.29166900	1.41159600
C	-2.80811900	0.34917200	-0.32010600
H	-1.48274700	-2.18305800	2.30158000
H	-3.93898400	-1.83036300	2.03381100
H	-3.15996100	1.04219400	-1.07752500
N	5.09309900	0.19221800	-0.29610900
N	-5.09314500	-0.19224100	0.29659700

C	5.75349400	-1.03909300	-0.23923300
C	7.54341900	-3.19750300	-0.12760100
C	5.24798800	-2.33607400	-0.35784600
C	7.13424200	-0.81272500	-0.09075600
C	8.02201500	-1.89348300	-0.03173900
C	6.15255400	-3.38636900	-0.29626900
H	4.19021400	-2.52923800	-0.49976700
H	9.08236000	-1.69568400	0.08420600
H	5.76198500	-4.39604200	-0.38410700
C	6.04815200	1.20642600	-0.18052300
C	8.32121600	2.82081100	0.14110700
C	7.32252700	0.62445600	-0.04965400
C	5.88411100	2.59285300	-0.12591600
C	7.02324900	3.36980100	0.02987000
C	8.45314000	1.43501200	0.10597000
H	4.90583300	3.05712100	-0.18976900
H	6.89891800	4.44808700	0.07094400
H	9.42593400	0.96493800	0.20388000
C	-6.04819900	-1.20645000	0.18095400
C	-8.32126100	-2.82081500	-0.14073600
C	-5.88419900	-2.59289700	0.12665000
C	-7.32253700	-0.62446200	0.04977300
C	-8.45314500	-1.43500800	-0.10588300
C	-7.02334100	-3.36983300	-0.02916900
H	-4.90596100	-3.05719900	0.19078300
H	-9.42591300	-0.96492800	-0.20405200
H	-6.89904600	-4.44813300	-0.07000200
C	-5.75348700	1.03907800	0.23932000
C	-7.54330000	3.19752300	0.12689400
C	-7.13421400	0.81272500	0.09063300
C	-5.24794800	2.33606900	0.35772000
C	-6.15246100	3.38638400	0.29573200
C	-8.02193200	1.89350200	0.03124000
H	-4.19019000	2.52922300	0.49975000
H	-5.76187300	4.39606400	0.38339100
H	-9.08226700	1.69572700	-0.08486200
C	8.45939900	-4.42389700	-0.06353800
C	8.32922300	-5.23189300	-1.36702400
H	8.98432500	-6.10939800	-1.33284600
H	8.61411100	-4.62186900	-2.23033300
H	7.30544600	-5.58405100	-1.52410700
C	9.93121800	-4.03511400	0.11425200
H	10.54359800	-4.94123000	0.15651200
H	10.09236700	-3.47935700	1.04393900
H	10.29216300	-3.42697200	-0.72186600
C	8.04795900	-5.30836400	1.12706700
H	8.69821200	-6.18825700	1.18472600
H	7.01596900	-5.65883300	1.03203100
H	8.13220100	-4.75446600	2.06763900
C	9.51800800	3.76361600	0.30268100
C	9.60295400	4.69698500	-0.91826200
H	9.72892900	4.11859700	-1.83926400
H	10.45814400	5.37409100	-0.81586400
H	8.70149700	5.30811200	-1.02262000
C	10.84195500	2.99978300	0.41517100
H	11.03883300	2.39920100	-0.47907500
H	10.85362600	2.33852400	1.28795000
H	11.66446800	3.71322400	0.52613900
C	9.33985400	4.60775200	1.57726100
H	9.27402100	3.96478900	2.46088600
H	8.43326100	5.21853800	1.53442300
H	10.19358600	5.28258800	1.70390900
C	-9.51807900	-3.76357000	-0.30232400
C	-8.45924500	4.42391100	0.06237400
C	-10.84193200	-2.99961200	-0.41507900
H	-11.66450600	-3.71297600	-0.52607600
H	-10.85341100	-2.33844700	-1.28793400

H	-11.03886400	-2.39890400	0.47907200
C	-9.33984300	-4.60788800	-1.57677600
H	-10.19363300	-5.28264500	-1.70344600
H	-8.43332500	-5.21877100	-1.53374400
H	-9.27382700	-3.96504700	-2.46047500
C	-9.60325500	-4.69676000	0.91874300
H	-10.45850800	-5.37378600	0.81634600
H	-9.72926600	-4.11823200	1.83965100
H	-8.70187600	-5.30797200	1.02328400
C	-9.93102900	4.03505300	-0.11554200
H	-10.29210200	3.42714700	0.72069300
H	-10.09201300	3.47901800	-1.04509200
H	-10.54341900	4.94114500	-0.15816400
C	-8.04763100	5.30806500	-1.12840600
H	-8.13175000	4.75392600	-2.06884600
H	-7.01565400	5.65855800	-1.03332400
H	-8.69787300	6.18794400	-1.18637900
C	-8.32926700	5.23224000	1.36567700
H	-8.61424000	4.62242100	2.22910300
H	-8.98440300	6.10970600	1.33119500
H	-7.30552500	5.58448000	1.52280300

a3-T_1 Geometry (Solvent: Toluene)

H	1.47310100	2.22212800	-2.24998700
C	1.86243800	1.51584600	-1.52431100
C	2.78618700	-0.37181400	0.32926600
C	0.92574200	0.76411500	-0.77159000
C	3.21173600	1.33606800	-1.36165400
C	3.68037200	0.37798800	-0.41625000
C	1.40318500	-0.19475600	0.16668400
H	3.92678300	1.87662600	-1.97310600
H	3.12858200	-1.07988300	1.07719200
C	-0.49701500	0.99869600	-0.98271500
C	0.49701400	-0.99874400	0.98266700
O	-0.91720900	1.84430900	-1.80519900
O	0.91720900	-1.84434100	1.80516600
C	-0.92574300	-0.76415500	0.77154900
C	-3.68037200	-0.37800900	0.41622800
C	-1.40318500	0.19471700	-0.16672400
C	-1.86243700	-1.51587800	1.52427900
C	-3.21173600	-1.33609100	1.36163100
C	-2.78618700	0.37178400	-0.32929700
H	-1.47310000	-2.22215900	2.24995600
H	-3.92678100	-1.87664100	1.97309100
H	-3.12858100	1.07985200	-1.07722400
N	5.06456200	0.18764100	-0.26562200
N	-5.06456200	-0.18765000	0.26561200
C	5.72164800	-1.04295100	-0.21218100
C	7.49958300	-3.20448600	-0.12845400
C	5.20630500	-2.33679700	-0.33036000
C	7.10698900	-0.81973700	-0.08389800
C	7.98926500	-1.89600500	-0.04005000
C	6.10822700	-3.39035600	-0.27936900
H	4.14635800	-2.52093400	-0.46219200
H	9.05255000	-1.70566300	0.05628900
H	5.71393400	-4.39827300	-0.36298200
C	6.01703400	1.20151700	-0.16059800
C	8.27786400	2.82535100	0.13841600
C	7.29688300	0.62351700	-0.04904300
C	5.83997600	2.58585400	-0.08337000
C	6.97759600	3.36901800	0.05537600
C	8.42034100	1.43328000	0.09505400
H	4.85502500	3.03806600	-0.11231900
H	6.84847800	4.44541600	0.11207600
H	9.39794400	0.97199400	0.18169600

C	-6.01704100	-1.20151900	0.16057800
C	-8.27788300	-2.82533400	-0.13844700
C	-5.83999300	-2.58585600	0.08332800
C	-7.29688600	-0.62351000	0.04903800
C	-8.42035100	-1.43326300	-0.09506400
C	-6.97761800	-3.36901000	-0.05542200
H	-4.85504500	-3.03807500	0.11226200
H	-9.39795000	-0.97196900	-0.18169300
H	-6.84850800	-4.44540900	-0.11214000
C	-5.72164100	1.04294600	0.21219300
C	-7.49956300	3.20449200	0.12850900
C	-7.10698400	0.81974300	0.08391400
C	-5.20629000	2.33678800	0.33039100
C	-6.10820500	3.39035300	0.27942200
C	-7.98925300	1.89601600	0.04008600
H	-4.14634200	2.52091500	0.46222500
H	-5.71390700	4.39826600	0.36305000
H	-9.05253900	1.70568200	-0.05625100
C	8.41377300	-4.43046400	-0.07581000
C	8.26074500	-5.23876200	-1.37733100
H	8.91587300	-6.11610200	-1.35021100
H	8.53385300	-4.63099800	-2.24587800
H	7.23480300	-5.59076500	-1.51788900
C	9.88901800	-4.04564900	0.08104400
H	10.49766000	-4.95421200	0.11664500
H	10.06582900	-3.48979000	1.00778500
H	10.24160200	-3.44168300	-0.76162100
C	8.01314100	-5.31071000	1.12240800
H	8.66328600	-6.19079100	1.17209600
H	6.97988600	-5.65991700	1.04056500
H	8.11058800	-4.75597700	2.06102800
C	9.47260400	3.76993100	0.28823300
C	9.52756500	4.71539700	-0.92583300
H	9.63881900	4.14743000	-1.85503700
H	10.38213100	5.39396000	-0.83167900
H	8.62245900	5.32436700	-1.00600700
C	10.80227400	3.01199100	0.36892000
H	10.98589700	2.41992200	-0.53374900
H	10.83606100	2.34538700	1.23698000
H	11.62196900	3.72980900	0.46903700
C	9.31038900	4.59980800	1.57498100
H	9.26203800	3.94818300	2.45321300
H	8.40198500	5.20855900	1.55302300
H	10.16455300	5.27518600	1.69277600
C	-9.47263000	-3.76990500	-0.28827000
C	-8.41374600	4.43047700	0.07588800
C	-10.80229900	-3.01195700	-0.36893200
H	-11.62199700	-3.72977000	-0.46905700
H	-10.83608900	-2.34533600	-1.23697900
H	-10.98591200	-2.41990500	0.53374900
C	-9.31043200	-4.59976000	-1.57503400
H	-10.16460000	-5.27513000	-1.69283300
H	-8.40203100	-5.20851700	-1.55309400
H	-9.26208400	-3.94812000	-2.45325500
C	-9.52758600	-4.71539200	0.92577900
H	-10.38215600	-5.39395000	0.83162000
H	-9.63883000	-4.14744200	1.85499500
H	-8.62248200	-5.32436700	1.00593500
C	-9.88899600	4.04567600	-0.08096000
H	-10.24157800	3.44170100	0.76170000
H	-10.06581800	3.48983100	-1.00770700
H	-10.49763100	4.95424300	-0.11654400
C	-8.01311700	5.31073600	-1.12232000
H	-8.11057400	4.75601600	-2.06094700
H	-6.97986000	5.65993600	-1.04047900
H	-8.66325800	6.19082100	-1.17199300
C	-8.26070400	5.23875800	1.37741800

H	-8.53381100	4.63098500	2.24596000
H	-8.91582600	6.11610300	1.35031300
H	-7.23475900	5.59075200	1.51797400

a3-S_1 Geometry (Solvent: Toluene)

H	1.47221700	-2.01863600	2.42743600
C	1.86228000	-1.37720100	1.64383200
C	2.79110300	0.32811400	-0.37465000
C	0.93298100	-0.69118000	0.83484100
C	3.21713400	-1.21484200	1.46372600
C	3.67948300	-0.35035500	0.43699500
C	1.40757800	0.17079100	-0.19109000
H	3.93475600	-1.70484400	2.11414100
H	3.13867200	0.97332600	-1.17565100
C	-0.49770700	-0.90066600	1.07602900
C	0.49771600	0.90011200	-1.07648200
O	-0.91359100	-1.66227900	1.97174400
O	0.91359900	1.66185400	-1.97208800
C	-0.93297200	0.69071500	-0.83521800
C	-3.67947400	0.35004500	-0.43724600
C	-1.40757100	-0.17125700	0.19071300
C	-1.86226900	1.37679400	-1.64416200
C	-3.21712400	1.21449600	-1.46400700
C	-2.79109700	-0.32849300	0.37434400
H	-1.47220600	2.01821500	-2.42777700
H	-3.93475000	1.70455100	-2.11437900
H	-3.13866800	-0.97369100	1.17535600
N	5.07198200	-0.16959800	0.27320500
N	-5.07198100	0.16943200	-0.27335000
C	5.73816900	1.05018900	0.22474600
C	7.52801300	3.19890000	0.12537900
C	5.22933400	2.34788300	0.33306800
C	7.12220000	0.81491600	0.08602200
C	8.00974900	1.88621000	0.03560800
C	6.13820600	3.39523600	0.27824400
H	4.16842500	2.53486700	0.45534000
H	9.07129700	1.69065500	-0.06932400
H	5.75148300	4.40652500	0.35621900
C	6.00542500	-1.19068700	0.15645100
C	8.23729200	-2.85071800	-0.13767800
C	7.29504200	-0.63014700	0.04275200
C	5.80466400	-2.57335900	0.09427800
C	6.92946000	-3.37470500	-0.04410900
C	8.40350800	-1.45951000	-0.10011200
H	4.81092800	-3.00556900	0.13724500
H	6.78501500	-4.44959800	-0.09123100
H	9.38940500	-1.01645300	-0.18759800
C	-6.00529800	1.19062600	-0.15651600
C	-8.23694400	2.85091500	0.13781400
C	-5.80437000	2.57327500	-0.09436800
C	-7.29496700	0.63023300	-0.04269300
C	-8.40332400	1.45972400	0.10027200
C	-6.92906000	3.37475100	0.04412100
H	-4.81058800	3.00536800	-0.13743200
H	-9.38926400	1.01678200	0.18785600
H	-6.78448700	4.44962800	0.09122200
C	-5.73829800	-1.05027800	-0.22482200
C	-7.52836700	-3.19878900	-0.12526500
C	-7.12229000	-0.81485000	-0.08596700
C	-5.22961400	-2.34802800	-0.33317400
C	-6.13859600	-3.39528100	-0.27826000
C	-8.00995100	-1.88604600	-0.03545700
H	-4.16873500	-2.53512700	-0.45554100
H	-5.75199200	-4.40661300	-0.35626000
H	-9.07146800	-1.69037400	0.06957400
C	8.45036300	4.41840300	0.06653000

C	8.30598900	5.23170400	1.36603300
H	8.96648500	6.10487000	1.33438200
H	8.57771400	4.62488100	2.23566200
H	7.28261100	5.59039600	1.50833500
C	9.92277000	4.02365400	-0.09269100
H	10.53723400	4.92818900	-0.13066700
H	10.09422100	3.46557700	-1.01909300
H	10.27304900	3.41845400	0.75005400
C	8.05313200	5.29819600	-1.13330200
H	8.70976100	6.17320900	-1.18724200
H	7.02277400	5.65544100	-1.04982900
H	8.14400500	4.73994900	-2.07049000
C	9.41627500	-3.81437100	-0.28960000
C	9.46176600	-4.75798700	0.92643700
H	9.58594500	-4.18979100	1.85384200
H	10.30532000	-5.44987200	0.82993700
H	8.54779200	-5.35275500	1.01199800
C	10.75784200	-3.07850900	-0.37812600
H	10.95550600	-2.48755600	0.52229700
H	10.79862000	-2.41457600	-1.24791700
H	11.56504800	-3.81004000	-0.48033300
C	9.23518400	-4.64479500	-1.57367200
H	9.19355300	-3.99460500	-2.45330000
H	8.31713400	-5.23863700	-1.54625000
H	10.07790300	-5.33409800	-1.69330700
C	-9.41580300	3.81470300	0.28983800
C	-8.45084600	-4.41819000	-0.06631800
C	-10.75744600	3.07899300	0.37847200
H	-11.56455900	3.81061600	0.48075900
H	-10.79822400	2.41505300	1.24825700
H	-10.95525700	2.48807500	-0.52194200
C	-9.23451100	4.64510200	1.57389700
H	-10.07714500	5.33449600	1.69360600
H	-8.31640000	5.23884500	1.54640100
H	-9.19287700	3.99490300	2.45351900
C	-9.46128300	4.75832800	-0.92619300
H	-10.30474600	5.45031400	-0.82961900
H	-9.58561000	4.19014800	-1.85358900
H	-8.54724600	5.35298500	-1.01183000
C	-9.92319600	-4.02327700	0.09302600
H	-10.27348000	-3.41804400	-0.74969400
H	-10.09450500	-3.46517200	1.01943800
H	-10.53775600	-4.92774200	0.13106300
C	-8.05360500	-5.29800900	1.13349100
H	-8.14433500	-4.73973800	2.07067900
H	-7.02329200	-5.65536700	1.04993200
H	-8.71032400	-6.17294900	1.18750200
C	-8.30667700	-5.23152500	-1.36582200
H	-8.57841000	-4.62468500	-2.23543600
H	-8.96726700	-6.10461700	-1.33410100
H	-7.28335000	-5.59033400	-1.50820900

a4-S_0 Geometry (Solvent: Toluene)

H	3.14912100	1.53005900	0.00000000
C	2.12979100	1.90132400	0.00000000
C	-0.51749500	2.80457400	0.00000000
C	1.07850700	0.98191500	0.00000000
C	1.85902700	3.26430300	0.00000000
C	0.53499700	3.71360700	0.00000000
C	-0.24876300	1.43547800	0.00000000
H	2.66233700	3.99496000	0.00000000
H	-1.54583000	3.15303900	0.00000000
C	1.39528400	-0.47746500	0.00000000
O	2.54654100	-0.87313200	0.00000000
C	-1.39528400	0.47746500	0.00000000

O	-2.54654100	0.87313200	0.00000000
C	-1.07850700	-0.98191500	0.00000000
C	-0.53499700	-3.71360700	0.00000000
C	0.24876300	-1.43547800	0.00000000
C	-2.12979100	-1.90132400	0.00000000
C	-1.85902700	-3.26430300	0.00000000
C	0.51749500	-2.80457400	0.00000000
H	-3.14912100	-1.53005900	0.00000000
H	-2.66233700	-3.99496000	0.00000000
H	1.54583000	-3.15303900	0.00000000
N	-0.26414200	-5.11442100	0.00000000
N	0.26414200	5.11442100	0.00000000
C	0.15503400	5.78615700	1.22686500
C	-0.06009200	7.08476000	3.70673300
C	0.28495400	5.06971900	2.43053500
C	-0.08554800	7.17072800	1.26191000
C	-0.18794500	7.78662100	2.51302600
C	0.17856500	5.71386800	3.65577900
H	0.46863200	4.00213700	2.40855100
H	-0.37602900	8.85568500	2.55486500
H	0.28222200	5.13688400	4.56927200
H	-0.14654200	7.60005200	4.65719900
C	0.15503400	5.78615700	-1.22686500
C	-0.06009200	7.08476000	-3.70673300
C	-0.08554800	7.17072800	-1.26191000
C	0.28495400	5.06971900	-2.43053500
C	0.17856500	5.71386800	-3.65577900
C	-0.18794500	7.78662100	-2.51302600
H	0.46863200	4.00213700	-2.40855100
H	0.28222200	5.13688400	-4.56927200
H	-0.37602900	8.85568500	-2.55486500
H	-0.14654200	7.60005200	-4.65719900
C	-0.24466300	8.01651000	0.00000000
C	0.82587200	9.12917700	0.00000000
H	0.72548600	9.76612500	0.88328700
H	0.72548600	9.76612500	-0.88328700
H	1.82968200	8.69474600	0.00000000
C	-1.64910100	8.65849200	0.00000000
H	-1.78887900	9.28697700	0.88423500
H	-2.42305400	7.88567000	0.00000000
H	-1.78887900	9.28697700	-0.88423500
C	-0.15503400	-5.78615700	-1.22686500
C	0.06009200	-7.08476000	-3.70673300
C	0.08554800	-7.17072800	-1.26191000
C	-0.28495400	-5.06971900	-2.43053500
C	-0.17856500	-5.71386800	-3.65577900
C	0.18794500	-7.78662100	-2.51302600
H	-0.46863200	-4.00213700	-2.40855100
H	-0.28222200	-5.13688400	-4.56927200
H	0.37602900	-8.85568500	-2.55486500
H	0.14654200	-7.60005200	-4.65719900
C	-0.15503400	-5.78615700	1.22686500
C	0.06009200	-7.08476000	3.70673300
C	-0.28495400	-5.06971900	2.43053500
C	0.08554800	-7.17072800	1.26191000
C	0.18794500	-7.78662100	2.51302600
C	-0.17856500	-5.71386800	3.65577900
H	-0.46863200	-4.00213700	2.40855100
H	0.37602900	-8.85568500	2.55486500
H	-0.28222200	-5.13688400	4.56927200
H	0.14654200	-7.60005200	4.65719900
C	0.24466300	-8.01651000	0.00000000
C	1.64910100	-8.65849200	0.00000000
H	1.78887900	-9.28697700	0.88423500
H	2.42305400	-7.88567000	0.00000000
H	1.78887900	-9.28697700	-0.88423500
C	-0.82587200	-9.12917700	0.00000000

H	-0.72548600	-9.76612500	0.88328700
H	-0.72548600	-9.76612500	-0.88328700
H	-1.82968200	-8.69474600	0.00000000

a4-T_1 Geometry (Solvent: Toluene)

H	3.15178900	1.49686800	0.00000000
C	2.13567000	1.87818000	0.00000000
C	-0.51054400	2.78977100	0.00000000
C	1.08008400	0.93830300	0.00000000
C	1.88159300	3.23174700	0.00000000
C	0.54041300	3.67521400	0.00000000
C	-0.26220000	1.40076000	0.00000000
H	2.68604100	3.96140900	0.00000000
H	-1.53951900	3.13748100	0.00000000
C	1.40273600	-0.48652400	0.00000000
O	2.58439400	-0.90112900	0.00000000
C	-1.40273600	0.48652400	0.00000000
O	-2.58439400	0.90112900	0.00000000
C	-1.08008400	-0.93830300	0.00000000
C	-0.54041300	-3.67521400	0.00000000
C	0.26220000	-1.40076000	0.00000000
C	-2.13567000	-1.87818000	0.00000000
C	-1.88159300	-3.23174700	0.00000000
C	0.51054400	-2.78977100	0.00000000
H	-3.15178900	-1.49686800	0.00000000
H	-2.68604100	-3.96140900	0.00000000
H	1.53951900	-3.13748100	0.00000000
N	-0.27634400	-5.09225100	0.00000000
N	0.27634400	5.09225100	0.00000000
C	0.16719400	5.75547900	1.21875700
C	-0.04823600	7.03721700	3.69585200
C	0.31052800	5.02608300	2.42030000
C	-0.08525400	7.14413400	1.25830200
C	-0.18866700	7.75189700	2.51036300
C	0.20405000	5.66440000	3.64248700
H	0.50112100	3.96103600	2.38161300
H	-0.38749300	8.81786300	2.56392900
H	0.31537400	5.08921500	4.55536800
H	-0.13619200	7.54528500	4.65008800
C	0.16719400	5.75547900	-1.21875700
C	-0.04823600	7.03721700	-3.69585200
C	-0.08525400	7.14413400	-1.25830200
C	0.31052800	5.02608300	-2.42030000
C	0.20405000	5.66440000	-3.64248700
C	-0.18866700	7.75189700	-2.51036300
H	0.50112100	3.96103600	-2.38161300
H	0.31537400	5.08921500	-4.55536800
H	-0.38749300	8.81786300	-2.56392900
H	-0.13619200	7.54528500	-4.65008800
C	-0.25714000	7.98220300	0.00000000
C	0.79533700	9.11501700	0.00000000
H	0.68024400	9.74849700	0.88321400
H	0.68024400	9.74849700	-0.88321400
H	1.80687600	8.69987500	0.00000000
C	-1.67583600	8.59873500	0.00000000
H	-1.82281800	9.22402400	0.88469200
H	-2.43616400	7.81303400	0.00000000
H	-1.82281800	9.22402400	-0.88469200
C	-0.16719400	-5.75547900	-1.21875700
C	0.04823600	-7.03721700	-3.69585200
C	0.08525400	-7.14413400	-1.25830200
C	-0.31052800	-5.02608300	-2.42030000
C	-0.20405000	-5.66440000	-3.64248700
C	0.18866700	-7.75189700	-2.51036300
H	-0.50112100	-3.96103600	-2.38161300

H	-0.31537400	-5.08921500	-4.55536800
H	0.38749300	-8.81786300	-2.56392900
H	0.13619200	-7.54528500	-4.65008800
C	-0.16719400	-5.75547900	1.21875700
C	0.04823600	-7.03721700	3.69585200
C	-0.31052800	-5.02608300	2.42030000
C	0.08525400	-7.14413400	1.25830200
C	0.18866700	-7.75189700	2.51036300
C	-0.20405000	-5.66440000	3.64248700
H	-0.50112100	-3.96103600	2.38161300
H	0.38749300	-8.81786300	2.56392900
H	-0.31537400	-5.08921500	4.55536800
H	0.13619200	-7.54528500	4.65008800
C	0.25714000	-7.98220300	0.00000000
C	1.67583600	-8.59873500	0.00000000
H	1.82281800	-9.22402400	0.88469200
H	2.43616400	-7.81303400	0.00000000
H	1.82281800	-9.22402400	-0.88469200
C	-0.79533700	-9.11501700	0.00000000
H	-0.68024400	-9.74849700	0.88321400
H	-0.68024400	-9.74849700	-0.88321400
H	-1.80687600	-8.69987500	0.00000000

a4-S_1 Geometry (Solvent: Toluene)

H	3.15553200	1.48716100	0.00000000
C	2.14034000	1.87123700	0.00000000
C	-0.50279700	2.78772200	0.00000000
C	1.08515900	0.93694300	0.00000000
C	1.89133800	3.22798700	0.00000000
C	0.55090000	3.67426500	0.00000000
C	-0.25769500	1.40348300	0.00000000
H	2.69793100	3.95458900	0.00000000
H	-1.53074600	3.13835400	0.00000000
C	1.40603700	-0.49136300	0.00000000
O	2.58150500	-0.90891700	0.00000000
C	-1.40603700	0.49136300	0.00000000
O	-2.58150500	0.90891700	0.00000000
C	-1.08515900	-0.93694300	0.00000000
C	-0.55090000	-3.67426500	0.00000000
C	0.25769500	-1.40348300	0.00000000
C	-2.14034000	-1.87123700	0.00000000
C	-1.89133800	-3.22798700	0.00000000
C	0.50279700	-2.78772200	0.00000000
H	-3.15553200	-1.48716100	0.00000000
H	-2.69793100	-3.95458900	0.00000000
H	1.53074600	-3.13835400	0.00000000
N	-0.28553200	-5.08711000	0.00000000
N	0.28553200	5.08711000	0.00000000
C	0.17376800	5.75036700	1.21862400
C	-0.04819300	7.03066500	3.69633500
C	0.32235000	5.02233400	2.42015100
C	-0.08672300	7.13767400	1.25851000
C	-0.19325400	7.74444700	2.51007700
C	0.21256900	5.66025400	3.64291500
H	0.52022300	3.95891800	2.38211300
H	-0.39855300	8.80920200	2.56371200
H	0.32817400	5.08564600	4.55557300
H	-0.13908900	7.53859900	4.65032000
C	0.17376800	5.75036700	-1.21862400
C	-0.04819300	7.03066500	-3.69633500
C	-0.08672300	7.13767400	-1.25851000
C	0.32235000	5.02233400	-2.42015100
C	0.21256900	5.66025400	-3.64291500
C	-0.19325400	7.74444700	-2.51007700
H	0.52022300	3.95891800	-2.38211300
H	0.32817400	5.08564600	-4.55557300

H	-0.39855300	8.80920200	-2.56371200
H	-0.13908900	7.53859900	-4.65032000
C	-0.26357700	7.97504900	0.00000000
C	0.78267900	9.11328300	0.00000000
H	0.66441000	9.74618000	0.88324000
H	0.66441000	9.74618000	-0.88324000
H	1.79639300	8.70345200	0.00000000
C	-1.68554200	8.58335300	0.00000000
H	-1.83626200	9.20778000	0.88470000
H	-2.44135500	7.79329800	0.00000000
H	-1.83626200	9.20778000	-0.88470000
C	-0.17376800	-5.75036700	-1.21862400
C	0.04819300	-7.03066500	-3.69633500
C	0.08672300	-7.13767400	-1.25851000
C	-0.32235000	-5.02233400	-2.42015100
C	-0.21256900	-5.66025400	-3.64291500
C	0.19325400	-7.74444700	-2.51007700
H	-0.52022300	-3.95891800	-2.38211300
H	-0.32817400	-5.08564600	-4.55557300
H	0.39855300	-8.80920200	-2.56371200
H	0.13908900	-7.53859900	-4.65032000
C	-0.17376800	-5.75036700	1.21862400
C	0.04819300	-7.03066500	3.69633500
C	-0.32235000	-5.02233400	2.42015100
C	0.08672300	-7.13767400	1.25851000
C	0.19325400	-7.74444700	2.51007700
C	-0.21256900	-5.66025400	3.64291500
H	-0.52022300	-3.95891800	2.38211300
H	0.39855300	-8.80920200	2.56371200
H	-0.32817400	-5.08564600	4.55557300
H	0.13908900	-7.53859900	4.65032000
C	0.26357700	-7.97504900	0.00000000
C	1.68554200	-8.58335300	0.00000000
H	1.83626200	-9.20778000	0.88470000
H	2.44135500	-7.79329800	0.00000000
H	1.83626200	-9.20778000	-0.88470000
C	-0.78267900	-9.11328300	0.00000000
H	-0.66441000	-9.74618000	0.88324000
H	-0.66441000	-9.74618000	-0.88324000
H	-1.79639300	-8.70345200	0.00000000

b1-S_0 Geometry (Solvent: Toluene)

C	3.69989400	0.69634500	-0.28437400
H	3.20939900	-1.27518000	0.46941900
C	2.82790800	-0.33080600	0.09366100
C	1.77292400	2.08853700	-0.79788900
C	1.44760500	-0.15896800	0.02561000
C	3.14769000	1.90821100	-0.73433700
C	0.91004700	1.05826400	-0.41919700
C	0.56014900	-1.28669700	0.44349900
H	3.80675200	2.70789700	-1.05850300
H	1.34701600	3.02209700	-1.15048900
C	-0.91105900	-1.06933500	0.36923000
C	-1.77386800	-2.09779100	0.75295400
C	-1.44870400	0.14637800	-0.07961400
H	-3.21005600	1.28701700	-0.45718200
C	-0.56124800	1.27429800	-0.49702800
C	-3.14858700	-1.91761200	0.68936700
H	-1.34791500	-3.03764000	1.08836600
H	-3.80762700	-2.73599400	0.96269600
C	-3.70111900	-0.70250600	0.24815400
C	-2.82896400	0.32351500	-0.13322000
O	1.02570700	-2.34550800	0.83026300
O	-1.02680400	2.33183100	-0.88728700
C	-5.16787500	-0.51250300	0.18882100

C	-7.96915200	-0.15164900	0.07517900
C	-5.75728100	0.25900900	-0.82209000
C	-6.01203100	-1.09776200	1.14253800
C	-7.38771700	-0.91641300	1.09655700
C	-7.13327100	0.43203000	-0.88760700
H	-5.13486500	0.70106900	-1.59474100
H	-5.58575800	-1.67204500	1.96011100
H	-8.02052600	-1.36024900	1.85844300
H	-7.56957200	1.01556100	-1.69196400
C	5.16735600	0.51097900	-0.21065600
C	7.96856400	0.15745300	-0.06727300
C	5.75509800	-0.72921900	-0.49405700
C	6.01263500	1.56990800	0.14675300
C	7.38963700	1.40338200	0.21313400
C	7.12995800	-0.90957700	-0.42010200
H	5.13014600	-1.56751600	-0.78810200
H	5.58887100	2.53961300	0.39161200
H	8.02441800	2.23796700	0.49289500
H	7.56253500	-1.87959800	-0.64300400
N	-9.36590800	0.02655900	0.01823700
N	9.36527900	-0.01834900	0.00549300
C	-9.91194900	1.26040300	-0.42022600
C	-10.99862200	3.69594700	-1.28085700
C	-9.35864200	2.47193600	0.01067800
C	-11.01435000	1.27606000	-1.28259600
C	-11.55620100	2.48839800	-1.70088000
C	-9.89573700	3.67942700	-0.42671600
H	-8.50766000	2.45966600	0.68501900
H	-11.44268100	0.33597100	-1.61683000
H	-12.41187300	2.48670100	-2.36897800
H	-9.45618900	4.61185300	-0.08637100
H	-11.41900400	4.63892200	-1.61430600
C	-10.23711300	-1.02994600	0.38817100
C	-11.96828400	-3.11255600	1.11028100
C	-11.38194100	-0.76495300	1.14878600
C	-9.96602500	-2.34422200	-0.01071400
C	-10.82368600	-3.37685400	0.35791700
C	-12.24346300	-1.80126200	1.49815400
H	-11.59185300	0.25458200	1.45768700
H	-9.08231600	-2.54883100	-0.60753700
H	-10.60151300	-4.39154800	0.04236500
H	-13.12841900	-1.58185700	2.08741000
H	-12.63849700	-3.91870400	1.38965200
C	10.02726000	-0.88232300	-0.90440400
C	11.34674600	-2.58278800	-2.69950300
C	9.68256900	-0.88145600	-2.26124300
C	11.03869400	-1.73802600	-0.45282300
C	11.69732600	-2.57496400	-1.34949000
C	10.33406500	-1.73450900	-3.14773900
H	8.90263100	-0.21283800	-2.61295100
H	11.30535700	-1.73847100	0.59970000
H	12.48060500	-3.23303400	-0.98629600
H	10.05603200	-1.72504000	-4.19707700
H	11.85708600	-3.24145800	-3.39435100
C	10.12158800	0.66463000	0.99265200
C	11.62438900	2.00595300	2.94120700
C	9.64878200	0.76074500	2.30678400
C	11.35290000	1.24174200	0.66120300
C	12.10016300	1.89984300	1.63429600
C	10.39410900	1.43603700	3.26931800
H	8.69707400	0.30656700	2.56566600
H	11.71898900	1.16634900	-0.35825300
H	13.05412100	2.34209300	1.36433600
H	10.01547600	1.50423000	4.28444900
H	12.20597700	2.52540800	3.69556800

b1-T_1 Geometry (Solvent: Toluene)

C	3.68406300	0.72269000	0.12280600
H	3.15758000	-1.33164100	-0.37859500
C	2.79991700	-0.33844100	-0.12844200
C	1.75820200	2.19413000	0.43416600
C	1.41761700	-0.17432300	-0.11591000
C	3.11221800	2.01808700	0.40344300
C	0.86659700	1.11636400	0.17754000
C	0.56719700	-1.32989800	-0.40268700
H	3.75429600	2.85958100	0.63576700
H	1.32318400	3.16071900	0.66477300
C	-0.88146000	-1.09202200	-0.35078700
C	-1.76123500	-2.16278000	-0.60075300
C	-1.42104100	0.18156200	-0.05622600
H	-3.19767100	1.32924400	0.24446800
C	-0.56391000	1.34492500	0.21952700
C	-3.12784900	-1.98175300	-0.55900600
H	-1.33043500	-3.13057800	-0.83631000
H	-3.78938900	-2.81432000	-0.78072700
C	-3.68079300	-0.71263500	-0.26068200
C	-2.81655500	0.34602300	-0.01434100
O	1.04609700	-2.44970600	-0.66870800
O	-1.04127900	2.47542700	0.47610100
C	-5.14983200	-0.52340100	-0.20657900
C	-7.94848400	-0.15084400	-0.07609300
C	-5.74280200	0.67067200	-0.64076600
C	-5.99245400	-1.52843700	0.28796900
C	-7.36851700	-1.34673500	0.36302100
C	-7.11857100	0.85504500	-0.58644300
H	-5.11958500	1.45646600	-1.05758400
H	-5.56274200	-2.45430100	0.65954600
H	-8.00114300	-2.12933900	0.77081000
H	-7.55955000	1.78144900	-0.94175000
C	5.11157000	0.52696600	0.09707400
C	7.93591900	0.14429100	0.05035600
C	5.69409800	-0.77645200	0.14066800
C	6.01800500	1.62710200	0.02989300
C	7.37669000	1.45058500	-0.00430900
C	7.04914600	-0.96763500	0.12720800
H	5.05441600	-1.64846400	0.19754600
H	5.63380600	2.63862800	-0.00925700
H	8.03377500	2.31196700	-0.04374500
H	7.45384500	-1.97307500	0.14931600
N	-9.34912300	0.04301300	-0.00205700
N	9.29493000	-0.04114400	0.03287200
C	-9.86635500	1.29111200	0.42214100
C	-10.88899500	3.76642900	1.25940000
C	-9.23984100	2.00504800	1.45159500
C	-11.00936600	1.82744100	-0.18478100
C	-11.51816000	3.05145800	0.24001300
C	-9.74566800	3.23674600	1.85751700
H	-8.35493600	1.59160900	1.92550400
H	-11.49413900	1.27837600	-0.98623700
H	-12.40498100	3.45373000	-0.24013800
H	-9.24692300	3.77879500	2.65506300
H	-11.28384000	4.72402400	1.58233800
C	-10.22809300	-1.02317000	-0.30929900
C	-11.96520500	-3.14112800	-0.91897600
C	-11.37593600	-1.24298800	0.46291100
C	-9.95748400	-1.87325700	-1.38940000
C	-10.81754100	-2.92763000	-1.68281300
C	-12.23918200	-2.28970000	0.15144000
H	-11.58606500	-0.58888400	1.30355500
H	-9.07004300	-1.70291800	-1.99126000
H	-10.59341700	-3.57815400	-2.52267300
H	-13.12490200	-2.44679200	0.75940500
H	-12.63673100	-3.96009700	-1.15460400
C	9.88944300	-1.21154600	0.59576800

C	11.07575300	-3.46931100	1.70075200
C	10.87517600	-1.89444000	-0.12154900
C	9.50295100	-1.64276400	1.86787600
C	10.09723900	-2.77639800	2.41385700
C	11.46399200	-3.02455400	0.43603800
H	11.16640100	-1.54129000	-1.10586200
H	8.75353600	-1.08546100	2.42175400
H	9.80189400	-3.11137500	3.40239800
H	12.22358200	-3.56195900	-0.12161400
H	11.53813400	-4.35124200	2.13102100
C	10.16670000	0.93425100	-0.54049300
C	11.88396900	2.80857000	-1.66447700
C	11.30858100	1.33144500	0.15996500
C	9.88522900	1.45830900	-1.80529400
C	10.74645800	2.39960300	-2.36091400
C	12.16330100	2.27110200	-0.40704300
H	11.51379700	0.90887600	1.13869900
H	9.00700800	1.11863400	-2.34599000
H	10.53306600	2.80447800	-3.34439800
H	13.04654200	2.58782300	0.13732900
H	12.55421100	3.54034800	-2.10269200

b4-S_0 Geometry (Solvent: Toluene)

C	-3.68959600	0.57721000	0.52965500
H	-3.22484100	-1.00710300	-0.87281200
C	-2.83195300	-0.24031900	-0.21214700
C	-1.75227100	1.66740400	1.51025300
C	-1.44953900	-0.10953200	-0.09975800
C	-3.12915000	1.53371800	1.39204700
C	-0.90163000	0.84818500	0.76600700
C	-0.57325100	-1.00981700	-0.90897900
H	-3.78196600	2.19247500	1.95680000
H	-1.31628200	2.41026700	2.16994000
C	0.90161600	-0.84809000	-0.76594200
C	1.75225700	-1.66732200	-1.51017300
C	1.44952500	0.10962900	0.09982200
H	3.22482800	1.00718900	0.87288700
C	0.57323700	1.00992800	0.90902800
C	3.12913600	-1.53364800	-1.39195400
H	1.31626700	-2.41018600	-2.16985900
H	3.78195100	-2.19241500	-1.95669600
C	3.68958200	-0.57713800	-0.52956400
C	2.83194000	0.24040300	0.21222400
O	-1.04838800	-1.84389100	-1.65992600
O	1.04837400	1.84397200	1.66000800
C	5.16182100	-0.43635300	-0.40791900
C	7.94053700	-0.17249400	-0.17808100
C	5.75281900	-0.17622700	0.83579400
C	5.98557200	-0.56065300	-1.53471000
C	7.36608800	-0.42799900	-1.42204300
C	7.13340100	-0.04686400	0.95128200
H	5.13097200	-0.09815800	1.72262200
H	5.54233800	-0.73636800	-2.51042800
H	8.00773500	-0.51358200	-2.29398500
H	7.59664700	0.14512500	1.91453000
C	-5.16183500	0.43641200	0.40802000
C	-7.94054800	0.17251900	0.17819700
C	-5.98557900	0.56069200	1.53481700
C	-5.75283800	0.17629100	-0.83569300
C	-7.13341800	0.04691100	-0.95117300
C	-7.36609500	0.42802000	1.42215700
H	-5.54234100	0.73640300	2.51053300
H	-5.13099600	0.09823800	-1.72252500
H	-7.59666800	-0.14507500	-1.91441900
H	-8.00773800	0.51358700	2.29410500
N	9.35867600	-0.03937700	-0.06083800

H	9.74117100	-4.53600400	0.64614800
C	10.25082900	-3.58226400	0.55110500
H	8.42846500	-2.51100400	0.22301100
C	9.50567500	-2.43584400	0.31125300
C	12.24971700	-2.26325800	0.54162400
C	10.13238500	-1.18235700	0.18252600
C	11.63644200	-3.50495000	0.66841200
C	11.53065400	-1.08855300	0.30028000
H	12.23039400	-4.39302200	0.85568600
H	13.33055400	-2.20411600	0.63437300
C	9.93758200	1.23008500	-0.19512100
C	9.11955800	2.34844400	-0.44103600
C	11.32974300	1.39679500	-0.08703300
H	12.92368300	2.82899700	-0.14719000
C	12.28452400	0.23422300	0.17748100
C	9.66965600	3.61567100	-0.57695600
H	8.04671600	2.22305500	-0.52539100
H	9.01493000	4.46069700	-0.76574800
C	11.04662800	3.79626600	-0.47144700
H	11.48908000	4.78112400	-0.57536000
C	11.84969200	2.68702600	-0.22927700
C	13.05030100	0.50080300	1.49160700
H	13.75882600	-0.30609700	1.69958300
H	12.35391200	0.57357900	2.33190500
H	13.61549500	1.43520100	1.42990700
C	13.29150300	0.13409900	-0.98881800
H	13.86233900	1.06148500	-1.09094400
H	12.76827600	-0.05440200	-1.93065500
H	14.00250000	-0.67974000	-0.82014700
N	-9.35868600	0.03938000	0.06096300
H	-9.01485400	-4.46070400	0.76576500
C	-9.66959100	-3.61569500	0.57693700
H	-8.04669200	-2.22302400	0.52555500
C	-9.11952200	-2.34844600	0.44110100
C	-11.84962600	-2.68711100	0.22908700
C	-9.93756100	-1.23010700	0.19514200
C	-11.04654700	-3.79633100	0.47130100
C	-11.32970600	-1.39686000	0.08692100
H	-11.48897700	-4.78120600	0.57515000
H	-12.92360500	-2.82911500	0.14690300
C	-10.13240600	1.18233300	-0.18249700
C	-9.50572200	2.43583800	-0.31116900
C	-11.53066000	1.08848500	-0.30038500
H	-13.33056400	2.20399200	-0.63464400
C	-12.28450300	-0.23431200	-0.17763900
C	-10.25088900	3.58223500	-0.55109500
H	-8.42852300	2.51103300	-0.22282200
H	-9.74125200	4.53599100	-0.64609300
C	-11.63648800	3.50487800	-0.66853200
H	-12.23045000	4.39293100	-0.85586300
C	-12.24973800	2.26316700	-0.54179700
C	-13.05020000	-0.50090300	-1.49181000
H	-13.61537500	-1.43531300	-1.43014800
H	-12.35376300	-0.57365600	-2.33207000
H	-13.75873400	0.30598000	-1.69982100
C	-13.29154900	-0.13422100	0.98860400
H	-14.00255700	0.67960100	0.81989700
H	-12.76838000	0.05428800	1.93047200
H	-13.86236600	-1.06162400	1.09069200

b4-T_1 Geometry (Solvent: Toluene)

C	3.66203500	-0.55075100	0.57950200
H	3.21043800	0.90427000	-0.95037700
C	2.81825200	0.19736200	-0.22484400
C	1.72281800	-1.54063000	1.65548800
C	1.41757500	0.10304000	-0.11731000

C	3.08988700	-1.43260700	1.53547600
C	0.85453900	-0.78081700	0.84017500
C	0.58865300	0.92472300	-0.99987800
H	3.73688900	-2.04744700	2.15524200
H	1.27513400	-2.22086400	2.37342900
C	-0.85453900	0.78081400	-0.84017900
C	-1.72281800	1.54063300	-1.65548600
C	-1.41757600	-0.10304500	0.11730500
H	-3.21043900	-0.90427500	0.95037200
C	-0.58865400	-0.92473100	0.99987100
C	-3.08988700	1.43261300	-1.53547200
H	-1.27513400	2.22087000	-2.37342400
H	-3.73689000	2.04745800	-2.15523200
C	-3.66203500	0.55075300	-0.57950000
C	-2.81825300	-0.19736500	0.22484000
O	1.09205300	1.70355500	-1.84489200
O	-1.09205300	-1.70355900	1.84488800
C	-5.13214700	0.42784400	-0.45046900
C	-7.90910700	0.17540900	-0.20455200
C	-5.72811500	0.21180200	0.80252900
C	-5.96650300	0.51976100	-1.57565500
C	-7.34736500	0.39550300	-1.45865800
C	-7.10664800	0.08207100	0.92924800
H	-5.10379800	0.16310200	1.68895500
H	-5.52727000	0.66460700	-2.55768500
H	-7.99023300	0.45702900	-2.33202700
H	-7.56535100	-0.08096800	1.90021000
C	5.13214700	-0.42784100	0.45047100
C	7.90910700	-0.17540700	0.20455500
C	5.96650300	-0.51975400	1.57565800
C	5.72811600	-0.21180300	-0.80252700
C	7.10664800	-0.08207200	-0.92924500
C	7.34736400	-0.39549700	1.45866200
H	5.52726900	-0.66459800	2.55768800
H	5.10379900	-0.16310500	-1.68895300
H	7.56535200	0.08096500	-1.90020800
H	7.99023200	-0.45702100	2.33203100
N	-9.33717400	0.04296100	-0.07666000
H	-9.68177900	4.53159900	0.62813100
C	-10.19105600	3.57774700	0.54249700
H	-8.37963700	2.49949200	0.18108400
C	-9.45479700	2.43450200	0.29096700
C	-12.20803100	2.26417700	0.56943500
C	-10.09787700	1.18169800	0.17636800
C	-11.57860300	3.49956400	0.68446600
C	-11.49973400	1.08854600	0.31614800
H	-12.16380700	4.39103800	0.88222400
H	-13.28680300	2.21553500	0.68141700
C	-9.90859700	-1.22037600	-0.20558200
C	-9.07856200	-2.33515400	-0.45999900
C	-11.30492800	-1.38908100	-0.08132800
H	-12.89046600	-2.83126700	-0.12873700
C	-12.24926500	-0.23059000	0.20305600
C	-9.62584600	-3.59809400	-0.59252400
H	-8.00804700	-2.20013500	-0.54952300
H	-8.97451500	-4.44339000	-0.78674100
C	-11.00616500	-3.77873300	-0.47459200
H	-11.44362300	-4.76589500	-0.57663200
C	-11.81959500	-2.67871300	-0.22153800
C	-12.98874000	-0.50307900	1.53409500
H	-13.69194200	0.30394700	1.75535400
H	-12.27718700	-0.57993200	2.36068800
H	-13.55454700	-1.43651100	1.47539400
C	-13.28009300	-0.12679300	-0.94518200
H	-13.84908100	-1.05553300	-1.03696500
H	-12.77795100	0.06799500	-1.89671600
H	-13.98838300	0.68353000	-0.75420000

N	9.33717500	-0.04296000	0.07666300
H	8.97451800	4.44339300	0.78673500
C	9.62584800	3.59809500	0.59251800
H	8.00804900	2.20013700	0.54952300
C	9.07856400	2.33515500	0.45999700
C	11.81959600	2.67871400	0.22153100
C	9.90859800	1.22037700	0.20558100
C	11.00616700	3.77873400	0.47458300
C	11.30492900	1.38908100	0.08132400
H	11.44362600	4.76589700	0.57662100
H	12.89046800	2.83126600	0.12872700
C	10.09787700	-1.18169800	-0.17636300
C	9.45479600	-2.43450200	-0.29095700
C	11.49973400	-1.08854700	-0.31614500
H	13.28680000	-2.21553700	-0.68141600
C	12.24926400	0.23058900	-0.20305900
C	10.19105300	-3.57774800	-0.54248600
H	8.37963600	-2.49949200	-0.18107300
H	9.68177600	-4.53160000	-0.62811600
C	11.57860100	-3.49956600	-0.68445800
H	12.16380400	-4.39104100	-0.88221500
C	12.20802900	-2.26417900	-0.56943100
C	12.98873700	0.50307300	-1.53410100
H	13.55454600	1.43650500	-1.47540300
H	12.27718100	0.57992600	-2.36069200
H	13.69193600	-0.30395400	-1.75536000
C	13.28009600	0.12679600	0.94517600
H	13.98838600	-0.68352800	0.75419400
H	12.77795700	-0.06799100	1.89671200
H	13.84908300	1.05553600	1.03695600

ACRXTN-S_0 Geometry (Solvent: DCM)

H	-3.15573400	1.96366600	0.00000000
C	-2.12835500	1.61453800	0.00000000
C	0.54593000	0.78688100	0.00000000
C	-1.10549900	2.57674000	0.00000000
C	-1.82730400	0.26586600	0.00000000
C	-0.48243300	-0.14012100	0.00000000
C	0.22606700	2.14818400	0.00000000
H	-2.60758600	-0.48823200	0.00000000
H	1.58369100	0.47049500	0.00000000
C	-1.41810600	4.01865900	0.00000000
C	-0.23855400	4.90131800	0.00000000
C	2.01645300	6.54753100	0.00000000
C	-0.38076000	6.29872100	0.00000000
C	1.04902700	4.35440900	0.00000000
C	2.18251700	5.17228300	0.00000000
C	0.73274100	7.11876500	0.00000000
H	-1.38648400	6.70645600	0.00000000
H	3.16490500	4.71313200	0.00000000
H	0.61885500	8.19705300	0.00000000
H	2.89250600	7.18783600	0.00000000
O	1.27592300	3.01043600	0.00000000
O	-2.56716000	4.44411000	0.00000000
N	-0.16813600	-1.53354600	0.00000000
C	-0.06477100	-2.20641900	1.22607800
C	0.14977100	-3.50500700	3.70699300
C	-0.20931700	-1.49194300	2.42979600
C	0.18785600	-3.58930400	1.26176200
C	0.29079600	-4.20533900	2.51331000
C	-0.10353800	-2.13608300	3.65548600
H	-0.40496100	-0.42645200	2.40653600
H	0.48946500	-5.27245500	2.55607800
H	-0.21955100	-1.56094400	4.56871700
H	0.23645400	-4.02007100	4.65764800
C	-0.06477100	-2.20641900	-1.22607800

C	0.14977100	-3.50500700	-3.70699300
C	0.18785600	-3.58930400	-1.26176200
C	-0.20931700	-1.49194300	-2.42979600
C	-0.10353800	-2.13608300	-3.65548600
C	0.29079600	-4.20533900	-2.51331000
H	-0.40496100	-0.42645200	-2.40653600
H	-0.21955100	-1.56094400	-4.56871700
H	0.48946500	-5.27245500	-2.55607800
H	0.23645400	-4.02007100	-4.65764800
C	0.36007600	-4.43294700	0.00000000
C	-0.69333400	-5.56195800	0.00000000
H	-0.58277900	-6.19751100	0.88292800
H	-0.58277900	-6.19751100	-0.88292800
H	-1.70374400	-5.14268400	0.00000000
C	1.77371900	-5.05437500	0.00000000
H	1.92191600	-5.68096500	0.88423500
H	2.53666200	-4.27031400	0.00000000
H	1.92191600	-5.68096500	-0.88423500

ACRXTN-T_1 Geometry (Solvent: DCM)

H	-3.18753400	1.98078900	0.00000000
C	-2.16321200	1.62590900	0.00000000
C	0.51374500	0.77245800	0.00000000
C	-1.12389700	2.60158300	0.00000000
C	-1.88083600	0.26871100	0.00000000
C	-0.55227800	-0.15115700	0.00000000
C	0.21445700	2.11926400	0.00000000
H	-2.67931400	-0.46819500	0.00000000
H	1.54779600	0.44298700	0.00000000
C	-1.40444300	4.00608700	0.00000000
C	-0.22444900	4.88047500	0.00000000
C	2.06203600	6.52700100	0.00000000
C	-0.34080100	6.27955000	0.00000000
C	1.07088900	4.33520000	0.00000000
C	2.20491700	5.13651200	0.00000000
C	0.78721000	7.09657600	0.00000000
H	-1.34191200	6.69890900	0.00000000
H	3.18255700	4.66533600	0.00000000
H	0.67447400	8.17626900	0.00000000
H	2.94598400	7.15647200	0.00000000
O	1.28292700	2.97695300	0.00000000
O	-2.58135100	4.47829500	0.00000000
N	-0.24464500	-1.56355700	0.00000000
C	-0.09798400	-2.21574000	1.21541100
C	0.17772500	-3.46994700	3.68457700
C	-0.25382000	-1.47405800	2.41295400
C	0.20109500	-3.59906200	1.25468300
C	0.33129900	-4.19625600	2.50827800
C	-0.11562700	-2.10070200	3.63290300
H	-0.48156400	-0.41718400	2.36035300
H	0.55832000	-5.25503400	2.57315100
H	-0.23510600	-1.53010200	4.54681000
H	0.28618000	-3.96833100	4.64180100
C	-0.09798400	-2.21574000	-1.21541100
C	0.17772500	-3.46994700	-3.68457700
C	0.20109500	-3.59906200	-1.25468300
C	-0.25382000	-1.47405800	-2.41295400
C	-0.11562700	-2.10070200	-3.63290300
C	0.33129900	-4.19625600	-2.50827800
H	-0.48156400	-0.41718400	-2.36035300
H	-0.23510600	-1.53010200	-4.54681000
H	0.55832000	-5.25503400	-2.57315100
H	0.28618000	-3.96833100	-4.64180100
C	0.37981900	-4.42890400	0.00000000
C	-0.66740600	-5.57043800	0.00000000
H	-0.54262500	-6.20031600	0.88362400

H	-0.54262500	-6.20031600	-0.88362400
H	-1.68181100	-5.16329600	0.00000000
C	1.80593600	-5.03437000	0.00000000
H	1.95303900	-5.65909100	0.88385300
H	2.56080200	-4.24380100	0.00000000
H	1.95303900	-5.65909100	-0.88385300

ACRXTN-S_1 Geometry (Solvent: DCM)

H	-3.19312100	1.97047000	0.00000000
C	-2.16917400	1.61379300	0.00000000
C	0.52554400	0.77302900	0.00000000
C	-1.12155700	2.59775200	0.00000000
C	-1.89004400	0.26979700	0.00000000
C	-0.54748800	-0.16279600	0.00000000
C	0.21743100	2.11614500	0.00000000
H	-2.68756000	-0.46825600	0.00000000
H	1.55980800	0.44738700	0.00000000
C	-1.40848300	3.99351400	0.00000000
C	-0.22106100	4.87659800	0.00000000
C	2.05337800	6.52201100	0.00000000
C	-0.34652600	6.27219700	0.00000000
C	1.06857400	4.32950400	0.00000000
C	2.20365600	5.13694400	0.00000000
C	0.77652600	7.09250900	0.00000000
H	-1.35021100	6.68546200	0.00000000
H	3.18293300	4.66986600	0.00000000
H	0.66317200	8.17168400	0.00000000
H	2.93502800	7.15480900	0.00000000
O	1.28273800	2.97813300	0.00000000
O	-2.56999600	4.47510900	0.00000000
N	-0.24379900	-1.55471900	0.00000000
C	-0.09419500	-2.21315000	1.21571200
C	0.18042500	-3.46233700	3.68649300
C	-0.24548600	-1.46943600	2.41072000
C	0.20133400	-3.59674700	1.25557500
C	0.33019000	-4.19126200	2.50815100
C	-0.10730400	-2.09531200	3.63407800
H	-0.46863000	-0.41222600	2.35480800
H	0.55286400	-5.25093300	2.57472800
H	-0.22311400	-1.52235600	4.54680600
H	0.28843100	-3.96151200	4.64320200
C	-0.09419500	-2.21315000	-1.21571200
C	0.18042500	-3.46233700	-3.68649300
C	0.20133400	-3.59674700	-1.25557500
C	-0.24548600	-1.46943600	-2.41072000
C	-0.10730400	-2.09531200	-3.63407800
C	0.33019000	-4.19126200	-2.50815100
H	-0.46863000	-0.41222600	-2.35480800
H	-0.22311400	-1.52235600	-4.54680600
H	0.55286400	-5.25093300	-2.57472800
H	0.28843100	-3.96151200	-4.64320200
C	0.37498400	-4.42907900	0.00000000
C	-0.68011900	-5.56197400	0.00000000
H	-0.56058300	-6.19278500	0.88384800
H	-0.56058300	-6.19278500	-0.88384800
H	-1.69149500	-5.14717400	0.00000000
C	1.79633200	-5.04260700	0.00000000
H	1.94048400	-5.66814000	0.88385800
H	2.55578900	-4.25636200	0.00000000
H	1.94048400	-5.66814000	-0.88385800

MCz-XT-S_0 Geometry (Solvent: Toluene)

H	-2.34200500	0.00043200	3.24283300
C	-1.85287900	0.00026100	2.27414700
C	-0.67030000	-0.00016400	-0.26128700
C	-2.66674000	0.00010000	1.13077600
C	-0.47576900	0.00021500	2.15966400
C	0.10882700	0.00001900	0.88495800
C	-2.06150400	-0.00013100	-0.13069300
H	0.16676300	0.00032600	3.03381300
H	-0.21431300	-0.00031100	-1.24650300
C	-4.13905100	0.00020800	1.24724600
C	-4.85305800	0.00005100	-0.04334000
C	-6.17913900	-0.00035400	-2.49914000
C	-6.25609800	0.00015100	-0.09079200
C	-4.13679800	-0.00023800	-1.24442800
C	-4.79408600	-0.00045100	-2.47776300
C	-6.91829700	-0.00004300	-1.30495800
H	-6.79289300	0.00038400	0.85245000
H	-4.20514700	-0.00068700	-3.38824100
H	-8.00216100	0.00004000	-1.33756100
H	-6.69510100	-0.00052100	-3.45384400
O	-2.77401400	-0.00033300	-1.28833800
O	-4.71489900	0.00042100	2.32602900
N	1.53236900	0.00002200	0.75130000
C	2.25245300	-1.13673100	0.34310600
C	4.08836400	-3.01756500	-0.60580900
C	1.93860700	-2.49948600	0.50232300
C	3.45281300	-0.72372800	-0.26473800
C	4.37532700	-1.66615500	-0.73365800
C	2.87846300	-3.40028900	0.00889900
H	5.30455300	-1.34221600	-1.19578000
H	2.66975400	-4.46326500	0.11765600
C	2.25242200	1.13676300	0.34304700
C	4.08828400	3.01757900	-0.60604500
C	3.45277400	0.72375500	-0.26482500
C	1.93857600	2.49952400	0.50220600
C	2.87840800	3.40031300	0.00869100
C	4.37527200	1.66616100	-0.73380300
H	2.66968700	4.46329000	0.11741300
H	5.30450700	1.34220100	-1.19589100
C	0.69553500	2.99964000	1.19475700
H	-0.21875000	2.71050400	0.66729600
H	0.72017900	4.08971700	1.25698200
H	0.62094000	2.60341400	2.21215000
C	0.69553100	-2.99953400	1.19486000
H	0.72042500	-4.08956900	1.25769600
H	-0.21868400	-2.71090100	0.66699500
H	0.62057300	-2.60276400	2.21200900
C	5.04366100	-4.07577400	-1.10021400
H	4.57761000	-4.69967500	-1.86950700
H	5.35538500	-4.73763300	-0.28604600
H	5.94075400	-3.62400400	-1.53018700
C	5.04338800	4.07575900	-1.10088100
H	5.94199300	3.62405400	-1.52775000
H	5.35243500	4.74002400	-0.28766600
H	4.57829600	4.69716300	-1.87278400

MCz-XT-T_1 Geometry (Solvent: Toluene)

H	-2.04827700	0.22612400	3.15008400
C	-1.68327400	0.15672500	2.13154100
C	-0.82733200	-0.02945900	-0.54466900
C	-2.65808700	0.09162500	1.08659400
C	-0.32999100	0.13065500	1.85372900
C	0.09724000	0.03607500	0.52582100
C	-2.17357100	0.00078800	-0.24865300
H	0.40655300	0.18031800	2.65230700
H	-0.49721900	-0.09979700	-1.57640200

C	-4.06049600	0.11566300	1.36859700
C	-4.93518200	0.03107900	0.18291000
C	-6.57664200	-0.12674900	-2.09308100
C	-6.33113600	0.03121700	0.30305700
C	-4.38681000	-0.05272500	-1.10493900
C	-5.18849700	-0.13045700	-2.23777500
C	-7.14805500	-0.04667400	-0.82124800
H	-6.74321300	0.09306800	1.30540400
H	-4.71665100	-0.19246100	-3.21299900
H	-8.22778200	-0.04516000	-0.70969700
H	-7.20658000	-0.18683300	-2.97492500
O	-3.02941800	-0.06230200	-1.31616500
O	-4.53513300	0.19923200	2.53055500
N	1.49830600	0.00984400	0.22998200
C	2.25865200	-1.13863300	0.08912500
C	4.21801400	-3.07680700	-0.26759200
C	1.86396200	-2.49056600	0.19891900
C	3.59988800	-0.76008500	-0.18555200
C	4.57572900	-1.71219500	-0.36324800
C	2.89160800	-3.42337300	0.00902700
H	5.60495200	-1.43224600	-0.57089900
H	2.63815900	-4.47721700	0.08325600
C	2.29289300	1.12920400	0.05237600
C	4.30618300	2.99477300	-0.38199200
C	3.62235700	0.70169000	-0.20832800
C	1.93419600	2.49501000	0.09395400
C	2.98846600	3.38977100	-0.12988200
C	4.62473600	1.61759400	-0.42432800
H	2.76343600	4.45244100	-0.10779300
H	5.64433100	1.30021800	-0.62655400
C	0.54821100	3.01522800	0.34766600
H	-0.17360500	2.59510200	-0.35851500
H	0.54354200	4.10248400	0.25657300
H	0.19549300	2.74328900	1.34663100
C	0.46844000	-2.96054200	0.49507600
H	0.45367600	-4.05008000	0.55110600
H	-0.23430100	-2.63661200	-0.27791200
H	0.09827000	-2.55033700	1.43884300
C	5.26703700	-4.13377400	-0.46627200
H	4.86016100	-5.13434100	-0.31270900
H	6.09903000	-3.98671100	0.22934900
H	5.67720400	-4.08019200	-1.48004000
C	5.38659200	4.01287800	-0.61248700
H	5.83974100	3.87539500	-1.59942700
H	6.18361900	3.90158700	0.12955900
H	4.99692800	5.02998600	-0.55031600

MCz-XT-S_1 Geometry (Solvent: Toluene)

H	-2.04520700	0.00010900	3.15839200
C	-1.67711900	0.00005100	2.13841200
C	-0.82386600	-0.00021100	-0.55308500
C	-2.65305100	0.00002400	1.08596300
C	-0.33149200	0.00000100	1.86515500
C	0.10630500	-0.00013000	0.52520500
C	-2.16900300	-0.00012500	-0.25047700
H	0.40281800	0.00003400	2.66664200
H	-0.49610100	-0.00034500	-1.58678700
C	-4.05364200	0.00013500	1.37276400
C	-4.93108700	0.00003800	0.17951800
C	-6.56905300	-0.00019100	-2.09753300
C	-6.32587400	0.00006300	0.30253700
C	-4.37955000	-0.00013000	-1.10740000
C	-5.18364300	-0.00023100	-2.24438000
C	-7.14286000	-0.00004900	-0.82278400
H	-6.73671100	0.00018000	1.30732600
H	-4.71283000	-0.00035300	-3.22178600

H	-8.22242100	-0.00002500	-0.71254600
H	-7.19965100	-0.00027900	-2.98091900
O	-3.02646300	-0.00019800	-1.31870000
O	-4.53320100	0.00019900	2.52799300
N	1.49564300	-0.00004400	0.23425200
C	2.27659700	-1.13367300	0.07191600
C	4.26184100	-3.03573100	-0.33068400
C	1.89994400	-2.49399800	0.15182500
C	3.60802700	-0.73078100	-0.20162700
C	4.59820300	-1.66696800	-0.40239300
C	2.93962900	-3.40659000	-0.05927800
H	5.62087800	-1.36735300	-0.61210500
H	2.70209700	-4.46612900	-0.00876500
C	2.27646900	1.13367800	0.07189600
C	4.26154500	3.03592900	-0.33071100
C	3.60794100	0.73091600	-0.20166100
C	1.89970900	2.49397100	0.15187900
C	2.93932300	3.40665900	-0.05920100
C	4.59802500	1.66719400	-0.40242800
H	2.70170900	4.46617400	-0.00859800
H	5.62072300	1.36766600	-0.61213600
C	0.51049300	2.98716600	0.43741900
H	-0.20687700	2.60827200	-0.29577000
H	0.49799700	4.07804400	0.41618000
H	0.15869200	2.64811500	1.41583100
C	0.51072700	-2.98728100	0.43722400
H	0.49827500	-4.07815800	0.41587900
H	-0.20661200	-2.60834200	-0.29597700
H	0.15884900	-2.64834100	1.41564300
C	5.30888700	-4.09317900	-0.54488400
H	5.05002500	-4.72243000	-1.40194400
H	5.38127200	-4.74692100	0.32945300
H	6.29015300	-3.65161100	-0.72697500
C	5.30840300	4.09346500	-0.54540200
H	6.29052500	3.65205300	-0.72322000
H	5.37772300	4.75034500	0.32680200
H	5.05152100	4.71947500	-1.40545200

3-PXZ-XO-S_0 Geometry (Solvent: Toluene)

H	-1.37139500	-0.00018800	3.02230900
C	-1.09260900	-0.00011900	1.97349900
C	-0.45858500	0.00005200	-0.75362200
C	-2.12551200	-0.00007800	1.02147000
C	0.23037700	-0.00007400	1.57723500
C	0.53848700	0.00000100	0.20482700
C	-1.79462300	0.00002200	-0.33637500
H	1.03823500	-0.00009700	2.30242700
H	-0.21478800	0.00010700	-1.81031400
C	-3.54214000	-0.00016200	1.43987400
C	-4.50736500	-0.00005100	0.32470500
C	-6.31095300	0.00022100	-1.80542600
C	-5.89020700	-0.00005100	0.56732300
C	-4.05429800	0.00008300	-0.99829100
C	-4.95132000	0.00022000	-2.06989700
C	-6.78814600	0.00008500	-0.48450200
H	-6.22166000	-0.00015600	1.60076300
H	-4.56269500	0.00032400	-3.08224100
H	-7.85548300	0.00008700	-0.29325000
H	-7.01267500	0.00033100	-2.63325200
O	-3.88208500	-0.00036400	2.61436000
O	-2.72963900	0.00007700	-1.32216700
N	1.90279300	0.00002300	-0.22298000
C	2.63087300	1.20556200	-0.13191800
C	4.17701700	3.54831800	-0.04204800
C	2.04103100	2.43289100	0.17303100

C	4.00872100	1.17325500	-0.39265500
C	4.77715600	2.32327500	-0.34141500
C	2.81129500	3.59741200	0.21170600
H	0.97609800	2.48132200	0.37196600
H	5.83882300	2.24238500	-0.54963600
H	2.33012700	4.54147900	0.44398500
H	4.77800800	4.45040800	-0.01087400
C	2.63086600	-1.20553800	-0.13200100
C	4.17698900	-3.54830800	-0.04231500
C	4.00871400	-1.17321800	-0.39273600
C	2.04100700	-2.43288700	0.17283200
C	2.81126200	-3.59741500	0.21141600
C	4.77714100	-2.32324800	-0.34157700
H	0.97607100	-2.48133100	0.37174200
H	2.33008800	-4.54150100	0.44360400
H	5.83881100	-2.24235100	-0.54978500
H	4.77797200	-4.45040500	-0.01120100
O	4.63220500	0.00002800	-0.75897000

3-PXZ-XO-T_1 Geometry (Solvent: Toluene)

H	-1.52366800	-1.35498200	2.88346600
C	-1.18091800	-0.91790000	1.95267400
C	-0.38634800	0.24358600	-0.48772200
C	-2.17835600	-0.50178000	1.01476200
C	0.16510800	-0.76489200	1.68465900
C	0.56393600	-0.18607500	0.47385900
C	-1.72466700	0.07677200	-0.20393300
H	0.91654800	-1.08297500	2.40317800
H	-0.08177300	0.68381800	-1.43177600
C	-3.57417300	-0.66156300	1.28705400
C	-4.47509400	-0.18175800	0.22036000
C	-6.16548700	0.74328300	-1.82522200
C	-5.86768300	-0.26662400	0.34667800
C	-3.95524200	0.37732500	-0.95554500
C	-4.78144200	0.83834000	-1.97405000
C	-6.70899800	0.19053000	-0.66334900
H	-6.25797900	-0.70124200	1.26140900
H	-4.33119100	1.26320400	-2.86527200
H	-7.78582700	0.11723500	-0.54846000
H	-6.81462100	1.10190900	-2.61774300
O	-4.02189600	-1.16712700	2.34759500
O	-2.60333800	0.50017300	-1.16459600
N	1.96193500	-0.03692500	0.17252500
C	2.55179600	1.21336800	0.21534600
C	3.82680200	3.70243400	0.27907500
C	1.82669000	2.36679000	0.58127100
C	3.92233400	1.34272600	-0.09937400
C	4.55920200	2.58103600	-0.07014300
C	2.46279700	3.59089400	0.60780400
H	0.77921900	2.26636500	0.83879600
H	5.61347900	2.63035600	-0.31720600
H	1.90238700	4.47484100	0.88996200
H	4.31202600	4.67168200	0.30605000
C	2.72364400	-1.13678300	-0.17990100
C	4.34553600	-3.30511100	-0.88581700
C	4.09640300	-0.96392000	-0.46390100
C	2.16885000	-2.43016300	-0.27570500
C	2.97537300	-3.49378900	-0.62340900
C	4.90663000	-2.04224500	-0.81094400
H	1.11222400	-2.56229100	-0.07598200
H	2.54401900	-4.48550100	-0.69725500
H	5.95495500	-1.86024500	-1.01833900
H	4.96630200	-4.15204300	-1.15588000
O	4.67275900	0.26213500	-0.42422300

3-PXZ-XO-S_1 Geometry (Solvent: Toluene)

H	-1.52236300	0.00140400	3.17533700
C	-1.17692000	0.00100100	2.14768800
C	-0.38369000	-0.00011500	-0.56316100
C	-2.17584900	0.00049900	1.11646400
C	0.16267000	0.00095100	1.84567900
C	0.56934300	0.00040500	0.49568000
C	-1.72215400	-0.00002200	-0.23005500
H	0.91309600	0.00128300	2.63173600
H	-0.08013700	-0.00057600	-1.60391900
C	-3.57010800	0.00053200	1.43374800
C	-4.47341700	0.00014800	0.25989200
C	-6.16033100	-0.00061400	-1.98116600
C	-5.86517400	0.00024300	0.41317000
C	-3.95021600	-0.00032400	-1.03867700
C	-4.77854300	-0.00070600	-2.15806700
C	-6.70633800	-0.00014700	-0.69416300
H	-6.25385900	0.00059900	1.42664000
H	-4.32905000	-0.00105800	-3.14545300
H	-7.78325700	-0.00007800	-0.56062700
H	-6.80999600	-0.00090600	-2.85062400
O	-4.02427600	0.00140700	2.59903600
O	-2.60201000	-0.00042700	-1.27901800
N	1.95772600	0.00010500	0.16784200
C	2.63647300	1.19407600	0.00163500
C	4.08138900	3.56192500	-0.33310100
C	1.98930100	2.43801000	0.14189000
C	4.01462800	1.17122600	-0.31144200
C	4.73581500	2.34822400	-0.47627700
C	2.70972200	3.60353400	-0.02593700
H	0.93303900	2.45133600	0.37993800
H	5.79168900	2.28438000	-0.71242800
H	2.20947100	4.55876600	0.08141100
H	4.63541900	4.48513500	-0.45968700
C	2.63607100	-1.19416900	0.00215000
C	4.08015200	-3.56264400	-0.33177100
C	4.01422900	-1.17191500	-0.31097200
C	1.98846700	-2.43783100	0.14286900
C	2.70847700	-3.60366600	-0.02456000
C	4.73500100	-2.34922500	-0.47539200
H	0.93220300	-2.45070500	0.38092800
H	2.20789500	-4.55868600	0.08312600
H	5.79089000	-2.28583600	-0.71159800
H	4.63385400	-4.48609200	-0.45805500
O	4.68041600	-0.00048800	-0.45856400

PTZ-XT-S_0 Geometry (Solvent: THF)

H	1.71974800	-0.00148200	3.25948600
C	1.35686300	-0.00098200	2.23692500
C	0.49269600	0.00030100	-0.42390000
C	2.30364100	-0.00051100	1.20256300
C	0.00217900	-0.00080100	1.95471100
C	-0.42294000	-0.00014700	0.61864500
C	1.85647900	0.00013100	-0.12390300
H	-0.74196000	-0.00115700	2.74358400
H	0.16663000	0.00076800	-1.45949200
C	3.75046900	-0.00068300	1.49512200
C	4.61645000	-0.00014900	0.30320900
C	6.23166700	0.00086500	-1.97378300
C	6.01555800	-0.00024300	0.42640000
C	4.05186000	0.00046600	-0.97671200
C	4.85422600	0.00097300	-2.12113200
C	6.82039000	0.00025900	-0.69813400
H	6.43668500	-0.00071600	1.42662700
H	4.38178800	0.00143900	-3.09720300
H	7.90012700	0.00018500	-0.59895500

H	6.85998700	0.00125500	-2.85848600
O	2.70465100	0.00060800	-1.18569700
O	4.19119800	-0.00125800	2.63785200
N	-1.83092200	-0.00003400	0.35082200
C	-2.42652800	-1.23193900	-0.02060000
C	-3.66436300	-3.66104600	-0.74314100
C	-3.82651800	-1.34037500	-0.07939500
C	-1.66221000	-2.37061900	-0.30628800
C	-2.27887800	-3.57317000	-0.65043400
C	-4.43410200	-2.53327200	-0.46437200
H	-1.66017300	-4.44093000	-0.85453400
H	-5.51782800	-2.57649000	-0.51893200
H	-4.14445300	-4.59247100	-1.02257300
C	-2.42651200	1.23201700	-0.02013000
C	-3.66436800	3.66139300	-0.74175200
C	-3.82650500	1.34046000	-0.07891400
C	-1.66220900	2.37083400	-0.30530900
C	-2.27888600	3.57350800	-0.64900600
C	-4.43409500	2.53349600	-0.46344900
H	-0.58160000	2.33008500	-0.24801900
H	-1.66018800	4.44136300	-0.85272400
H	-5.51782000	2.57671800	-0.51802200
H	-4.14446300	4.59291700	-1.02084400
H	-0.58159700	-2.32985600	-0.24905200
S	-4.83321200	-0.00005000	0.46330100

PTZ-XT-T_1 Geometry (Solvent: THF)

H	1.67960600	-0.14920100	3.17073300
C	1.33897800	-0.10523400	2.14253300
C	0.52554400	0.00746000	-0.54404700
C	2.33027100	-0.06572700	1.11845200
C	-0.01369200	-0.08591500	1.83940300
C	-0.41536100	-0.02933300	0.50640000
C	1.86797400	-0.01038800	-0.22587100
H	-0.76275300	-0.11481900	2.62632900
H	0.21207600	0.05022100	-1.58235700
C	3.73048100	-0.07973500	1.41982000
C	4.62214000	-0.02140200	0.25396100
C	6.30262600	0.08565800	-2.00489700
C	6.01910000	-0.01307300	0.39185600
C	4.09631100	0.02825300	-1.04829500
C	4.91455900	0.08039300	-2.16896200
C	6.85295600	0.03975200	-0.72243800
H	6.42236700	-0.04878500	1.39888100
H	4.45796700	0.11598400	-3.15286100
H	7.93080200	0.04500900	-0.59325700
H	6.94528700	0.12592400	-2.87838600
O	2.74161000	0.02689700	-1.28081100
O	4.18554900	-0.13498000	2.60153300
N	-1.82424100	-0.00602700	0.17981800
C	-2.46626900	-1.22397500	0.00537500
C	-3.72074000	-3.73205300	-0.31874400
C	-3.84869400	-1.32051200	-0.28866200
C	-1.72830000	-2.42619500	0.12715800
C	-2.34532000	-3.64754200	-0.03278000
C	-4.46168100	-2.57725600	-0.44530000
H	-1.75662500	-4.55280100	0.06495100
H	-5.52335300	-2.61997300	-0.66719900
H	-4.19721900	-4.69808800	-0.44023300
C	-2.43599500	1.23325900	0.05253500
C	-3.62495100	3.78239900	-0.18743700
C	-3.81393100	1.37538300	-0.24422900
C	-1.66997100	2.41120800	0.22722300
C	-2.25511500	3.65275200	0.10812500
C	-4.39377100	2.65206300	-0.36048100
H	-0.61591500	2.32587300	0.45613800

H	-1.64536300	4.53867700	0.24566300
H	-5.45235100	2.72922300	-0.58790800
H	-4.07570100	4.76400500	-0.27839400
H	-0.67037700	-2.37541300	0.34848300
S	-4.87743200	0.04434800	-0.47245400

PTZ-XT-S_1 Geometry (Solvent: THF)

H	1.65552200	-0.00047900	3.17542100
C	1.31809400	-0.00022600	2.14517100
C	0.52875700	0.00040300	-0.56351000
C	2.32131900	-0.00014500	1.11702900
C	-0.02162400	-0.00002100	1.83781600
C	-0.42611800	0.00024300	0.48980200
C	1.86704800	0.00021300	-0.23039400
H	-0.77540600	-0.00014300	2.62064000
H	0.22366700	0.00060000	-1.60416900
C	3.71309600	-0.00042100	1.43078800
C	4.61781800	-0.00007400	0.26112500
C	6.30649100	0.00060000	-1.98248000
C	6.01118500	-0.00009900	0.41261600
C	4.09585500	0.00030500	-1.03918800
C	4.92423300	0.00064100	-2.15866300
C	6.85231800	0.00023400	-0.69491100
H	6.40518500	-0.00037300	1.42393100
H	4.47540100	0.00092300	-3.14643400
H	7.92918300	0.00021500	-0.56114300
H	6.95589900	0.00086200	-2.85195200
O	2.74850100	0.00033700	-1.27889200
O	4.17034200	-0.00085900	2.60188400
N	-1.81681800	0.00007700	0.15928500
C	-2.44699400	-1.23061800	0.00592000
C	-3.66097400	-3.75993100	-0.25525300
C	-3.83508900	-1.35061400	-0.25444200
C	-1.68466800	-2.41679300	0.12612100
C	-2.28243400	-3.65257200	-0.00481400
C	-4.42664400	-2.61734400	-0.37841700
H	-1.67661900	-4.54650000	0.08869300
H	-5.49254000	-2.68343100	-0.57191800
H	-4.12610400	-4.73421200	-0.35263500
C	-2.44728000	1.23063200	0.00612500
C	-3.66179000	3.75971400	-0.25464700
C	-3.83538800	1.35036000	-0.25426100
C	-1.68521400	2.41693800	0.12659100
C	-2.28323900	3.65261200	-0.00414500
C	-4.42721200	2.61698400	-0.37804400
H	-0.62416400	2.34247500	0.32263900
H	-1.67762700	4.54665500	0.08957000
H	-5.49311600	2.68287400	-0.57157100
H	-4.12712600	4.73391100	-0.35188200
H	-0.62362100	-2.34216000	0.32210500
S	-4.89067700	-0.00023200	-0.42286500

MC2-S_0 Geometry (Solvent: Toluene)

H	-5.13939400	-2.66373900	-0.49887100
C	-5.11656900	-1.59412900	-0.31621400
C	-5.02872000	1.14941100	0.20453900
C	-3.91492800	-1.00864600	0.11679600
C	-6.24736500	-0.82500400	-0.51083800
C	-6.20956300	0.55458800	-0.25004500
C	-3.89012200	0.36296600	0.37640900
H	-7.17783300	-1.26058100	-0.85891800
H	-2.98261800	0.83124800	0.74505300
H	-4.98374400	2.20765000	0.42958100
C	-2.74225300	-1.89573000	0.35544000

O	-2.90077900	-3.07848300	0.61894000
C	-1.35324400	-1.33811500	0.27123000
C	1.29134300	-0.45421000	0.10238800
C	-0.36273500	-1.91223800	1.07592900
C	-1.00389000	-0.32602600	-0.62924700
C	0.31568900	0.10383100	-0.72721400
C	0.94872800	-1.46138400	1.01113000
H	-0.64056200	-2.70598400	1.76197100
H	-1.75486600	0.10132500	-1.28645100
H	0.59996700	0.85806500	-1.45417700
H	1.71185900	-1.87563800	1.66248800
N	2.62862700	-0.00472600	0.01918000
C	3.75027600	-0.81569600	-0.15223300
C	6.24470000	-1.96447500	-0.51987800
C	3.82394000	-2.20045400	-0.32512300
C	4.90406500	-0.00179000	-0.18165100
C	6.16060400	-0.58657800	-0.36427200
C	5.08478900	-2.75776800	-0.50493600
H	2.93367400	-2.82015400	-0.32797200
H	7.05488000	0.02916200	-0.38864000
H	5.17165100	-3.83115300	-0.64040700
H	7.21211900	-2.43469600	-0.66118100
C	3.04834500	1.32225500	0.10112100
C	4.37448100	3.75140300	0.24701400
C	4.45520900	1.36627000	-0.01755500
C	2.29353700	2.47923000	0.31188700
C	2.97696600	3.68814700	0.37929400
C	5.11895500	2.59437100	0.05383000
H	1.21567700	2.43928200	0.42695000
H	2.41541500	4.60281600	0.54116700
H	6.20029800	2.64034800	-0.03593300
H	4.87400700	4.71278700	0.30335700
O	-7.36467500	1.22467200	-0.46386100
C	-7.38329500	2.62049500	-0.21275600
H	-8.39199600	2.95349400	-0.45078800
H	-7.16554500	2.83217400	0.83954100
H	-6.66340300	3.14284000	-0.85192400

MC2-T_1 Geometry (Solvent: Toluene)

H	-4.99030800	-2.66506100	-0.43355100
C	-5.03454900	-1.59009600	-0.28905100
C	-5.14137100	1.16238900	0.15049700
C	-3.90734100	-0.93605200	0.23420200
C	-6.18240600	-0.88699300	-0.61140200
C	-6.24130700	0.49689200	-0.39536500
C	-3.98692600	0.43915000	0.45778800
H	-7.05314100	-1.38388500	-1.02664200
H	-3.14806700	0.96396300	0.90754900
H	-5.17135800	2.22655900	0.35085300
C	-2.71905700	-1.75549700	0.61406700
O	-2.89704200	-2.91443500	1.07320600
C	-1.37956600	-1.27144000	0.43141900
C	1.33083100	-0.43584600	0.17189800
C	-0.28219900	-2.04496400	1.01419300
C	-1.02227500	-0.10912300	-0.37167900
C	0.26631200	0.29957000	-0.47549500
C	1.00510500	-1.63883300	0.89281100
H	-0.54420500	-2.92630400	1.58631800
H	-1.79423900	0.40211400	-0.93509800
H	0.52891100	1.13028600	-1.12380000
H	1.80091600	-2.17750800	1.39801800
N	2.63965000	-0.01427600	0.04502200
C	3.75523400	-0.82999700	-0.21502200
C	6.20599000	-1.97639500	-0.77616100
C	3.79588500	-2.19744200	-0.48872400
C	4.91137200	-0.02442700	-0.26920900

C	6.14661500	-0.60255800	-0.54728900
C	5.04271600	-2.75780400	-0.75596000
H	2.89707900	-2.80229500	-0.51608900
H	7.04369900	0.00671700	-0.59677000
H	5.10866100	-3.82018700	-0.96540900
H	7.16034900	-2.44473900	-0.99197400
C	3.08679200	1.31342000	0.15366200
C	4.44077400	3.71361300	0.34128700
C	4.48307200	1.34757600	-0.03850200
C	2.35927200	2.45519100	0.49017700
C	3.05842700	3.65757600	0.56551600
C	5.16476400	2.55772600	0.05183100
H	1.29793000	2.41205100	0.70632900
H	2.52088800	4.56548700	0.81794600
H	6.24043400	2.59862400	-0.08870400
H	4.95520000	4.66589700	0.41375000
O	-7.40924500	1.10206700	-0.73873400
C	-7.51978900	2.49572200	-0.51730700
H	-8.51596400	2.77649900	-0.85602600
H	-7.41399500	2.73513800	0.54679300
H	-6.76831300	3.04738000	-1.09352600

MC2-S_1 Geometry (Solvent: Toluene)

H	-3.37739200	-1.49013500	-1.42672500
C	-4.11789100	-0.90122000	-0.89451900
C	-6.04190700	0.58084400	0.51548800
C	-3.92496400	-0.61055700	0.47217700
C	-5.21926300	-0.40219400	-1.55896700
C	-6.18953800	0.34142700	-0.85783800
C	-4.91947400	0.09059200	1.17120400
H	-5.36876000	-0.57524800	-2.61924900
H	-4.79145000	0.26723900	2.23413400
H	-6.78453900	1.14011700	1.07040500
C	-2.72729200	-1.11566600	1.20109400
O	-3.13641200	-1.97066900	2.07725500
C	-1.35839100	-0.83069300	0.90923500
C	1.34415400	-0.27259500	0.36851500
C	-0.32588600	-1.50693500	1.60393300
C	-0.99978600	0.13206900	-0.06406400
C	0.33269600	0.39675500	-0.33248700
C	1.00362500	-1.21909500	1.34117600
H	-0.58610800	-2.24446200	2.35622900
H	-1.77422200	0.66569800	-0.60676800
H	0.60533100	1.12444600	-1.09143200
H	1.79371400	-1.72325000	1.89043800
N	2.70609500	0.00895900	0.09245900
C	3.65985400	-0.91698600	-0.31499900
C	5.87796200	-2.35000700	-1.15174900
C	3.51023000	-2.28393100	-0.56565100
C	4.89863400	-0.25895800	-0.49093400
C	6.01500900	-0.98826800	-0.91140400
C	4.63517000	-2.98564000	-0.98274300
H	2.55134300	-2.77680700	-0.44319700
H	6.97309300	-0.49575900	-1.05031800
H	4.54960700	-4.04886900	-1.18420500
H	6.73506500	-2.93029900	-1.47704100
C	3.31183100	1.25721100	0.18190100
C	4.95371600	3.48798600	0.23096300
C	4.67473600	1.13708900	-0.17397600
C	2.75924200	2.47869900	0.57724600
C	3.59871500	3.58642800	0.59347100
C	5.49768900	2.26722500	-0.14931900
H	1.71614600	2.55694900	0.86579200
H	3.19753000	4.54862800	0.89618900
H	6.54666800	2.18952000	-0.42023300
H	5.57886600	4.37447200	0.25410900

O	-7.22890600	0.77568500	-1.59331200
C	-8.25292700	1.51857000	-0.94562100
H	-8.97845900	1.76029800	-1.71971500
H	-8.73201300	0.92041900	-0.16413500
H	-7.85021000	2.44130300	-0.51585200

OPM-S_0 Geometry (Solvent: Toluene)

H	6.10262100	-1.72721100	-1.30077400
C	6.03757700	-0.76920800	-0.79473600
C	5.85017400	1.65421400	0.56345600
C	4.84594900	-0.44677400	-0.12919000
C	7.11048900	0.11670600	-0.80140700
C	7.02037800	1.33514200	-0.12578100
C	4.77226400	0.77157000	0.56196200
H	8.02176900	-0.14406700	-1.33129000
H	7.85862400	2.02491100	-0.12707500
H	3.87850900	1.01846800	1.12875400
H	5.78021900	2.58798400	1.11339600
C	3.74892300	-1.45572900	-0.11613700
C	2.36578200	-1.07527900	-0.11867900
C	-0.41395500	-0.44623200	0.09881100
C	1.35895600	-2.11444600	0.06737200
C	1.86675200	0.25788400	-0.41833800
C	0.54691300	0.55489200	-0.29739700
C	0.04098100	-1.80725400	0.18983600
H	1.70328300	-3.13926900	0.12366500
H	2.55379800	1.01563600	-0.77626700
H	0.19719200	1.55098500	-0.54961300
H	-0.67651400	-2.60519600	0.33874300
O	4.04207500	-2.68788900	-0.14959300
N	-1.74036100	-0.10178200	0.27671700
C	-2.18486000	1.25180400	0.17238300
C	-3.06919000	3.85743600	-0.17369400
C	-1.75242900	2.23393500	1.07720500
C	-3.10963800	1.54991600	-0.83655100
C	-3.55080000	2.85961300	-1.01593600
C	-2.19900200	3.54125600	0.86877100
H	-4.27173700	3.08476600	-1.79495600
H	-1.87023300	4.31973800	1.55106800
H	-3.40365100	4.88087000	-0.30730600
C	-2.78821200	-1.06681000	0.35521300
C	-4.85881700	-2.92072300	0.39552400
C	-3.78887900	-1.01484200	-0.62255200
C	-2.85415200	-2.00519000	1.38495400
C	-3.88379300	-2.94145200	1.39440800
C	-4.82539800	-1.94882800	-0.60086300
H	-2.09610900	-1.99726300	2.16230000
H	-3.92948200	-3.68052200	2.18680800
H	-5.60357500	-1.90238700	-1.35598900
H	-5.66362800	-3.64799400	0.40676300
S	-3.71761700	0.24347500	-1.85797900
C	-0.85218500	1.90042100	2.23823500
H	-0.99357600	2.62804800	3.03973000
H	0.20362100	1.91804800	1.94868500
H	-1.06630500	0.90230000	2.62954100

OPM-T_1 Geometry (Solvent: Toluene)

H	6.10262100	-1.72721100	-1.30077400
C	6.03757700	-0.76920800	-0.79473600
C	5.85017400	1.65421400	0.56345600
C	4.84594900	-0.44677400	-0.12919000
C	7.11048900	0.11670600	-0.80140700
C	7.02037800	1.33514200	-0.12578100

C	4.77226400	0.77157000	0.56196200
H	8.02176900	-0.14406700	-1.33129000
H	7.85862400	2.02491100	-0.12707500
H	3.87850900	1.01846800	1.12875400
H	5.78021900	2.58798400	1.11339600
C	3.74892300	-1.45572900	-0.11613700
C	2.36578200	-1.07527900	-0.11867900
C	-0.41395500	-0.44623200	0.09881100
C	1.35895600	-2.11444600	0.06737200
C	1.86675200	0.25788400	-0.41833800
C	0.54691300	0.55489200	-0.29739700
C	0.04098100	-1.80725400	0.18983600
H	1.70328300	-3.13926900	0.12366500
H	2.55379800	1.01563600	-0.77626700
H	0.19719200	1.55098500	-0.54961300
H	-0.67651400	-2.60519600	0.33874300
O	4.04207500	-2.68788900	-0.14959300
N	-1.74036100	-0.10178200	0.27671700
C	-2.18486000	1.25180400	0.17238300
C	-3.06919000	3.85743600	-0.17369400
C	-1.75242900	2.23393500	1.07720500
C	-3.10963800	1.54991600	-0.83655100
C	-3.55080000	2.85961300	-1.01593600
C	-2.19900200	3.54125600	0.86877100
H	-4.27173700	3.08476600	-1.79495600
H	-1.87023300	4.31973800	1.55106800
H	-3.40365100	4.88087000	-0.30730600
C	-2.78821200	-1.06681000	0.35521300
C	-4.85881700	-2.92072300	0.39552400
C	-3.78887900	-1.01484200	-0.62255200
C	-2.85415200	-2.00519000	1.38495400
C	-3.88379300	-2.94145200	1.39440800
C	-4.82539800	-1.94882800	-0.60086300
H	-2.09610900	-1.99726300	2.16230000
H	-3.92948200	-3.68052200	2.18680800
H	-5.60357500	-1.90238700	-1.35598900
H	-5.66362800	-3.64799400	0.40676300
S	-3.71761700	0.24347500	-1.85797900
C	-0.85218500	1.90042100	2.23823500
H	-0.99357600	2.62804800	3.03973000
H	0.20362100	1.91804800	1.94868500
H	-1.06630500	0.90230000	2.62954100

OPM-S_1 Geometry (Solvent: Toluene)

H	6.35439200	-0.30802600	-1.97470700
C	6.21985400	-0.16150900	-0.90759500
C	5.85213900	0.21409800	1.83639200
C	4.90438200	-0.09399000	-0.38365600
C	7.31576300	-0.04163700	-0.06570500
C	7.14830900	0.14685200	1.31091600
C	4.74384000	0.09660300	1.01132500
H	8.31661600	-0.09550900	-0.48466900
H	8.01061700	0.23932000	1.96238500
H	3.74255900	0.14998000	1.43064600
H	5.70848100	0.35973200	2.90322800
C	3.77461400	-0.21494600	-1.24203700
C	2.34005500	-0.18060200	-0.85468300
C	-0.36910900	-0.10583800	-0.07328800
C	1.65640100	-1.36593900	-0.51149400
C	1.60162800	1.01864200	-0.93212900
C	0.28447900	1.06776600	-0.52868000
C	0.34066700	-1.33367300	-0.10136600
H	2.19225900	-2.30951400	-0.53918800
H	2.09338000	1.91753100	-1.29047900
H	-0.25929400	2.00352600	-0.58085600
H	-0.15115600	-2.25314400	0.18855600

O	3.77522500	-0.39559700	-2.51885800
N	-1.67653200	-0.05092600	0.37067200
C	-2.44803300	1.14849400	0.25204900
C	-3.96066400	3.44396300	-0.10780400
C	-2.22751000	2.23881800	1.10463100
C	-3.46005800	1.17546900	-0.71384300
C	-4.22017400	2.32915700	-0.90004800
C	-2.98957600	3.39089900	0.89114700
H	-5.01029000	2.34331500	-1.64368100
H	-2.82597400	4.25339200	1.53060400
H	-4.54316300	4.34869600	-0.24746800
C	-2.46024000	-1.23580000	0.53197400
C	-4.00485300	-3.53730100	0.73632900
C	-3.48064600	-1.47407800	-0.39468100
C	-2.24868600	-2.11632500	1.59059700
C	-3.01211800	-3.27716900	1.68267800
C	-4.25547400	-2.63046200	-0.29037700
H	-1.47556900	-1.89195500	2.31969100
H	-2.83793900	-3.97317900	2.49609100
H	-5.05382000	-2.80812500	-1.00393700
H	-4.60428100	-4.43842200	0.81164900
S	-3.77461500	-0.28187600	-1.66495600
C	-1.20022200	2.17107500	2.20277600
H	-1.40647200	2.92735800	2.96243600
H	-0.18930600	2.34550900	1.81837900
H	-1.20187300	1.18538900	2.67619600

***p*-Cz-S$_0$ Geometry (Solvent: THF)**

H	-3.53660500	1.43820300	0.75014900
C	-4.46463900	1.07681800	0.31766400
C	-6.87113100	0.14813000	-0.75410000
C	-4.63040500	-0.28649500	0.04721700
C	-5.50400900	1.96926400	0.06125100
C	-6.70229200	1.50764400	-0.48250200
C	-5.84334900	-0.74756000	-0.47838700
H	-5.37776600	3.02325600	0.28599600
H	-5.96301500	-1.80927800	-0.66993900
H	-7.80431700	-0.21080500	-1.17583200
C	-3.57135900	-1.29388000	0.36663300
O	-3.88225500	-2.42871800	0.69731200
C	-2.12778300	-0.91126900	0.27822000
C	0.60110200	-0.34547200	0.10669800
C	-1.66191000	0.04401800	-0.63215500
C	-1.21262700	-1.58830900	1.09318700
C	0.14252000	-1.29591800	1.02598800
C	-0.30116800	0.31474700	-0.73129900
H	-2.35562900	0.55259300	-1.29403000
H	-1.58072100	-2.33225100	1.79224800
H	0.84945400	-1.78872400	1.68575200
H	0.06798500	1.02475200	-1.46433100
N	1.98142300	-0.05440100	0.02380700
C	3.00385000	-0.99153800	-0.12452900
C	5.35386600	-2.42387000	-0.45499600
C	4.24336500	-0.31504100	-0.16360200
C	2.92047700	-2.37931300	-0.26940100
C	4.11080000	-3.07986500	-0.43085800
C	5.42658900	-1.04203000	-0.32731800
H	1.96632200	-2.89508700	-0.26352800
H	4.07541800	-4.15877700	-0.54374700
H	6.38513100	-0.53243000	-0.35868000
H	6.26204900	-3.00381100	-0.58109700
C	2.54983900	1.21787000	0.08222900
C	4.14567400	3.48202500	0.19004400
C	1.93158800	2.45773200	0.26735900
C	3.95304700	1.09857500	-0.02909800

C	4.75379500	2.24374200	0.02269300
C	2.74916700	3.58159400	0.31582800
H	0.85599800	2.54559500	0.37610300
H	5.83354400	2.16362100	-0.06184600
H	2.29521400	4.55730900	0.45723400
H	4.75171600	4.38094100	0.23098000
H	-7.50673800	2.20585100	-0.69117600

p-Cz-T_1 Geometry (Solvent: THF)

H	-3.68271300	1.58267000	0.70176900
C	-4.55816900	1.13509100	0.23892700
C	-6.84769000	0.00027000	-0.87178000
C	-4.62548000	-0.25553000	0.07326700
C	-5.62010400	1.94867800	-0.15202900
C	-6.76484300	1.38547000	-0.71697400
C	-5.79139300	-0.81284100	-0.47035800
H	-5.55705400	3.02277900	-0.00647600
H	-5.85403900	-1.89195600	-0.57167600
H	-7.73883800	-0.44586200	-1.30284000
C	-3.54360000	-1.17643100	0.53486700
O	-3.86776000	-2.28457100	1.05298100
C	-2.15478300	-0.84799100	0.37465300
C	0.63483500	-0.32985700	0.14722900
C	-1.65548000	0.22876700	-0.45826100
C	-1.16737000	-1.69960000	1.02084300
C	0.16467200	-1.44648600	0.91328700
C	-0.32299600	0.48744800	-0.55093000
H	-2.35041600	0.80688200	-1.05592100
H	-1.53077600	-2.51845500	1.62908800
H	0.88204000	-2.04525100	1.46601500
H	0.03894500	1.25788000	-1.22503300
N	1.98581800	-0.05870000	0.04183600
C	3.01029700	-1.00363200	-0.14391900
C	5.33019500	-2.43790600	-0.56943700
C	4.24974000	-0.33233600	-0.19789700
C	2.90409500	-2.37881100	-0.35280100
C	4.08851200	-3.08592600	-0.55153300
C	5.41930000	-1.05375400	-0.40751600
H	1.94484200	-2.88190500	-0.38242900
H	4.04201900	-4.15813600	-0.70849400
H	6.38026300	-0.55161700	-0.45644500
H	6.23247400	-3.01783900	-0.73123700
C	2.57189300	1.21698000	0.10447300
C	4.17263500	3.46150100	0.22646400
C	1.96344300	2.44435500	0.36785100
C	3.96850200	1.08991700	-0.04460300
C	4.77567200	2.22006500	0.01240900
C	2.78803700	3.56725000	0.41000100
H	0.89900900	2.52749600	0.55395900
H	5.85261400	2.13918500	-0.09500400
H	2.34665400	4.53887500	0.60396300
H	4.78666200	4.35454700	0.27181000
H	-7.58988000	2.02003300	-1.02515200

p-Cz-S_1 Geometry (Solvent: THF)

H	-3.57535400	1.58598700	0.36018300
C	-4.52406000	1.14185000	0.07794500
C	-7.00595100	0.01987000	-0.54527700
C	-4.66981300	-0.25855600	0.05085100
C	-5.60638700	1.95880800	-0.22611900
C	-6.85081000	1.40611500	-0.54167600
C	-5.92941600	-0.81143500	-0.25243300
H	-5.48046400	3.03679100	-0.20220800
H	-6.05009700	-1.88969100	-0.27327000
H	-7.96991500	-0.41947900	-0.78224100

C	-3.55434200	-1.14547600	0.38233000
O	-3.83149600	-2.34819800	0.77294300
C	-2.12383600	-0.83498100	0.28875200
C	0.62689800	-0.32254500	0.11019300
C	-1.63018800	0.08944800	-0.65253600
C	-1.21010500	-1.52868600	1.10797700
C	0.14983700	-1.26543000	1.02543900
C	-0.27037500	0.34828100	-0.73137500
H	-2.30848300	0.58210000	-1.34069900
H	-1.57907300	-2.24391200	1.83546300
H	0.84680000	-1.77290600	1.68520500
H	0.10888500	1.05077400	-1.46725900
N	2.01055700	-0.05048900	0.02294900
C	3.01770700	-0.99903900	-0.14495500
C	5.34200100	-2.46428300	-0.50338500
C	4.26846000	-0.34195700	-0.17377600
C	2.91033600	-2.38270200	-0.31231600
C	4.08848300	-3.10028400	-0.48790300
C	5.43853700	-1.08567100	-0.35216600
H	1.94674300	-2.88081000	-0.31236800
H	4.03550300	-4.17643100	-0.61878400
H	6.40585800	-0.59255100	-0.37647000
H	6.24028900	-3.05686800	-0.64109400
C	2.59855200	1.21069800	0.10602900
C	4.22455200	3.44987200	0.24986300
C	1.99715600	2.45475900	0.31642800
C	3.99988300	1.07362700	-0.01251300
C	4.81589000	2.20651400	0.05749700
C	2.83000600	3.56649100	0.38251700
H	0.92291300	2.55324000	0.43047100
H	5.89425400	2.11421300	-0.03202600
H	2.39028100	4.54563600	0.54373500
H	4.84300100	4.33952500	0.30529300
H	-7.69116200	2.05166600	-0.77445300

ODFRCZ-S_0 Geometry (Solvent: DCM)

H	-2.19583300	-3.15258400	-0.88737600
C	-2.23235500	-2.07611800	-1.02136800
C	-2.29968900	0.67850600	-1.41117400
C	-1.03952100	-1.37921000	-1.24698700
C	-3.44405500	-1.40060100	-0.96808800
C	-3.49424900	-0.01222400	-1.16873900
C	-1.08267900	0.00551200	-1.44282600
H	-4.36099200	-1.95072700	-0.78608500
H	-0.17018400	0.56005800	-1.63840700
H	-2.32051600	1.75386400	-1.55870200
C	0.23278300	-2.15832500	-1.34041600
C	1.52682300	-1.51994000	-0.94626000
C	4.00157800	-0.46093300	-0.20467000
C	2.69941400	-1.95654100	-1.57416900
C	1.60641400	-0.55640900	0.06554400
C	2.84005800	-0.04095900	0.44878600
C	3.92962200	-1.41763800	-1.22361600
H	2.63094500	-2.70818800	-2.35393500
H	0.70960400	-0.23280500	0.58438700
H	2.91016100	0.67539500	1.26092900
H	4.83508200	-1.72261300	-1.73842400
O	0.21876200	-3.31372200	-1.73986500
N	5.25300300	0.08048000	0.16650000
C	6.39334500	-0.65362000	0.49272200
C	8.87200000	-1.63378800	1.25416200
C	6.56280300	-2.03873200	0.57487100
C	7.44047000	0.24122700	0.80618300
C	8.69015700	-0.25771400	1.18670800
C	7.81436300	-2.51034200	0.95553500

H	5.75120900	-2.72472100	0.35772500
H	9.50240400	0.42110900	1.42944900
H	7.97408700	-3.58160600	1.02565600
H	9.83547300	-2.03822900	1.54599600
C	5.55535900	1.43847500	0.26600300
C	6.65488600	3.96629200	0.56163600
C	4.74955000	2.54873600	-0.00239000
C	6.90482600	1.58002500	0.65938000
C	7.45422100	2.85701600	0.81041900
C	5.31881400	3.80778700	0.15435400
H	3.72034800	2.43901500	-0.32662300
H	8.49026900	2.97733800	1.11308800
H	4.71481600	4.68715400	-0.04563100
H	7.06434500	4.96447000	0.67556600
C	-4.78339200	0.71744300	-1.15093400
C	-7.26843000	2.14914000	-1.11685900
C	-5.05603600	1.75988800	-2.04603700
C	-5.80726500	0.42666000	-0.24675600
C	-7.02796700	1.11646900	-0.20840900
C	-6.26916400	2.46015500	-2.03383600
H	-4.30552200	2.01608400	-2.78784900
H	-6.42849900	3.25263100	-2.75721100
H	-8.20734500	2.69314700	-1.10660500
O	-5.74391000	-0.53420500	0.72934500
C	-6.93475800	-0.47405100	1.40844100
C	-9.40521200	-0.08032300	2.53423700
C	-7.77471500	0.51769200	0.88536600
C	-7.28229300	-1.28431200	2.47950800
C	-8.54107800	-1.06756300	3.03652000
C	-9.03351200	0.71986800	1.45816400
H	-6.60563300	-2.04210300	2.85783200
H	-8.85906700	-1.67564600	3.87704000
H	-9.70410500	1.48142600	1.07290900
H	-10.37758100	0.05829600	2.99490300

ODFRCZ-T_1 Geometry (Solvent: DCM)

H	-2.12761000	-2.83609800	-1.42600100
C	-2.20331700	-1.75384400	-1.38926700
C	-2.38943500	1.01696000	-1.35730300
C	-1.03652900	-0.99148800	-1.54551500
C	-3.43718300	-1.14518500	-1.19561900
C	-3.55107200	0.25323700	-1.17858300
C	-1.15306400	0.40591600	-1.53717400
H	-4.32516400	-1.75679100	-1.07226700
H	-0.27474100	1.02512600	-1.69640300
H	-2.45262700	2.10135900	-1.34803100
C	0.25124900	-1.69617300	-1.80664700
C	1.49829500	-1.20978000	-1.28311500
C	4.04355300	-0.38229200	-0.31798700
C	2.72664300	-1.82317600	-1.75697800
C	1.61820000	-0.20684400	-0.24636200
C	2.83751600	0.20159200	0.20250400
C	3.94357700	-1.42332100	-1.29530000
H	2.64578400	-2.58291400	-2.52433600
H	0.72397200	0.18979900	0.21987500
H	2.90890000	0.90819100	1.02378500
H	4.85315100	-1.83968500	-1.71720200
O	0.23439200	-2.76479000	-2.48746200
N	5.27491900	0.03278600	0.15446900
C	6.35717500	-0.79900700	0.49030100
C	8.69855400	-1.99102600	1.33400300
C	6.41392200	-2.19268900	0.51264300
C	7.43125000	0.00157000	0.93244600
C	8.61253100	-0.59674600	1.35340700
C	7.61055400	-2.77401800	0.92843600
H	5.56292600	-2.80588800	0.24082500

H	9.44636400	0.00452800	1.70128500
H	7.69172500	-3.85534800	0.94974600
H	9.61381000	-2.47564000	1.65657100
C	5.65685600	1.36518900	0.38384200
C	6.87682600	3.77995700	0.92237000
C	4.94727900	2.53469400	0.11028400
C	6.98077700	1.38735800	0.86975700
C	7.59537200	2.60255700	1.14554300
C	5.57623100	3.74336000	0.40421100
H	3.95762000	2.51464900	-0.33101200
H	8.61516000	2.63845300	1.51517500
H	5.05021300	4.67223500	0.21236500
H	7.33979000	4.73780200	1.13403800
C	-4.86408600	0.91843700	-1.01296800
C	-7.40065600	2.22807600	-0.69111300
C	-5.20581600	2.07493700	-1.72655400
C	-5.84950500	0.45273400	-0.13886000
C	-7.09317000	1.07696300	0.03767500
C	-6.44192400	2.71677200	-1.57282700
H	-4.49005400	2.47275800	-2.43978400
H	-6.65189200	3.60479800	-2.15966200
H	-8.35786200	2.72536100	-0.57195500
O	-5.72097200	-0.64495100	0.67487000
C	-6.89086200	-0.74008400	1.38322300
C	-9.33983000	-0.63080600	2.61588500
C	-7.78319400	0.28503100	1.04242000
C	-7.17566700	-1.72096000	2.32228600
C	-8.42424100	-1.64695100	2.93651700
C	-9.03080900	0.34208900	1.66999000
H	-6.45979400	-2.49948700	2.56048800
H	-8.69361200	-2.39160000	3.67842000
H	-9.74122900	1.12524600	1.42480800
H	-10.30264400	-0.60665200	3.11530400

ODFRCZ-S$_1$ Geometry (Solvent: DCM)

H	-2.27730500	-2.86739900	-1.23788800
C	-2.28616600	-1.78803300	-1.12428000
C	-2.31622100	0.97804400	-0.83687500
C	-1.06396800	-1.08683300	-1.05312400
C	-3.49474400	-1.11166800	-1.04455400
C	-3.53592000	0.28304900	-0.89680700
C	-1.10120000	0.31586700	-0.91703700
H	-4.42151600	-1.67233900	-1.10768600
H	-0.18065000	0.88911000	-0.89334400
H	-2.31902100	2.05753400	-0.71704100
C	0.18721400	-1.81384200	-1.16084500
C	1.53150600	-1.31798700	-0.81239600
C	4.11380900	-0.44561300	-0.16675200
C	2.64553500	-1.77639500	-1.54390700
C	1.73924800	-0.46193300	0.28624200
C	3.01531400	-0.01637100	0.59274600
C	3.92210400	-1.33426200	-1.23095700
H	2.49289800	-2.44443300	-2.38481200
H	0.90324600	-0.16296700	0.90959700
H	3.17492000	0.63811300	1.44396000
H	4.77280200	-1.65129600	-1.82571400
O	0.18940700	-3.04862500	-1.54498100
N	5.40754700	0.01179900	0.15393800
C	6.53632400	-0.78998500	0.32895400
C	9.01716800	-1.92490300	0.81397500
C	6.64679600	-2.18297600	0.29438200
C	7.64414500	0.03518300	0.62482400
C	8.89421100	-0.54068800	0.86564500
C	7.90125800	-2.73273300	0.53683900
H	5.78974500	-2.81692100	0.09503600
H	9.75291700	0.08346700	1.09436900

H	8.01658700	-3.81163600	0.51434000
H	9.98076700	-2.38866600	0.99668000
C	5.77952600	1.34591800	0.33059900
C	7.01236300	3.79643400	0.72220000
C	5.00800300	2.50437100	0.20453900
C	7.15988600	1.40228300	0.62480400
C	7.77720500	2.63974200	0.82548000
C	5.64447900	3.72415200	0.40884000
H	3.95464800	2.46134100	-0.04997500
H	8.83774000	2.69561700	1.05227500
H	5.06893000	4.63992700	0.31940700
H	7.47507700	4.76551000	0.87634800
C	-4.81826600	1.01750000	-0.83466500
C	-7.29685100	2.46505800	-0.69966500
C	-4.98549700	2.27506800	-1.43116000
C	-5.94792000	0.52391000	-0.17595000
C	-7.16654500	1.21322300	-0.09420600
C	-6.19213000	2.98411100	-1.36740500
H	-4.15201400	2.70290100	-1.98015300
H	-6.26221500	3.95054000	-1.85535500
H	-8.23082700	3.01522500	-0.64921600
O	-6.00363000	-0.67136700	0.49621400
C	-7.26917400	-0.76433700	1.01558100
C	-9.86296700	-0.62270300	1.89840800
C	-8.04151200	0.35878700	0.69149400
C	-7.74450500	-1.82828600	1.76867800
C	-9.06389600	-1.73617300	2.20725400
C	-9.36311300	0.43225600	1.14072300
H	-7.11684100	-2.68138400	2.00023200
H	-9.48150800	-2.54313800	2.80042700
H	-9.98437400	1.29022700	0.90367800
H	-10.88603500	-0.58739700	2.25767400

ODBTCZ-S_0 Geometry (Solvent: DCM)

H	-1.55017800	-3.50214400	-2.38184200
C	-1.78582000	-2.68158000	-1.71166700
C	-2.37588100	-0.53820300	-0.03304200
C	-0.81710400	-1.70012700	-1.47285800
C	-3.02983100	-2.60259000	-1.09989000
C	-3.34301400	-1.52618500	-0.25654100
C	-1.12055400	-0.62781700	-0.62713000
H	-3.77696500	-3.36762200	-1.28914600
H	-0.38566300	0.14975900	-0.44324700
H	-2.59684400	0.29474900	0.62743500
C	0.48943700	-1.80131600	-2.19364900
C	1.72887500	-1.24401800	-1.56790500
C	4.12083000	-0.28840300	-0.49232300
C	2.74601200	-0.77927900	-2.40982300
C	1.92525500	-1.23721200	-0.18250300
C	3.11703600	-0.76275900	0.35574500
C	3.93539400	-0.29873700	-1.87919900
H	2.59562700	-0.80778700	-3.48415400
H	1.15258700	-1.61144700	0.48165400
H	3.26837100	-0.74529100	1.43024600
H	4.73020000	0.05331300	-2.52908600
O	0.54539400	-2.33606400	-3.29120800
N	5.33065100	0.20050300	0.05156600
C	5.90465500	1.44028400	-0.22725500
C	7.40313400	3.76449400	-0.41032800
C	5.42922100	2.47399900	-1.03921700
C	7.10611800	1.56456100	0.50536000
C	7.86047400	2.73800000	0.40729400
C	6.19690300	3.63058300	-1.11997000
H	4.49535500	2.38487400	-1.58384200
H	8.78595100	2.84470800	0.96546200

H	5.85251400	4.44872900	-1.74455800
H	7.97664300	4.68115300	-0.49914000
C	6.15006900	-0.47580900	0.95461800
C	8.10782600	-1.40282500	2.68371300
C	6.00955500	-1.75974200	1.48890100
C	7.26444800	0.33721500	1.25996900
C	8.24804000	-0.13358600	2.13546700
C	7.00076000	-2.20519200	2.35654400
H	5.16506900	-2.39141000	1.23501800
H	9.10917600	0.48243500	2.37737000
H	6.91643700	-3.19792800	2.78714600
H	8.86101800	-1.78346500	3.36551000
C	-4.67628400	-1.45795000	0.39393600
C	-7.23244200	-1.39175100	1.62297600
C	-5.20247700	-2.57583900	1.04358100
C	-5.46615900	-0.29548900	0.36746100
C	-6.73981400	-0.25604900	0.96892800
C	-6.46150600	-2.54492300	1.65645500
H	-4.60717000	-3.48346100	1.08526900
H	-6.83235700	-3.43124000	2.16017500
H	-8.21152800	-1.37049500	2.09189700
C	-6.59524000	1.91516800	0.03116900
C	-9.08509300	2.73909500	0.92550400
C	-7.39227800	1.03051600	0.77947700
C	-7.02619500	3.20856000	-0.27534300
C	-8.27656700	3.61046200	0.17766500
C	-8.65053500	1.45566400	1.22703900
H	-6.40022300	3.88034200	-0.85368900
H	-8.63085400	4.61054000	-0.05028100
H	-9.27982400	0.78580200	1.80555700
H	-10.05820700	3.07313000	1.26991900
S	-5.06627100	1.21002700	-0.43212100

ODBTCZ-T_1 Geometry (Solvent: DCM)

H	1.61980200	-4.40397000	1.29551000
C	1.86957900	-3.41555400	0.92651200
C	2.43962100	-0.81156700	0.04457000
C	0.80707700	-2.45275700	0.81153700
C	3.14723500	-3.11013700	0.58128000
C	3.52886300	-1.77149500	0.14952500
C	1.15425200	-1.13583100	0.35405900
H	3.91095600	-3.86840400	0.70692700
H	0.38288400	-0.38145000	0.24848700
H	2.63337200	0.17170500	-0.36601900
C	-0.53141500	-2.85284600	1.20975300
C	-1.72053000	-2.00778900	0.86610600
C	-4.03773400	-0.55703200	0.26975800
C	-2.74478100	-1.88350900	1.81222900
C	-1.88277500	-1.41552900	-0.39171200
C	-3.03166600	-0.68908700	-0.68994400
C	-3.89618500	-1.16173600	1.52209800
H	-2.63252300	-2.37010100	2.77584500
H	-1.11485700	-1.53040700	-1.15120100
H	-3.15096500	-0.21931800	-1.66131100
H	-4.69398800	-1.07328300	2.25312700
O	-0.71118700	-3.90024600	1.85908900
N	-5.20707400	0.18492200	-0.02820800
C	-5.71309300	1.23963200	0.72739600
C	-7.07740600	3.37889400	1.84268300
C	-5.19963100	1.82043100	1.89079000
C	-6.88728600	1.72675800	0.10980200
C	-7.57346000	2.80489400	0.67826600
C	-5.89973500	2.89072900	2.43603000
H	-4.28788100	1.45547800	2.35154400
H	-8.47733000	3.18779100	0.21350900
H	-5.52492700	3.35984500	3.34026900

H	-7.59838400	4.21478600	2.29764300
C	-6.04075000	-0.01479100	-1.12574000
C	-7.99828600	-0.03455800	-3.08780200
C	-5.95750500	-0.97695500	-2.13659000
C	-7.09787800	0.92209600	-1.07726000
C	-8.08168600	0.90946700	-2.07134400
C	-6.94751800	-0.96871700	-3.11262200
H	-5.15577000	-1.70735500	-2.15898300
H	-8.89886800	1.62444100	-2.04511800
H	-6.90725200	-1.70384800	-3.91022000
H	-8.75231300	-0.05740400	-3.86756600
C	4.86127200	-1.46225000	-0.15521700
C	7.55839100	-0.93103200	-1.04412300
C	5.80406600	-2.51479900	-0.49863000
C	5.42158200	-0.12159500	-0.19450400
C	6.72644400	0.12743600	-0.64131000
C	7.07405300	-2.25266300	-0.94506500
H	5.46342300	-3.54173900	-0.48159600
H	7.71524500	-3.07769400	-1.23641100
H	8.56870800	-0.73869800	-1.38787400
C	6.05882300	2.28829200	0.04427100
C	8.41423300	3.53049600	-0.69598600
C	7.09016400	1.52768400	-0.53525900
C	6.18413900	3.66499600	0.26001100
C	7.37083500	4.27592100	-0.11639900
C	8.28333400	2.16627800	-0.90524800
H	5.37681600	4.23478800	0.70780200
H	7.49544200	5.34249700	0.03831400
H	9.09339700	1.59916900	-1.35329800
H	9.33253500	4.03207300	-0.98263200
S	4.68085300	1.31049700	0.43915600

ODBTCZ-S_1 Geometry (Solvent: DCM)

H	1.60843800	-3.54773000	2.34732300
C	1.84353900	-2.72329000	1.68217100
C	2.45365500	-0.62172800	-0.03927700
C	0.84481000	-1.78781000	1.34731600
C	3.12165900	-2.60436800	1.15256100
C	3.45031300	-1.55769300	0.28117600
C	1.17477700	-0.72162100	0.48866300
H	3.88211000	-3.32971700	1.42765400
H	0.43649100	0.03543000	0.24720900
H	2.67791700	0.19310500	-0.72149400
C	-0.48773500	-1.90840900	1.93621900
C	-1.74012800	-1.35375000	1.40980200
C	-4.17063300	-0.34846600	0.43922100
C	-2.80102800	-1.06925600	2.29128200
C	-1.92973200	-1.15884400	0.02760700
C	-3.12368000	-0.63729800	-0.44588300
C	-4.00686800	-0.57906600	1.80676900
H	-2.67240900	-1.22396100	3.35701800
H	-1.14355100	-1.41821600	-0.67293100
H	-3.25346500	-0.45351400	-1.50792500
H	-4.82734800	-0.37560500	2.48787700
O	-0.59452400	-2.58876900	3.03222400
N	-5.39051300	0.17202900	-0.05201000
C	-6.02450800	1.32714400	0.40267800
C	-7.63048000	3.52896900	0.91135500
C	-5.61937200	2.23083700	1.38945200
C	-7.20971900	1.52386000	-0.34116300
C	-8.01833500	2.63412300	-0.07873800
C	-6.43972200	3.32740600	1.63108700
H	-4.69826700	2.08979400	1.94475500
H	-8.93218900	2.79453100	-0.64320800
H	-6.14977600	4.04418700	2.39286500

H	-8.24677300	4.39488700	1.12923500
C	-6.15376200	-0.37734900	-1.08023900
C	-8.01341600	-1.07805300	-3.01285000
C	-5.94122700	-1.54941700	-1.81225800
C	-7.29234300	0.43268700	-1.29119900
C	-8.22602200	0.07672700	-2.26929900
C	-6.88399300	-1.88195100	-2.77888400
H	-5.07948300	-2.18305800	-1.63173100
H	-9.10429800	0.69206400	-2.44112200
H	-6.74332700	-2.78595700	-3.36289100
H	-8.72716700	-1.36832100	-3.77665800
C	4.81394300	-1.46500900	-0.29511300
C	7.43687400	-1.35372500	-1.38077500
C	5.43325700	-2.59624300	-0.83179300
C	5.54737100	-0.26492700	-0.31122000
C	6.85037300	-0.20137000	-0.84363200
C	6.72495900	-2.54468400	-1.37055500
H	4.88466000	-3.53363900	-0.84440400
H	7.16712900	-3.44470600	-1.78463700
H	8.44074600	-1.31536700	-1.79263800
C	6.54818500	2.02044600	-0.07689600
C	9.04090900	2.90875500	-0.89936000
C	7.42798400	1.12790700	-0.71522900
C	6.89799600	3.35339300	0.15497500
C	8.15061300	3.78736200	-0.26073100
C	8.68663500	1.58608500	-1.12770100
H	6.20838800	4.03126600	0.64756300
H	8.44251100	4.81840200	-0.08931900
H	9.37867800	0.91138000	-1.62302800
H	10.01398400	3.26843000	-1.21709500
S	5.03224200	1.27025100	0.35603900

C1-S_0 Geometry (Solvent: THF)

H	2.50949000	0.86604400	-0.28800200
C	2.46896400	-0.17767500	0.00901600
C	2.34562300	-2.88300600	0.68325400
C	1.23047600	-0.79008400	0.22159800
C	3.64152600	-0.91057500	0.17210700
C	3.58138300	-2.26575700	0.51425500
C	1.16856600	-2.14764300	0.55265800
H	4.50305400	-2.82362500	0.64890200
H	0.20848500	-2.63550400	0.68568000
H	2.30032600	-3.93788200	0.93156600
C	-0.00180900	0.03067100	-0.00632000
C	-1.25611900	-0.28296400	0.75038800
C	-3.64722400	-0.70987400	2.12473700
C	-2.47602100	0.00441900	0.13226400
C	-1.23305600	-0.79064100	2.05328200
C	-2.43062700	-0.99817900	2.73598500
C	-3.66904400	-0.21435500	0.81650600
H	-2.48696700	0.38809900	-0.88343300
H	-0.28736300	-0.99814600	2.54340900
H	-2.41578600	-1.37220300	3.75400700
O	0.01432100	0.95307700	-0.80513200
H	-4.58266900	-0.85269800	2.65697600
N	-4.90552800	0.07429900	0.19105800
C	-5.25476800	1.28875900	-0.39677100
C	-6.42415400	3.47319900	-1.63748900
C	-4.51900700	2.47403700	-0.48430600
C	-6.56841800	1.19005700	-0.90745100
C	-7.15329900	2.29463600	-1.53500400
C	-5.12267700	3.55772000	-1.11245500
H	-3.51599700	2.55202300	-0.07815600
H	-8.16224300	2.23117900	-1.93201100
H	-4.57346400	4.49006200	-1.19705100

H	-6.86189400	4.33909900	-2.12296300
C	-5.97718900	-0.80647800	0.06302700
C	-8.33290200	-2.16491700	-0.47190700
C	-7.03052900	-0.15214000	-0.61450500
C	-6.07888700	-2.14178100	0.46373300
C	-7.26962000	-2.80506100	0.18845800
C	-8.21809800	-0.84126200	-0.87962900
H	-5.26074900	-2.64780800	0.96533100
H	-7.37662300	-3.84247600	0.48923800
H	-9.03459500	-0.34916800	-1.39984900
H	-9.24780800	-2.71378700	-0.66871000
N	4.89828900	-0.28482200	-0.01272300
C	5.90474000	-0.72307400	-0.87017100
C	8.16029300	-1.15536200	-2.42046300
C	5.90531200	-1.80730300	-1.75234900
C	7.00883700	0.15268600	-0.76783400
C	8.14584400	-0.07143900	-1.55073300
C	7.04730600	-2.00873400	-2.51950900
H	5.04739000	-2.46514000	-1.84304700
H	9.00076800	0.59479200	-1.48182800
H	7.07602400	-2.84332500	-3.21288400
H	9.03466500	-1.34403800	-3.03442100
C	5.33978400	0.86276100	0.64195200
C	6.67498600	3.05423700	1.68760200
C	6.64755200	1.16768100	0.20181100
C	4.69262700	1.63240800	1.61271600
C	5.37842500	2.72888400	2.12333400
C	7.31595500	2.27638100	0.73087600
H	3.69472100	1.38592500	1.95968400
H	4.89931300	3.34582800	2.87694200
H	8.32103800	2.52153700	0.40030800
H	7.17848100	3.91887500	2.10706300

C1-T_1 Geometry (Solvent: THF)

H	2.38928000	0.63419900	-0.35335600
C	2.44127500	-0.41249500	-0.06898600
C	2.57734900	-3.12113100	0.55651000
C	1.25104600	-1.14221400	0.06782400
C	3.67478200	-1.02149800	0.14465100
C	3.75354500	-2.37878100	0.46878800
C	1.33947700	-2.51215600	0.36346300
H	4.72203800	-2.83769900	0.64180200
H	0.43762400	-3.11526200	0.40910000
H	2.62772000	-4.18277900	0.77727100
C	-0.03479400	-0.44120300	-0.21981400
C	-1.24474900	-0.79037800	0.47702200
C	-3.75285300	-1.41404500	1.75144100
C	-2.45714000	-0.23541800	0.00650100
C	-1.33317500	-1.60315700	1.67111500
C	-2.54963200	-1.89494900	2.26868900
C	-3.67325700	-0.54993400	0.61088500
H	-2.43033100	0.36753800	-0.89358800
H	-0.42452100	-1.96529600	2.13839300
H	-2.56352900	-2.48512300	3.17940800
O	-0.03037600	0.48783000	-1.08518200
H	-4.69443500	-1.55777900	2.26575300
N	-4.84642200	0.00726900	0.12050800
C	-5.01346300	1.33782700	-0.30983700
C	-5.87343600	3.82160700	-1.14339000
C	-4.09977700	2.39179900	-0.28409200
C	-6.35714200	1.52090400	-0.69847700
C	-6.79301700	2.76644200	-1.12177000
C	-4.55296200	3.63572200	-0.72273800
H	-3.08424500	2.26451900	0.06994600
H	-7.82447800	2.92446600	-1.41893000
H	-3.86535000	4.47400100	-0.72685900

H	-6.19471300	4.80297900	-1.47534800
C	-6.07572300	-0.66264600	-0.00853200
C	-8.64043700	-1.53428400	-0.51547500
C	-7.03572500	0.24100100	-0.50900700
C	-6.35942100	-2.01517200	0.18419900
C	-7.66805900	-2.43046100	-0.06063200
C	-8.32806700	-0.19142300	-0.76012500
H	-5.59702800	-2.72185400	0.48859600
H	-7.92707800	-3.47270800	0.08849800
H	-9.07787900	0.48867200	-1.15051600
H	-9.64707600	-1.89097400	-0.70509800
N	4.86746200	-0.25692300	0.02931400
C	5.94294400	-0.55340500	-0.80037600
C	8.28844700	-0.69376100	-2.27185400
C	6.09050100	-1.59933600	-1.71680100
C	6.94667200	0.42689300	-0.62456800
C	8.12903600	0.34981900	-1.36814000
C	7.27439800	-1.65344900	-2.44325400
H	5.30906800	-2.33823600	-1.85961200
H	8.90722500	1.09714300	-1.24237000
H	7.41605300	-2.45480100	-3.16169000
H	9.19976000	-0.76882200	-2.85593000
C	5.16171500	0.90161200	0.73951900
C	6.21690900	3.18145400	1.91275300
C	6.44578400	1.35983900	0.36410400
C	4.40013700	1.56364500	1.70765200
C	4.94608000	2.70541100	2.28286000
C	6.97203200	2.51211200	0.95737400
H	3.42000100	1.20111900	1.99893700
H	4.37576000	3.24063600	3.03564500
H	7.95670000	2.87438300	0.67632300
H	6.60938600	4.07787300	2.38144600

C1-S_1 Geometry (Solvent: THF)

H	2.48523300	0.78108200	-0.17541100
C	2.47220500	-0.25015600	0.16238600
C	2.47264600	-2.90945100	1.00011400
C	1.25499100	-0.88934000	0.45970200
C	3.67347600	-0.94106300	0.31025700
C	3.68518700	-2.26970200	0.73901100
C	1.26401200	-2.23873500	0.86178300
H	4.63011100	-2.78768000	0.86609900
H	0.33167700	-2.76197900	1.04139300
H	2.47134100	-3.94702600	1.31773800
C	-0.00008900	-0.15806200	0.26811800
C	-1.29171900	-0.45337000	0.90309500
C	-3.78163500	-0.95123600	2.09767200
C	-2.47820200	-0.08161100	0.24808400
C	-1.36179300	-1.06504700	2.16914000
C	-2.60076200	-1.32347000	2.74188500
C	-3.71037200	-0.32757700	0.85077200
H	-2.44440900	0.36857000	-0.73888000
H	-0.45330100	-1.31224800	2.70681500
H	-2.64812600	-1.79485300	3.71807500
O	0.02018400	0.84475900	-0.54766100
H	-4.74824200	-1.12417600	2.55953600
N	-4.89960300	0.05561400	0.18234500
C	-5.19347700	1.33461100	-0.28401000
C	-6.24800300	3.66853400	-1.34172800
C	-4.44101800	2.50820300	-0.18163000
C	-6.46799100	1.31930400	-0.89427200
C	-6.99463200	2.49964200	-1.42861200
C	-4.98680200	3.66813000	-0.72010900
H	-3.46954500	2.51808900	0.30132200
H	-7.97299600	2.50127100	-1.90018200
H	-4.42403900	4.59406900	-0.65645200

H	-6.64097500	4.59261600	-1.75250500
C	-5.96684600	-0.78526300	-0.12214000
C	-8.29114300	-2.03204800	-0.97214400
C	-6.96284800	-0.03920000	-0.79211800
C	-6.10978000	-2.15530300	0.11578200
C	-7.28402000	-2.76177800	-0.31622900
C	-8.13514000	-0.67284800	-1.21655800
H	-5.33426000	-2.72740200	0.61403600
H	-7.42273400	-3.82445200	-0.14399400
H	-8.90808100	-0.11085700	-1.73259400
H	-9.19489600	-2.53881900	-1.29388000
N	4.89277600	-0.28152800	0.01776900
C	5.85902500	-0.72748100	-0.88055900
C	8.02467700	-1.15809100	-2.55421700
C	5.85044600	-1.86173800	-1.69737300
C	6.92687100	0.19766400	-0.90384200
C	8.01889200	-0.02566700	-1.74816000
C	6.94720700	-2.06093700	-2.52855500
H	5.01930100	-2.55891300	-1.68916000
H	8.84605300	0.67762700	-1.77529600
H	6.96817700	-2.93371900	-3.17349000
H	8.86455100	-1.34698800	-3.21453600
C	5.32310700	0.92065300	0.57385300
C	6.62358900	3.22409700	1.39996000
C	6.58413800	1.25290200	0.02902200
C	4.70710900	1.71932900	1.54171200
C	5.37479100	2.87193300	1.94115900
C	7.23509600	2.41742500	0.44742400
H	3.74680200	1.45162500	1.96959100
H	4.91915300	3.51214600	2.68988100
H	8.20429400	2.68362000	0.03598900
H	7.11405700	4.13221800	1.73435300

C2-S_0 Geometry (Solvent: THF)

H	-2.32107100	-0.43566400	-1.07671300
C	-2.33301700	-0.01066000	-0.07775500
C	-2.37140300	1.11718000	2.47811500
C	-1.13278200	0.20156500	0.61158500
C	-3.54005200	0.31996800	0.52214600
C	-3.56448500	0.88638400	1.80050300
C	-1.15550400	0.76686500	1.88982100
H	-4.52030500	1.14316000	2.24900300
H	-0.22687300	0.95656000	2.41900300
H	-2.38576600	1.56885300	3.46415700
C	0.14781300	-0.12659300	-0.09233200
C	1.34543400	-0.55242100	0.70077600
C	3.63817000	-1.40742200	2.04791000
C	2.61013600	-0.26509100	0.18078400
C	1.23185700	-1.27789200	1.89311100
C	2.37662100	-1.71013500	2.55871300
C	3.74988800	-0.68462600	0.86077400
H	2.70034900	0.28515300	-0.75200200
H	0.25202600	-1.52570600	2.28898500
H	2.28659100	-2.28403100	3.47463700
O	0.21592900	-0.04801000	-1.30795800
H	4.54213500	-1.72733600	2.55678900
N	5.04945800	-0.37958800	0.34610600
N	-4.76338600	0.08140900	-0.18108500
C	5.57273500	0.91130600	0.55776600
C	6.74101700	3.43806100	0.94693500
C	4.95317700	1.86279600	1.36997000
C	6.78909400	1.24954100	-0.05496800
C	7.36467500	2.49528800	0.12555000
C	5.53921500	3.11579400	1.56698100
H	4.01524900	1.62096000	1.85742900

H	8.30480900	2.70663000	-0.37326500
H	5.04304700	3.83594400	2.20891500
H	7.19731100	4.41052300	1.09546900
C	5.52634800	-1.10339300	-0.76530600
C	6.60632900	-2.49994100	-2.95259700
C	6.74324300	-0.71348800	-1.34499500
C	4.86231200	-2.20879800	-1.29998800
C	5.40418600	-2.90458300	-2.38356300
C	7.27559600	-1.39140700	-2.42801200
H	3.92379600	-2.53265300	-0.86408500
H	4.87272100	-3.76425000	-2.77764900
H	8.21822200	-1.04645200	-2.84013400
H	7.02795300	-3.03597500	-3.79571000
O	7.47659100	0.32936100	-0.81809500
C	-5.55416300	-1.02100300	0.20051700
C	-7.20048600	-3.20654900	0.84655400
C	-6.82670900	-1.16479400	-0.37329200
C	-5.12118800	-1.99511700	1.10215800
C	-5.93888300	-3.08330600	1.41727500
C	-7.64528300	-2.23393600	-0.05238300
H	-4.13826800	-1.90889300	1.55183900
H	-5.57697200	-3.83137900	2.11462200
H	-8.62093200	-2.29603000	-0.52312400
H	-7.83864600	-4.04878300	1.09015200
C	-5.41734600	1.17583800	-0.78283200
C	-6.79725200	3.27087400	-2.05096400
C	-4.84403400	2.44452100	-0.88518900
C	-6.69347800	0.97610300	-1.33100400
C	-7.38168900	2.00610000	-1.94868300
C	-5.52945900	3.48266600	-1.52104900
H	-3.85476100	2.61922100	-0.47706500
H	-8.36658600	1.79842300	-2.35392500
H	-5.05948800	4.45753400	-1.59567000
H	-7.33283900	4.07488600	-2.54355200
O	-7.28351800	-0.27030100	-1.31787900

C2-T_1 Geometry (Solvent: THF)

H	2.28318100	-0.23438800	0.92621700
C	2.37561900	0.22039100	-0.05485500
C	2.63636400	1.43685100	-2.54447500
C	1.20938500	0.56441500	-0.76738600
C	3.63218300	0.45449800	-0.59252600
C	3.78115200	1.06063800	-1.84353100
C	1.36967000	1.19397800	-2.01601000
H	4.77568100	1.23977600	-2.24212900
H	0.49686300	1.53427000	-2.56400600
H	2.73103300	1.93540000	-3.50451100
C	-0.09976400	0.32759900	-0.11067400
C	-1.29523300	0.05475900	-0.87888600
C	-3.76355200	-0.56740100	-2.20927100
C	-2.54023500	0.07315100	-0.18520000
C	-1.34241400	-0.32624900	-2.25387600
C	-2.53497700	-0.62319200	-2.89056200
C	-3.70794100	-0.22182100	-0.85714700
H	-2.55273300	0.32592500	0.86980500
H	-0.41946000	-0.42970600	-2.81331300
H	-2.52048400	-0.92129600	-3.93461900
O	-0.14880400	0.35173200	1.16330900
H	-4.70811600	-0.79598500	-2.68996100
N	-4.95345000	-0.17367500	-0.11418400
N	4.79668700	0.06381700	0.15167900
C	-5.72203300	0.97149400	-0.13727600
C	-7.35605600	3.24040400	-0.11152000
C	-5.33278000	2.12520900	-0.84904900
C	-6.93668600	0.99145000	0.58256400
C	-7.75322300	2.12066200	0.59628100

C	-6.14491100	3.23956300	-0.83156200
H	-4.39792500	2.11815300	-1.39644000
H	-8.67628700	2.08990400	1.16364200
H	-5.84336700	4.12594000	-1.37766200
H	-7.98151000	4.12584600	-0.10838600
C	-5.36786200	-1.27552600	0.60478900
C	-6.30441500	-3.44782700	2.09510600
C	-6.58844100	-1.20703500	1.31217100
C	-4.61786500	-2.46900300	0.65331200
C	-5.08687400	-3.53531300	1.39109200
C	-7.05658800	-2.28800400	2.05657600
H	-3.68087100	-2.52733300	0.11271400
H	-4.50784400	-4.45073700	1.42919800
H	-7.99763100	-2.18900800	2.58524400
H	-6.65643400	-4.29565200	2.67175200
O	-7.34900600	-0.08740100	1.28866400
C	5.47646700	-1.10701600	-0.22672500
C	6.89900700	-3.44608000	-0.87716000
C	6.69924400	-1.41024000	0.39341100
C	4.97955900	-2.00197400	-1.17709000
C	5.68489700	-3.16554200	-1.49396000
C	7.40806900	-2.55459900	0.07112300
H	4.03340000	-1.78931900	-1.66208600
H	5.27327600	-3.84835500	-2.22986100
H	8.34899500	-2.73865700	0.57944700
H	7.45045300	-4.34715700	-1.12234100
C	5.52939200	1.05315200	0.83070100
C	7.05477600	2.94255400	2.25138600
C	5.08601100	2.37083100	0.96421400
C	6.75067800	0.70079500	1.42674000
C	7.51013500	1.62792000	2.11922100
C	5.84257000	3.30565600	1.67515800
H	4.14075500	2.65989700	0.51855200
H	8.44740300	1.30216400	2.55859500
H	5.47190300	4.32075300	1.77188200
H	7.64550100	3.66619600	2.80233600
O	7.21050900	-0.59964500	1.38588300
